# Enhanced three-dimensional visualization reconstruction for perforator flaps: A case series on clinical applications and outcomes

**DOI:** 10.1016/j.jpra.2026.04.015

**Published:** 2026-05-14

**Authors:** Yin Qixiang, Cai Huazhong, Zhou Feng, Yao Qun, Hua Yong, Mi Jingyi

**Affiliations:** a Suzhou Medical College of Soochow University, Suzhou, Jiangsu Province, 215123, China; b Department of Sports Medicine, Wuxi Ninth People’s Hospital Affiliated to Soochow University, Wuxi, Jiangsu Province, 214062, China; c Department of Emergency, Affiliated Hospital of Jiangsu University, Zhenjiang, Jiangsu Province, 212001, China

**Keywords:** Three-dimensional visualization, Perforator flap, Computed tomography angiography (CTA), Digital reverse flap design, Surgical guide, Accuracy

## Abstract

**Introduction:**

Perforator flaps play a vital role in soft tissue repair; however, they face challenges due to anatomical variability. Although computed tomography angiography (CTA)-based three-dimensional visualization can assist in surgical planning, it is limited by image quality and vessel extraction accuracy. This study presents advanced 3D techniques designed to enhance accuracy and evaluates their clinical outcomes.

**Methods:**

This case series investigated the clinical applications and outcomes of enhanced three-dimensional visualization for anterolateral thigh perforator (ALTP) flaps in 12 patients (13 flaps) treated between January 2021 and December 2024. Technical enhancements included: 1) optimized CTA protocols (adjusted voltage based on body mass index, high-contrast injection, and nitroglycerin administration); 2) an "Indirect Extraction Method" with a reduced threshold (105–115 HU) for small vessel reconstruction; 3) manual tracing of perforators; 4) "Digital Reverse Flap Design" (an expanded wound model projected onto the donor site); and 5) 3D-printed surgical guides for enhanced precision. The outcomes assessed were perforator accuracy, flap fit, survival, and complications.

**Results:**

All flaps were successfully transplanted in the present study. Perforator localization was excellent (<1 cm) (81.3%, n = 13/16) and moderate (1–2 cm) (18.8%, n = 3/16). Surgical findings were consistent with 3D planning in 100% of cases regarding perforator type, source artery, and pedicle route. The flap fit was excellent (84.6%, n = 11/13) and moderate (15.4%, n = 2/13). The survival rate was 100% (n = 13/13). Foot function, assessed by the American Orthopaedic Foot & Ankle Society (AOFAS) score, was good in both cases. Hand function, measured by the Michigan Hand Outcomes Questionnaire (MHQ), was rated as excellent or good in 60% (n = 6/10) of cases. Complications occurred in two cases (12.5%), including infection (n = 1) and delayed fracture healing (n = 1).

**Conclusion:**

The advanced 3D technique enhances perforator localization, flap design, and surgical precision, resulting in high success rates. Limitations of the present study include a small sample size and its retrospective design.

Perforator flaps, pioneered by Koshima et al. beginning in 1989,**^[^**^1,^^2^**^]^** are important for complex soft tissue reconstruction.**^[^**^3,^^4^**^]^** However, their use faces challenges due to anatomical variations in perforator vessels, which increase surgical risks. Indeed, inaccurate preoperative localization may lead to intraoperative failure, prolonged surgery, donor-site morbidity, and suboptimal outcomes.**^[^**5, 6, 7**^]^** While Computed Tomography Angiography (CTA)-based 3D visualization**^[^**^8^**^]^** provides objective models for preoperative planning, its clinical application is limited. Conventional CT resolution typically exhibits limited ability to depict perforators <0.5 mm,**^[^**^9^**^]^** and suboptimal scanning protocols can further degrade image quality.**^[^**^10^**^]^** Standard vessel extraction techniques often fail to adequately isolate small perforators from adjacent tissues, resulting in inaccurate models. Existing digital flap design methods rely on oversimplified wound measurements, neglecting 3D geometric complexities and result in poor flap-wound congruence.**^[^**^11^**^]^** Indeed, transferring virtual designs to the operative field remains error-prone with manual marking or flexible templates; meanwhile, emerging mixed reality solutions face technical barriers such as registration inaccuracy.**^[^**^12^**^]^**

To address these limitations, an enhanced 3D visualization protocol was developed and evaluated in a retrospective case series. By incorporating high-fidelity wound modeling, 3D-printed surgical guides, and augmented reality verification, the protocol ensured optimal flap-wound congruence and precise intraoperative transfer. Preliminary clinical results confirmed its efficacy in overcoming the core limitations of conventional CTA-based planning. .

## Patients and methods

This study was conducted and reported in accordance with the Strengthening the Reporting of Observational Studies in Epidemiology (STROBE) guidelines. The completed checklist is provided in the Supplemental Appendix.

### Study design and setting

This retrospective case series was conducted at two tertiary hospitals. The study protocol received approval from the respective Institutional Review Boards, and all patients provided written informed consent.

### Patient population

In the present study, the medical records of 12 consecutive patients (13 flaps) with traumatic soft tissue defects who underwent ALTP perforator flap reconstruction using an enhanced 3D visualization protocol between January 2021 and December 2024 were reviewed. Patient demographics and flap characteristics are summarized in Table 1.

**Table 1:** Patient demographics and flap characteristics.

Demographics	Total population, n = 12
Sex	
Men	9
Women	3
Median age, years	45.0 ± 13.0
Injury cause, n(%)	
Machinery crush	9(75.0)
Traffic accident	2(16.7)
Thermal crush	1(8.3)
Time from injury to flap surgery, day	14.2 ± 6.0
Wound location, n(%)	
Hand	10(83.3)
Foot	2(16.7)
Wound area, cm^2^	112.2 ± 56.8
Harvested flap area (cm×cm) Reported as range	(9 × 6-20 × 15)
Flap donor site repair method, n(%)	
Primary closure	8 (66.7)
skin grafting	4 (33.3)

Abbreviations: ALTP, Anterolateral Thigh Perforator, IQR, interquartile range.

Note: One patient, who sustained severe trauma to the left hand requiring extensive soft tissue coverage, underwent reconstruction with two ALTP flaps to address the defect areas.

### Follow-up protocol

Patients were followed up monthly for the first three postoperative months, with subsequent periodic assessments conducted via clinical visits or digital communication platforms (WeChat).

### Imaging protocol

CTA was performed using either a GE Revolution CT scanner at Jiangsu University's Affiliated Hospital or a Siemens SOMATOM Definition Flash scanner at Wuxi Ninth People's Hospital, Soochow University.

Recipient Site: Scans encompassed the forearm and distal limb segment, starting from the mid-calf level adjacent to the wound.

Donor Site: For all patients, the inguinal region and thigh were scanned for ALTP flap planning.

A standardized CTA protocol was implemented, as detailed in Table 2.

**Table 2:** CTA scanning protocol.

Step	Parameter	Specification
1	Pharmacological Preparation	Administer 0.5 mg nitroglycerin**^[^**^38,^^39^**^]^** sublingually 5 min prior to scan.
2	Patient Positioning	Supine, legs extended, feet apart at shoulder width.
3	Bolus Timing	Perform a small bolus test to determine peak arterial enhancement time.
4	Contrast Injection	Inject contrast medium (350 mg I/mL) at 5.0 mL/s, followed by 40 mL saline flush.
5	Scan Trigger	Initiate scanning at the peak time identified from the bolus test.
6	Technical Parameters	Slice thickness: 1 mm; Reconstruction thickness: 0.625 mm; Matrix: 512 × 512; Tube voltage: 100 kV (BMI < 25) or 120 kV (BMI ≥ 25).

### 3D reconstruction and vessel extraction

1. Recipient Site Modeling and Wound Surface Creation
  CTA data from the recipient site were imported into Mimics software for 3D reconstruction. The model was subsequently transferred to 3-matic software, where the precise tissue defect area was demarcated using the "Lasso Area Mark" tool to generate a detailed wound surface model. This model was subsequently imported into SolidWorks and converted into a 2D plane using the "Surface Flattening" function.
2. Donor Site Modeling and Vessel Extraction
  Donor site CTA data were imported into Mimics software to reconstruct bones, muscles, and skin. A systematic post-processing protocol was employed to enhance the reconstruction of small-calibre vessels:

Threshold Setting: A lower Hounsfield Unit (HU) threshold, ranging from 105 to 115, was applied to maximize the capture of small vessels.

Indirect Extraction Method: Tissues with CT values from 110 to 2404 were initially extracted. Cortical bone (HU 450-2404) was then subtracted using Boolean operations, isolating structures with HU values between 110 and 449 (vessels and cancellous bone). Region growing was applied to tissues ≥110 HU to obtain a preliminary vascular structure.

Manual Perforator Tracing: Perforator vessels (typically HU 50-80), often below the automated extraction threshold, were meticulously identified on sequential CTA cross-sections. These were manually traced and reconstructed into 3D models using the "Thin Structure" tool in Mimics, accurately mapping their course from source vessels to the superficial fascia.

### Flap design and surgical planning

1. Flap Design
  The 2D wound plane model was expanded by 10-15% and projected onto the 3D donor site model. The projected area was positioned to ensure inclusion of the deep fascial exit points of the target perforator(s). The positioning was optimized based on key surgical considerations, including pedicle length, donor site morbidity, and scar placement.
2. Surgical Planning
  A 3D flap model was created, with its thickness adjusted to match the recipient wound depth. Virtual transfer to the recipient site was performed to verify pedicle length for anastomosis, recipient vessel compatibility, and complete wound coverage.
3. 3D-printed Guide Fabrication
  A coordinate system was defined using anatomical landmarks (anterior superior iliac spine, patella). The location of the perforator's deep fascial exit point (point P) was recorded within this system as a radial distance and angle from the origin. A patient-specific, 1:1-scale 3D-printed surgical guide was fabricated, incorporating alignment slots and a central hollow corresponding to the flap design and perforator location.

The systematic workflow for 3D reconstruction and preoperative planning is outlined below and summarized in Table 3.

**Table 3:** Detailed workflow for 3D reconstruction and preoperative planning.

Step	Procedure	Software / Action
1. Recipient Site & Wound Modeling		
1.1	Import recipient site CTA data and perform 3D reconstruction.	Mimics
1.2	Transfer the 3D model and precisely demarcate the tissue defect area.	3-matic ("Lasso Area Mark" tool)
1.3	Construct a detailed 3D model of the wound surface.	3-matic
1.4	Flatten the 3D wound surface model into a 2D plane.	Solidworks ("Surface Flattening" tool)
2. Donor Site Modeling & Vessel Extraction		
2.1	Import donor site CTA data and reconstruct bones, muscles, and skin.	Mimics
2.2	Vessel Extraction Strategy:	
	a. Set a lower Hounsfield Unit (HU) threshold between 105 and 115 to capture small vessels.	Mimics
	b. Employ "Indirect Extraction Method": Extract tissues (CT: 110-2404), subtract cortical bone (CT: 450-2404) to isolate vessels & cancellous bone (CT: 110-449). Apply region growth (≥110).	Mimics (Boolean Operations, Region Growing)
	c. Manually identify and trace perforator vessels (CT: 50-80) across sequential CTA cross-sections.	Mimics ("Thin Structure" tool)
3. Flap Design & Simulation		
3.1	Expand the 2D wound plane model by 10-15%.	3-matic
3.2	Project the expanded model onto the 3D donor site to define the flap harvest area, ensuring it encompasses the target perforator(s).	3-matic (Project Mesh command)
3.3	Create a 3D flap model and adjust its thickness to match the wound depth.	3-matic
3.4	Simulate the transfer of the flap and pedicle to the recipient site to verify length, fit, and vessel compatibility.	3-matic
4. Surgical Guide Preparation		
4.1	Define a coordinate system on the donor site model using anatomical landmarks (e.g., ASIS and patella).	3-matic
4.2	Record the precise location (distance and angle) of the perforator's exit point (Point P).	3-matic
4.3	Design and fabricate a patient-specific, 1:1 scale 3D-printed surgical guide with alignment features.	3-matic

### Surgical procedure

The surgical procedure was performed according to the preoperatively defined resection area and perforator locations. The recipient site was first prepared by exposing the corresponding recipient vessels. The initial incision was made along the marked flap border, and the flap was elevated from the deep fascial plane. A circumferential fascial incision was then made, maintaining a 2-cm safety margin around the identified target perforator. Following localization, the perforator was dissected retrograde to its source artery, and its anatomical type and origin were documented. After confirmation of robust flap perfusion, the flap was harvested with an adequate vascular pedicle. The free flap was then transplanted to the recipient site, and microvascular anastomoses were performed to complete the reconstruction. (All contents about sural neurocutaneous flaps are deleted in this paragraph.)

### Postoperative care

Postoperative management focused on optimizing flap viability and preventing complications. Core principles included maintaining patient warmth and hydration to support peripheral perfusion, alongside protocols to prevent vasospasm, manage analgesia, and guard against infection. Flap perfusion was monitored closely for the first postoperative week, with any vascular compromise addressed immediately. For patients with lower limb immobilization, deep vein thrombosis (DVT) prophylaxis was instituted using subcutaneous heparin and compression therapy.**^[^**^13^**^]^** Follow-up was scheduled with monthly clinic visits for the first three months, transitioning to periodic assessments conducted either in person or via remote telemedicine consultations.

### Outcome

The following parameters were evaluated:

1. Perforator Localization Precision: Horizontal error (*d′*) between planned and actual perforator location. (Excellent: <1 cm; Moderate: 1-2 cm; Poor: >2 cm).
2. Anatomical Consistency: Agreement between 3D planning and surgical findings for perforator type, source artery, and ALTP pedicle route.
3. Pedicle Diameter Discrepancy: Relative difference (r) between planned and measured pedicle diameter. (Excellent: r < 10%; Moderate: 10-20%; Poor: >20%).
4. Flap-Wound Fit: Quality of wound coverage. (Excellent: perfect fit; Moderate: minor size discrepancy; Poor: major discrepancy requiring significant adjustment).
5. Flap Survival and Quality: Assessed using established criteria,**^[^**^14^**^]^** which defined outcomes as Excellent, Good, Poor, or Very Poor.
6. Functional Outcomes: Hand function was evaluated with the Michigan Hand Outcomes Questionnaire.**^[^**^15^**^]^** Foot function was assessed with the AOFAS Ankle Hindfoot Scale.**^[^**^16^**^]^** For both instruments, scores were categorized as follows: Excellent: 100–80 points, Good: 79–60 points, Poor: 59–40 points, or Very Poor: below 39 points.
7. Complications: All adverse events were documented.
8. Workflow Efficiency: Pre-operative 3D labor time, skin-to-skin operating time, perforator search time, and flap ischemia time were compared to those of a historical control group. The control group consisted of 13 ALTP flaps planned with conventional Doppler and paper templates in 2019.

### Statistical analysis

All statistical analyses were performed using SAS software, version 9.4 (SAS Institute Inc.). Categorical variables are summarized as counts and percentages. The normality of continuous variables was assessed using the Shapiro-Wilk test. Normally distributed data were presented as mean ± standard deviation (x̄ ± s), while skewed data were presented as median with first and third quartiles [Median (Q1, Q3)].

## Results

The enhanced 3D visualization technique was applied to a cohort of 12 patients undergoing ALTP flap reconstruction. The median patient age was 45 years and the median wound area was 112 cm². 10 of 12 (83%) defects were located on the hand and 9 of 12 (75%) resulted from machinery crush injuries. Key outcomes are summarized below and detailed in Table 4.

**1. Perforator Localization Accuracy and Anatomical Consistency**

**Table 4:** Results.

Variables	n (%)
Perforator Localization Accuracy (n = 16), n (%)	
Excellent	13(81.3)
Moderate	3(18.8)
Poor	0
Perforator Type (n = 16), n (%)	
Intermuscular	6(37.5)
Musculocutaneous	10(62.5)
Perforator Type Consistency (n = 16), n (%)	
Consistent	16(100)
Inconsistent	0(0)
Perforator Source Artery (n = 16), n (%)	
Descending branch of LCFA	12(75.0)
Oblique branch of LCFA	4(25.0)
Perforator Source Artery Consistency (n = 16), n (%)	
Consistent	16(100)
Inconsistent	0(0)
ALTP Flap Pedicle Pathway (n = 16), n (%)	
descending branch of LCFA - vastus lateralis muscle	7(43.8)
descending branch of LCFA - vastus lateralis muscle interval	5(31.3)
oblique branch of LCFA - vastus lateralis muscle	3(18.8)
oblique branch of LCFA - vastus lateralis muscle interval	1(6.3)
ALTP Flap Pedicle Pathway Consistency (n = 16), n (%)	
Consistent	16(100)
Inconsistent	0(0)
Mean number of ALTP Flap Pedicle diameter, mm (n = 13)	
Preoperative (SD)	3.1(0.9)
Intraoperative (SD)	3.0(0.6)
Relative Diameter Difference Rate of ALTP Flap Pedicle (n = 13), n (%)	
Excellent	3(23.1)
Moderate	8(61.5)
Poor	2(15.4)
Flap and Wound Matching (n = 13), n (%)	
Excellent	11(84.6)
Moderate	2(15.4)
Poor	0(0)
Flap Survival (n = 13), n (%)	
complete survival	13(100)
partial survival	0(0)
Flap Quality (n = 13), n (%)	
Excellent	12(92.3)
Good	1(7.7)
Poor	0(0)
Very Poor	0(0)
follow-up time, months [IQR] (n = 12)	7.0(6.3,10.0)
Hand Function (n = 10), n (%)	
Excellent	2(20.0)
Good	4(40.0)
Poor	3(30.0)
Very Poor	1(10.0)
Foot Function (n = 2)	
Excellent	0
Good	2
Poor	0
Very Poor	0
Complications (n = 12), n (%)	
Local infection	1(6.7)
Delayed fracture healing	1(6.7)

Abbreviations: LCFA, lateral circumflex femoral artery; ALTP, anterolateral thigh perforator; IQR, interquartile range.

In total, 16 perforators were evaluated across the 13 ALTP flaps.

(1) Perforator Localization Accuracy
  Preoperative localization accuracy was excellent (*d′* < 1 cm) in 13 of 16 perforators (81.3%) and moderate (1 cm ≤ *d′* ≤ 2 cm) in 3(18.8%).
(2) Anatomical Consistency
  100% concordance (n = 16/16) was observed between the preoperative 3D planning and intraoperative findings for perforator type (musculocutaneous: 62.5%, n = 10/16; intermuscular: 37.5%, n = 6/16) and source artery (descending branch of LCFA: 75.0%, n = 12/16; oblique branch of LCFA: 25.0%, n = 4/16). Besides, all 13 ALTP flap pedicle pathways precisely matched the preoperative reconstructions (100% consistency).
(3) Pedicle Diameter Discrepancy
  The mean discrepancy between planned and actual pedicle diameter was minimal. The relative discrepancy rate was graded as excellent (23.1%, n = 3/13), moderate (61.5%, n = 8/13), and poor (15.4%, n = 2/13).

**2. Flap-Wound Fit and Surgical Efficiency**

(1) Flap-Wound Fit:
  The majority of flaps (84.6%, n = 11/13) achieved an excellent fit (complete coverage without tension), while the remainder (15.4%, n = 2/13) showed a moderate fit (minor size discrepancy not requiring trimming). No flaps exhibited a poor fit.
(2) Flap Survival and Quality
  All 13 flaps survived completely. Flap quality was rated excellent (92.3%, n = 12/13) and good (7.7%, n = 1/13).
(3) Functional Outcomes
  Both patient’s assessed foot functions (AOFAS) were graded as good. In contrast, hand function (MHQ, n = 10) outcomes were classified as excellent (20%, n = 2/10), good (40%,n = 4/10), poor (30%, n = 3/10), and very poor (10%, n = 1/10).
(4) Complications
  Complications occurred in two patients (16.7%, n = 2/12), including local infection (6.7%, n = 1/12) and delayed fracture healing (6.7%, n = 1/12).
(5) Workflow Efficiency
  Although 3D workflow required an additional pre-operative labor (all completed the day before surgery, causing no theatre delay), this significantly shortened key intra-operative times, including perforator search time, skin-to-skin time, and flap ischemia time (Table 5).

**Table 5:** Efficiency comparison (mean ± SD).

Metric	3D cohort (12 patients,13 flaps)	Control cohort (13 patients,13 flaps)	P value
Pre-operative 3D labour (min) [IQR]	233(187,262)	—	—
Perforator search time (min)	15 ± 4	28 ± 7	< 0.001
Skin-to-skin time (min)	178 ± 37	202 ± 42	0.03
Flap ischaemia time (min)	67 ± 11	81 ± 14	0.01

Abbreviations: IQR, interquartile range.
(6) Follow-up
  The median follow-up duration was 7.5 months (IQR: 6.3-11.0), which facilitated the assessment of outcome stability, with no late complications observed.

## Discussion

1. Evolution of Perforator Flap Localization Techniques

Given the significant anatomical variation in perforator vessel distribution,**^[^**^17,^^18^**^]^** perforator flap surgery carries inherent risks such as suboptimal or missing perforators and intraoperative perforator damage. Localization methods, such as ultrasound,**^[^**^19^**^]^** photoacoustic tomography,**^[^**^20^**^]^** CTA**,^[^**^21^**^]^** magnetic resonance angiography (MRA),**^[^**^22^**^]^** digital subtraction angiography (DSA),**^[^**^23^**^]^** infrared thermography,**^[^**^24^**^]^** and 3D visualization**^[^**^25^**^]^** have emerged over the past three decades to mitigate these risks effectively. 3D visualization technology enables the digital reconstruction of tissues into 3D models, aiding in preoperative planning, surgical simulation, and intraoperative navigation.**^[^**^26^**^]^**. This technology offers distinct benefits in perforator flap applications, facilitating three-dimensional visualization, reliable perforator localization, and objective, reproducible outcomes.

2. Challenges in Achieving Effective 3D Visualization Reconstruction

Due to resolution limitations, clinical CT scanners often fail to reliably depict vessels smaller than 0.5 mm in diameter.**^[^**^27^**^]^** Studies, such as those by Zhang et al.,**^[^**^28^**^]^** have indicated that advanced Force CT can consistently visualize and reconstruct microvessels as fine as 0.2-0.5 mm. However, this technology requires specialized equipment, hindering its broad clinical adoption. Moreover, the efficacy of these technologies is also highly dependent on optimized scanning protocols, with suboptimal parameters leading to inadequate image quality.**^[^**^29^**^]^**

A fundamental challenge in 3D visualization is the accurate isolation and reconstruction of minor vessels, which is critical for surgical planning. The conventional approach begins by establishing a CT value threshold for vessel extraction, capturing all encompassed tissues, followed by the application of region growth algorithms to extract additional contiguous tissues along the primary vascular axis.**^[^**^30^**^]^** This "direct extraction approach" often co-extracts "non-vascular" elements in contact with vessels, thereby diminishing the accuracy of the extraction process.

For optimal flap-wound congruence, surgeons must account for the flap's geometric configuration, tissue characteristics, vascular pedicle length, caliber, and other critical design elements. Existing digital design methodologies typically begin by measuring the wound's dimensions (length, width, depth, surface area) and then delineating the flap's harvesting area on the donor site accordingly.**^[^**^31^**^]^** This basic design approach often fails to incorporate details such as wound curvature, edge profile, and vascular pedicle conditions, potentially leading to designs that result in suboptimal wound coverage.**^[^**^11^**^]^**

The translation of 3D visualization into surgical navigation requires the accurate overlay of preoperative designs onto the intraoperative field. Perfecting this projection process and reducing inaccuracies is a significant area for research. While manual mapping is prone to substantial error, proposed solutions include printing the flap design on a flexible medium and aligning it with the donor site.**^[^**^32^**^]^** However, this method is susceptible to distortions from material and tissue deformations during fitting. Alternatively, mixed reality**^[^**^33,^^34^**^]^** technology can superimpose the flap design onto the donor site, presenting both on a head-mounted display for real-time interaction. However, mixed reality technology faces challenges, such as registration inaccuracies, latency in display, and potential for user discomfort.**^[^**^35^**^]^**

3. Technical Enhancements in Perforator Flap 3D Visualization Reconstruction

This study introduces systematic optimizations across several key aspects of perforator flap 3D visualization:

To obtain high-quality source data, a high-resolution CT scanner with rapid acquisition capability was employed, and scanning parameters were systematically optimized as detailed in the Imaging Protocol section and Table 2. In addition, to address the limitations of conventional direct extraction methods, an “Indirect Extraction Method” was developed, significantly improving the purity and accuracy of small vessel reconstruction, while meticulous manual tracing of perforator vessels was performed using Mimics’ “Thin Structure” tool, as described in the Methods section and Table 3. This technique enables precise 3D modeling of perforator courses through muscle (Level I) and superficial fascia (Level II), substantially enhancing anatomical accuracy according to Wei's classification.**^[^**^36^**^]^** Importantly, the ability to reliably identify perforators as small as 0.3 mm—while particularly relevant for supramicrosurgery—also confers significant benefits to all microsurgeons. It enhances surgical safety by minimizing intraoperative surprises and expands the range of viable perforators that can be confidently included in flap design, thereby improving surgical flexibility and outcomes.

Our refined "Digital Reverse Flap Design" technique incorporates wound mapping, surface reconstruction, flattening, and donor site projection while preserving comprehensive wound geometry (length, width, depth, curvature, and edge profile). This standardized approach improved reproducibility and minimized variability, resulting in a perfect tension-free fit in 11 out of 13 cases and a near-ideal fit in the remaining 2 cases, with no instances of size mismatch.

Using bony landmarks (anterior superior iliac spine and patellar edge) as reference points, we digitally quantified their spatial relationship to perforators and transposed these coordinates to the surgical field. Patient-specific 3D-printed guides were then fabricated to accurately locate perforators and delineate flap boundaries, minimizing manual measurement errors and tissue distortion, and providing a practical clinical solution pending widespread adoption of mixed reality technologies.

4. Study impact and clinical benefits

3D visualization for perforator flap design represents a convergence of computer science and microsurgery, offering precise localization, detailed planning, clear imaging, and reproducible results. Its increasing use has lowered complications and enhanced surgical success, emerging as a key area in flap surgery research.**^[^**^37^**^]^** Importantly, our study introduced enhancements in imaging, vessel extraction, perforator mapping, flap planning, and data transfer, aiming to boost 3D reconstruction efficacy and integrate it into clinical settings.

In contrast to the conventional ALT, where most tailoring is done freehand after vessel anastomosis, our workflow relocates more than 90% of the shaping process to the preoperative phase. By mirroring the 3D wound surface onto the donor site, verifying that the selected perforator(s) lie within the template, and translating the virtual design to the skin with a 3D-printed rigid guide, secondary edge adjustments were reduced from 46.2% (n = 6/13) in historical controls to 9.2 % (n = 1/13), flap ischemia time was shortened, and marginal perforators that would otherwise have been sacrificed during intra-operative “eye-ball” trimming were preserved. The observed reduction in flap ischemia time can be attributed to precise preoperative planning, which reduced intraoperative vessel exploration and matching. Improved flap-wound congruence minimized the need for flap trimming both before and after microvascular anastomosis. The surgical team’s growing proficiency may have also contributed to this improvement.

The clinical application of our refined 3D approach across 13 cases indicates that, with meticulous preoperative planning, it effectively manages perforator diversity, ensuring precise and successful surgeries.

**Figure 1:**
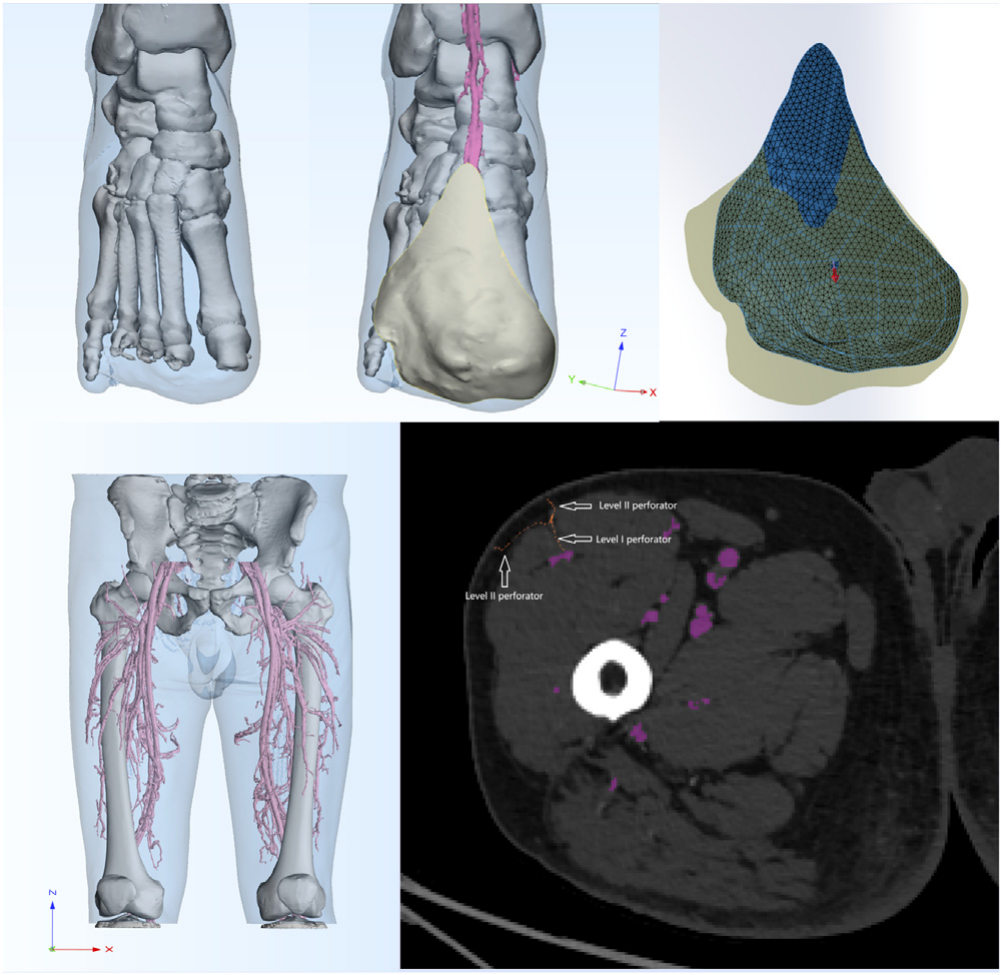
3D Model reconstruction and flap planning. *(First line, left)* donor site model reconstruction. *(First line, middle)* creation of the recipient site model from surface scanning of the wound. *(First line, right)* flattening of the recipient site model for flap template design. *(Second line, left)* Donor site model extraction and reconstruction *(Second line, right)* schematic of perforator anatomy. Arrows illustrate the course of a Level I perforator and its subsequent bifurcation into two Level II branches.

**Figure 2:**
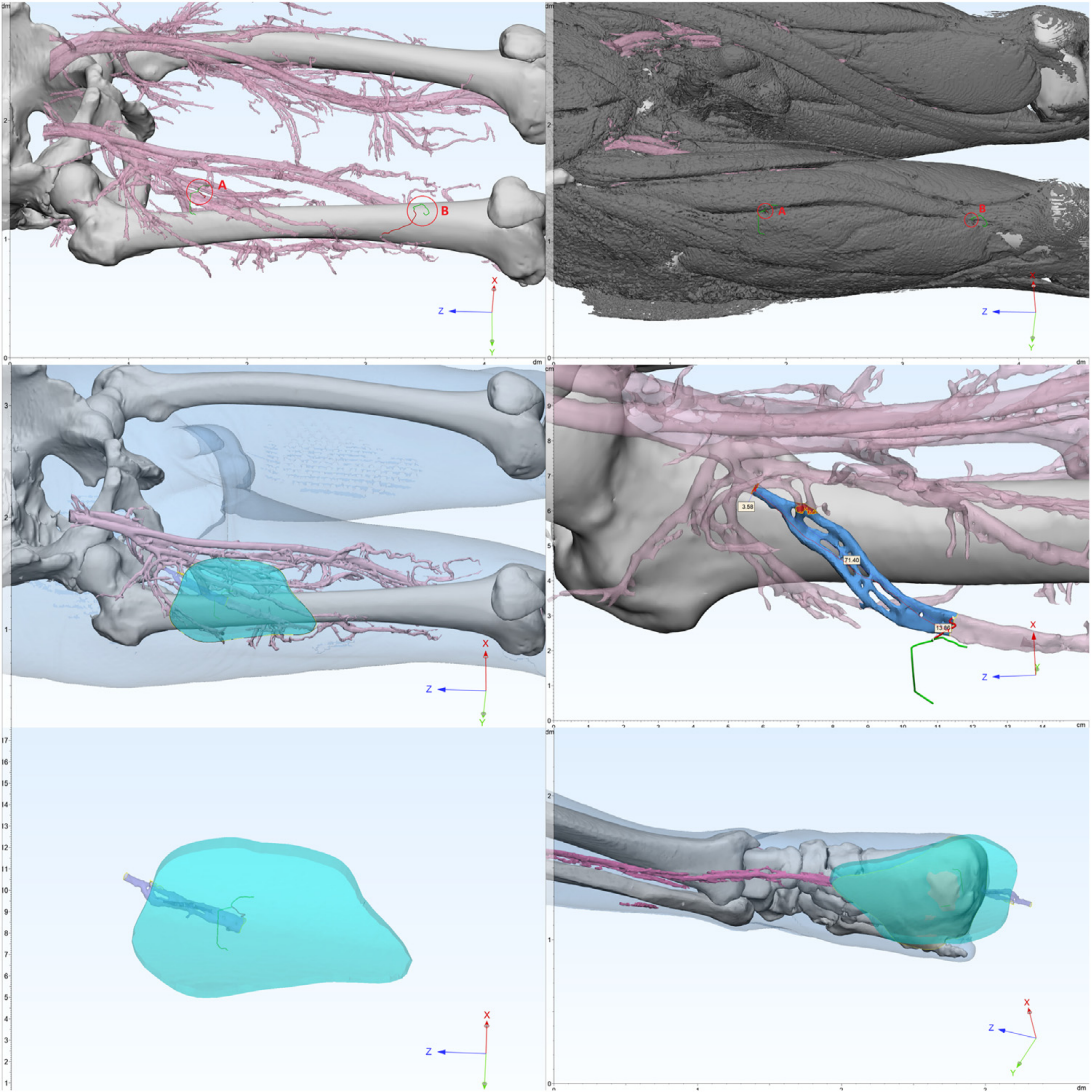
*(First line, left)* Preoperative 3D modeling in 3-matic software. The imported vascular supply area model highlights two dominant perforators: Branch A (red, Grade I) and Branch B (green, Grade II), both identified for surgical guidance. *(First line, right)* Perforator A surfaces after traversing the vastus lateralis, classifying it as a musculocutaneous type; Perforator B emerges from the intermuscular space between the rectus femoris and vastus lateralis, indicating an intermuscular type. *(Second line, left) Simulated skin flap harvest area.* *(Second line, right)* The internal diameter of the vascular pedicle is 3.58 mm; the length of the vascular pedicle is 71.40 mm + 13.86 mm = 85.25 mm. *(Third line, left)* Simulated Flap Harvest Model. *(Third line, right)* Preview of repair: The flap fully covers the wound.

**Figure 3:**
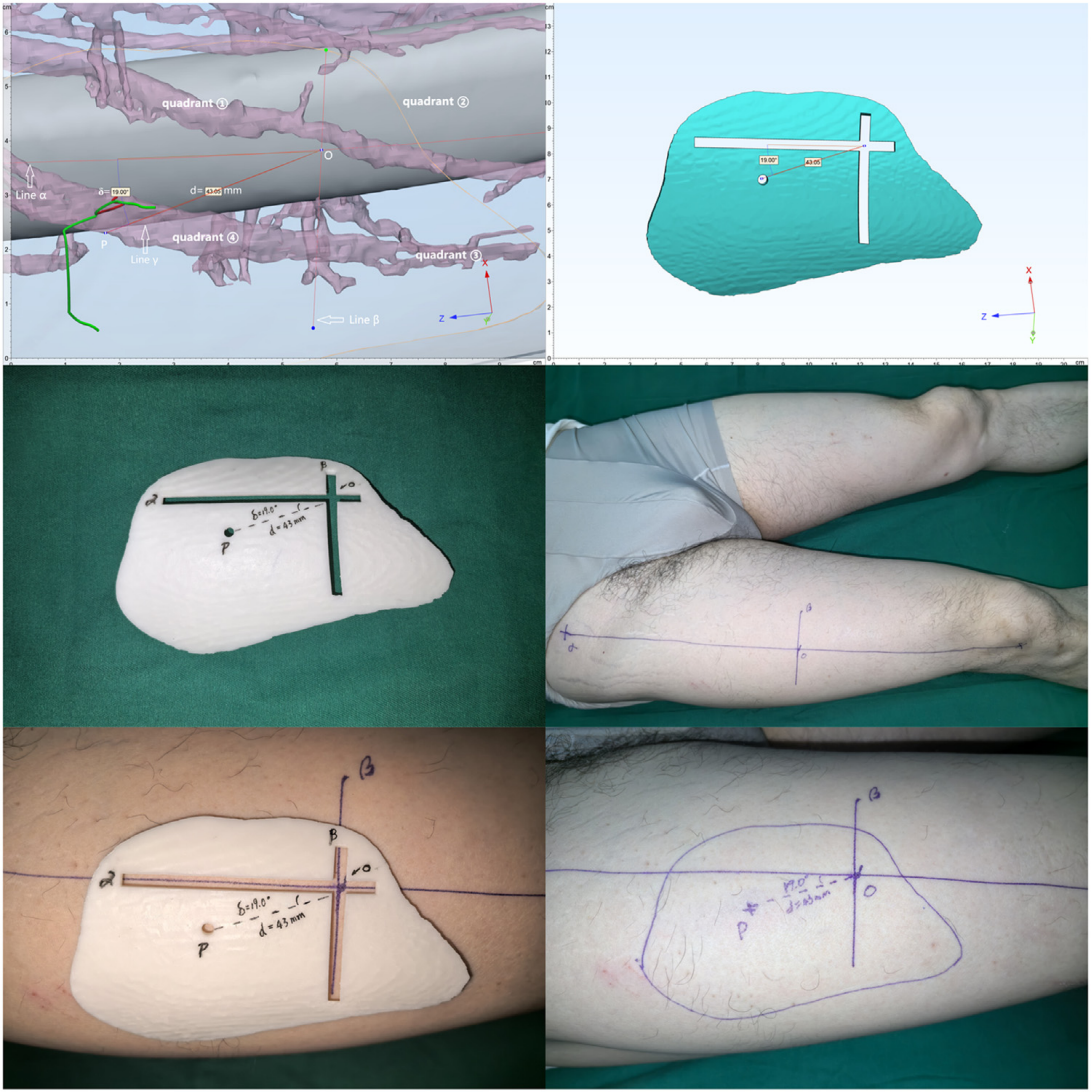
*(First line, left)* Location of Perforator A: Plotted in the 4th quadrant at a distance of 43.05 mm and an angle of 19.0° from reference point P. *(First line, right) 3D-printed guide design with landmark P, and hollowed channels for alignment lines α and β.* *(Second line, left) 3D-printed guide fabrication.* *(Second line, right)* Alignment lines α and β are drawn on the donor site and intersect to define the coordinate origin (Point O). *(Third line, left)* The guide is secured to the donor site with lines α and β engaged in their respective slots. The circular window accurately transposes the preoperatively located perforator point onto the skin. *(Third line, right)* Flap area delineated by the guide edge.

**Figure 4:**
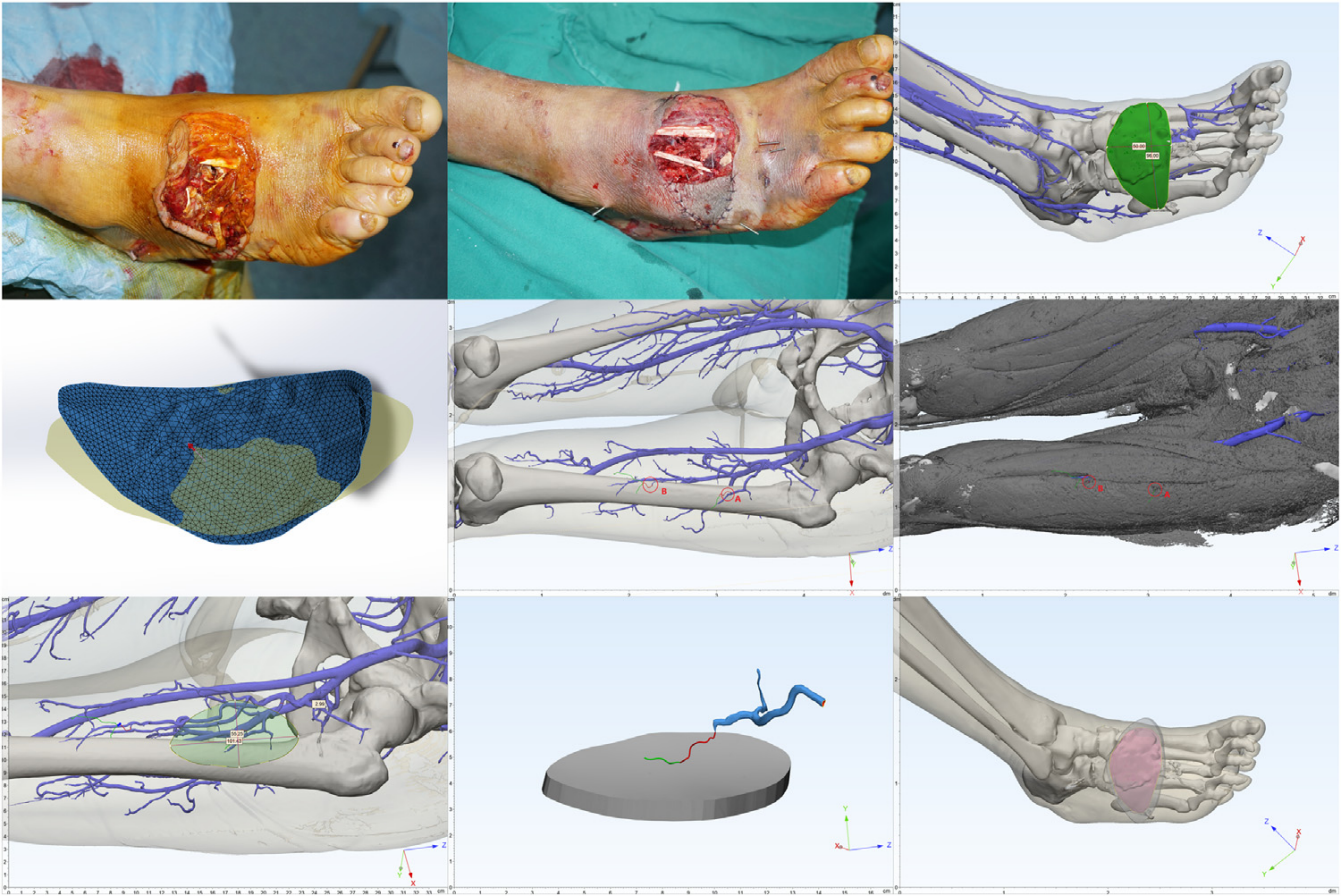
Case : A 57-year-old man who sustained a soft tissue defect on the dorsum of the right foot following a traffic accident. The defect was reconstructed with an ALTP flap. *(First line, left)* Wound appearance post-injury. *(First line, middle)* Post-debridement: 9.6 cm × 5.0 cm wound on the foot dorsum, with tendons and bones exposed. *(First line, right)* CT reconstruction of the foot, marking the right foot wound. *(Second line, left)* Flattening of the wound model. *(Second line, middle)* CTA and 3D reconstruction of the donor site, showing the location of perforators A and B. *(Second line, right)* Perforator A: musculocutaneous; Perforator B: intermuscular. *(Third line, left)* Design of the ALTP flap, harvest area 10.1 cm × 5.5 cm. *(Third line, middle)* Surgical marking outlining the planned incision for the ALTP flap harvest, based on the preoperatively identified perforators. *(Third line, right)* Intraoperative view showing the ALTP flap partially inset into the dorsal foot defect.

**Figure 5:**
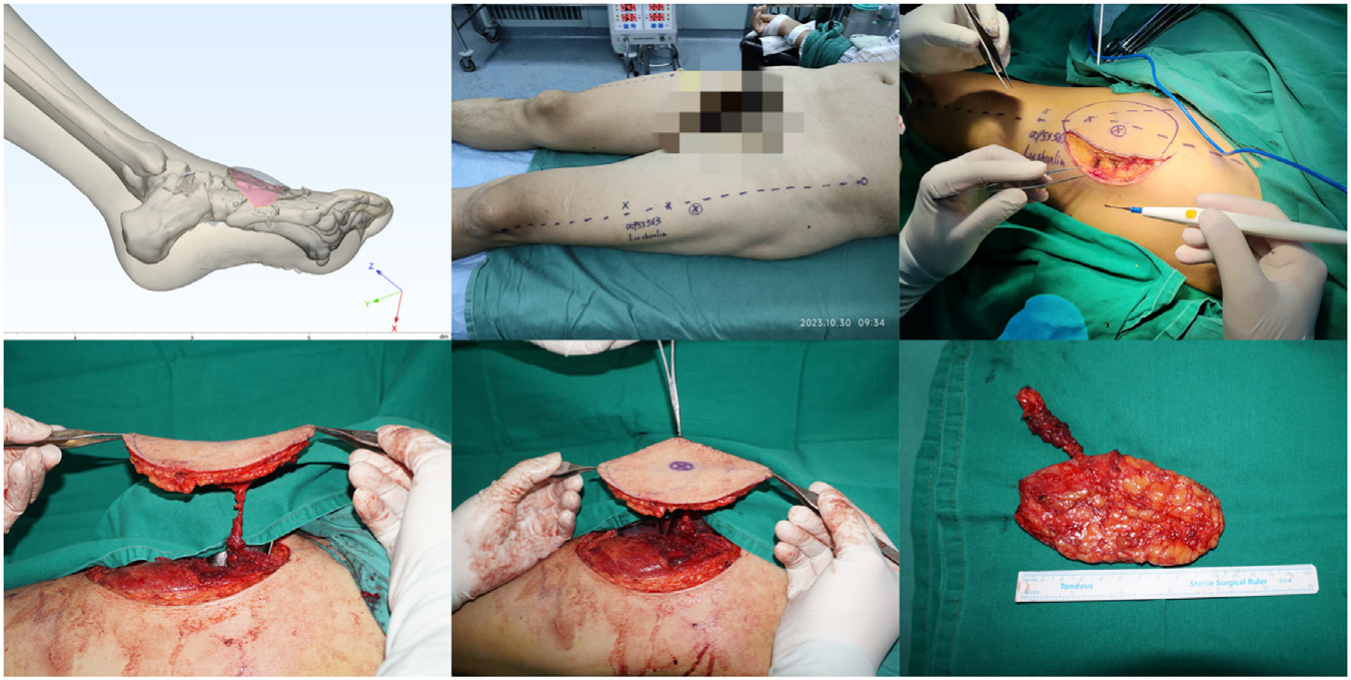
*(First line, left)* Intraoperative view showing the raised ALTP flap prior to transfer and inset onto the recipient site. *(First line, middle)* Intraoperative photograph showing the marking of surface anatomy and perforator points. *(First line, right)* Incising one side of the flap. *(Second line, left and middle)* Exploring target perfs, confirming accurate localization and type assessment. *(Second line, right)* The flap is detached.

**Figure 6:**
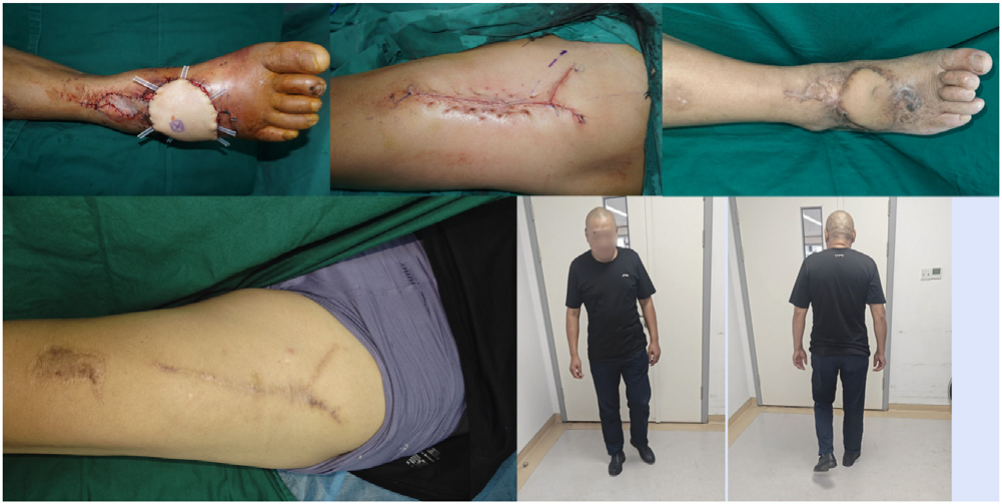
*(First line, left)* The flap fully covers the wound with good blood supply. *(First line, middle)* Layered, tension-minimizing sutures in the donor site. *(First line, right)* Follow-up at 10 months showing a fully viable, well-integrated flap with a good functional and aesthetic result. *(Second line, left)* Linear scar in the donor area *(Second line, middle and right)* The patient resumed weight-bearing walking with good foot function.

This study is subject to several important limitations. First, the clinical cohort of 12 patients is relatively modest, which may limit the generalizability of the findings. Second, the retrospective nature of the analysis inherently constrains the strength of the evidence. Furthermore, the technology itself requires a substantial learning curve, currently rendering it unsuitable for time-sensitive emergency procedures. Finally, several procedural steps, such as the translation of curved surfaces into two-dimensional planes, perforator mapping, and surface marking, involve manual operations that introduce a potential for subjective error (Figure 1, Figure 2, Figure 3, Figure 4, Figure 5, Figure 6).

## Conclusion

The enhanced 3D visualization technique significantly improves perforator localization accuracy (81.3% with <1 cm error) and anatomical consistency (100% match for type/source artery), ensuring high flap survival (100%), optimal wound fit (84.6% excellent), and refined surgical precision. Despite these promising results, the study's small sample size, retrospective design, technical learning curve, subjective errors in manual operations, and short follow-up duration may limit the generalizability and reliability of the findings. Future research should include multicenter prospective studies, long-term follow-up, and technological optimization to validate these findings and investigate broader surgical applications.

## Ethical approval statement

Approval was obtained from the Ethics Committees of Wuxi Ninth People’s Hospital Affiliated to Soochow University and Affiliated Hospital of Jiangsu University.

## Conflicts of interests

All the authors declare no conflicts of interest.

## Appendix Supplementary materials

Image, application 1

Image, application 2

Image, application 3

Image, application 4

Image, application 5

Image, application 6

## References

[1] Koshima I., Soeda S. (1989). Inferior epigastric artery skin flaps without rectus abdominis muscle. Br J Plast Surg.

[2] Koshima I., Soeda S. (1991). Free posterior tibial perforator-based flaps. Ann Plast Surg.

[3] Hallock G.G. (2022). Perforator and perforator flap spinoffs: a historical journey. Plast Reconstr Surg.

[4] Kwon J.G., Brown E., Suh H.P., Pak C.J., Hong J.P. (2023). Planes for perforator/skin flap elevation-definition, classification, and techniques. J Reconstr Microsurg.

[5] Han T., Khavanin N., Zhu S. (2022). A comparison of handheld doppler and indocyanine green angiography for perforator localization. Ann Plast Surg.

[6] Wang Y., Jiang Y., Lu G. (2023). Improving visualization of free fibula flap perforators and reducing radiation dose in dual-energy CT angiography. Quant Imaging Med Surg.

[7] Visconti G., Bianchi A., Di Leone A., Franceschini G., Masetti R., Salgarello M. (2024). The ultrasound evolution of lateral thoracic perforator flaps design and harvest for partial and total breast reconstruction. Aesthet Plast Surg.

[8] Jiang Y., Ding M., Zhou Z. (2021). A 3D visualization layered anatomy for acromial arterial rete and flap design. Surg Radiol Anat.

[9] Rzeplinski R., Slugocki M., Kwiatkowska M. (2023). Standard clinical computed tomography fails to precisely visualise presence, course and branching points of deep cerebral perforators. Folia Morphol (Warsz).

[10] Zhang Y.H., Cui W.J., Song K.X. (2023). A prospective study of the perforator evaluation and eccentric design of anterolateral thigh flap based on superficial fascial perforators assisted by modified computed tomography angiography]. Zhonghua Shao Shang Yu Chuang Mian Xiu Fu Za Zhi.

[11] Rogers L.C., Bevilacqua N.J., Armstrong D.G., Andros G. (2010). Digital planimetry results in more accurate wound measurements: a comparison to standard ruler measurements. J Diabetes Sci Technol.

[12] Winkler D., Kropla F., Busse M. (2024). Mixed reality for spine surgery: a step into the future with a human cadaveric accuracy study. Neurosurg Focus.

[13] Wan B., Fu H.Y., Yin J.T., Ren G.Q. (2015). Low-molecular-weight heparin and intermittent pneumatic compression for thromboprophylaxis in critical patients. Exp Ther Med.

[14] Gy P.D., De S., Kuishui S. (2000). Trial standards for the functional assessment of the upper limb by the hand surgery society of the chinese medical association. Chin J Hand Surg.

[15] Kroon F.P.B., Boersma A., Boonen A. (2018). Performance of the Michigan hand outcomes questionnaire in hand osteoarthritis. Osteoarthr Cartil.

[16] Kitaoka H.B., Alexander I.J., Adelaar R.S., Nunley J.A., Myerson M.S., Sanders M. (1994). Clinical rating systems for the ankle-hindfoot, midfoot, hallux, and lesser toes. Foot Ankle Int.

[17] Shimbo K., Okuhara Y., Yokota K. (2021). Perforator switching flap: A breakthrough option for anatomical variation and perforator injury of anterolateral thigh flap. J Plast Reconstr Aesthet Surg.

[18] Lee Y.C. (2024). A simplified classification and economical application of anterolateral thigh flap. J Plast Reconstr Aesthet Surg.

[19] Perez-Iglesias C.T., Laikhter E., Kang C.O. (2022). Current applications of ultrasound imaging in the preoperative planning of DIEP Flaps. J Reconstr Microsurg.

[20] Tsuge I., Saito S., Munisso M.C. (2024). Noninvasive visualization of the midline-crossing arterial variation in the deep inferior epigastric artery perforator flap using photoacoustic tomography for application in patients with abdominal scars. J Plast Reconstr Aesthet Surg.

[21] Yu X.X., Yang S.F., Ji C.S., Qiu S.Q., Qi Y.D., Wang X.M. (2022). A novel computed tomography angiography technique: guided preoperative localization and design of anterolateral thigh perforator flap. Insights Imaging.

[22] Thimmappa N.D. (2024). MRA for preoperative planning and postoperative management of perforator flap surgeries: a review. J Magn Reson Imaging.

[23] Yang L., Cheng J., Liu Z. (2023). Morphological study of branches of lateral femoral circumflex artery based on digital subtraction angiography. J Plast Reconstr Aesthet Surg.

[24] Berner J.E., Pereira N., Troisi L., Will P., Nanchahal J., Jain A. (2021). Accuracy of infrared thermography for perforator mapping: a systematic review and meta-analysis of diagnostic studies. J Plast Reconstr Aesthet Surg.

[25] Hauck T., Arkudas A., Horch R.E. (2022). The third dimension in perforator mapping-comparison of cinematic rendering and maximum intensity projection in abdominal-based autologous breast reconstruction. J Plast Reconstr Aesthet Surg.

[26] Faderani R., Singh P., Krumhuber E., Mosahebi A., Ponniah A. (2022). 3D photography and computer modelling in nasal reconstruction. J Oral Biol Craniofac Res.

[27] Liu S.C., Chiu W.K., Chen S.Y., Lee T.P., Wang H.W., Chen S.G. (2011). Comparison of surgical result of anterolateral thigh flap in reconstruction of through-and-through cheek defect with/without CT angiography guidance. J Craniomaxillofac Surg.

[28] Zhang Di Q.Y., Shenqiang Q., Hongzhi T., Zengtao W. (2022). Force CT microvascular anatomical imaging technology and its clinical application. Chin J Microsurg.

[29] von Spiczak J., Mannil M., Peters B. (2018). Photon counting computed tomography with dedicated sharp convolution kernels: tapping the potential of a new technology for stent imaging. Invest Radiol.

[30] Cui H., Ding M., Mao Y. (2020). Three-dimensional visualization for extended deep inferior epigastric perforator flaps. Ann Plast Surg.

[31] Taotao H.. "Clinical application of preoperative precise design of perforator flaps assisted by mimics combined with CTA,"(in Chinese) Soochow University, 2019.

[32] Li L.. "The application of digital visualization technology based on MRI in superficial tumor excision and perforator flap operations in plastic surgery,"(in Chinese) Chinese PLA General Hospital, 2019.

[33] Jiang T., Yu D., Wang Y., Zan T., Wang S., Li Q. (2020). HoloLens-based vascular localization system: precision evaluation study with a three-dimensional printed model. J Med Internet Res.

[34] Nuri T., Mitsuno D., Iwanaga H., Otsuki Y., Ueda K. (2022). Application of augmented reality (AR) technology to locate the cutaneous perforator of anterolateral thigh perforator flap: a case report. Microsurgery.

[35] Qi Z., Jin H., Wang Q. (2024). The feasibility and accuracy of holographic navigation with laser crosshair simulator registration on a mixed-reality display. Sens (Basel).

[36] Zairong W. (2014). Analysis of vascular factors affecting the survival of skin flaps. Chin J Aesthet Plast Surg.

[37] Sampieri G., Tran J., Feng A.L., Agur A., Davies J. (2024). Characterization of the MSAP flap in head and neck surgical oncology: a 3D cadaveric study. Laryngoscope.

[38] Zhou K., Parker J.D. (2019). The role of vascular endothelium in nitroglycerin-mediated vasodilation. Br J Clin Pharmacol.

[39] Yang L., Yan Z., Lu G. (2023). Nitroglycerin improves the visibility of fibula-free flap perforators on computed tomography angiography in patients with oral or maxillofacial lesion. Eur J Radiol.

