# Supplementary material for: Enhanced three-dimensional visualization reconstruction for perforator flaps: A case series on clinical applications and outcomes
Source: JPRAS Open. 2026 May 14;50:344–59. doi: 10.1016/j.jpra.2026.04.015 (PMC13240778; doi:10.1016/j.jpra.2026.04.015)
Supplement: Supplementary file 1 [file mmc1.pdf]

·功能评定标准·

中华医学会手外科学会上肢部分功能评定试用标准

潘达德　顾玉东　侍德　寿奎水

1989 年,中华医学会骨科学会手外科学组,在广州召开手功能评定标准专题讨论会,会上制定一个手功能评定标准(手外科杂志,1990,6:57 - 72),从而我国有了第一个正式的手功能评定标准。但事后发现该标准有不够完善的地方,北京积水潭医院赵书强、王澍寰等(手外科杂志,1994,10:69 - 72)在 1994 年又加以改进。这个改进的标准已编成计算机软件,只要把手功能检查数据输入,便能得出手功能丧失的数值。这项工作对于科学研究、工伤残废评定、医疗保险及医学法律等有重要的参考价值。然而,对于进行断肢再植及断指再植的疗效及临床科研来讲,有必要制定一个更为适应复查的标准。2000 年 3 月 26 日、27 日,中华医学会手外科学会在无锡市又一次召开了全国上肢功能评定标准专题研讨会,以下是讨论会提出的具体方案,提供在全国范围内试行。

方案制定的前提是适应我国目前基层的情况,简单实用,容易掌握,不需要特殊检查仪器或设备;另一方面,又要求尽可能客观、科学、准确,与国际标准接轨。为了掌握方便,评定采用百分制,再评为优、良、差、劣。对于断肢再植,陈中伟于 1978 年曾提出一个断肢再植功能评定标准,国际手外科学会联合会于 1984 年推荐使用。我们在此基础上,提出了一个上肢断肢再植后功能评定标准,从关节活动度、肌力、感觉恢复、外形、后遗症状与工作能力等六方面加以评定,提供全国试用。

中华医学会手外科学会上肢断肢再植功能评定试用标准

一、关节活动度 TAM(30 分)

1. 肩关节(外展)	评分
90° ~ 60°	6 分
59° ~ 45°	5 分
44° ~ 30°	3 ~ 4 分
< 30°	0 ~ 2 分

2. 肘关节(伸屈)	评分
120° ~ 90°	7 ~ 8 分
89° ~ 60°	5 ~ 6 分
59° ~ 30°	3 ~ 4 分
< 30°	0 ~ 2 分

3. 腕关节(伸屈)	评分
60° ~ 90°	3.1 ~ 4 分
45° ~ 59°	2 ~ 3 分
30° ~ 44°	1.5 ~ 2 分
< 30°	0 ~ 1.5 分

4. 掌指关节(伸屈)	评分
90° ~ 70°	4.1 ~ 5.0 分
69° ~ 50°	3.1 ~ 4.0 分
49° ~ 30°	2.1 ~ 3.0 分
< 30°	0 ~ 2.0 分

5. 近指关节(伸屈)	评分
100° ~ 80°	3.1 ~ 4.0 分
79° ~ 60°	2.1 ~ 3.0 分
59° ~ 30°	1.5 ~ 2.0 分
< 30°	0 ~ 1.4 分

6. 远指关节(伸屈)	评分
45° ~ 30°	2.1 ~ 3 分
29° ~ 20°	1.1 ~ 2 分
19° ~ 15°	1 分
< 15°	0 分

二、肌力(20 分)	评分
M4 以上	17 ~ 20 分
M4	13 ~ 16 分
M3	8 ~ 12 分
M2 以下	0 ~ 7 分

三、感觉(20 分)	评分
S4	16 ~ 20 分
$S3^+$	12 ~ 15 分
S3	8 ~ 11 分
< S2	0 ~ 7 分

四、外形(10 分)	评分
正常或略显萎缩	8.1 ~ 10 分

中华医学会杂志社 Q2xvdWRWZX[illegible]zaW9uLQo?

轻度萎缩	6.1 ～ 8 分
中度萎缩	3 ～ 6 分
明显萎缩	0 ～ 2 分

五、遗留症状(10 分)　评分

无麻木、疼痛或其它不适	10 分
轻度麻/痛,轻度不适	7 分
不适或麻/痛	3 分
疼痛、过敏、成为累赘	0 分

六、工作情况(10 分)　评分

恢复原工作	10 分
从事轻工作	7 分
能满足日常生活需要	3 分
无实用功能	0 分

评定方法：

优(总分)	100 ～ 80 分
良(总分)	79 ～ 60 分
差(总分)	59 ～ 40 分
劣(总分)	39 分以下

说明：

1. 肌力与感觉均采用英国医学研究会 1954 年颁布的标准评定。
2. 现在应用的关节测量器可以有 2 % ～ 3 %的误差,因此,临界角度上计分有交叉,实际计算时,可就高分计算。

目前,文献上有关断指再植术后功能评定的标准有米满(整形外科(日)1977,28 :183),Jones (J Hand Surg,1982,7 :183),Nakamura Tamai (J Hand Surg,1983,8 (part 2) :780),中国人民解放军显微外科专业组(1988)张家界会议和朱盛修等(中华显微外科杂志,1989,12 :116－117)制订的五种标准。其中,Nakamura Tamai 标准是国际手外科学会联合会所推荐使用的。五种标准分别从运动、感觉、遗留症状、外形、综合活动能力、病人满意程度和从事工作情况等项目进行计分,然后,评为优、良、差、劣等四个等级。有些单位用上述几组标准对几组病例进行实际随访评定(中华显微外科杂志,1989,12 :148-151,J Hand Surg (Am),1998,23 :635-646),总体是适用的,能反映出功能情况 ;然而,有的项目内容是重复的,有的项目在我国现在条件下很不适用,影响准确性。例如,病人满意程度,很受病人的意愿、情绪、周围环境的影响 ;同样情况下,有人感到很满意,有人则表示很不满意。对于工作状况也一样,在现代社会,工作流动性大,信息科技的发展,很多人由体力劳动向脑力劳动转化。工农业生产方式也不同,原来是学生,现在长大成人,有了职业 ;因此,所担任的工作不一定代表手功能的好坏。为此,与会代表建议综合各家之长,制定一个简便实用,适合国情,从运动——关节活动功能、完成日常生活活动、感觉恢复程度、再植手指外观、血液循环状态、从事工作能力等六个方面进行综合评定的标准,采取的方法尽量与国际接轨。

中华医学会手外科学会
断指再植功能评定试用标准

一、运动功能

用 TAM 系统评定标准(20 分)

1. 拇指 :A 拇指对指(10 分)

可以	10 分
困难	5 分
不能	0 分

B 拇指关节自主活动度(10 分)

掌指关节 ROM + 指间关节 ROM = 总 ROM

总 ROM	评分
> 90°	10 分
< 90°	5 分
强直	0 分

2. 手指 :关节自主活动度(20 分)

掌指关节 + 近位指间关节 + 远位指间关节总屈曲度 – 总欠伸度 = 总 TAM

总 TAM	评分
260° ～ 200°	20 ～ 16 分
190° ～ 130°	15 ～ 11 分
130° ～ 100°	10 ～ 6 分
< 100°	5 ～ 0 分

二、日常生活活动 :ADL(20 分)

1. 捡针(指甲捏)
2. 捡分币(指腹捏)
3. 写字(三指捏)
4. 提(提箱柄,壶柄等重物)
5. 拿大茶缸(握)
6. 锤钉子(强力握持)
7. 上螺丝(中央握持)
8. 结鞋带(综合细动作)
9. 扣钮扣(综合细动作)
10. 开广口瓶(综合强力握持和精细握持)

每项评分 :完成良好	2 分
可以完成,动作不太好	1 分

不能完成　0 分

三、感觉恢复(20 分)

按照英国医学研究会评定标准(1954)

分级		评分
S4	感觉恢复正常,两点分辨觉 < 6 mm	20 分
S3⁺	除 S3 外,尚有部分两点分辨觉存在	16 分
S3	浅痛觉与触觉完全恢复,没有过敏	12 分
S2	浅感觉与触觉有少许恢复	8 分
S1	皮肤深痛觉恢复	4 分
S0	神经管辖区无任何感觉	0 分

四、血液循环状态(10 分)

分级		评分
优	皮肤色泽、温度正常,不需特殊保护	10 分
良	色泽稍差,温度略低,怕冷	8 分
差	肤色苍白或发绀,温度明显发凉特别怕冷	4 分
劣	肤色灰暗或发绀,冷天不敢外露	2 分

五、外观(20 分)

分级		评分
优	再植指没有旋转、非功能成角畸形外形丰满,短缩 < 1 cm 无明显功能影响	20 分
良	再植指轻度旋转、非功能成角畸形轻度萎缩,短缩 < 1.5 cm 无明显功能影响	16 分
差	旋转、成角畸形影响功能有萎缩,短缩不超过 2 cm	8 分
劣	畸形明显,短缩超过 2 cm 严重影响功能及外观	4 分

六、恢复工作情况(10 分)

分级		评分
优	恢复原工作	10 分
良	参加轻工作	7 分
差	不能工作,但能自理生活	3 分
劣	不能工作,生活也不能自理	0 分

根据以上六项评分,等级分值:

优　100 ~ 80 分　良　79 ~ 60 分

差　59 ~ 40 分　劣　< 40 分

说明:

1. 多指离断时,对于关节活动各指各个关节独立检查,然后相加,除以指数,取其平均值。

2. TAM = total active motion 总主动活动度。

3. ADL = activities of daily living 日常生活活动。

附件(一)

陈中伟(1984 年)提出的断肢再植评定标准。

陈中伟(1984 年)断肢再植评定标准

分级	关节活动度	肌力	感觉	工作能力
优	原有的 60%	4 ~ 5 级	> S3	原工作
良	原有的 40%	3 ~ 4 级	S3	轻工作
可	原有的 30%	2 ~ 3 级	S2	生活自理
差	< 原有的 30%	0 ~ 1 级	S0 ~ 1	无实用功能

附件(二)

顾玉东在会上提出的断指再植功能标准,与陈中伟提出的断肢再植功能评定标准相对应的简易标准。

顾玉东(2000 年)断指再植评定标准

评分	运动	感觉	外观	工作能力
4	TAM 正常	> S3	正常	原工作
3	> 健侧的 75%	S3	较满意	轻工作
2	> 健侧的 50%	S2	尚可	有部分功能
1	< 健侧的 50%	S0 ~ 1	不满意	无功能

综合评价:优　13 ~ 16 分　良　9 ~ 12 分

可　5 ~ 8 分　差　4 分以下

青岛解放军第四〇一医院
潘达德、程国良起草

中华医学会手外科学会 拇、手指再造功能评定试用标准

一、功能活动度(6 分)

1. 再造拇或手指对捏功能

能相互触及或相距 < 1 cm	3 分
相距 1 ~ 2 cm	2 分
相距 ≤ 3 cm	1 分
相距 > 3 cm	0 分

2. 再造拇对掌功能(3 分)

拇对掌距掌 ≥ 5 cm 活动到 ≤ 2 cm	3 分
拇对掌距掌 ≥ 5 cm 活动到 ≤ 3 cm	2 分
拇对掌距掌 ≥ 5 cm 活动到 ≤ 4 cm	1 分
拇对掌距掌 > 5 cm 无活动	0 分

3. 2 ~ 5 再造手指屈曲功能(3 分)

屈曲指端距掌纹 ≤ 3 cm	3 分
屈曲指端距掌纹 < 4 cm	2 分
屈曲指端距掌纹 < 5 cm	1 分
屈曲指端距掌纹 > 5 cm 或不能屈指	0 分

* 再造拇或手指的对捏功能为必测项目,再造拇对掌功能、再造指屈曲功能为参考项目,评分只取其中一项高分计算。运动功能总分以 6 分计算。

二、再造指力量(3 分)

中华医学会杂志社 QZwdWRWZXJzaW9uLQo?

检测捏力或握力，取其中一项高分计算：

再造手为非优势手

大于健手的 60 %	3 分
大于健手的 40 %	2 分
大于健手的 20 %	1 分
小于健手的 20 %	0 分

再造指为优势手，占健手的百分比相应增加 10%。

三、感觉测定(指腹，3 分)

≥S3，两点分辨觉 5 ~ 7 cm	3 分
S3	2 分
S2	1 分
S1	0 分

四、手使用情况(3 分)

1. 工作能力(3 分)

恢复原工作或生活自理	3 分
轻工作，生活自理	2 分
部分生活自理	1 分
大部分生活不能自理或无功能	0 分

2. 综合功能检测(3 分)

用 6 项运动检测，每项得 0.5 分。

① 捡分币或针　② 写字或捻线

③ 系带子或钮扣　④ 握用锤子或切菜刀具

⑤ 拧螺丝或瓶盖　⑥ 持碗或杯子

手的使用情况，从工作能力和综合功能二项检测中取高分的一项记录评定。

综合评价 以上一至四评分相加：

优　13 ~ 15 分　良　9 ~ 12 分

可　5 ~ 8 分　差　4 分及 4 分以下

无锡市手外科医院

寿奎水、芮永军起草

中华医学会手外科学会 上肢周围神经功能评定试用标准

一、上肢五大神经功能评定试用标准

(一)腋神经修复后功能评定试用标准

(1)评分标准

腋神经功能评分试用标准

分数	肩外展	肌力
4	> 90°	≥M4
3	60 ~ 90°	≥M3
2	30 ~ 60°	≥M2
1	< 30°	< M2

(2)综合评价

分级：优　7 ~ 8 分　良　5 ~ 6 分

可　3 ~ 4 分　差　2 分以下

(二)肌皮神经修复后功能评定试用标准

(1)评分标准

肌皮神经功能评定试用标准

分数	肘关节屈曲	肌力
4	> 90°	≥M4
3	60 ~ 90°	≥M3
2	30 ~ 60°	≥M2
1	< 30°	< M2

(2)综合评价

分级：优　7 ~ 8 分　良　5 ~ 6 分

可　3 ~ 4 分　差　2 分以下

(三)桡神经修复后功能评定试用标准

(1)评分标准

桡神经功能评定试用标准

评分	伸腕	肌力	伸拇	伸指
4	> 45°	> M3	TAM 优	TAM 优
3	≥30°	M3	TAM 良	TAM 良
2	< 30°	M2	TAM 可	TAM 可
1	不能	M0 ~ 1	TAM 差	TAM 差

注：伸指功能取 4 指 TAM 的平均值

(2)综合评价

分级：优　13 ~ 16 分　良　9 ~ 12 分

可　5 ~ 8 分　差　4 分以下

(四)正中神经修复后功能评定试用标准

(1)评分标准

正中神经功能评定试用标准

分数	屈腕肌力	屈指	拇对掌	感觉
4	> M4	TAM 优	正常	S4
3	M3	TAM 良	能对环指	S3
2	M2	TAM 可	能对示中指	S2
1	M0 ~ 1	TAM 差	不能	S0 ~ 1

注：屈指功能取示、中指 TAM 的平均值

(2)综合评价

分级：优　13 ~ 16 分　良　9 ~ 12 分

可　5 ~ 8 分　差　4 分以下

(五)尺神经修复后功能评定试用标准

(1)评分标准

尺神经功能评定试用标准

分数	外形	屈指	感觉
4	无爪形畸形	TAM 优	S4
3	轻度爪形畸形(不伴肌萎缩)	TAM 良	S3
2	中度爪形畸形(伴肌萎缩)	TAM 可	S2
1	重度爪形畸形(肌萎缩明显)	TAM 差	S0 ~ 1

注：屈指功能取环、小指 TAM 的平均值

(2)综合评价

QZxvdWRWZXJzaW9uLQo?

中华手外科杂志 2000 年 9 月第 16 卷第 3 期 Chin J Hand Surg, September 2000, Vol 16, No.3

分级：优 10 ~ 12 分 良 7 ~ 9 分
可 4 ~ 6 分 差 3 分以下

上海医科大学附属华山医院手外科
郑忆柳、劳杰
同济医科大学附属协和医院手外科
洪光祥起草

二、臂丛神经

(一)单根神经评定试用标准

分级：优 肌力 M4 以上 感觉 S3 以上
良 肌力 M3 感觉 S3
可 肌力 M2 感觉 S2
差 肌力 M0 ~ 1 S0 ~ 1

(二)上肢各关节功能评定试用标准

1. 肩关节功能评定试用标准

(1)评分标准

肩关节功能评定试用标准

分类	肩外展	肌力	肩外旋
4 分	> 90°	≥M4	> 30°
3 分	60 ~ 90°	≥M3	10 ~ 90°
2 分	30 ~ 60°	≥M2	0 ~ 10°
1 分	< 30°	< M2	< 0°

(2)综合评价

分级：优 10 ~ 12 分 良 7 ~ 9 分
可 4 ~ 6 分 差 3 分以下

2. 肘关节功能评定试用标准

(1)评分标准

肘关节功能评定试用标准

分数	屈曲	肌力	伸直	肌力	前臂旋转
4	> 90°	≥M4	0°	≥M4	正常
3	60 ~ 90°	≥M3	< - 30°	≥M3	轻度受限
2	30 ~ 60°	≥M2	- 30 ~ 50°	≥M2	重度受限
1	< 30°	< M2	> 50°	< M2	不能

(2)综合评价

分级：优 13 ~ 16 分 良 9 ~ 12 分
可 5 ~ 8 分 差 4 分以下

3. 腕关节功能评定试用标准

(1)评分标准

腕关节功能评定试用标准

分数	背伸	肌力	掌屈	肌力
4	> 45°	> M3	> 45°	> M3
3	≥30°	M3	≥30°	M3
2	< 30°	M2	< 30°	M2
1	不能	M0-1	不能	M0-1

(2)综合评价

分级：优 13 ~ 16 分 良 9 ~ 12 分
可 5 ~ 8 分 差 4 分以下

4. 手功能评定试用标准

(1)评分标准

手功能评定试用标准

分数	拇对掌	手指活动度	感觉
4	正常	指屈伸好	S4
3	能对环指	指屈伸活动为正常的 60 %	S3
2	能对示中指	指有微屈或微伸活动	S2
1	不能	指无活动	S0-1

(2)综合评价

分级：优 10 ~ 12 分 良 7 ~ 9 分
可 4 ~ 6 分 差 3 分以下

5. 臂丛功能综合评价试用标准

臂丛功能综合评价标准

分级	肩关节	肘关节	腕关节	手	上干或下干	全臂丛
优	4	4	4	4	7 ~ 8	13 ~ 16
良	3	3	3	3	5 ~ 6	9 ~ 12
可	2	2	2	2	3 ~ 4	5 ~ 8
差	1	1	1	1	1 ~ 2	1 ~ 4

上海医科大学附属华山医院手外科
顾玉东起草

中华医学会手外科学会 手部肌腱修复后评定标准

屈伸肌腱的疗效评定，宜用 1975 年美国手外科学会推荐的 TAM(total active movement, TAM)系统评定方法。即总主动活动度测定法。将掌指关节(MP)，近位指间关节(PIP)，远位指间关节(DIP)主动屈曲度之和，减去各关节主动伸直受限度之和，即为该手指总的主动活动度(TAM)。各关节伸直以 0°为准，过伸部分不计。

$$TAM = (MP + PIP + DIP) - (MP + PIP + DIP)$$

总主动活动度 = 各关节屈曲度之和 - 各关节伸直受限度之和

评定标准：

优 活动范围正常。
良 TAM > 健侧的 75 %。
可 TAM > 健侧的 50 %。
差 TAM < 健侧的 50 %。

中华医学会手外科学会 肘关节功能减损评定试用标准

肘关节损害的功能评定，由肘关节运动的丧失或

O2xydWRWZXJzaW9uLOo?

关节强直程度所决定。正常肘关节在运动时,是以屈曲和背伸为肘关节的主要功能,而其旋前、旋后功能则较次要,故前者占肘关节功能的 70 %,后者为 30 %。肘关节的正常运动幅度:屈曲由 0° 到 150°,伸直由 0° 到过伸 10°(图 1)。旋转功能:旋前由 0° 到 90°,旋后由 0° 到 90°(图 2)。丧失屈曲和背伸能力所引起的肘关节功能减损值,可按图 3 计算再乘以 70 %;旋转功能损害值可按图 4 计算再乘以 30 %,这两个数值相加即可得出肘关节的全部减损值。其对整个肢体(患肢)的功能减损值再乘以 95 %(肘关节功能减损值占肢体的百分率)即可获得。

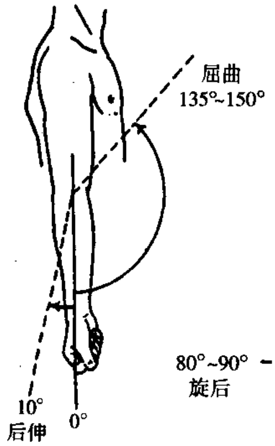

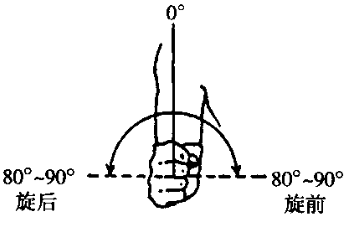

图 1 肘关节屈曲后伸 图 2 尺桡关节旋前旋后

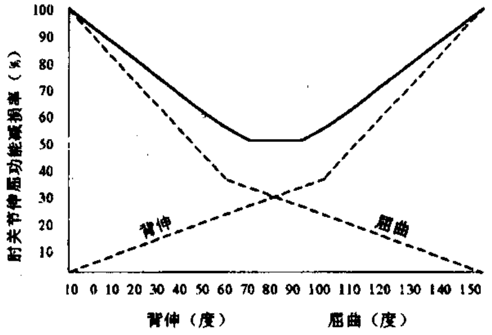

图 3 肘关节功能减损百分率

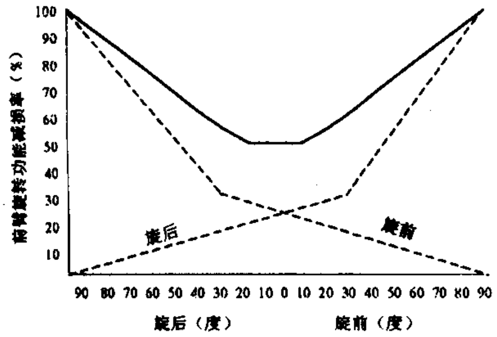

图 4 前臂旋转功能减损百分率

中华医学会手外科学会 腕关节功能评定试用标准

腕关节损害的功能评定,由腕关节运动丧失或关节强直的程度所决定。正常腕关节运动时,掌屈、背伸两功能占腕关节功能的 70 %;而腕关节的桡偏、尺偏,两者只占其功能的 30 %。腕关节屈伸的正常幅度:是从背伸 60° 到掌屈 60°(图 1);侧偏的功能位是 0° ~ 10°,腕关节侧偏的正常幅度是从桡偏 20° 到尺偏 30°(图 2)。将因丧失背伸和掌屈能力,引起的功能减损值按图 3 再乘以70 %;桡、尺偏的损害值按图 4 再乘以 30 %;这两个数值相加得出腕关节功能的全部减损值,其对整个肢体的功能损减情况只要再乘以 60 %即可获得。

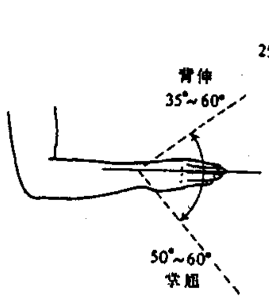

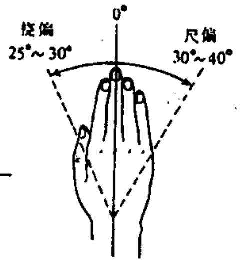

图 1 腕关节掌屈背伸 图 2 腕关节桡偏尺偏

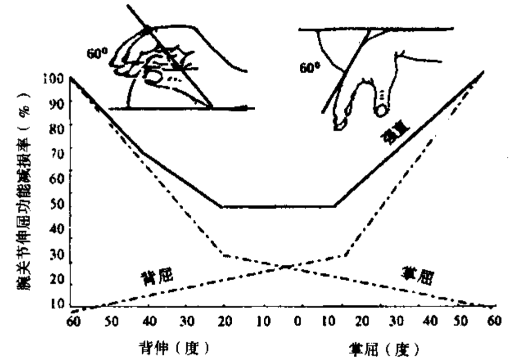

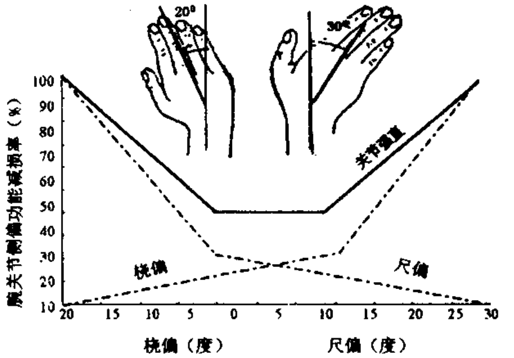

图 3,4 腕关节伸屈、侧偏功能减损百分率

南通医学院附属医院骨科 侍德起草
