# Supplementary material for: Enhanced three-dimensional visualization reconstruction for perforator flaps: A case series on clinical applications and outcomes
Source: JPRAS Open. 2026 May 14;50:344–59. doi: 10.1016/j.jpra.2026.04.015 (PMC13240778; doi:10.1016/j.jpra.2026.04.015)
Supplement: Supplementary file 2 [file mmc2.pdf]

· 临床研究 ·

Force CT 微细血管解剖成像技术及其临床应用

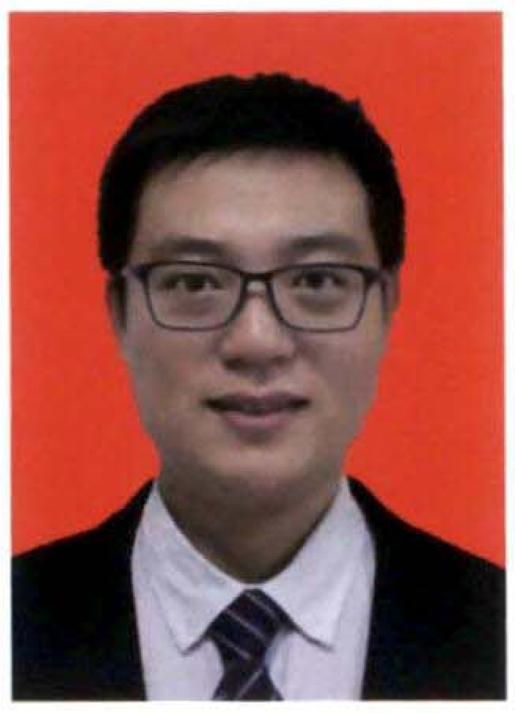

张迪[1] 齐耀东[2] 仇申强[1] 陶鸿志[3] 王增涛[1]

[1]山东第一医科大学附属山东省立医院手足外科,济南 250021;[2]山东第一医科大学附属山东省立医院影像科,济南 250021;[3]山东第一医科大学,济南 250021

通讯作者:仇申强,Email:qiushenqiang@163.com,电话:+86 151 6888 8797

【摘要】 **目的** 探讨 Force CT 微细血管解剖成像技术扫描及图像重建参数、后处理技术及临床应用效果。 **方法** 回顾 2019 年 4 月至 2021 年 6 月山东第一医科大学附属山东省立医院手足外科使用 Force CT 微细血管解剖成像技术进行术前评估的游离组织瓣移植患者共 50 例,其中男 34 例,女 16 例;年龄 3~67(平均 37)岁;包括游离皮瓣 33 例、游离复合组织瓣 10 例及手指再造 7 例,分别进行 CTA 图像评分及临床应用评价,术后采用门诊复诊、电话、微信等方式进行随访。 **结果** 50 例患者共 73 个部位的 Force CT 微细血管解剖成像评分合格率以及与临床实际的吻合率为 100%;所有组织瓣及再造手指均完全成活,未发生血管危象,无并发症。术后随访 3~15 个月,平均 11 个月,皮瓣愈合良好;7 例再造手指中 6 例外观满意,1 例因指体臃肿行整形术;所有皮瓣感觉恢复良好。 **结论** Force CT 微细血管解剖成像技术能对微细血管进行精准显示与重建,对游离组织瓣移植手术方案的设计和实施有指导意义,具有很好的推广价值。

【关键词】 微血管; CT 血管造影; 外科皮瓣; 复合组织瓣; 手指再造; 显微外科

基金项目: 山东省自然科学基金(ZR202102181095)

DOI: 10.3760/cma.j.cn441206-20220327-00059

Force CT microvascular anatomical imaging technology and its clinical application

ZHANG Di[1], QI Yaodong[2], QIU Shenqiang[1], TAO Hongzhi[3], WANG Zengtao[1]

[1]Department of Hand and Foot Surgery, Provincial Hospital Affiliated to Shandong First Medical University, Jinan 250021,China; [2]Imaging Department, Provincial Hospital Affiliated to Shandong First Medical University, Jinan 250021,China; [3]Shandong First Medical University, Jinan 250021, China

Corresponding author: QIU Shenqiang, Email: qiushenqiang@163.com, Tel:+86 151 6888 8797

【Abstract】 **Objective** To explore the scanning and image reconstruction parameters, post-processing technology and effect of clinical application of Force CT microvascular anatomy imaging technology. **Methods** From April 2019 to June 2021, 50 cases of free tissue flap transfer were reviewed in Department of Hand and Foot Surgery of Provincial Hospital Affiliated to Shandong First Medical University, including 34 males and 16 females in 3-67 (mean, 37) years old. In which, 33 cases were free flap, 10 were free composite tissue flap and 7 were digit reconstruction. CTA image score and clinical application evaluation were performed respectively. Follow-up was conducted by outpatient visit, telephone call and WeChat. **Results** The qualified rate of Force CT microvascular anatomy imaging in 73 parts of 50 patients and the coincidence rate with clinical practice were 100%. All tissue flaps and reconstructed digits survived completely. No vascular compromise and other complicatiors occurred. The postoperative follow-up was 3 to 15 months, with an average of 11 months, and the flap healed well. Of the 7 reconstructed digits, 6 were satisfactory in appearance and 1 was reconstructed with flap plasty because of digit bloat. **Conclusion** Force CT microvascular anatomical imaging technology can accurately display and reconstruct microvessels, and has guiding significance for the design and transfer of free tissue flap, which has a good promotion value.

【Key words】 Microvascular; CT angiography; Surgical flap; Composite tissue flap; Digit reconstruction; Microsurgery

Fund program: *General Program of Natural Science Foundation of Shandong Province (ZR202102181095)*

DOI: 10.3760/cma.j.cn441206-20220327-00059

显微外科手术中,血供是手术成败的关键。按照设计及切取的需要,我们将组织瓣的血供划分为 4 个要素:主干血管起源(Arise)、主要分支(Branch)、走行位置(Course)和终末支(Destination),本文称"ABCD"法。术前血供的评估对于手术方案的设计和实施具有十分重要的指导意义。常用的影像学方法种类繁多,但是都无法全面、完整、直观的评估微细血管的走行[1-4]。传统的 CTA 可以显露粗大血管的走行情况,但无法完整而稳定的显示 0.5 mm 以下的血管,如细小的皮支、肌支、骨膜支等。从 2019 年 4 月开始,我们根据皮支、骨膜支等血管终末支的解剖结构、循环状态设计特定的扫描、图像重建、图像后处理方案,采用西门子第三代双源螺旋 CT(Force CT),精准、清晰的显示和重建直径为 0.2~0.5 mm 的微细血管的解剖形态,用于指导临床。本文回顾了 2019 年 4 月至 2021 年 6 月山东第一医科大学附属山东省立医院手足外科收治的 50 例采用游离组织瓣移植治疗软组织缺损的患者,术前应用 Force CT 微细血管解剖成像技术进行血管评估,并进行游离组织瓣移植手术的病例,取得了满意效果,报道如下。

资料与方法

一、Force CT 微细血管解剖成像技术

1.CTA 扫描与重建参数及后处理技术:采用Force CT 进行扫描。采用与传统 CTA 不同的扫描与图像重建参数、后处理技术(表 1);手动阈值触发监测,根据各级动脉中强化程度的变化速度,5~10 s 内迅速决定最佳扫描时机。

2.CTA 图像评价:由经验丰富的 2 名显微外科医生和 1 名放射科医师按照"ABCD"法[A(主干血管)、B(主干血管的沿途分支)、C(皮支、骨膜支和关节支近端)、D(皮支、骨膜支和关节支的终末支)]的四要素对 VR/CR 图像进行质量评估。评分标准[5]如下:0 分,血管未显示,无法进行临床诊断;1 分,血管隐约可见,伪影明显,断续显示,不能满足临床诊断;2 分,血管清晰,连续性好,可满足临床诊断要求;3 分,血管结构清晰、连续,分支完整,完全满足临床诊断要求。

二、临床应用

1.一般资料:本组 50 例,包括游离皮瓣 33 例、游离复合组织瓣 10 例及手指再造 7 例。其中男 34 例,女 16 例,年龄 3~67 岁,平均 36 岁。

33 例游离皮瓣中,男 18 例,女 15 例;年龄 3~67 岁,平均 37 岁。外伤导致创面 29 例(挤压伤 25 例,热压伤 2 例,切割伤 2 例),瘢痕挛缩畸形切除术后创面 4 例;创面大小 2.2 cm×3.1 cm~27.3 cm×35.4 cm。包括 ALTF 16 例,腹股沟皮瓣 8 例,足部皮瓣 9 例。

10 例游离复合组织瓣病例均为男性。年龄 18~62岁,平均 41 岁。外伤导致软组织及骨缺损 6 例(均为挤压伤),肿瘤切除后骨缺损 2 例,外伤致关节毁损、择期行关节移植 2 例;创面大小 2.8 cm×11.9 cm~9.6 cm×14.7 cm;骨质缺损长度 2.7~13.8 cm。包括腓骨复合组织瓣 5 例,髂骨复合组织瓣 2 例,跖骨复合组织瓣 1 例,第 2 趾关节复合组织瓣 2 例。

7 例手指再造病例中,男 6 例,女 1 例,年龄 15~34 岁,平均 23 岁。亚急诊再造 2 例(中指 1 例,小指 1 例),择期再造 5 例(拇指 1 例,示指 2 例,中指 1 例,环指 1 例)。

2.手术方法

(1)术前设计:①供区选择:术前对双侧可使用供

表 1 CTA 扫描与重建参数及后处理技术

Tab.1 CTA scanning and reconstruction parameters and post-processing technology

参数及后处理技术<br>Parameters and post-processing technology	传统 CTA<br>Traditional CTA	Force CT 微细血管解剖成像技术<br>Force CTA
硝酸甘油 Glyceryl trinitrate	不使用	使用
管电压 Tube voltage(KV)	100~140	70~80
注射速度 Injection speed(ml/s)	4.0~5.0	6.0~7.5
触发扫描层 Monitoring section	扫描范围起始处	扫描范围中部
期相 Phase	单期相	单/多期相
层厚 Section thickness(mm)	0.5~1.0	0.5~0.75
层间距 Slice spacing(mm)	0.3~0.7	0.3
重建野 Reconstruction vision(mm^2)	300~400	<160
迭代算法 Iterative algorithm	不使用	使用
最大密度投影 Maximum intensity projection(MIP)	使用	不使用
容积再现 Volume rendering(VR)	使用单一 VR 模板	同时使用多 VR 模板
实影渲染 Cinematic rendering(CR)	不使用	使用

区均行 Force CT 微细血管解剖成像检查并重建,选出最佳供区(包括皮瓣、骨瓣、关节瓣等的位置)。②组织瓣设计:根据缺损组织的类型和创面的大小及形态,围绕终末支“D”(即皮支、骨膜支、关节支、趾固有动脉及其终段等)设计皮瓣或复合组织瓣,确定其位置及切取范围,然后向近端依次将“C”(所需皮支、骨膜支、关节支及其近端的主要行程)、“B”(主干血管的沿途分支,术中需着重分离并结扎)、“A”(主干血管)进行评估、标记。

(2) 手术过程: 手术关键环节在于根据组织瓣的“ABCD”法 4 个要素,对照 CTA 重建图像,于相应供区仔细解剖分离皮支、骨膜支及主干血管走行过程中的各分支,结扎不需要的分支。①游离皮瓣:先处理受区,切除坏死肉芽组织或瘢痕,寻找动脉、静脉及神经,彻底止血。然后,在皮瓣供区按照设计逐层切开,按需留取浅静脉、感觉神经及适宜长度的蒂部血管。皮瓣转移至受区后修复动脉、静脉、神经,供区直接缝合,残余创面植皮。②游离复合组织瓣。分两类:一类是复合组织缺损合并创面:受区清创,修整骨质两端至健康新鲜骨面,寻找动脉、静脉及神经,彻底止血;供区逐层切开,切取包含所需组织的复合组织瓣,转移至受区,固定骨质,吻合动脉、静脉和神经。另一类是足趾近侧趾骨间关节移植:先显露、去除受区不健康关节,寻找动脉、静脉及指掌侧固有神经或指背神经,彻底止血。然后于第 2 趾近侧趾骨间关节为供区,切取复合组织瓣,根据关节骨瓣长度及关节的位置,于受区关节两骨端合适位置处截骨,纵行克氏针固定,缝合肌腱,修复动脉、静脉和神经。供区取髂骨块植骨,以保留足趾长度。③手指再造:先受区准备,寻找残端动脉、静脉、神经、肌腱等,修平残端骨质至健康。根据手指缺损情况于供足踇趾设计踇趾甲瓣,必要时携带第 2 趾设计关节复合组织瓣。逐层显露第 1 跖背动脉、第 1 跖底动脉和踇趾腓侧趾底动脉(及第 2 趾胫侧趾底动脉), 截取踇趾末节适当长度与宽度的骨质(及第 2 趾关节两端骨质)。将从供足切取的组织瓣及髂骨块进行拼装组合,用克氏针固定,修复动静脉、神经和肌腱。踇甲瓣供区采用直接缝合、植皮、跖底皮瓣转移修复或游离腹股沟皮瓣修复。携带第 2 趾关节病例供区髂骨植骨以保留足趾长度。

3.术后处理及随访:术后患者卧床 7~9 d, 应用抗凝、扩容、解痉、镇痛及抗感染药物,持续烤灯照射,密切观察血运,按需换药。术后按显微外科常规定期来门诊复诊,视具体恢复情况结合电话和微信等方式进行随访。

结　　果

一、CTA 图像评价

50 例患者共 73 个部位的 Force CT 微细血管解剖图像中,均能清楚地显示主干血管起源、主要分支、走行位置和终末支(图 1~3),图像合格率 100%(表 2)。

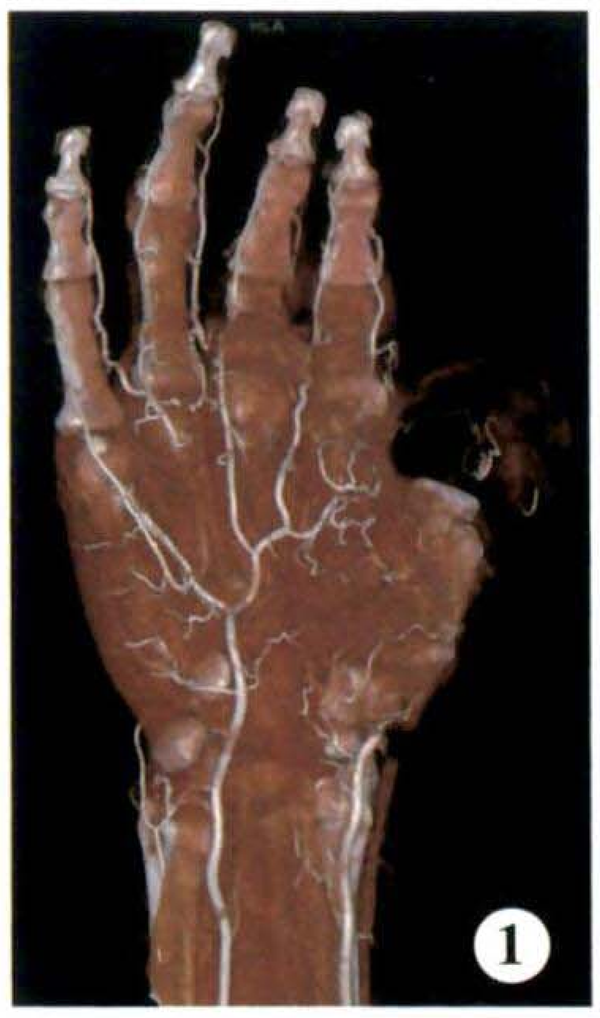

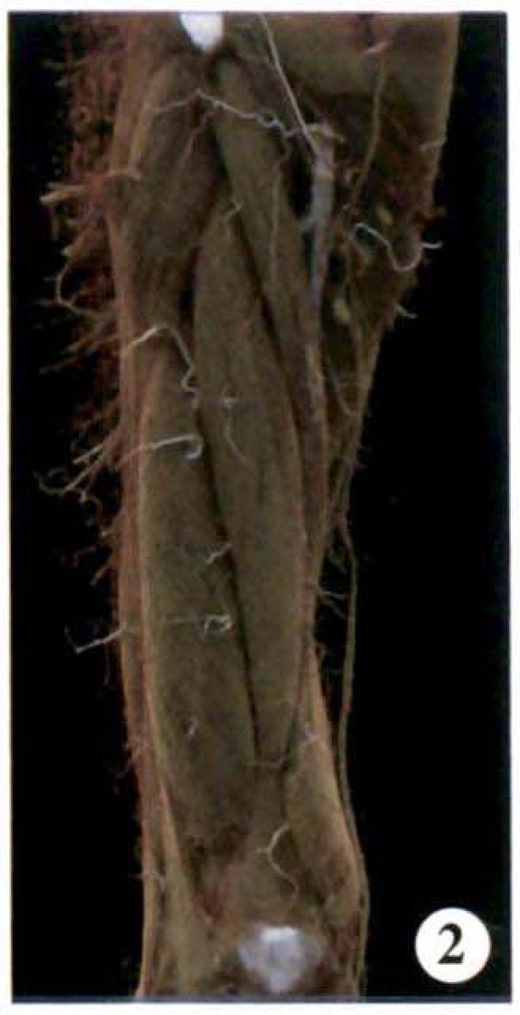

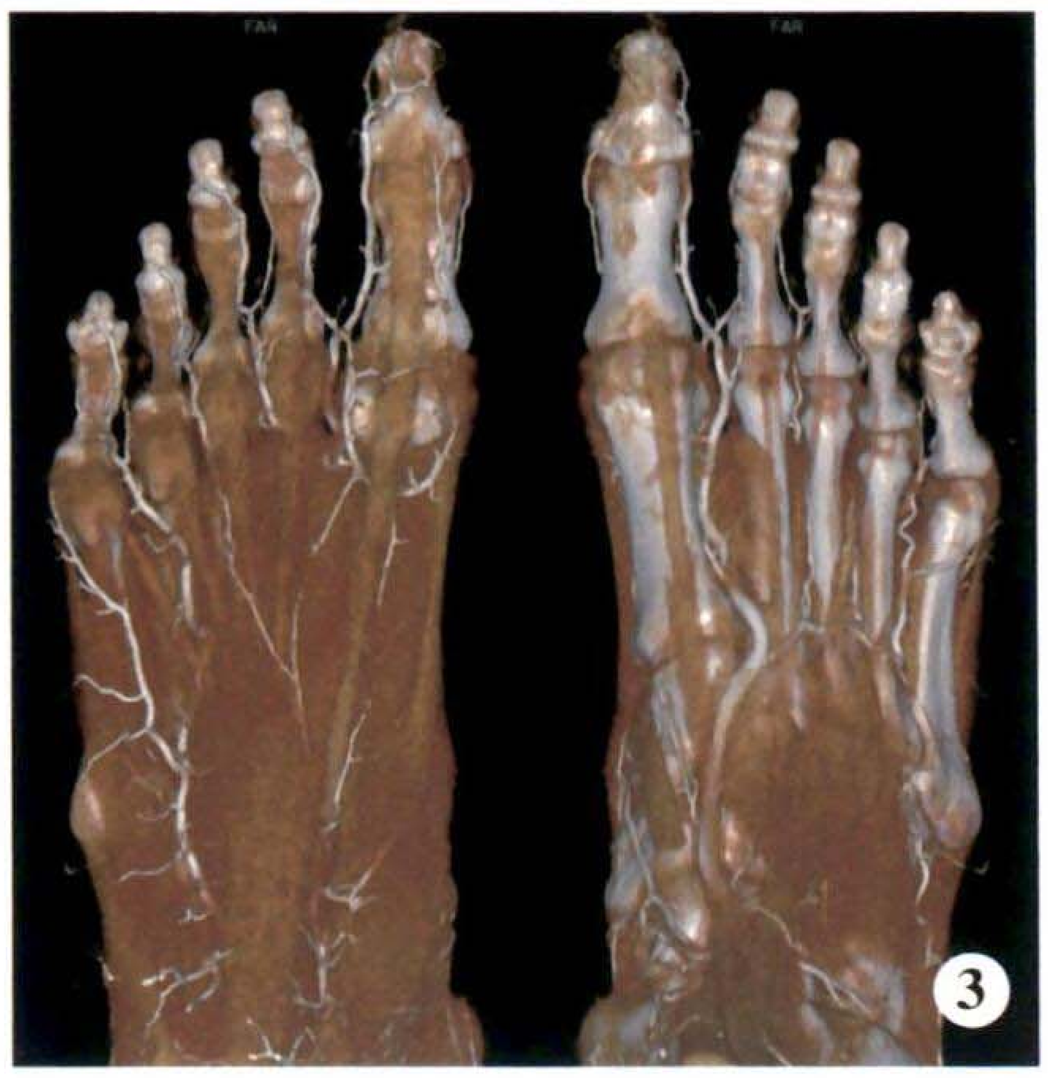

图 1　手指部动脉及皮支　**Fig.**1　Artery of digits and cutaneous branch　图 2　旋股外侧动脉降支皮支　**Fig.**2　Cutaneous branch of descending branch of lateral circumflex femoral artery　图 3　足部动脉及皮支、骨膜支　**Fig.**3　Artery of foot, cutaneous branch and periosteal branch

二、临床应用结果

所有组织瓣及再造手指均完全成活,未发生血管危象。伤口均愈合良好,无并发症。术前应用 Force CT 微细血管解剖成像技术重建“ABCD”四要素,与术中的吻合率为 100%。

术前发现解剖变异 4 例:2 例 ALTF 的主要皮支来源于股深动脉;1 例腓骨复合组织瓣需设计“Kiss”皮瓣和腓骨瓣,其近端皮瓣的穿支为高位皮支,因而,供区的血管需要两套供血动、静脉;1 例手指再造患者第 2 趾胫侧趾动脉是第 1 趾背动脉的延续(表 3)。

术后随访时间为 3~15 个月,平均 11 个月。33 例游离皮瓣质地柔软,瘢痕轻,其中 9 例因外观臃肿,二期行皮瓣整形术,6 例足底内侧皮瓣均缝接感觉神经,术后感觉恢复良好,其余病例均恢复保护性感觉;10 例游离复合组织瓣,皮瓣质地柔软,骨瓣及关节瓣骨断端均顺利愈合,无骨不连发生,2 例关节移植后关节活动度恢

表 2 本研究 CTA 图像质量评分表(例)

Tab.2 Evaluation of CTA image(Case)

血管位置 Location of vessels	部位数和修复方式 Number and repair method	3 分 3 points	2 分 2 points	1 分 1 points	0 分 0 points	合格率 Qualified rate
足趾部动脉及皮支或骨膜支 Artery and cutaneous or periosteum branches of toes	17 (游离皮瓣 9 个、游离复合组织瓣 1 个、再造 7 个)	16	1	0	0	100%
旋髂浅动脉及皮支 Superficial circumflex iliac artery and cutaneous branch	10 (游离皮瓣 9 个、游离复合组织瓣 1 个)	10	0	0	0	100%
旋股外侧动脉降支及皮支 Descending and cutaneous branches of lateral femoral circumflex artery	17 (游离皮瓣 16 个、游离复合组织瓣 1 个)	17	0	0	0	100%
腓动脉及皮支或腓骨支 Peroneal artery and cutaneous or fibula branches	4 (游离复合组织瓣 4 个)	4	0	0	0	100%
手指部动脉及皮支 Arteries and cutaneous branches of digits	25 (游离皮瓣 14 个、游离复合组织瓣 4 个、再造 7 个)	23	2	0	0	100%
总计 Total	73	70	3	0	0	100%

表 3 本研究组织瓣成活率及 CTA 图像与临床实际符合情况

Tab.3 Survival rate of flaps and coincidence rate between the CTA and clinical practice

皮瓣类型 Flap type	数量 Quantity	成活率 Survival rate	变异及病例数量 Number of anatomical variant cases	变异病例比例 Proportion of variant cases	图像与临床符合率 Coincidence rate of image and clinic
游离皮瓣 Free flap	33	100%	2 例 ALTF 皮支发自股深动脉	6.1%	100%
游离复合组织瓣 Free composite tissue flap	10	100%	1 例腓骨"Kiss"皮瓣的近端皮瓣血供来自高位皮支	10.0%	100%
手指再造 Digit reconstruction	7	100%	1 例第 2 趾胫侧趾动脉是第 1 趾背动脉的延续	14.3%	100%
总计 Total	50	100%	4	8.0%	100%

复至 70°和 80°;7 例再造病例中 6 例再造手指外观满意,1 例因指体臃肿,行皮瓣整形术,所有病例感觉均恢复良好,2 例同时携带趾骨间关节病例,术后关节活动度分别达到 65°和 80°。

典型病例

患者 男,31 岁。机器挤压伤致右中指背侧及其近端手背软组织合并骨、肌腱缺损,第 3 掌骨头残余薄层骨皮质及部分关节软骨,一期清创,骨水泥填充掌骨缺损(图 4),二期行第 1 跖骨复合组织瓣移植修复。术前 CTA 见患手动脉正常(图 5),左足第 1 跖背动脉为 II 型,可见营养腓侧跖骨的骨膜支 2 条以及细小皮支数条(图 6)。设计 13.5 cm×3.5 cm 大小的长条状皮瓣及长为 3.5 cm 的半跖骨骨瓣(图 7),形成以足背动脉为主干血管的跖骨复合组织瓣(图 8),松止血带见跖骨瓣血运良好(图 9)。移植至受区,动脉行端侧吻合。供区直接缝合。术后 3 个月复查,见外观及功能良好,掌骨骨质完全愈合,供区骨质再生良好(图 10)。

讨 论

Hou 等[6]应用"ABC"分类法来对第 1 跖背动脉进行分型,科学直观。我们借鉴该分类法,按照设计及切取的需要,将皮瓣或组织瓣的血供划分为"ABCD"4 个要素:弄清这四要素,就能够简单明了的明确组织瓣的各级血管的情况,指导设计及手术切取。

血管的终末支(如皮支、骨膜支等)微细,如何进行清晰显示与精准定位一直是困扰显微外科医师的难题[7-8]。传统的 CTA 能够初步显示组织瓣的"ABC"3个因素,而"D"(终末支)通常无法清晰的重建与显示[9-10]。为了稳定的获得高质量的终末支图像,首先,Force CT 低管电压下高管电流输出能力和独立的 ADMIRE 迭代算法是保证终末支血管显示的基础;其次,需要 350 mgI/ml 或更高浓度的碘对比剂、活塞式高压注射

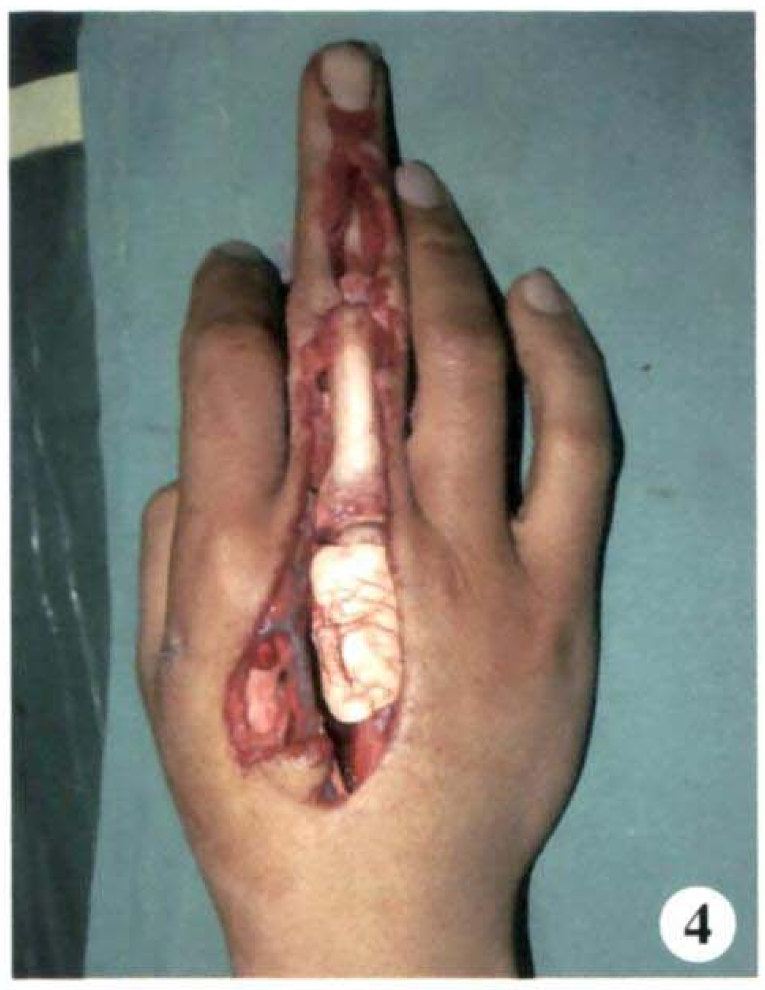

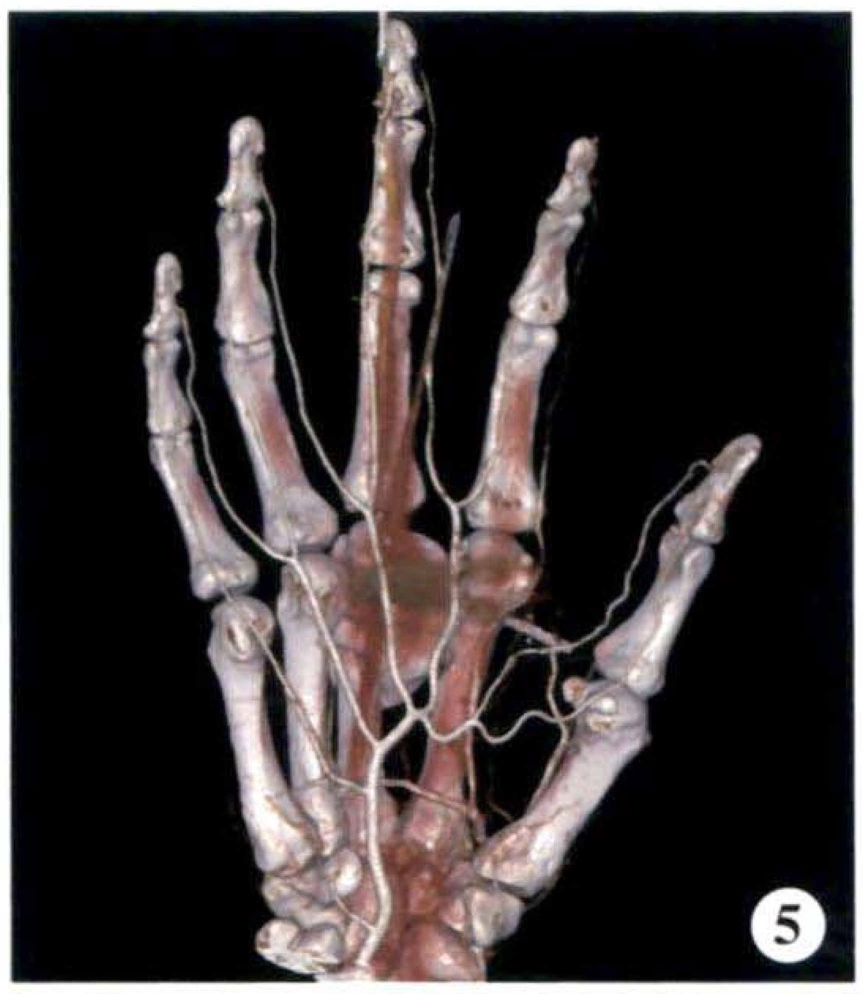

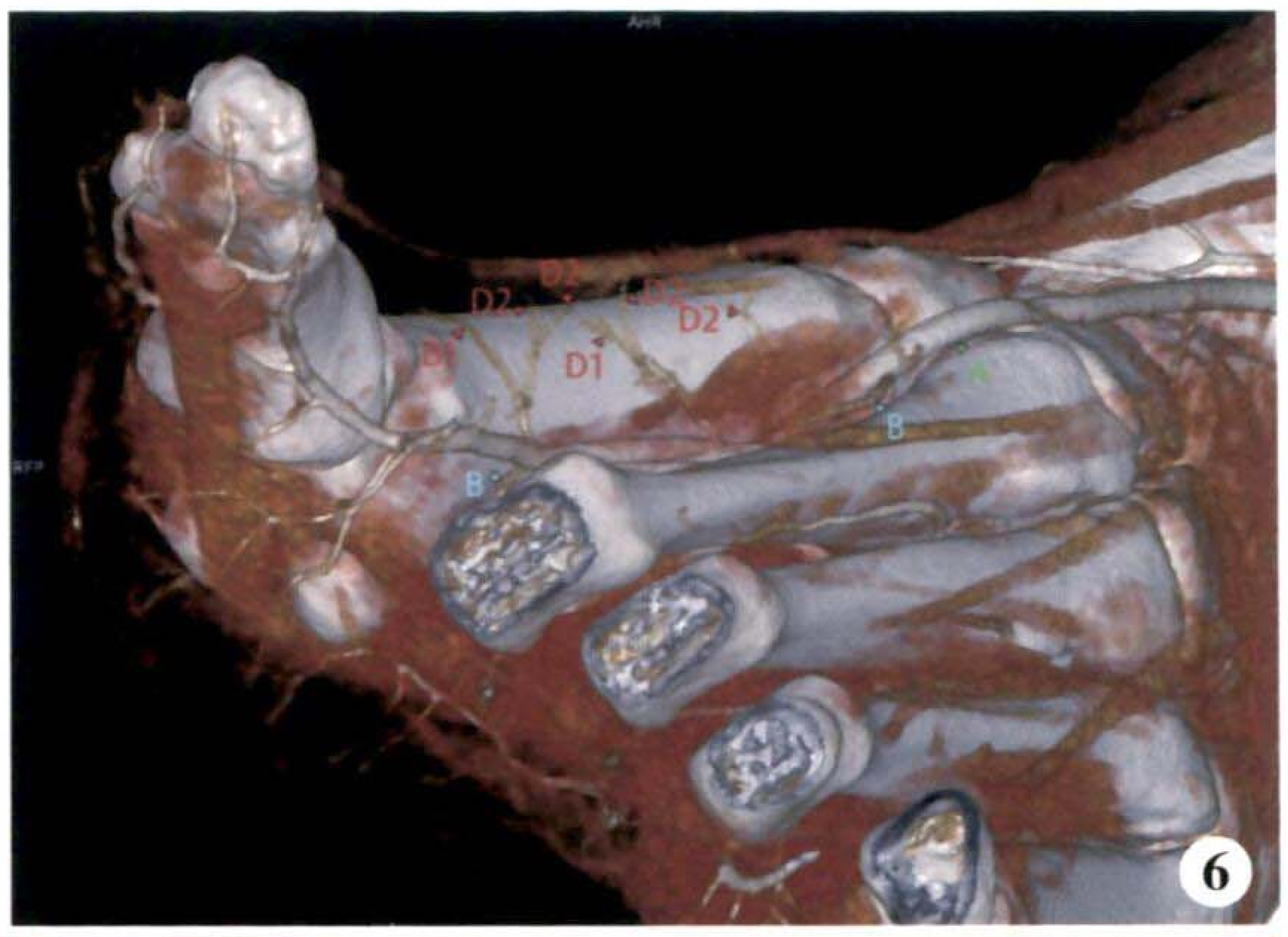

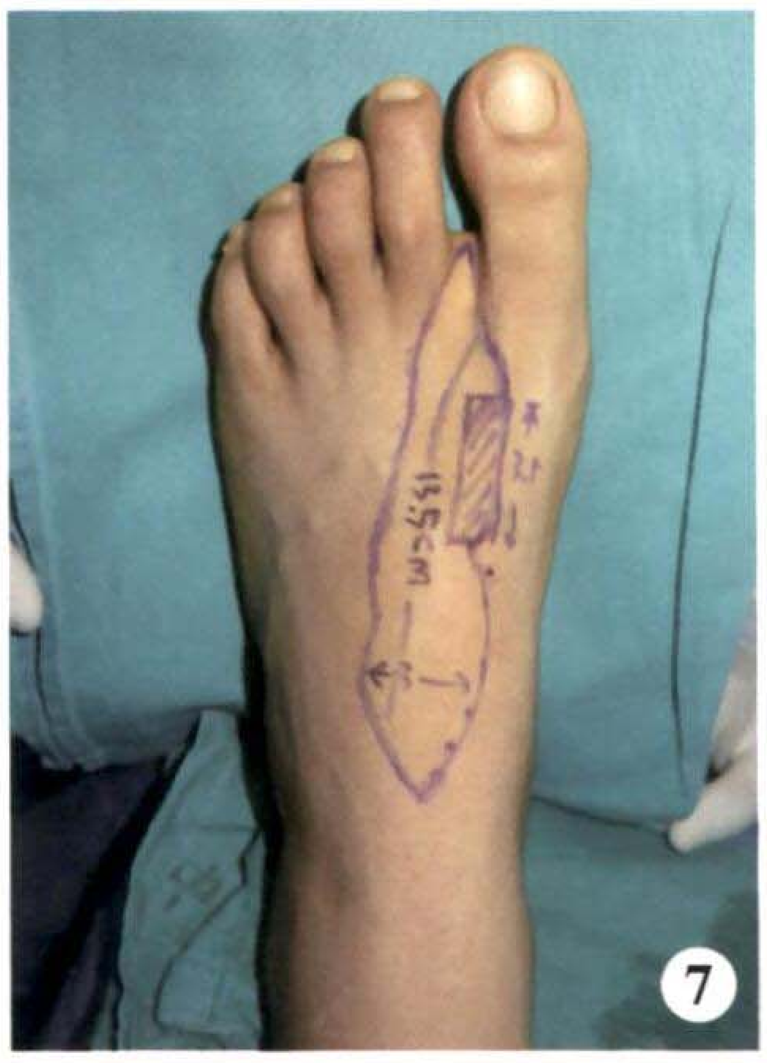

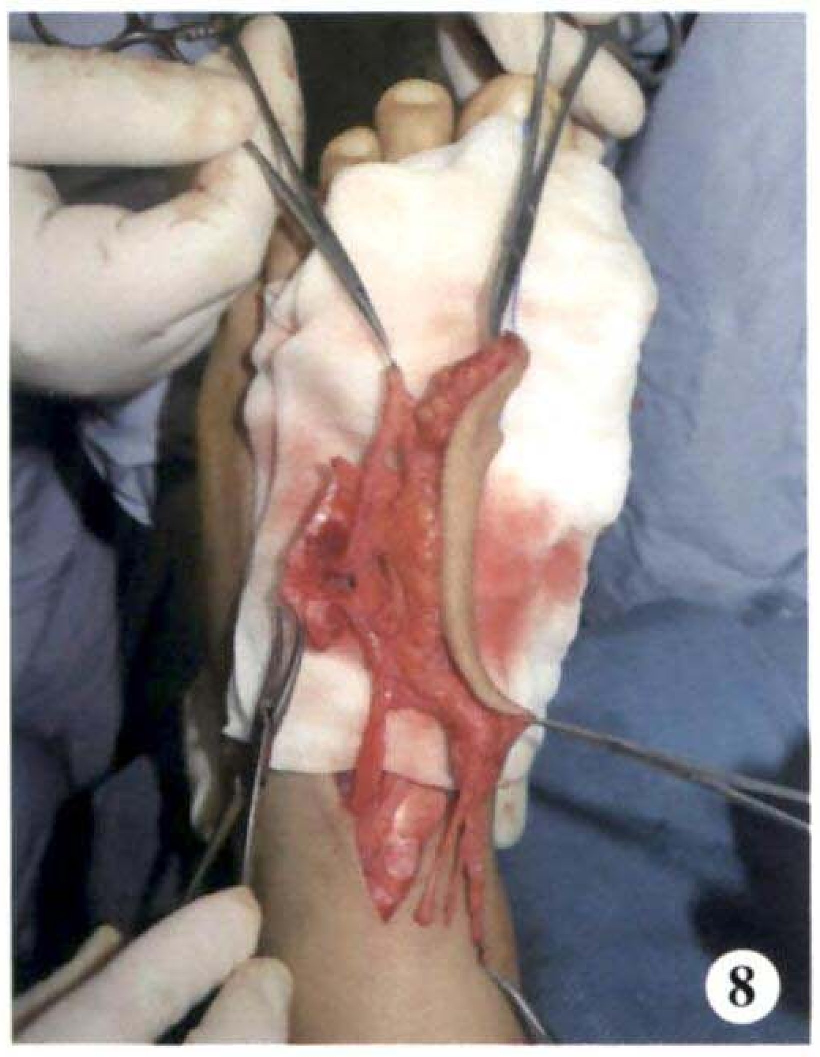

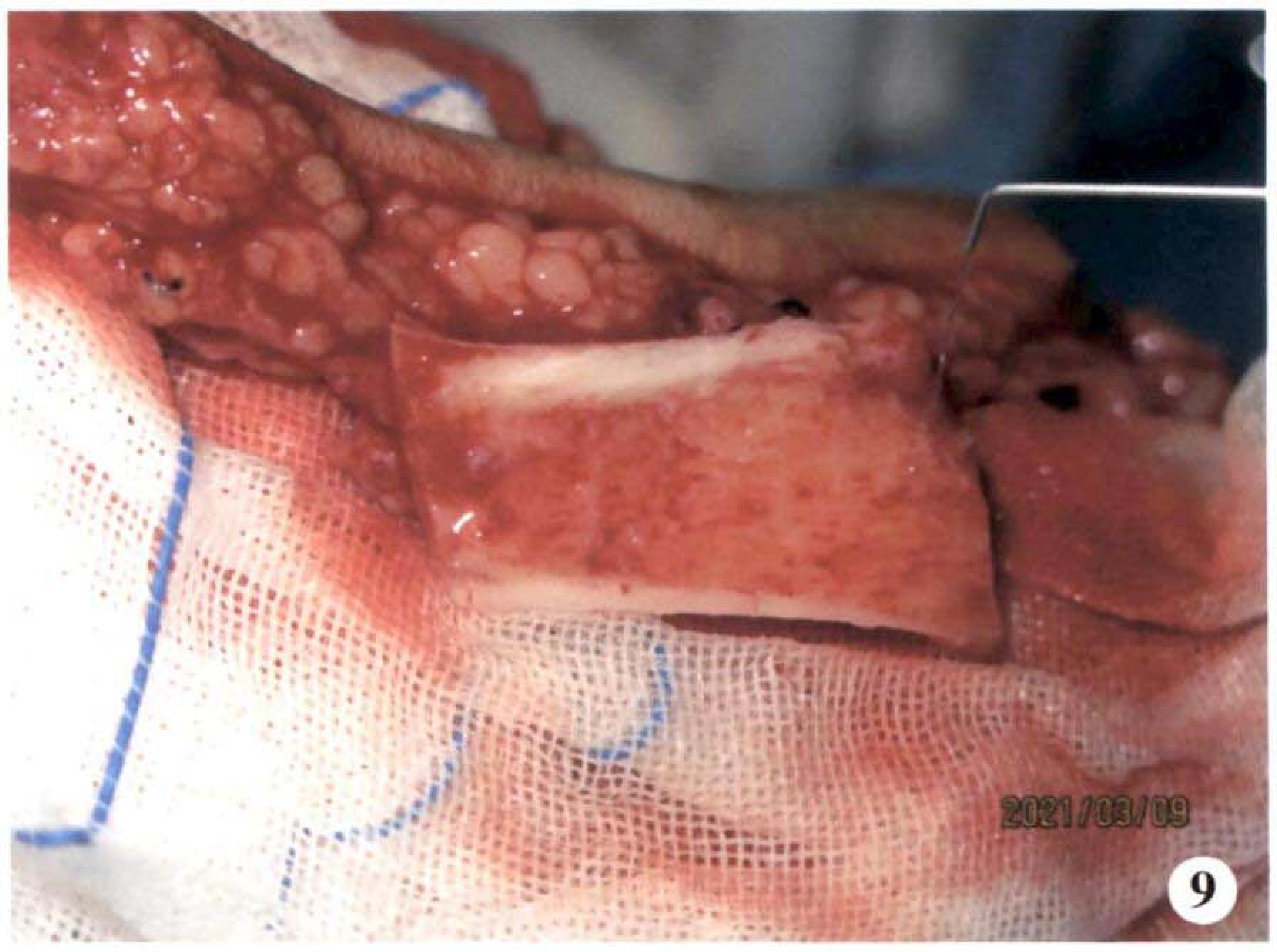

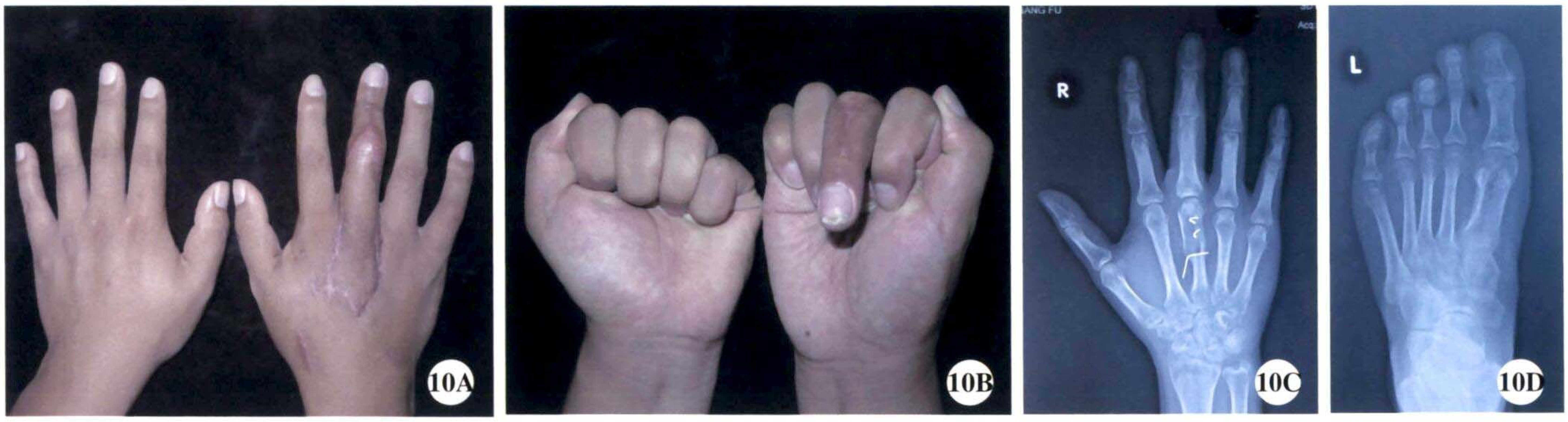

图 4　患者术前右手背面观(创面及骨水泥植入情况)　**Fig.**4　Preoperative view of dorsal right hand (with wound and bone cement implantation)　图 5　通过 Force CT 重建出受区血供情况　**Fig.**5　The arterial course of the recipient area was reconstructed by force CT　图 6　通过 Force CT 重建出供区血供情况　第 1 跖背动脉走行于第 1 跖骨与第 1 背侧骨间肌之间,可以清晰显示至第 1 跖骨的 2 条骨膜支(D1)、数条皮支(D2) 以及需要结扎的分支 (B)　**Fig.**6　The blood supply of the donor site was reconstructed by Force CT. The first dorsal metatarsal artery runs between the first metatarsal bone and the first dorsal interosseous muscle. It clearly shows 2 periosteal branches(D1), several cutaneous branches (D2) and branch(B) to be ligated to the first metatarsal bone　图 7　术前组织瓣设计　**Fig.**7 Design of preoperative tissue flap　图8　为术中复合组织瓣切取后及骨瓣(第 1 跖骨背侧半)侧面观　**Fig.**8　Lateral view of the composite tissue flap and the bone flap (dorsal half of the first metatarsal bone) during the operation　图 9　为术中复合组织瓣切取后及骨瓣(第 1 跖骨腓背侧半)跖侧观　**Fig.**9　The metatarsal side view of the composite tissue flap and the bone flap (dorsal half of the first metatarsal fibula)　图 10　术后 3 个月随访　**Fig.**10　Followed-up at 3 months after surgery　A. 双手背侧观　A. Dorsal view of both hands; B. 手指屈曲功能　B. Finger flexion; C. 受区 X 线正位片　C. Anteroposterior X-ray film of recipient site; D. 供区 X 线正位片　D. Anteroposterior X-ray film of donor site

器与 18 G 留置针,尽可能的提高对比剂注射的碘流率;次之,终末支动脉的扫描时间窗很窄,过早扫描会导致终末支动脉内对比剂浓度未达到峰值而不显影或显影不佳,过晚扫描会导致动脉的内对比剂浓度下降且伴行静脉显影,不易区分动脉和静脉,技师需要根据患者局部血流速度等个体化的差异,确定最佳扫描时机;最后,部分终末支血管在 VR 图像中与周围组织的对比度不高,通过在 MPR 图像中对微细血管进行分割,对微细血管单独设置 VR 模板,增加了终末支血管与周围组织对比度,清晰显示其与上级血管、骨骼、肌肉等组织

间的位置关系、走行等细节。

总之,Force CT 的性能优势再加上以上诸多扫描、重建后处理技术细节,使血管终末支的稳定显示获得突破性的进展,才能使所有的 CTA 图像 100%合格并获得高评分,而且与临床实际的吻合率达到 100%[11-12]。

Force CT 微细血管解剖成像技术对微细血管(0.2~0.5 mm)有更加准确、全面而又稳定的显影与重建能力,可以勾勒出自主干血管到需切取的组织的细小营养血管的全貌,显现它们与周围组织毗邻关系。因而可以术前发现血管解剖变异;可以准确判断受区血管的健康情况,确定所需血管蒂长度;可以提前标记出各级血管的主要分支的位置及毗邻,在切取过程中,针对性的分离这些分支并结扎,减少出血及损伤。这相当于在术前模拟了一次精确且无创的"解剖",改变了既往"经验性""探索式"的切取方法,使整个切取过程的目的性更强,也更精准。

需要指出的是,因为应用了硝酸甘油使外周血管有所扩张,同时由于图像层厚为 0.5 mm,因部分容积效应,CTA 显像的血管直径均大于 0.5 mm 且比临床实际的血管显得更粗,无法准确测量微细血管管腔直径,但根据临床实践有理由认为重建的血管的实际直径甚至低于 0.2 mm,需要进一步研究去证实。

综上所述,Force CT 微细血管解剖成像技术能对微细血管精准显示与重建,对游离组织瓣移植手术方案的设计和实施有指导意义,同时提高了手术安全性,具有很好的推广价值。

作者贡献声明 张迪负责实施研究、采集数据、分析数据、起草文章、统计分析;齐耀东负责实施研究、采集数据、分析数据、起草文章;仇申强负责酝酿和设计实验、实施研究、解释数据、起草文章、对文章的知识性内容作批评性审阅、统计分析、行政、技术或材料支持;陶鸿志参与实施研究、采集数据、分析数据、起草文章;王增涛直接参与并指导该研究

利益冲突 所有作者均声明不存在利益冲突

参考文献

[1] 莫勇军,杨克勤,谭海涛,等.CTA 联合彩色多普勒超声检测在股前外侧穿支皮瓣中的应用[J].中华显微外科杂志,2018,41(1):68-72. DOI:10.3760/cma.j.issn.1001-2036.2018.01.017.
Mo YJ, Yang KQ, Tan HT, et al. Application of CTA combined with color Doppler ultrasound in anterolateral thigh perforator flap[J]. Chin J Microsurg,2018,41(1):68-72. DOI:10.3760/cma.j.issn.1001-2036.2018.01.017.

[2] 周正虎,巨积辉,李雷,等.DSA 在膝降动脉-隐动脉链式穿支蒂皮瓣修复膝关节周围创面中的临床应用[J].中华显微外科杂志,2021,44(3):298-300. DOI:10.3760/cma.j.cn441206-20190702-00227.
Zhou ZH, Ju JH, Li L, et al. Clinical application of DSA in repairing the wound around the knee with the descending knee artery saphenous artery chain perforator pedicle flap[J]. Chin J Microsurg,2021,44(3):298-300. DOI:10.3760/cma.j.cn441206-20190702-00227.

[3] Vasile JV, Levine JL. Magnetic resonance angiography in perforator flap breast reconstruction[J]. Gland Surg,2016,5(2):197-211. DOI:10.3978/j.issn.2227-684X.2015.07.05.

[4] Smit JM, Klein S, Werker PM. An overview of methods for vascular mapping in the planning of free flaps[J]. J Plast Reconstr Aesthet Surg,2010,63(9):e674-e682. DOI:10.1016/j.bjps.2010.06.013.

[5] Xu L, Yang K, Wei P, et al. Computed tomography angiography allows the classification of the first dorsal metatarsal arteries[J]. J Reconstr Microsurg,2016,32(9):675-682. DOI:10.1055/s-0036-1585464.

[6] Hou Z, Zou J, Wang Z, et al. Anatomical classification of the first dorsal metatarsal artery and its clinical application[J]. Plast Reconstr Surg,2013,132(6):1028e-1039e. DOI:10.1097/PRS.0b013e3182a97de6.

[7] 罗翔,谭海涛,杨克勤,等.CTA 联合增强现实技术实施股前外侧穿支皮瓣游离移植舌再造九例[J].中华显微外科杂志,2019,42(4):339-343. DOI:10.3760/cma.j.issn.1001-2036.2019.04.007.
Luo X, Tan HT, Yang KQ, et al. The use of CTA combined augmented reality navigation in free anterolateral thigh perforator flap for tongue reconstruction: 9 cases report[J]. Chin J Microsurg,2019,42(4):339-343. DOI:10.3760/cma.j.issn.1001-2036.2019.04.007.

[8] 冯少清,喜雯婧,王珏,等.彩色多普勒超声与 CT 血管造影技术在下肢穿支血管定位中的比较[J].中华显微外科杂志,2016,39(1):26-32. DOI:10.3760/cma.j.issn.1001-2036.2016.01.008.
Feng SQ, Xi WJ, Wang J, et al. The comparison of color Doppler ultrasound and computed tomography angiography in the preoperative planning of lower extremity perforators[J]. Chin J Microsurg,2016,39(1):26-32. DOI:10.3760/cma.j.issn.1001-2036.2016.01.008.

[9] Chen SY, Lin WC, Deng SC, et al. Assessment of the perforators of anterolateral thigh flaps using 64-section multidetector computed tomographic angiography in head and neck cancer reconstruction[J]. Eur J Surg Oncol,2010,36(10):1004-1011. DOI:10.1016/j.ejso.2010.07.005.

[10] Liu SC, Chiu WK, Chen SY, et al. Comparison of surgical result of anterolateral thigh flap in reconstruction of through-and-through cheek defect with/without CT angiography guidance[J]. J Craniomaxillofac Surg,2011,39(8):633-638. DOI:10.1016/j.jcms.2011.01.004.

[11] 刘瑛,陈学志,高晓玲,等.Force CT 双能量模式不同管电压低剂量对比剂在头颈部 CTA 中的临床应用[J].中国中西医结合影像学杂志,2020,18(2):139-143.
Liu Y, Chen XZ, Gao XL, et al. Clinical application of different tube voltage low dose contrast agent in head and neck CTA with Force CT dual-energy mode[J]. Chin J Imageol Integ Trad Chin Western Med,2020,18(2):139-143.

[12] 王利军,王星,李志军,等.Force CT Turbo Flash 扫描模式联合迭代重建算法降低辐射剂量:主动脉 CTA 的比较研究[J].转化医学电子杂志,2017,4(4):19-22.
Wang LJ, Wang X, Li ZJ, et al. Force CT Turbo Flash scanning mode combined with iterative reconstruction algorithm to reduce radiation dose: a comparative study of aortic CTA[J]. J Translat Med,2017,4(4):19-22.

(收稿日期:2021-3-27)
