# Supplementary material for: Enhanced three-dimensional visualization reconstruction for perforator flaps: A case series on clinical applications and outcomes
Source: JPRAS Open. 2026 May 14;50:344–59. doi: 10.1016/j.jpra.2026.04.015 (PMC13240778; doi:10.1016/j.jpra.2026.04.015)
Supplement: Supplementary file 3 [file mmc3.pdf]

学校代码：10285
学　　号：20165232204

苏州大学
SOOCHOW UNIVERSITY

硕士学位论文

（专业学位）

Mimics 联合 CTA 辅助穿支皮瓣术前精准设计的临床应用

The Clinical Application of Mimics with CTA Assisting in the Accurate Preoperative Design of Perforator Flap

研究生姓名　惠涛涛
指导教师姓名　芮永军
专业名称　骨外科学
研究方向　数字骨科
所在院部　苏州大学附属第一医院

论文提交日期　2019 年 6 月

苏州大学学位论文独创性声明

本人郑重声明：所提交的学位论文是本人在导师的指导下，独立进行研究工作所取得的成果。除文中已经注明引用的内容外，本论文不含其他个人或集体已经发表或撰写过的研究成果，也不含为获得苏州大学或其它教育机构的学位证书而使用过的材料。对本文的研究作出重要贡献的个人和集体，均已在文中以明确方式标明。本人承担本声明的法律责任。

论文作者签名：惠涛涛 日期：2019.04.23

苏州大学学位论文使用授权声明

本人完全了解苏州大学关于收集、保存和使用学位论文的规定，即：学位论文著作权归属苏州大学。本学位论文电子文档的内容和纸质论文的内容相一致。苏州大学有权向国家图书馆、中国社科院文献信息情报中心、中国科学技术信息研究所（含万方数据电子出版社）、中国学术期刊（光盘版）电子杂志社送交本学位论文的复印件和电子文档，允许论文被查阅和借阅，可以采用影印、缩印或其他复制手段保存和汇编学位论文，可以将学位论文的全部或部分内容编入有关数据库进行检索。

涉密论文□

本学位论文属　　　　　　在_____年___月解密后适用本规定。

非涉密论文□

论文作者签名：惠涛涛　日期 2019.04.23

导 师 签 名：[illegible]　日期 2019.06.23.

Mimics 联合 CTA 辅助穿支皮瓣术前精准设计的临床应用

中文摘要

目的：探讨 Mimics 软件联合计算机断层扫描血管造影（Computed Tomography Angiography，CTA）辅助穿支皮瓣移植的临床效果及影响皮瓣大小设计的相关因素，并比较传统布样设计与 Mimics（Materialise's Interactive Medical Image Control System）软件设计的异同，为临床穿支皮瓣术前精准设计与个性化评估治疗效果提供有效的理论依据。

方法：2017年1月至2018年12月，选取40例四肢创伤皮肤软组织缺损、拟行穿支皮瓣手术治疗的患者，20例受区创面形状近似平面，20例受区创面具有明显弧度。其中男27例，女13例；年龄2～60岁，平均32.2岁。致伤原因：机器伤21例，车祸伤13例，重物挤压伤4例，高处坠落伤1例，跑步机擦伤1例。缺损部位：手背8例，腕关节5例，前臂11例，小腿7例，踝关节1例，足部8例。伤后至皮瓣修复时间3～80天，平均20.8天。受区创面均伴有骨或肌腱血管神经外露，缺损范围为6.0cm×4.0cm～29.0cm×7.0cm。术前行CTA检查，挑选合适的穿支及供区；受区采用计算机断层扫描（Computed Tomography，CT）技术进行扫描。然后将CTA及CT数据以DICOM（Digital Imaging and Communications In Medicine）格式导出影像系统，再将数据逐一导入Mimics20.0系统。数据导入完成后，整个Mimics操作界面分为四部分，左上方为冠状面图像，右上方为水平面图像，左下方为矢状面图像，右下方为对组织进行三维重建后显示的三维图像。（1）受区三维重建：在Mimics软件操作界面上，开始阈值分割，调整像素灰度值，将水平面图像中骨、血管、肌肉、脂肪和皮肤全部覆盖，重建受区的三维图像，再以Parts格式保存三维重建数据并导入3-matic软件。在3-matic操作界面中通过Mark填补缺损区域，填充成功后，即可生成软组织缺损区轮廓，再经过一系列操作后可另外单独生成软组织缺损区域。可分别测量缺损区域的长度和宽度，并利

用软件自动计算缺损区域的表面积。利用软件自带的测量工具，测量供区皮瓣的平均厚度，可生成具有厚度的缺损区域，再分别测量缺损区域的长度和宽度，利用软件自动测量缺损区域的表面积。（2）建立肌肉：先建立一个新的蒙版，在水平面图像中找到肌肉的起止点，从起点开始标记肌肉边界，一直标记到肌肉止点，即可生成水平面图像上所有层面的肌肉图像，生成三维图像并进行虚拟化。（3）穿支血管走行类型三维可视化建立：在Mimics软件操作界面上，开始阈值分割，调整像素灰度值，选择血管显示的阈值，即可生成血管三维图像，再逐一操作，分割选取所需要的血管区域，判断穿支血管的走行类型。

结果：40 例患者均成功建立受区缺损、供区穿支血管和肌肉的三维可视化模型。受区缺损包括无厚度和有厚度两部分，供区穿支血管和肌肉的三维可视化模型可清晰的显示出穿支血管的分布、走行、类型以及在体表皮肤穿出位置。我们发现，当受区创面形状近似平面时，传统布样设计和 Mimics 设计的皮瓣长度、宽度及表面积之间无统计学差异（$P>0.05$），皮瓣厚度对皮瓣大小设计无明显影响；当受区创面形状具有明显弧度时，传统布样设计和 Mimics 设计的皮瓣长度、宽度及表面积之间存在统计学差异（$P<0.05$），皮瓣厚度及创面弧度影响供区皮瓣大小设计，供受区无法完全匹配。40 例皮瓣，术前三维重建发现 76 支穿支血管，包括 58 支肌皮穿支血管，18 支肌间隙穿支血管；术中发现 76 支穿支血管，包括 58 支肌皮穿支血管，18 支肌间隙穿支血管。术前三维重建穿支血管走行与术中发现结果进行 kappa 值一致性检验，kappa 值=1，表明三维重建定位穿支血管走行类型与术中探查相一致。40 例手术中，股前外侧皮瓣 38 例，上臂外侧皮瓣 2 例；皮瓣最小面积为 $7.0\times4.5cm^2$，最大面积 $31.0\times7.0cm^2$。38 例单叶皮瓣，2 例分叶皮瓣。35 例供区一期直接缝合，5 例供区植皮，但无因供受区不匹配而增加第二供区损害。40 例中无一例出现血管危象。所有皮瓣术后随访 8 周顺利存活。术后随访 3-6 个月（平均 4.8 个月），皮瓣色泽、质地优良，皮瓣供、受区外形满意。术后所有皮瓣均顺利成活，皮瓣受区与供区均一期愈合。

结论：1.当受区创面近似平面图形时，供区皮瓣厚度对皮瓣大小设计无明显影响。2.当受区创面具有明显弧度时，创面弧度与皮瓣厚度均影响皮瓣大小设计，且弧度越大，皮瓣越厚，需切取更大面积的皮瓣方能覆盖同样大小的创面。3.三维穿支血管可以准确无误判断其分布、走行及类型，可为临床分离血管提供依据。4.Mimics软件联

合CTA可以实现穿支皮瓣术前精准设计，实现个性化治疗。

关键词： Mimics；CTA；穿支皮瓣；皮瓣厚度；创面弧度

作　　者：惠涛涛
指导老师：芮永军

The Clinical Application of Mimics with CTA Assisting in the Accurate Preoperative Design of Perforator Flap

Abstract

Objective: To explore the clinical effect of Mimics with computed tomography angiography (CTA) assisting in the transplantation of perforator flap and the factors influencing the size of flap design, and compare the similarities and differences between traditional cloth sample and Materialise's interactive medical image control system (Mimics) software design to provide effective theoretical basis for clinical preoperative accurate perforator flap design and individualized evaluation of treatment outcomes.

Methods: Between January 2017 and December 2018, 40 patients with skin and soft tissue defects due to limbs trauma who were about to undergo perforator flap surgery, 20 of them had approximately planar wound surface and 20 of them had obvious radian surface. There were 27 males and 13 females, with an average age of 32.2 years (range, 2-60 years). The time from post-traumatic admission to flap repair was 3-80 days (mean, 20.8 days). The causes of injury included machine injury in 21 cases, traffic accident in 13 cases, heavy crush injury in 4 cases, fall injury in 1 case and treadmill abrasion in 1 case. The wounds were located at the dorsum of hand in 8 cases, the wrist in 5 cases, the forearm in 11 cases, the leg in 7 cases, the ankle in 1 case and the foot in 8 cases. All wounds in the recipient site were combined with the exposure of bone, muscle tendon, blood vessel or nerve. The size of wounds ranged from 6.0 cm×4.0 cm to 29.0 cm×7.0 cm. Preoperative CTA examination was performed to select the appropriate perforator and donor site. Computed Tomography is used to scan the recipient site preoperatively. After scanning, CTA and CT data were imported into the Imaging system In DICOM (Digital Imaging and Communications in Medicine) format, followed by Mimics20.0 system. After data import, the Mimics operation interface is divided into four parts: the coronal plane image on the upper left, the horizontal plane image on the upper right, the sagittal plane image on the lower left, and the 3D image displayed after 3D reconstruction of the tissue on the lower right. (1) three-dimensional reconstruction of the receiving area: on the operating interface of Mimics software, threshold segmentation was started, pixel gray value was adjusted,

and all the bone, blood vessels, muscle, fat and skin in the horizontal image were covered to reconstruct the three-dimensional image of the receiving area. Then the 3D reconstruction data were saved in Parts format and imported into the 3-matic software. Mark was used to fill the defect area at the 3-matic interface. After successful filling, the contour of the defect area of soft tissue could be generated. After a series of operations, the defect area of soft tissue could be generated separately. The length and width of the defect area can be measured respectively, and the surface area and volume of the defect area can be calculated automatically by using the software. The thickness of the skin flap in the donor area can be measured by using the measurement tool provided by the software, and the defect area with thickness can be generated. Then the length and width of the defect area can be measured respectively, and the surface area and volume of the defect area can be automatically measured by the software. (2) establishment of three-dimensional visualization of the source of perforating blood vessels: on the Mimics software operating interface, threshold segmentation is started, pixel gray value is adjusted, and the threshold displayed by the blood vessels is selected to generate 3d images of the blood vessels. Then, the desired blood vessel regions are segmented and selected one by one. (3) muscle building: first, establish a new mask, find the starting and ending points of muscles in the horizontal image, mark the muscle boundary from the starting point to the muscle stopping point, then generate muscle images at all levels of the horizontal image, generate 3d images and conduct virtualization, and judge the direction of blood vessels.

Results: All the 40 cases successfully established three-dimensional visualization models of recipient area, perforating vessel and muscle. Defects in the recipient area include thickness without thickness and thickness with three-dimensional visualization models of perforating vessels and muscles clearly showing their distribution, walking, type and perforating branches in the skin surface. We found that when the wound surface of the affected area was approximately flat, there was no statistical difference between the surface area of the traditional layout design and the Mimics design ($P>0.05$), and the thickness of the flap had no significant impact on the flap design. When the shape of the wound in the receiving area was significantly curved, there was a statistical difference between the surface area of the traditional cloth sample design and the Mimics design ($P<0.05$). The thickness of the flap and the wound curvature affected the design of the flap size in the donor area, and the donor and receiving area could not completely match. In 40

cases of skin flaps, 76 perforating vessels were found in the preoperative three-dimensional reconstruction, including 58 perforating vessels of musculocutaneous and 18 perforating vessels of intermuscular space. During the operation, 76 perforating vessels were found, including 58 musculocutaneous perforating vessels and 18 intermuscular perforating vessels. Preoperative and intraoperative outcomes of three-dimensional reconstruction of perforator blood vessels were evaluated by kappa coherence test, and the three-dimensional reconstruction of perforator blood vessels was consistent with intraoperative detection, with kappa coherence test =1. In the 40 cases, there are 38 cases anterolateral femoral flaps and 2 lateral upper arm flaps. The minimum area of the flap was 7.0*4.5cm^2 and the maximum area was 31.0*7.0cm^2. There were 38 cases with a single flap and 2 cases with split flap. The donor sites in 35 cases were directly sutured and skin grafting was performed in 5 cases. There is no additional damage to the second donor region due to mismatch of the recipient region. There was no vascular crisis in 40 cases. All flaps survived successfully after 8 weeks of follow-up. The patients were followed up for 3-6 months (3.8 months on average) after surgery. The color and texture of the flap were excellent, and the shape of the flap donor and recipient area was satisfactory. All the skin flaps survived successfully after the operation, and the recipient and donor areas of the skin flaps all healed in one stage.

Conclusions: 1. When the wound surface of the recipient site is approximately planar, the thickness of the flap in the donor site has no significant influence on the design of the flap size.2. When the wound surface in the recipient site has an obvious radian, both radian wound and skin flap thickness affect the design of skin flap size. The larger the radian, the thicker the skin flap is, and a larger area of skin flap is needed to cover the same wound surface.3. Three-dimensional perforating vessels can accurately determine the course of the vessel and provide a basis for clinical blood vessel separation.4. Mimics combined with CTA can realize accurate preoperative evaluation of perforator flap and realize the personalized design.

Key words: Mimics; CTA; Perforator Flap; Flap Thickness; Flap Radian

Written by: Hui Taotao
Supervised by: Rui Yongjun

目　录

前　言......1
一、材料和方法......4
（一）实验材料......4
1、主要仪器及设备......4
2、实验对象......4
（二）实验方法......4
1、供区 CTA 及受区 CT 数据的扫描......4
2、受区缺损、供区肌肉及穿支血管三维图像的建立......5
3、皮瓣厚度与皮瓣表面积的测量与计算......15
4、按照上述方法完成 40 例皮瓣厚度以及皮瓣表面积的测量与计算，统计数据......16
5、统计学分析......16
二、实验结果......17
（一）平面组......17
1、皮瓣长度......17
2、皮瓣宽度......18
3、皮瓣厚度......19
4、皮瓣表面积......21
（二）弧度组......22
1、皮瓣长度......22
2、皮瓣宽度......23
3、皮瓣厚度......24
4、皮瓣表面积......26
（三）穿支血管走行类型......27
三、讨　论......28

一、Mimics 联合 CTA 在穿支皮瓣术前精准设计研究中的意义......29
二、皮瓣大小设计的影响因素......30
三、传统布样设计与 Mimics 软件设计的异同......30
四、建立穿支血管在肌肉骨骼中走行的临床意义......31
五、相关临床意义......31
六、创新性......31
七、实验的不足......32
四、结　论......33
参考文献......34
附录......38
综　述　数字化技术在组织移植中的发展应用......41
参考文献......49
中英文缩略词表......55
攻读学位期间公开发表的论文......56
本研究得到以下基金资助......57
致　谢......58

前

随着交通运输、工农业的迅速发展，
见[1-6]。由于软组织缺损不可避免地会出
和软组织感染、骨外露、骨折不愈合或
现全身并发症等[7-10]，因此，骨科医师
者后期治疗相当困难，最终截肢是唯一
担。随着治疗手段的不断提高，软组织缺损修复重建的发展逐渐走向成熟，并且尽早关闭创面已经成为软组织缺损修复公认的基本原则[11]。近年来，随着显微外科技术的提高，皮瓣移植覆盖技术已日渐成为四肢大面积软组织缺损重建的主要手段[12-14]。

精准的皮瓣覆盖主要依赖于形状设计与穿支定位技术。传统布样是目前临床上供区与受区之间形状转化的主要手段，但该方法目前仍停留在手工测量和目测估计上，制定手术方案缺乏标准化、规范化，且手术方案受医师的临床经验，习惯等主观思维因素影响较大，也无法考虑供区厚度与受区创面弧度，术后供受区常出现不匹配现象，导致供区皮瓣无法完全覆盖受区创面，需增加手术次数，牺牲第二供区。穿支定位技术目前主要包括彩色超声多普勒（Color Duplex Sonography，CDS）、计算机断层扫描血管造影（Computed Tomography Angiography，CTA）和核磁共振血管造影（Magnetic Resonance Angiography，MRA）[15-22]。虽然多项研究表明，CDS穿支定位检测阳性率超过90%[23-24]，穿支走行准确率可达100%[25]；CTA是穿支皮瓣术前定位的金标准[26-27]，MRA穿支定位准确率可达97%，穿支的位置及肌肉走行准确率为100%[28-30]。但CTA和MRA仅能在断层中观察血管走行，并且这些技术只能输出二维定位图像，难以三维观察穿支血管在肌肉中走行。因此对于经验欠缺的临床医师来说，术前未能明确穿支血管在肌肉中的三维走行，术中盲目分离供区血管，可能导致局部血运破坏。因此，术前皮瓣设计如何考虑供区皮瓣厚度与受区创面弧度，如何将传统的二维平面图像转变为三维立体图像，建立穿支血管三维模型，目前鲜有专题报道，仍需进一步研究。唐举玉等[31]提出显微削薄穿支皮瓣技术，即体胖患者的手（腕）、足（踝）、肘与膝关节周围、颈部等区域软组织缺损，不削薄则需切取更大面积的皮瓣方能覆盖同样大小的创面，但他并没阐述皮瓣厚度对皮瓣设计的影响机理。另外，该技术虽然明

显减小供区皮瓣厚度，但仍未考虑受区创面弧度的存在，术后依然会影响供受区匹配。而糜菁熠等[32]认为，尽管皮瓣一期削薄可明显改善其外观，但该术式明显增加了手术时间和对皮瓣本身的创伤，可能导致术后皮瓣肿胀而影响影响皮瓣血运。在穿支定位技术方面，CTA 是临床用于皮瓣穿支定位的常用方法，且目前被认为是金标准。Li 等[34]研究发现，与传统方式相比，利用 CTA 重建腓肠神经营养皮瓣指导修复足踝部皮肤软组织缺损，可明显缩短手术时间，提高皮瓣成活率。黎健伟等[33]报道了三维重建技术在髂骨瓣中的初步应用。还有研究者运用数字化技术重建乳房获得更加美观的外形。但是，利用 CT 扫描自带的软件所获得的图像不仅层次及毗邻结构显示不清，而且难以三维观察穿支血管在肌肉中走行，因此缺乏临床指导意义。

近年来，随着数字化影像技术和计算机图像处理技术的发展，数字化技术在医学领域的应用日益广泛，同时在修复重建领域已相继有学者应用该技术辅助骨瓣、皮瓣应用的报道。目前国内外广泛应用的三维重建软件 Mimics 可以利用 DICOM 格式的影像学资料完成三维重建，重建后的三维模型可以动态旋转观察，任意切割显示内部解剖结构，也可以对其进行编辑、修改，临床医生可以更深入细致地对病灶进行定位、定性、定量分析，在此基础上可以进行术前手术方案设计及模拟，同时可以进行生物力学有限元分析[35]。Mimics 软件是广泛用于数字医学的一个计算机软件，它是比利时 Materialise 公司发明的一种交互式医学影像控制系统。它能将 CT、MRI 等二维数据转换为三维数字模型，然后输出通用的计算机辅助设计(Computer Aided Design，CAD)等格式，可在个人计算机上进行大规模的数据转换处理，从而实现术前手术模拟及可视化操作。目前 Mimics 软件已广泛应用于骨科、神经外科、显微外科、整形外科、临床教学等多个领域。在显微外科领域，主要通过 CTA 扫描获得原始图像，利用 Mimics 软件对供区穿支血管进行三维可视化重建，并利用其自带软件进行虚拟模拟穿支皮瓣的设计与切取。唐举玉、徐永清等[36-37]利用 Mimics 结合 CTA 获得穿支皮瓣三维可视化模型，完成了皮瓣的个体化设计。Michael P. Chae 等[38]通过健侧肢体镜像重建患侧缺损，并进行 3D 打印缺损形状，虽然完成个性化皮瓣设计，但供受区皮瓣并不完全匹配。目前国内外研究，依然没有相关皮瓣厚度及创面弧度等研究。随着重建技术的日渐成熟，利用皮瓣的三维模型进行皮瓣厚度和表面积研究即皮瓣的空间研究将是一个新的方向，其主要目的是建立皮瓣厚度三维模型，这对修复重建领域具有深远的意义。整形外科已将 Mimics 软件广泛用于乳腺表面积、体积和形状的计

算。Michael P. Chae 等[39]利用数字化软件术前计算乳腺形状体积，间接考虑皮瓣厚度，从而实现了乳腺外形的精准设计。综上所述，对于四肢皮瓣设计研究，目前并没有无供区厚度及创面弧度以及穿支血管在肌肉中走行的研究结果，本研究旨在通过对创面 CT 扫描以及供区 CTA 扫描，运用 Mimics 三维重建技术，将皮瓣厚度加入皮瓣设计中，计算皮瓣表面积，包括无厚度和有厚度时的表面积，比较两组之间的变化，并建立穿支血管在肌肉中走行的三维模型，明确血管的走行，从而实现供受区精准匹配，为临床穿支皮瓣术前精准设计与个性化评估提供有效的理论依据。

一、材料和方法

（一）实验材料

1、主要仪器及设备

64 排螺旋 CT 机	GE 公司，美国
碘海醇造影剂	上海通用电气药业有限公司，中国
Mimics20.0 软件	Materialise 公司，比利时
3-matic12.0 软件	Materialise 公司，比利时
EOS 5D Mark IV 照相机	Cannon 公司，日本

2、实验对象

所有患者均签署知情同意书，获得苏州大学伦理委员会及无锡市第九人民医院伦理委员会批准。纳入标准：自 2017 年 1 月至 2018 年 12 月，选取 40 例四肢创伤皮肤软组织缺损、拟行穿支皮瓣手术治疗、无检查禁忌症的病人；排除标准：异常或重大病史、血管相关疾病、肾功能不全。术前供区行 CTA 检查，受区行 CT 扫描。

（二）实验方法

1、供区 CTA 及受区 CT 数据的扫描

（1）供区主干血管及其穿支血管的三维可视化重建：40例均无异常疾病史，无血管病变史、无肾功能不全病史，均获得患者同意并签署知情同意书。在行CTA检查前所有患者均行碘过敏试验。设置CTA扫描参数：100kV、250mA、层厚5.0mm（最后可拆成0.7mm的原始图像）。将扫描图像数据输入到Mimics图像工作站。利用Mimics图形工作站自身所携带的工具根据不同的像素灰度值分别对选定范围内动脉及软组织进行提取，采用表面重建的方法对各提取结构进行三维重建。

（2）受区创面扫描前，去除石膏等外固定物，患者下肢扫描采用水平仰卧位，上肢扫描采用俯卧位。设置CT扫描参数：120kV、250mA、层厚2.5mm（最后可拆成1.0mm的原始图像）。扫描范围包括创面远近端5.0cm。扫描完毕后以DICOM格式导出CT扫描数据。

2、受区缺损、供区肌肉及穿支血管三维图像的建立

2.1 数据的导入

（1）运行 Mimics 软件，点击主界面左上方的文件按钮，选择新的项目向导，会自动弹出图像选取对话框。

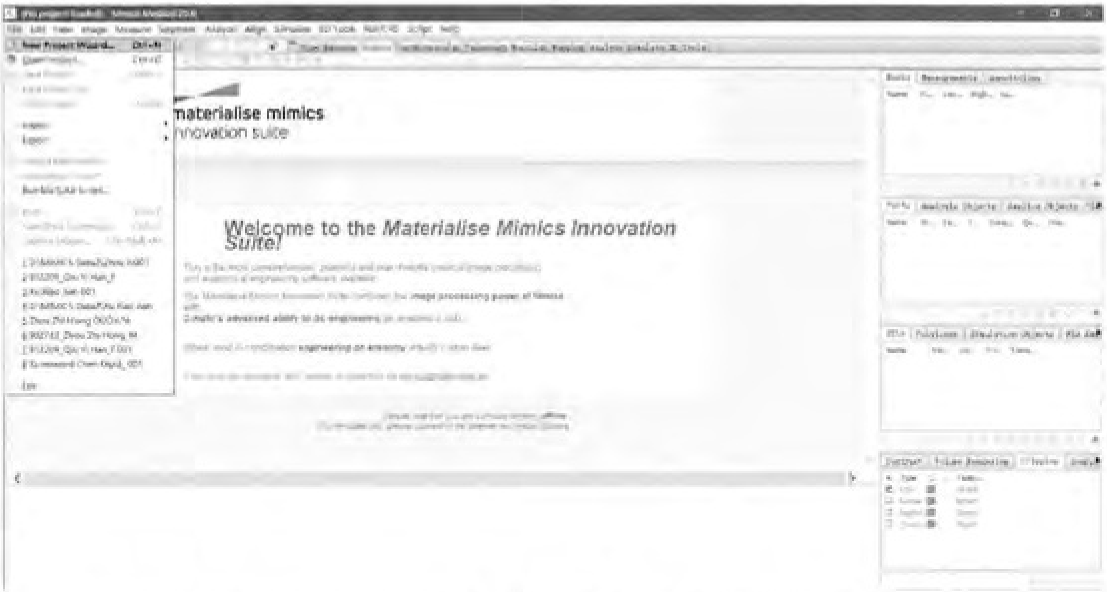

（2）在主界面 File name 中选定 CTA 或 CT 数据所在的文件夹，然后在主界面下方的 Target folder 中打开选定 CTA 或 CT 的同一位置的文件夹，一直点击 Next，点击转化（Convert）完成数据转化，生成 CTA 或 CT 原始图像。

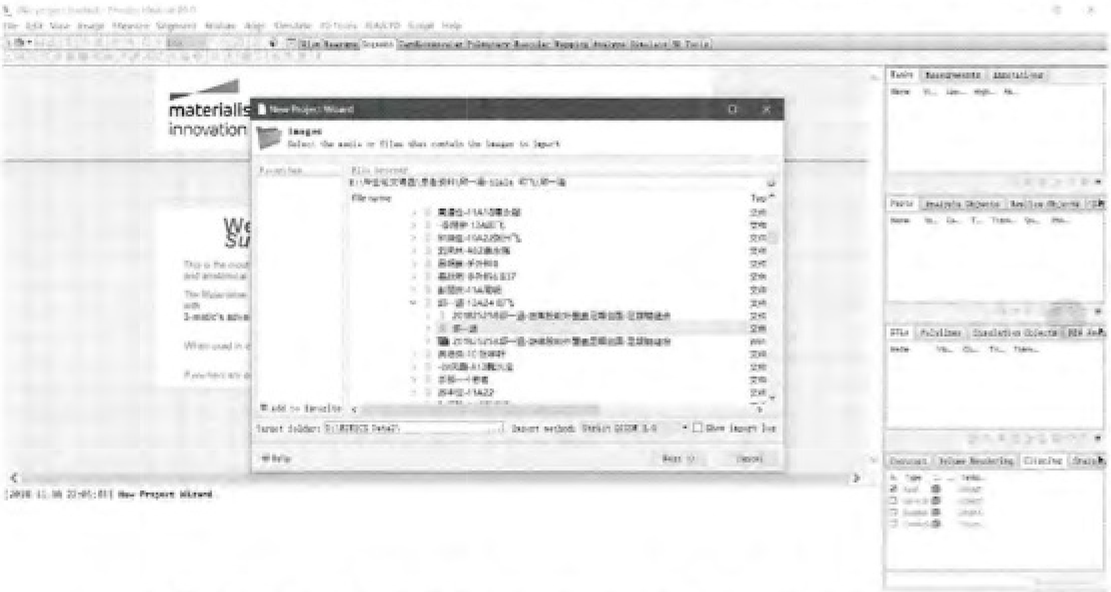

（3）图像观察面由三个面组成，除了原始水平面外，软件还将自动重组矢状面和冠状面的连续断层图像，需要在冠状面和矢状面根据人体的解剖结构来确定供受区各个方位，明确解剖体位下供受区前后左右的位置。

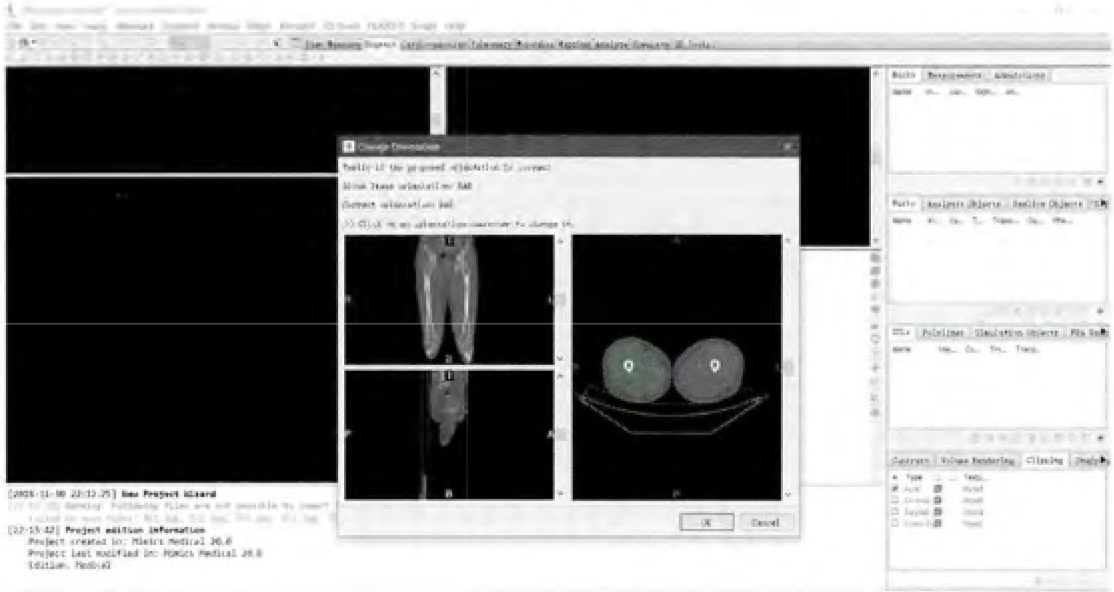

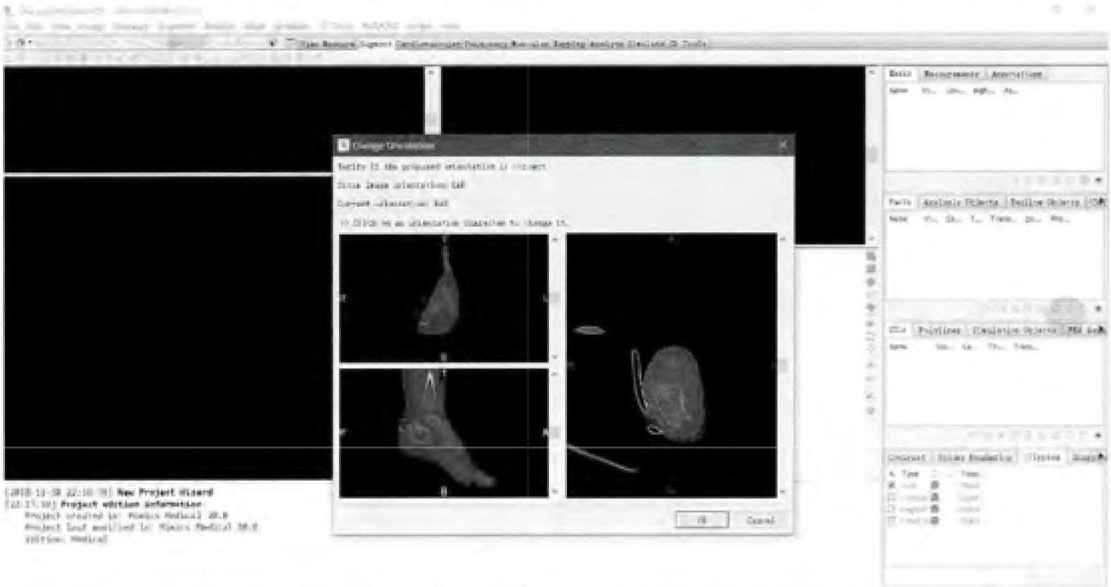

（4）完成数据的导入，整个操作界面分为四部分，左上方为冠状面图像，右上方为水平面图像，左下方为矢状面图像，右下方为对组织进行三维重建后显示的三维图像。

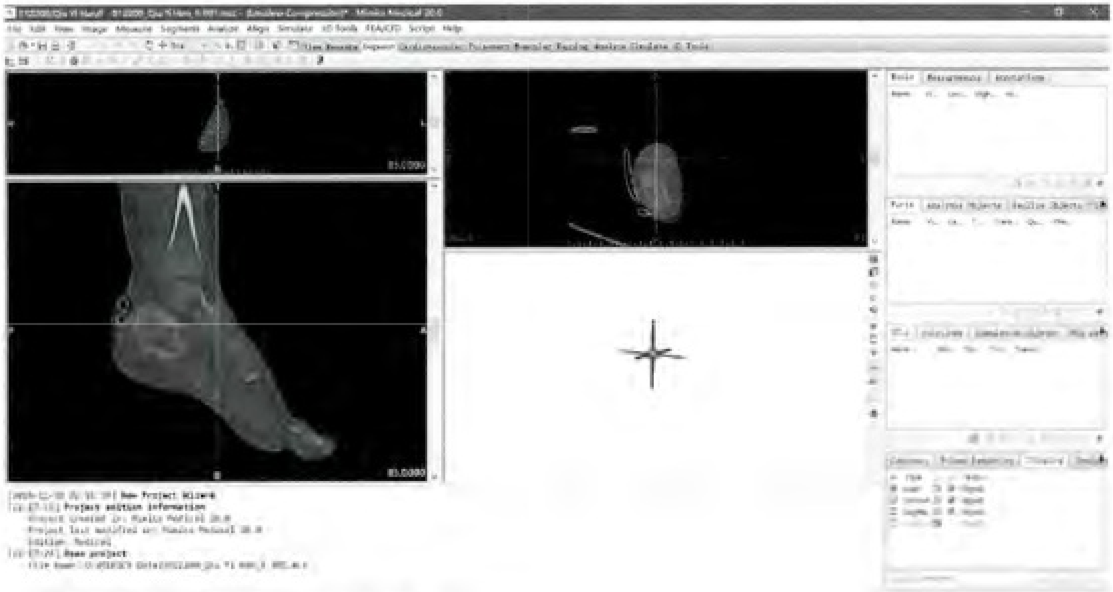

2.2 受区缺损三维图像重建

（1）点击开始阈值分割（Start Thresholding）选项，点击 Custom，调整像素灰度值，将水平面图像中骨、血管、肌肉、脂肪和皮肤全部覆盖。利用区域增长（Region Growing）及裁剪蒙版（Crop Mask）将缺损单独分离出来。

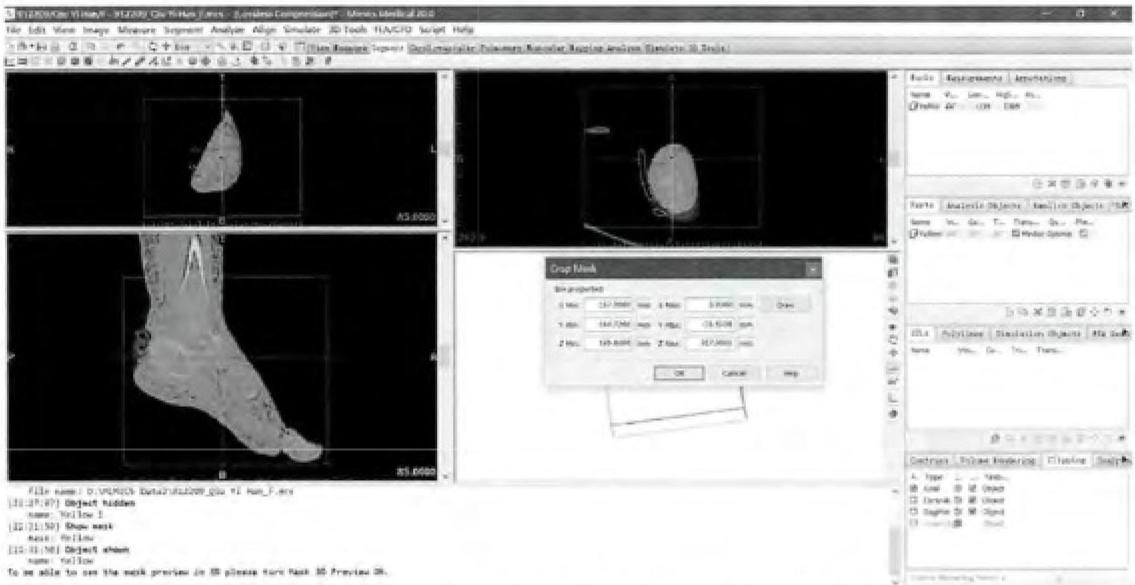

（2）在 Mask 中点击 Calculate Part from Mask，可在 Mask 下方的 Parts 生成患肢的三维图像。

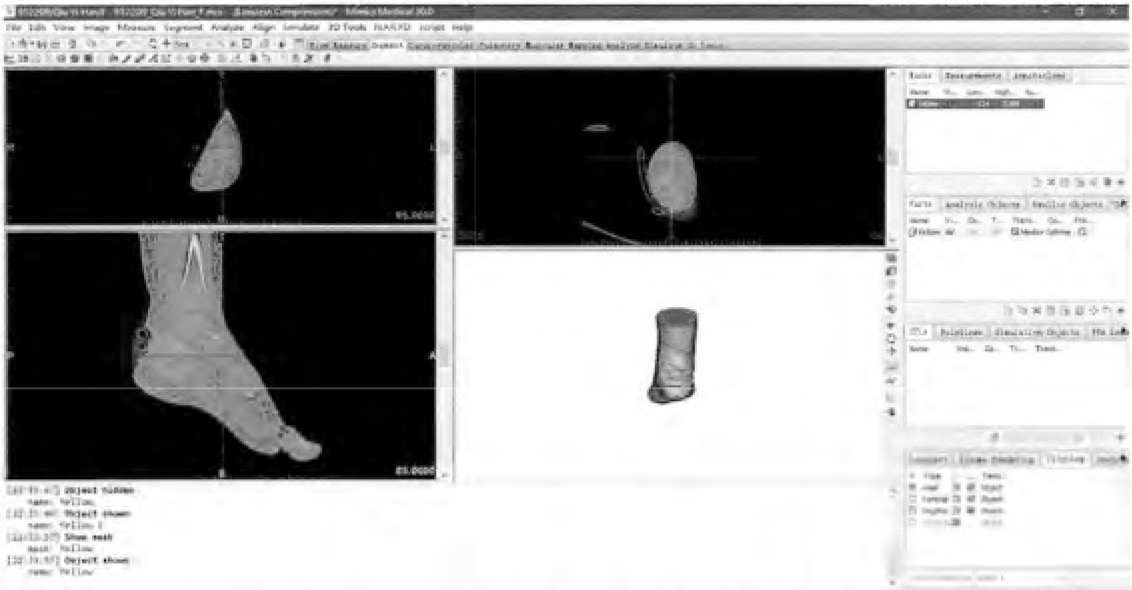

（3）在 Parts 中鼠标右键点击已生成的三维图像，点击 Copy，打开 3-matic Medical 12.0，在 Work Area 中按 Ctrl + V，即可显现 Mimics Medical 20.0 创建的患肢的三维图像。

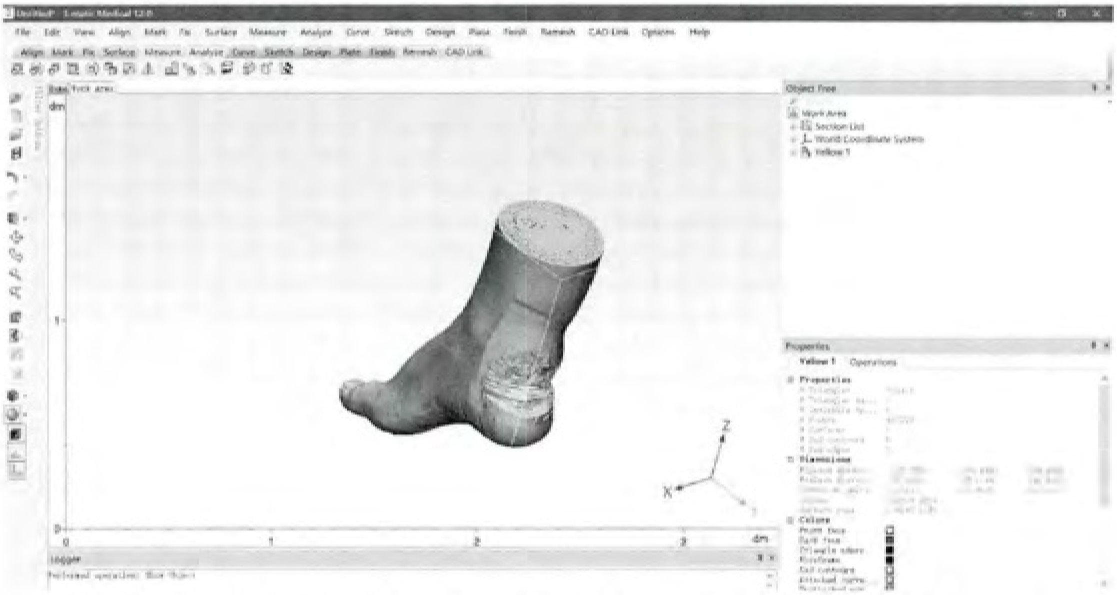

（4）鼠标左键点击键面上方主工具栏中 Mark，再点击分组工具栏中 Lasso Area Mark，填补缺损区域，在填充过程中不断点击 Expand，直到完全填补缺损。注意在填补过程中不要有遗漏。

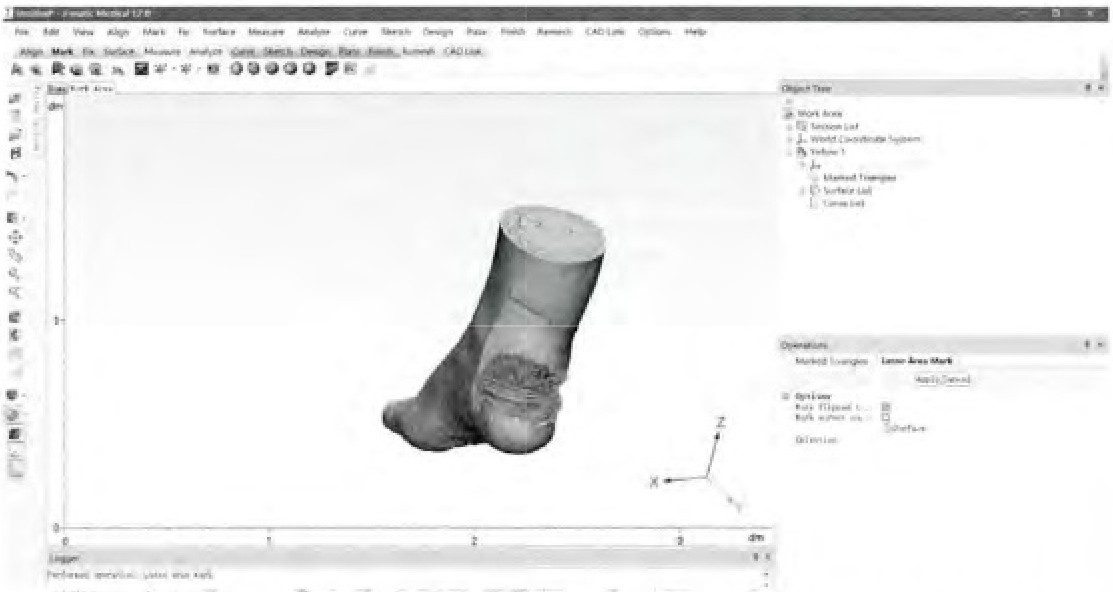

（5）填充成功后，鼠标左键点击键面主工具栏 Mark 中 Smooth Marking Border，即可生成软组织缺损区轮廓。

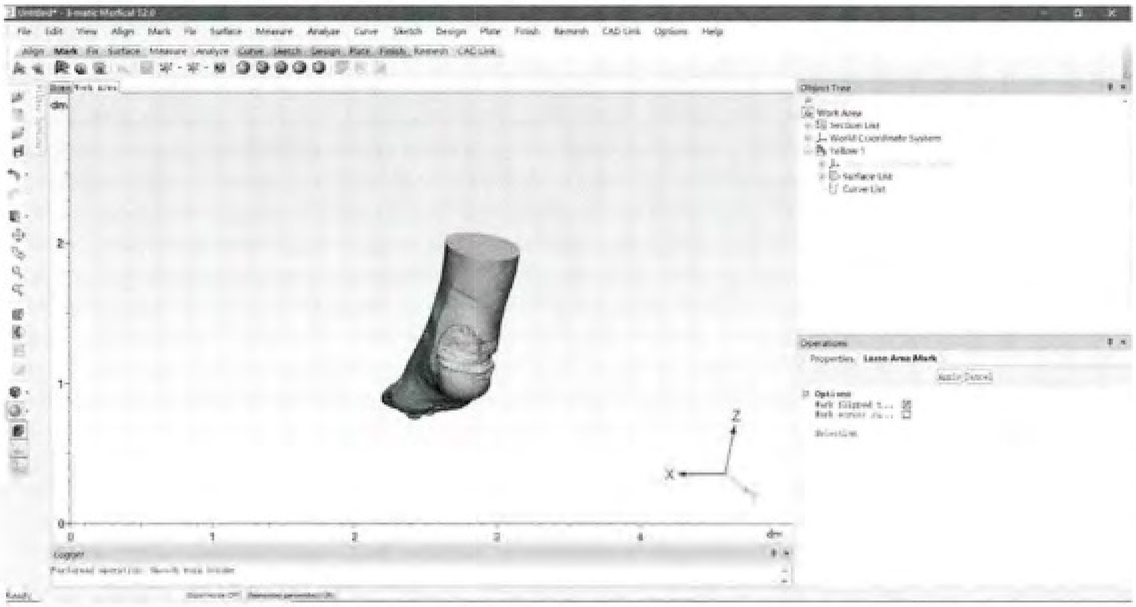

（6）鼠标左键点击缺损区轮廓，并按住 Shift 键，可弹出 Surface，点击 Surface，在右上方项目管理器中出现 Surface，鼠标右键点击 Surface，分别选择 Separate-Copy to Part-Create Part，可另外单独生成软组织缺损区域。鼠标左键点击左上方的 Surface，在右下方可分别自动显示缺损区域长度、宽度和表面积。

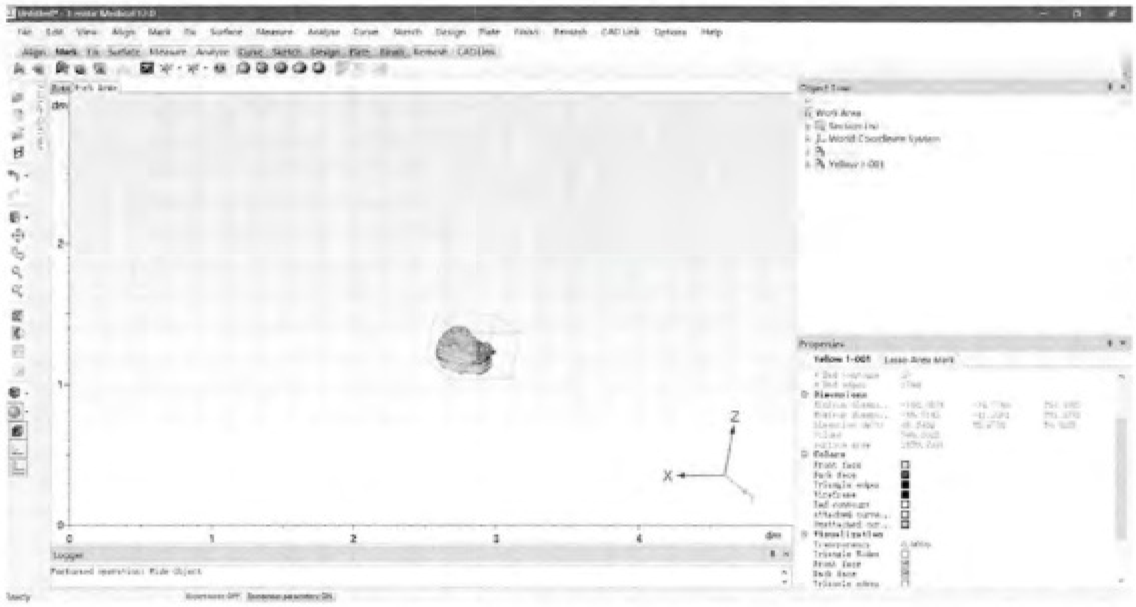

（7）利用软件自带的测量工具，测量供区皮瓣的厚度。

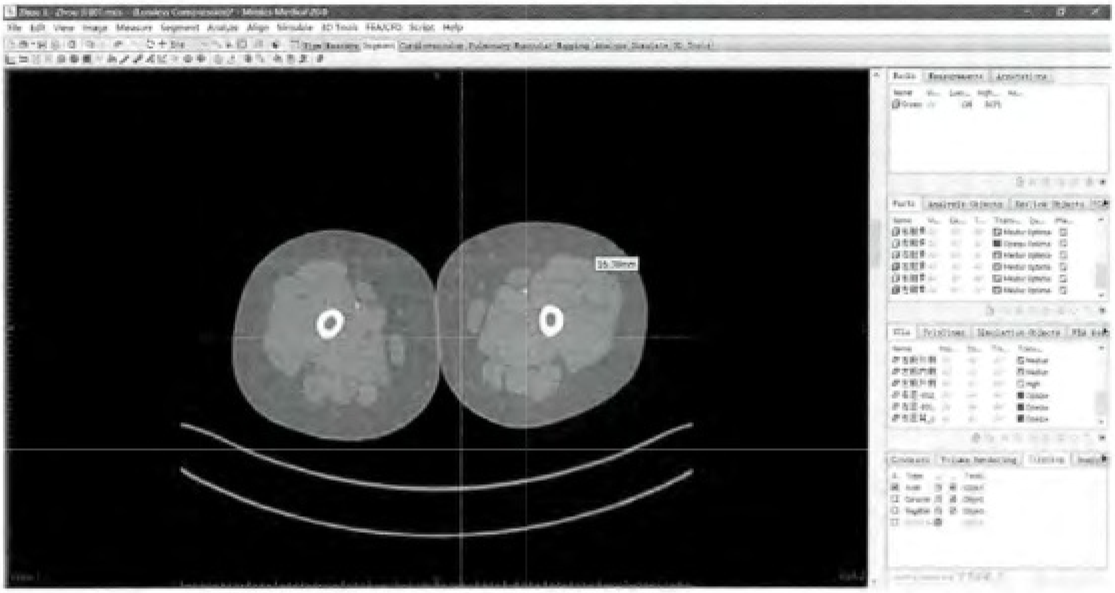

（8）点击键面左上方主工具栏中 Design，再点击 Local Offset，右下方弹出对话框，将 Offset 参数中 Offset distance 更改为供区的厚度，点击 Apply，可生成具有厚度的缺损区域。此时鼠标左键再次点击左上方的 Surface，在右下方可分别自动显示缺损区域长度、宽度和表面积。

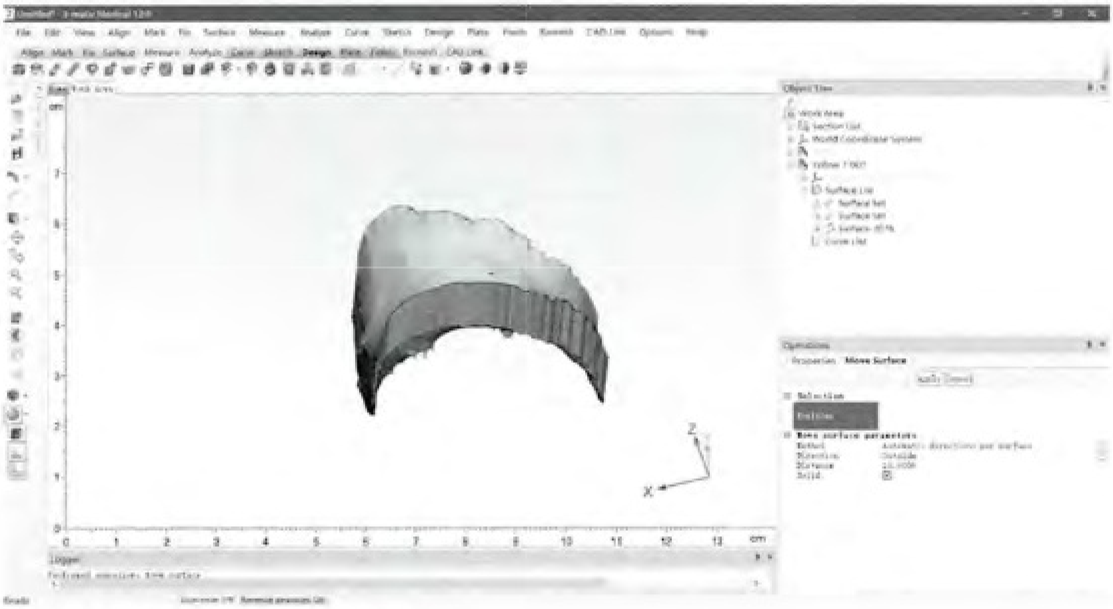

2.3 供区肌肉三维图像重建

（1）先建立一个新的蒙版（New Mask），建立成功后点击 Clear Mask，在水平面图像中找到肌肉的起止点。

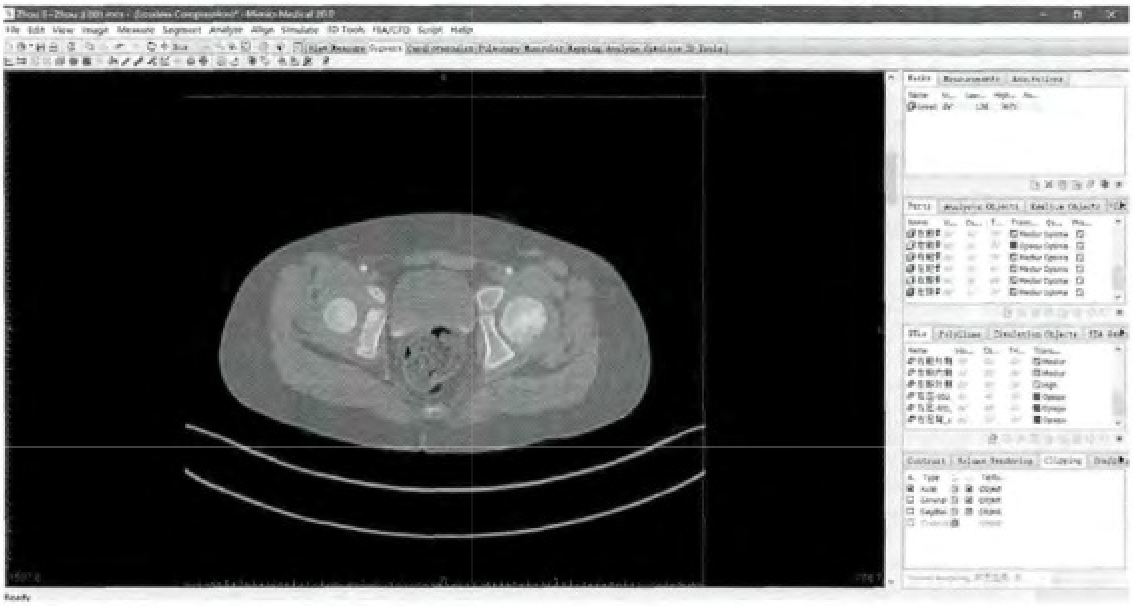

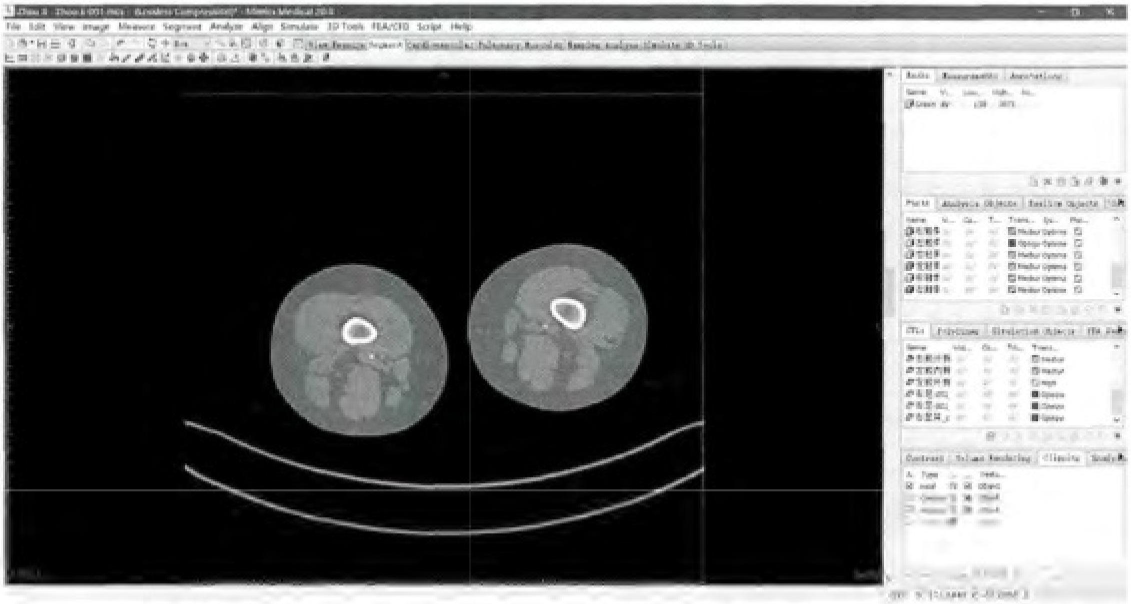

（2）找到肌肉起点，点击 Multiple Slice Edit,选择 Live Wire，可自行标记肌肉边界，一直标记到肌肉止点，所有层面均标记后点击 Interpolate，再点击 Apply，即可生成水平面图像上所有层面的肌肉图像。如果在标记过程中出现错误，可按 Ctrl + Z 进行修改。在 Mask 中点击 Calculate Part from Mask，可在 Mask 下方的 Parts 生成三维图像。在 Parts 中鼠标右键点击已生成的三维图像，点击 Copy。打开 3-matic Medical 12.0，在 Work Area 中按 Ctrl + V，即可显现 Mimics Medical 20.0 创建的肌肉三维图像。在窗口上方的功能区中，点击 Fix，选择 Reduce，将右下方 Reduce 参数中 Geometrical error 更改为 0.4000，点击 Apply；再点击 Fix，选中 Smooth，将右下方

Smooth 参数中 Smooth factor 更改为 0.8000，点击 Apply,即可生成表面比较光滑的肌肉。

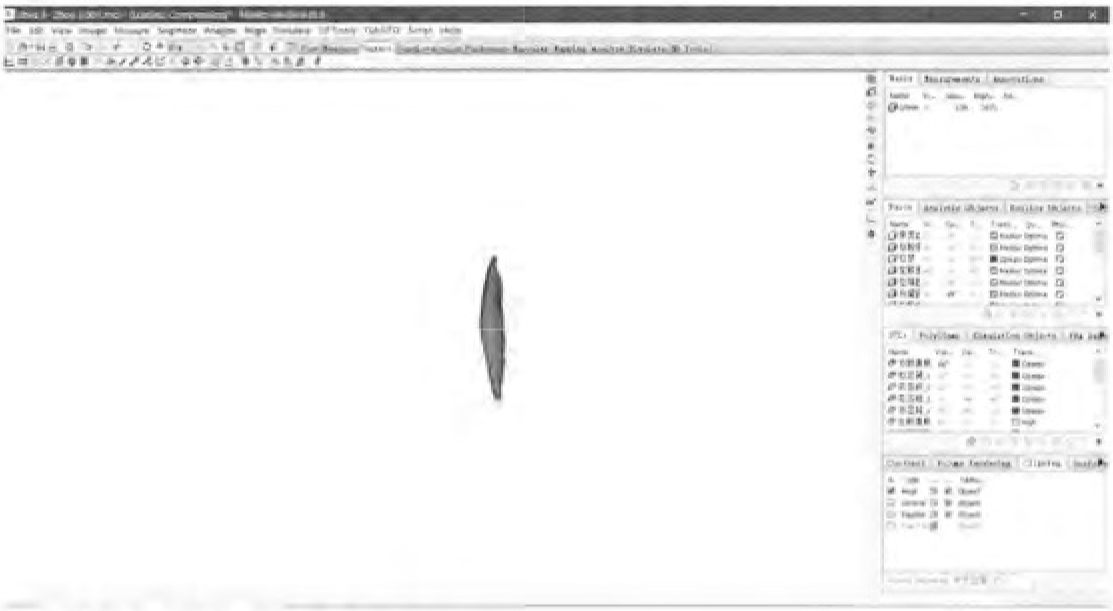

2.3 供区肌肉三维图像重建

（1）在 Mimics 软件操作界面上，开始阈值分割，调整像素灰度值，选择血管显示的阈值，即可生成血管三维图像。

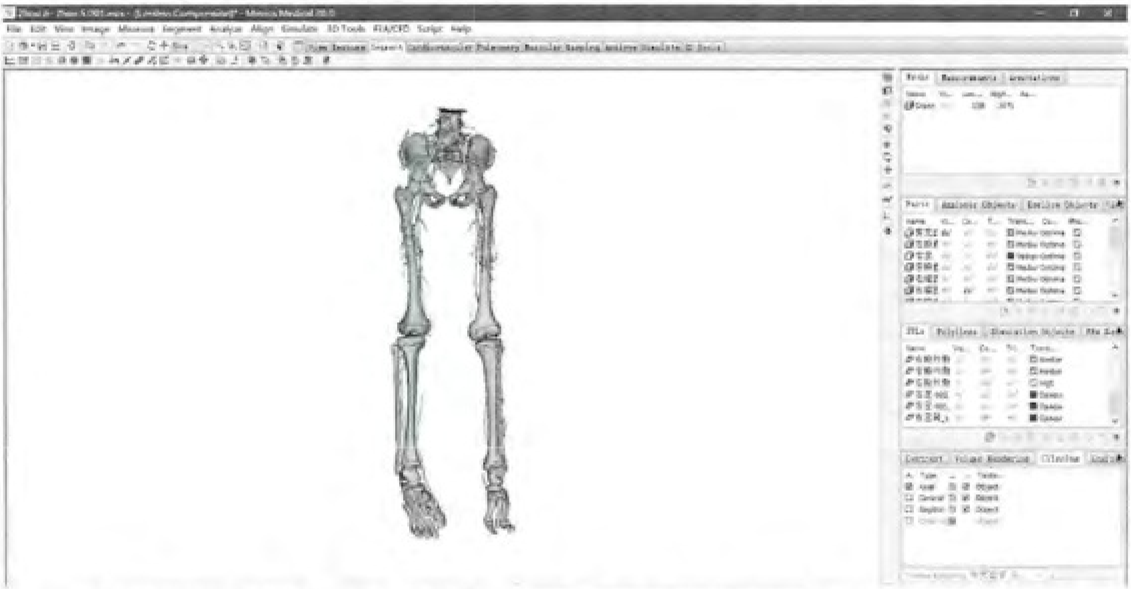

（2）利用区域增长（Region Growing）及裁剪蒙版（Crop Mask）将所需穿支血管单独分离出来，并将肌肉虚拟化，显示穿支血管在肌肉中的走行。

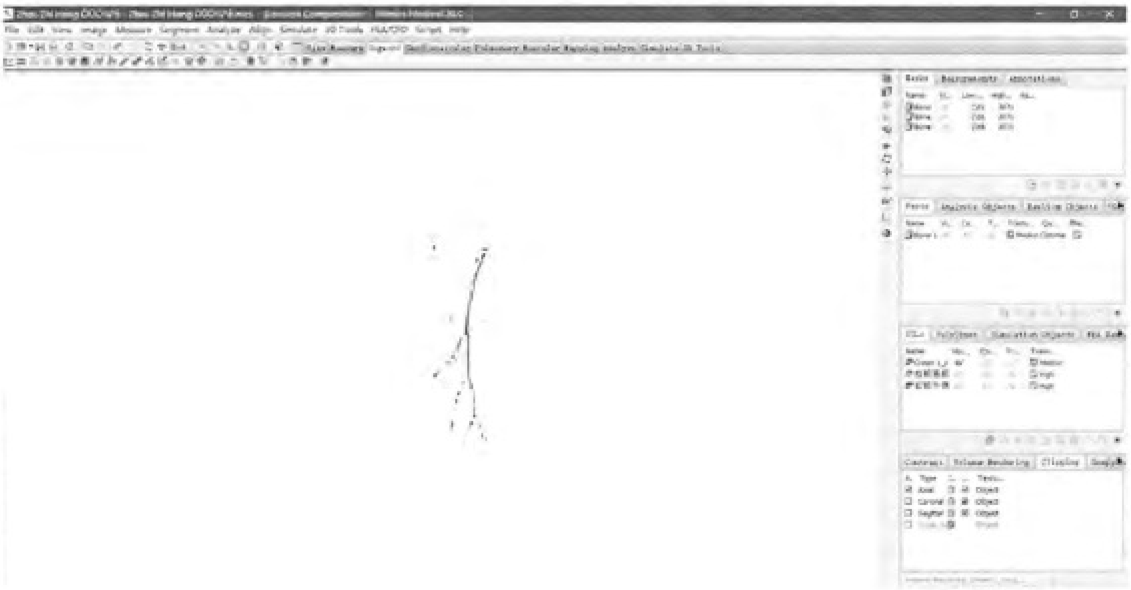

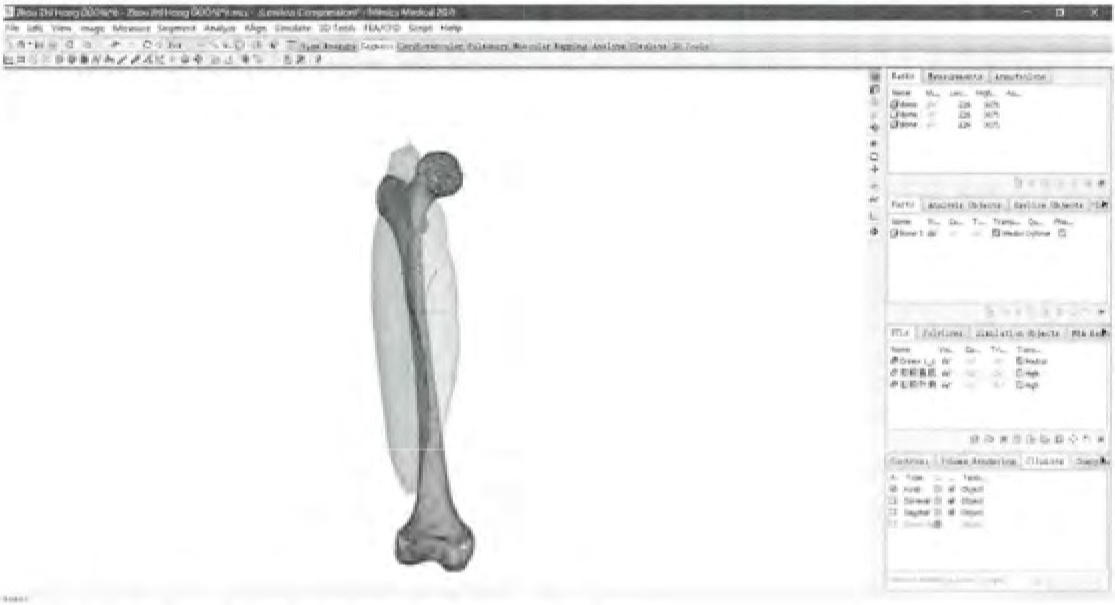

3、皮瓣厚度与皮瓣表面积的测量与计算

3.1 皮瓣厚度的测量

（1）在 Mimics 操作界面中，选取切取皮瓣部分，选择测量工具，测量皮瓣的平均厚度。

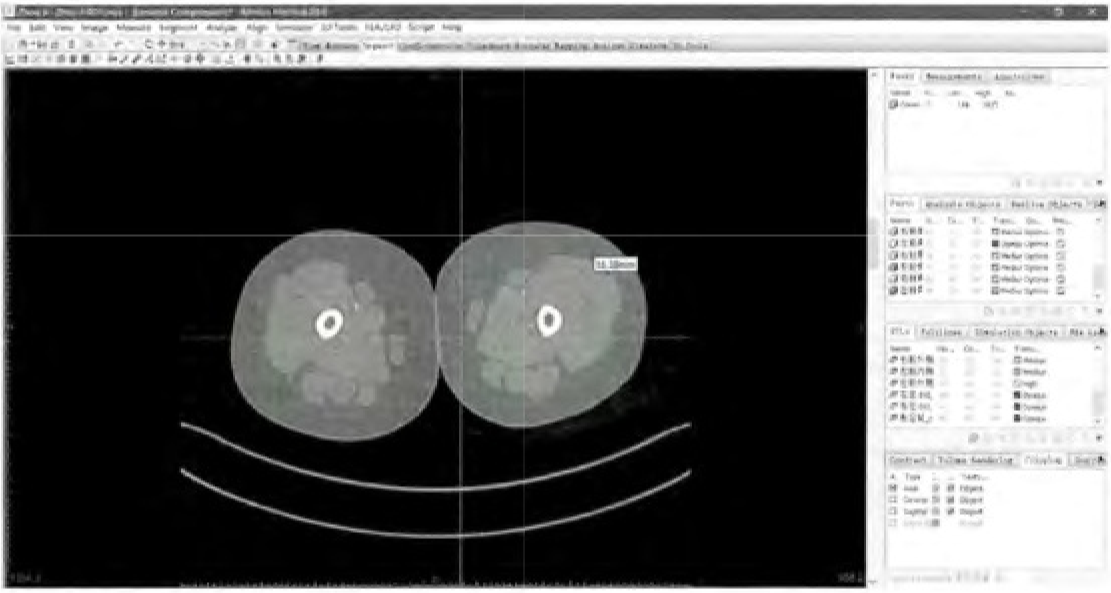

3.3 皮瓣表面积的计算

鼠标左键选择皮瓣表面，3-matic 自带的表面积计算功能将生成皮瓣的表面积。

4、按照上述方法完成 40 例皮瓣厚度以及皮瓣表面积的测量与计算，统计数据。

5、统计学分析

采用 SPSS 24.0（SPSS，Chicago，IL，USA）统计软件进行数据处理分析，分析前所有数据均进行方差齐性检验，皮瓣长度、宽度和表面积采用均数±标准差（ $\bar{x}\pm s$ ）表示，$P<0.05$ 表示差异具有统计学意义；Mimics 三维重建和术中发现的穿支血管走行，采用 kappa 值评估一致性，kappa 值=1 表示两组完全一致，kappa 值=0 表示偶然一致。

二、实验结果

（一）平面组

1、皮瓣长度

当受区创面近似平面图形时，20 例皮瓣术前传统布样设计长度为 17.8±8.1cm，Mimics 设计长度为 17.9±8.0cm。采用 SPSS24.0 进行统计学分析，传统布样设计的皮瓣长度与 Mimics 设计的皮瓣长度之间无统计学差异（P=0.09，P>0.05）具体数据如表 1。

表 1：传统布样设计与 Mimics 设计的皮瓣长度数据（平面组）

患者编号	布样设计（cm）	Mimics 设计（cm）	两者差值（cm）	厚度（cm）
1	16.0	16.0	0.0	0.3
2	10.0	10.0	0.0	0.4
3	13.0	13.0	0.0	0.5
4	16.0	16.0	0.0	0.6
5	30.0	30.0	0.0	0.7
6	15.0	15.0	0.0	0.8
7	15.0	15.0	0.0	0.8
8	31.0	31.0	0.0	0.8
9	13.0	13.0	0.0	0.8
10	30.0	30.0	0.0	1.0
11	7.0	7.0	0.0	1.2
12	18.0	18.2	0.2	1.2
13	25.0	25.3	0.3	1.2
14	28.0	28.0	0.0	1.2
15	25.0	25.4	0.4	1.3
16	10.0	10.0	0.0	1.4
17	12.0	12.0	0.0	1.5
18	14.0	14.2	0.2	1.6
19	14.0	14.0	0.0	1.6
20	10.0	10.5	0.5	1.8

2、皮瓣宽度

当受区创面近似平面图形时，20 例皮瓣术前传统布样设计宽度为 6.6±1.6cm，Mimics 设计宽度为 6.7±1.6cm。采用 SPSS24.0 进行统计学分析，传统布样设计的皮瓣宽度与 Mimics 设计的皮瓣宽度之间无统计学差异（P=0.056，P>0.05）具体数据如表 2。

表 2：传统布样设计与 Mimics 设计的皮瓣宽度数据（平面组）

患者编号	布样设计（cm）	Mimics 设计（cm）	两者差值（cm）	厚度（cm）
1	9.0	9.0	0.0	0.3
2	7.5	7.5	0.0	0.4
3	5.0	5.0	0.0	0.5
4	8.0	8.0	0.0	0.6
5	6.0	6.0	0.0	0.7
6	5.0	5.0	0.0	0.8
7	7.0	7.0	0.0	0.8
8	11.0	11.0	0.0	0.8
9	7.0	7.0	0.0	0.8
10	6.0	6.0	0.0	1.0
11	4.5	4.5	0.0	1.2
12	6.0	6.1	0.1	1.2
13	7.0	7.2	0.2	1.2
14	8.0	8.2	0.2	1.2
15	6.0	6.1	0.1	1.3
16	5.0	5.2	0.2	1.4
17	6.5	6.5	0.0	1.5
18	5.0	5.0	0.0	1.6
19	7.0	7.1	0.1	1.6
20	5.0	5.0	0.0	1.8

3、皮瓣厚度

当受区创面近似平面图形时，20 例皮瓣厚度为 1.0±0.4cm。随着皮瓣厚度逐渐增加，传统布样设计与 Mimics 设计的皮瓣长度、宽度及表面积差值无明显增加或减小。采用 SPSS24.0 进行统计学分析，如图 1 所示，传统布样设计与 Mimics 设计的皮瓣长度差值与厚度之间无相关性，差异无统计学意义（Pearson 相关系数 r=0.442，P=0.051，P>0.05）；如图 2 所示，宽度差值与厚度之间无相关性，差异无统计学意义（Pearson 相关系数 r=0.285，P=0.223，P>0.05）；如图 3 所示，表面积差值与厚度之间无相关性，差异无统计学意义（Pearson 相关系数 r=0.442，P=0.051，P>0.05）。

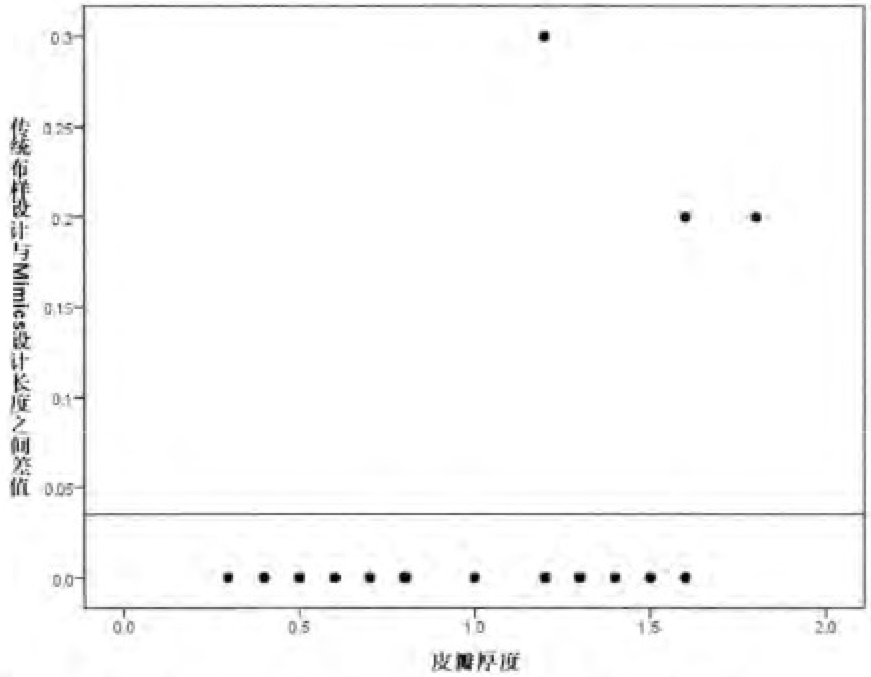

图 1：传统布样设计与 Mimics 设计的皮瓣长度差值与厚度之间相关性

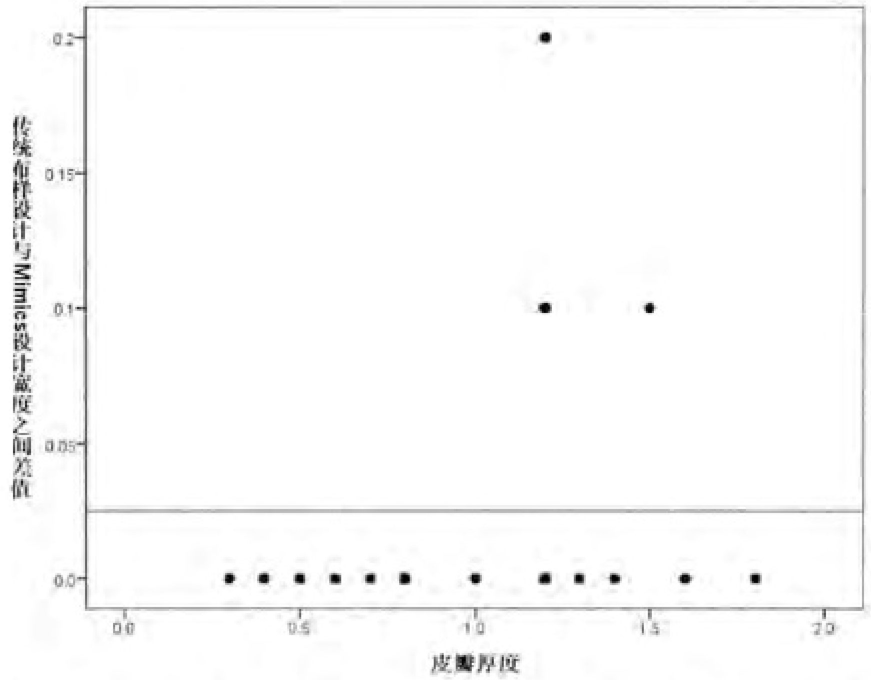

图 2：传统布样设计与 Mimics 设计的皮瓣宽度差值与厚度之间相关性

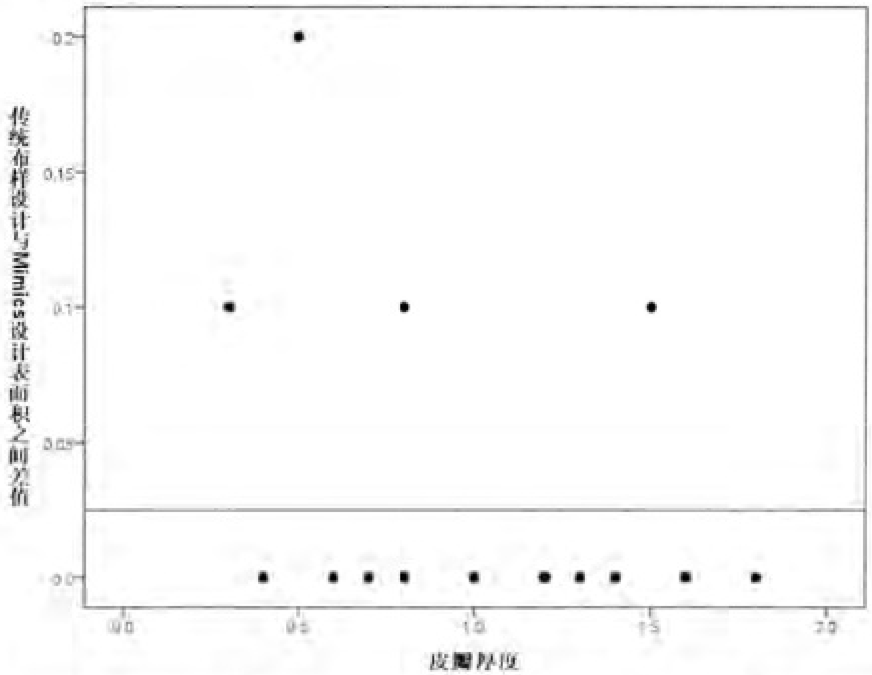

图 3：传统布样设计与 Mimics 设计的皮瓣表面积差值与厚度之间相关性

4、皮瓣表面积

当受区创面近似平面图形时，20 例皮瓣术前传统布样设计表面积为 64.8±35.6cm^2，Mimics 设计表面积为 64.9±35.6cm^2。采用 SPSS24.0 进行统计学分析，传统布样设计的皮瓣表面积与 Mimics 设计的皮瓣表面积之间无统计学差异（P=0.056，P>0.05）具体数据如表 3。

表 3：传统布样设计与 Mimics 设计的皮瓣表面积数据（平面组）

患者编号	布样设计（cm^2）	Mimics 设计（cm^2）	两者差值（cm^2）	厚度（cm）
1	74.1	74.1	0.0	0.3
2	38.7	38.7	0.0	0.4
3	38.6	38.6	0.0	0.5
4	53.9	53.9	0.0	0.6
5	116.8	116.8	0.0	0.7
6	49.3	49.3	0.0	0.8
7	63.2	63.2	0.0	0.8
8	165.1	165.1	0.0	0.8
9	50.8	50.8	0.0	0.8
10	98.3	98.3	0.0	1.0
11	21.8	21.8	0.0	1.2
12	59.2	59.7	0.5	1.2
13	83.5	83.8	0.3	1.2
14	112.1	112.5	0.4	1.2
15	73.2	73.8	0.6	1.3
16	28.1	28.5	0.4	1.4
17	48.3	48.3	0.0	1.5
18	37.2	37.2	0.0	1.6
19	52.3	52.7	0.4	1.6
20	32.3	32.3	0.0	1.8

（二）弧度组

1、皮瓣长度

20 例皮瓣术前传统布样设计长度为 17.9±7.9cm，Mimics 设计长度为 19.4±7.7cm。采用 SPSS24.0 进行统计学分析，传统布样设计的皮瓣长度与 Mimics 设计的皮瓣长度之间无统计学差异（P=0.000，P<0.05）具体数据如表 4。

表 4：传统布样设计与 Mimics 设计的皮瓣长度数据（弧度组）

患者编号	布样设计（cm）	Mimics 设计(cm)	两者差值（cm）	厚度（cm）
1	17.5	18.0	0.5	0.6
2	9.0	10.0	1.0	0.6
3	24.5	25.0	0.5	0.8
4	17.5	18.0	0.5	0.8
5	19.0	20.0	1.0	0.8
6	31.0	32.0	1.0	0.9
7	29.0	30.0	1.0	0.9
8	24.0	26.0	2.0	1.1
9	27.0	28.0	1.0	1.2
10	15.5	17.0	1.5	1.2
11	9.0	11.0	2.0	1.4
12	29.0	31.0	2.0	1.5
13	6.0	7.0	1.0	1.5
14	22.5	24.0	1.5	1.5
15	18.5	21.0	2.5	1.5
16	17.0	19.0	2.0	1.6
17	13.0	15.0	2.0	1.6
18	10.5	13.0	2.5	1.6
19	13.0	16.0	3.0	1.8
20	6.0	8.0	2.0	1.8

2、皮瓣宽度

20 例皮瓣术前传统布样设计宽度为 5.5±1.7cm，Mimics 设计宽度为 6.3±2.0cm。采用 SPSS24.0 进行统计学分析，传统布样设计的皮瓣宽度与 Mimics 设计的皮瓣宽度之间无统计学差异（P=0.001，P<0.05）具体数据如表 5。

表 5：传统布样设计与 Mimics 设计的皮瓣宽度数据（弧度组）

患者编号	布样设计（cm）	Mimics 设计（cm）	两者差值（cm）	厚度（cm）
1	7.0	7.0	0.0	0.6
2	5.0	5.0	0.0	0.6
3	5.0	5.0	0.0	0.8
4	5.0	5.0	0.0	0.8
5	7.0	7.0	0.0	0.8
6	5.5	6.0	0.5	0.9
7	8.0	9.0	1.0	0.9
8	5.5	6.0	0.5	1.1
9	6.0	7.0	1.0	1.2
10	6.0	7.0	1.0	1.2
11	5.5	8.0	2.5	1.4
12	5.0	7.0	2.0	1.5
13	3.0	3.0	0.0	1.5
14	4.0	6.0	2.0	1.5
15	4.0	5.0	1.0	1.5
16	10.0	12.0	2.0	1.4
17	3.0	3.0	0.0	1.6
18	5.5	7.0	1.5	1.6
19	5.0	6.0	1.0	1.8
20	4.0	5.0	1.0	1.8

3、皮瓣厚度

当受区创面近似平面图形时，20 例皮瓣厚度为 1.2±0.4cm。随着皮瓣厚度逐渐增加，传统布样设计与 Mimics 设计的皮瓣长度、宽度及表面积差值有明显改变。采用 SPSS24.0 进行统计学分析，如图 4 所示，传统布样设计与 Mimics 设计的皮瓣长度差值与厚度之间有相关性，差异具有明显统计学意义（Pearson 相关系数 r=0.823，P=0.000，P<0.05）；如图 5 所示，宽度差值与厚度之间有相关性，差异具有明显统计学意义（Pearson 相关系数 r=0.561，P=0.01，P<0.05）；如图 6 所示，表面积差值与厚度之间无相关性，差异无统计学意义（Pearson 相关系数 r=0.528，P=0.017，P<0.05）。

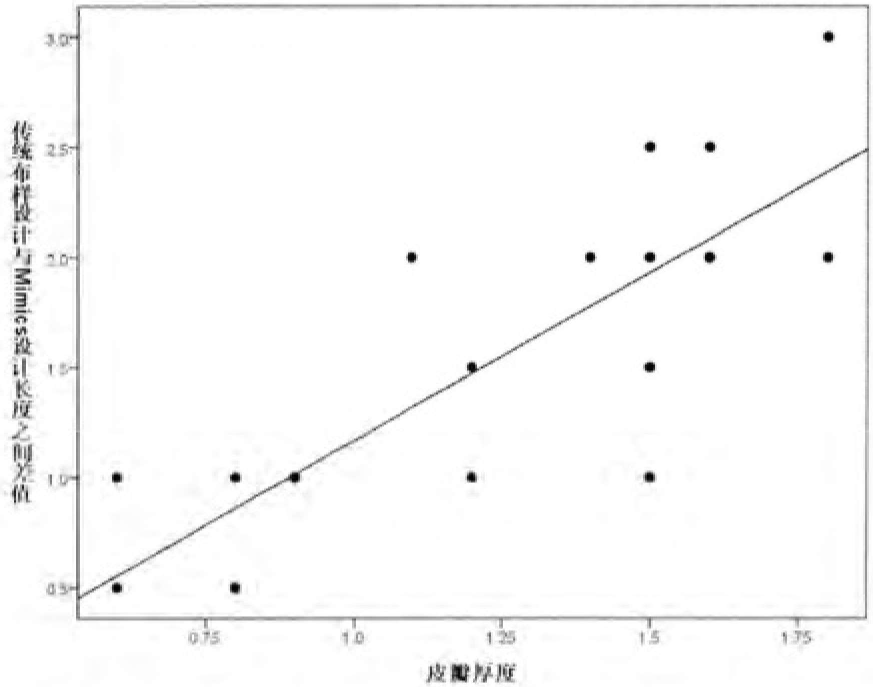

图 4：传统布样设计与 Mimics 设计的皮瓣长度差值与厚度之间相关性

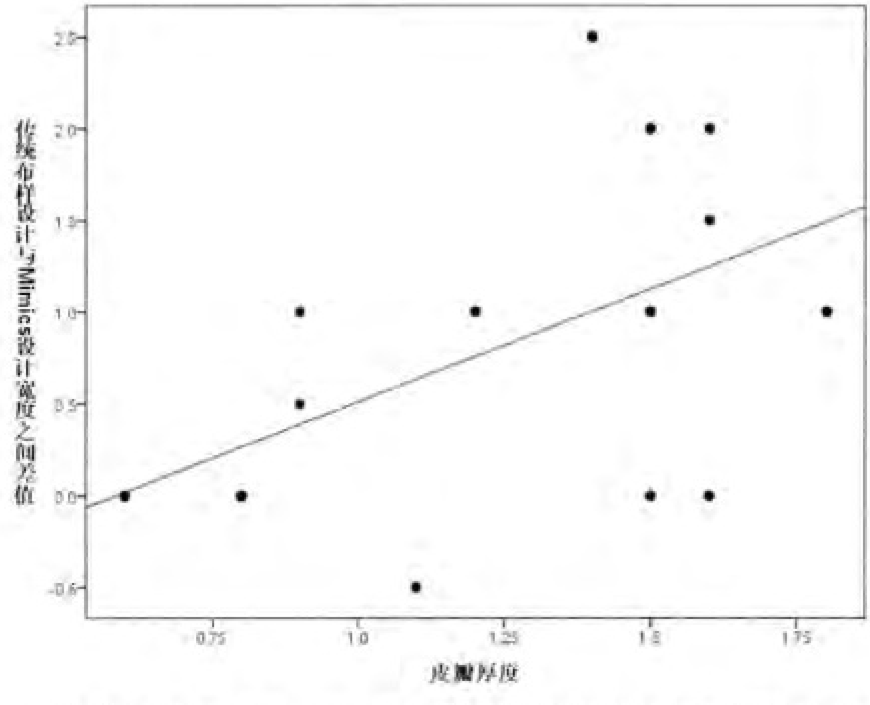

图 5：传统布样设计与 Mimics 设计的皮瓣宽度差值与厚度之间相关性

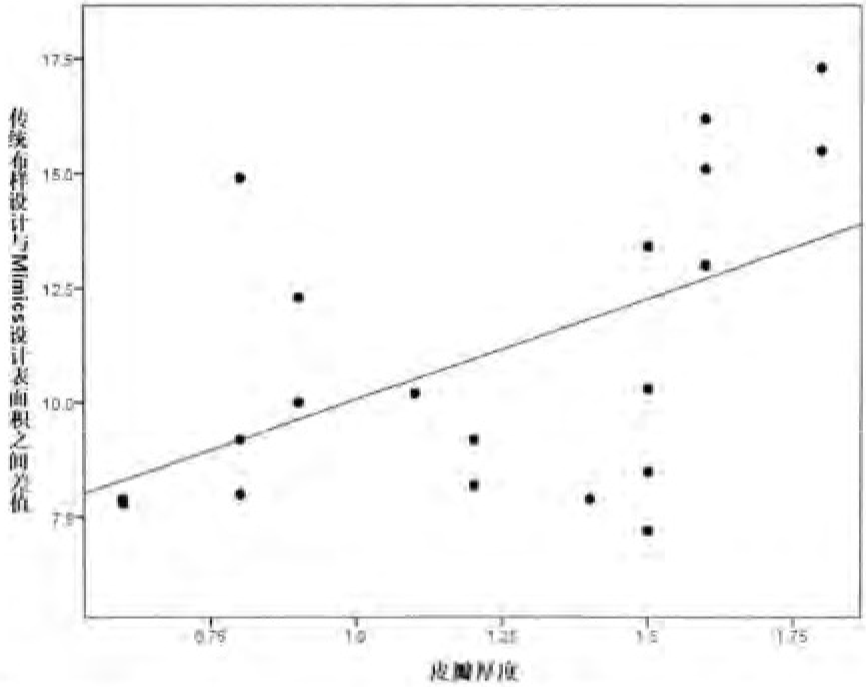

图 6：传统布样设计与 Mimics 设计的皮瓣表面积差值与厚度之间相关性

4、皮瓣表面积

20 例皮瓣术前传统布样设计表面积为 68.2±44.6cm^2，Mimics 设计表面积为 79.3±44.2cm^2。采用 SPSS24.0 进行统计学分析，传统布样设计的皮瓣表面积与 Mimics 设计的皮瓣表面积之间无统计学差异（P=0.000，P<0.05）具体数据如表 6。

表 6：传统布样设计与 Mimics 设计的皮瓣表面积数据（弧度组）

患者编号	布样设计（cm^2）	Mimics 设计（cm^2）	两者差值（cm^2）	厚度（cm）
1	77.3	85.1	7.8	0.6
2	22.8	30.8	8.0	0.6
3	76.9	85.1	8.2	0.8
4	54.8	62.7	7.9	0.8
5	83.2	91.7	8.5	0.8
6	109.8	119.0	9.2	0.9
7	159.3	168.5	9.2	0.9
8	61.9	72.1	10.2	1.1
9	117.2	127.2	10.0	1.2
10	58.1	68.4	10.3	1.2
11	43.1	56.5	13.4	1.4
12	138.4	150.7	12.3	1.5
13	8.1	15.3	7.2	1.5
14	41.2	54.2	13.0	1.5
15	37.8	52.9	15.1	1.5
16	143.1	158.0	14.9	1.6
17	17.4	33.6	16.2	1.6
18	39.7	57.0	17.3	1.6
19	54.8	69.8	15.0	1.8
20	18.1	33.6	15.5	1.8

（三）穿支血管走行类型

40 例皮瓣，术前三维重建发现 76 支穿支血管，包括 58 支肌皮穿支血管，18 支肌间隙穿支血管；术中发现 76 支穿支血管，包括 58 支肌皮穿支血管，18 支肌间隙穿支血管。术前三维重建穿支血管走行与术中发现结果进行 kappa 值一致性检验，kappa 值=1，表明三维重建定位穿支走行与术中探查相一致。

表 7：Mimics 三维重建与术中发现穿支血管类型对比

Mimics 三维重建	术中发现		合计
	肌皮穿支血管	肌间隙穿支血管	
肌皮穿支血管	58	0	58
肌间隙穿支血管	0	18	18
合计	58	18	76

三、讨 论

高能量损伤所致的四肢软组织缺损越来越多见，穿支皮瓣移植是目前主要的治疗手段。精准的皮瓣修复主要依赖于术前完美的形状设计与准确的穿支定位技术。传统布样是目前临床上主要采用的形状设计方法，但目前仍停留在手工测量和目测估计上，具有较大的随意性、盲目性和风险性，缺乏科学性和规范性，并且极易受到临床医师主观因素的影响。同时布样也无法考虑供区皮瓣的厚度与受区创面的弧度，术后常出现供受区不匹配现象，增加了手术次数、住院时间和费用，延缓患者功能康复。唐举玉等提出皮瓣不削薄则需要切取更大面积方能覆盖同样大小的创面。目前随着超薄皮瓣理念的出现，显微削薄技术已逐渐运用于肥胖患者皮瓣手术中，即通过显微外科技术一期修薄皮瓣，使其尽可能与受区创面匹配。但在某些情况下，即使将皮瓣削薄，由于未考虑受区创面弧度的存在，术后依然会影响供受区匹配。另外，唐举玉等未阐述皮瓣厚度对皮瓣设计的影响机理。并且糜菁熠等认为，尽管皮瓣一期削薄可明显改善外观，但该项术式增加了手术时间和对皮瓣本身的创伤，可能导致术后皮瓣肿胀而影响影响皮瓣血运，术前设计皮瓣时长宽仍要适当放大，增加了对供区的损害。

在穿支定位技术方面，随着医学影像技术的飞速发展，目前显微外科定位技术主要有 CDS 和 CTA。虽然多项研究表明，CDS 穿支定位检测阳性率超过 90%，穿支走行准确率可达 100%，但是 CDS 对操作者依赖性较高，主观性较强，也无法为术者提供直接、易懂的影像参考信息。CTA 作为目前穿支皮瓣术前定位的金标准，然而有研究表明，利用 CT 扫描仪自带的软件所获得的图像不仅层次及毗邻结构显示不清，无法为术者提供血管与周围组织的关系，而且难以三维观察穿支血管在肌肉中走行，缺乏指导意义。最重要的是，CDS 和 CTA 只能输出二维、平面和静态的定位图像，难以三维观察穿支血管在肌肉中走行。因此对于难以想象穿支血管三维走行的临床医师来说，术中可能会盲目分离供区血管，破坏皮瓣血运，导致手术失败。

截止到目前为止，根据本人所能查阅到的所有文献，皮瓣设计仍主要集中在外观形状以及穿支血管定位中，并没有供区皮瓣厚度与受区创面弧度对皮瓣大小设计的影响以及穿支血管在肌肉中三维走行的研究报道。

一、Mimics 联合 CTA 在穿支皮瓣术前精准设计研究中的意义

我们生活在一个数字化时代，数字医学正在影响临床医生的日常工作，它的兴起发展正改变传统的医疗模式[40]。数字化技术是数字医学中重要的研究工具和方法。目前在骨外科、整形外科、显微外科、颌面外科、影像科等，三维重建、分割综合、虚拟仿真及导航技术等数字化技术已取得了很好的临床应用效益。其中三维重建技术目前已经广泛的应用于临床，而进行三维重建研究的软件种类繁多，在医疗方面应用最广泛的就是 Mimics 软件。Mimics 软件是一种基于 CT 扫描数据的三维图像处理和编辑工具，可以利用动态三维图像对传统二维医学图像进行补充。它能将 CT、CTA 扫描获得的连续断层图像进行三维重建，可以精确地显示生物组织复杂的三维结构，并可进行任意旋转观察。另外，Mimics 利用自带的测量功能，可以精确测量长度、宽度、表面积、体积和角度等大量精确的解剖学参数，用于临床辅助诊断、辅助手术设计和手术模拟等。

Mimics 是作为一种新兴的三维图像处理和编辑软件，已被广泛应用于血管、皮瓣等器官、结构的研究中。CTA 是虽然目前临床穿支皮瓣血管定位的金标准，但是，利用 CT 自带的软件所获得的图像不仅层次及毗邻结构显示不清，很难对血管树细小的分支血管清晰展示。Zhang 等[41]在应用放射造影术进行穿支皮瓣研究过程中发现：将 CTA 扫描后获得的图像数据导入 Mimics 软件中，可轻松地对小血管进行定位。Li 等[34]研究发现，与传统的方式相比，Mimics 联合 CTA 三维重建腓肠神经营养皮瓣指导修复足踝部皮肤软组织缺损，明显缩短手术时间，提高皮瓣成活率。还有研究者运用数字化技术导航皮瓣移植重建乳房获得更加美观的外形。我们研究发现，Mimics 联合 CTA 三维重建骨骼、肌肉以及穿支血管，可明显辨别穿支血管与肌肉组织的结构关系，为术者分离血管提供依据。

Mimics 联合 CTA 三维建模具有以下优势：①Mimics 软件可以直接识别 DICOM 格式的 CT、CTA 及 MRI 扫描数据，并以 STL 格式输出，必要时可进行 3D 打印。②Mimics 软件可以自动建模，重建的结构具有直观、准确和可重复性。③对于原始图像进行预处理时不需要额外进行任何形式的图像转换，避免了人为因素造成的误差，同时也在很大程度上减少了数据信息的丢失。④建模速度快。Mimics 软件可以自动识别、读取 CT 和 CTA 导出的 DICOM 格式的原始扫描图像。⑤三维重建可以动

态、立体、多角度显示血管、肌肉与骨骼三维空间解剖关系，制定个性化手术方案。⑥术前能够准确的了解皮瓣的位置、厚度、大小、几何形状以及创面弧度，能够清晰显示穿支血管的走行及变异情况。避免因血管的变异而导致皮瓣的切取失败，有助于缩短手术时间，减少手术盲目性，提高手术效果。⑦可在个人电脑上模拟手术的关键步骤，实现术者的构思和手术经验，为手术组每位成员所共享。

二、皮瓣大小设计的影响因素

根据测量结果来看，当受区创面接近平面时，采用传统布样设计皮瓣的长度、宽度、表面积与 Mimics 设计的皮瓣长度、宽度、表面积在数值上无明显变化，通过 SPSS24.0 软件进行 t 检验分析后发现两种设计方法之间不存在统计学差异，且传统布样设计与 Mimics 设计的皮瓣长度差值、宽度差值及表面积差值与皮瓣厚度无相关性，差异无统计学差异，表明当受区创面接近平面时，传统布样设计能够实现供受区精准匹配；而当受区创面具有明显弧度时，传统布样设计的皮瓣长度、宽度、表面积与 Mimics 设计的皮瓣长度、宽度、表面积在数值上相比，具有明显改变，采用 SPSS24.0 软件进行 t 检验分析后发现两种设计方法之间存在明显统计学差异，且传统布样设计与 Mimics 设计的皮瓣长度差值、宽度差值及表面积差值与皮瓣厚度具有相关性，差异有统计学差异，说明当受区创面具有明显弧度时，传统布样设计无法实现供受区精准匹配，往往造成供区皮瓣无法完全覆盖受区创面，需二次植皮，增加第二供区损害。唐举玉等研究发现当修复体胖患者的手（腕）、足（踝）、肘与膝关节周围、头面等浅表区域创面时，皮瓣不修薄则需要切取更大面积皮瓣方能覆盖同样大小的创面，该结果与本课题研究皮瓣厚度影响皮瓣设计较为一致，但唐举玉等并未阐述需切取的多余面积数值。因此，我们研究发现，供区创面弧度与皮瓣厚度是影响皮瓣大小设计的两个主要因素。

三、传统布样设计与 Mimics 软件设计的异同

目前皮瓣设计仍停留在传统的布样手工测量及目测估计上，制定手术过程缺乏直观量化的评估标准，手术方案受医生个人经验、习惯等主观思维因素影响较大，并且目测估计不够准确，无法考虑供区皮瓣厚度及受区创面弧度，常常导致供受区不匹配，增加第二供区损害，缺乏科学性、规范性，具有盲目性。而 Mimics 设计改变传统的设计方案，术前能够将皮瓣厚度及受区创面弧度加入皮瓣设计中，不再需要依赖于术

者，实现了供区皮瓣与受区创面的精准匹配。

四、建立穿支血管在肌肉骨骼中走行的临床意义

学者们对穿支皮瓣血管定位进行了大量研究，从最初的手持式超声、CDS，再发展到 CTA、MRA 技术，尽管这些定位检测技术阳性率较高，但依然无法实现二维到三维、平面到立体、静态到动态图像的转变。建立穿支血管在肌肉骨骼中走行的三维可视化模型，目的是穿支进行了体表定位，并完成了皮瓣的个体化设计，为精确手术奠定了基础，降低了因皮瓣血管变异带来的手术风险。对比既往彩色多普勒超声定位及单纯 CTA、MRA 三维重建效果，Mimics 联合 CTA 完成的三维重建主要有以下优点：①能更清晰完整地显示血管与周围肌肉、骨骼的层次关系，真实反映了血管的分布和走行；②可以层次分明地重建主要穿支，显示穿支和皮瓣的关系；③通过 CTA 原始图像明确供区肢体后再行 Mimics 软件三维重建，明显减少了重建工作量；④通过对不同组织进行颜色的添加或调整，使重建图像更立体化和可视化；⑤能够在术前精确化及个体化设计术中所需皮瓣，使手术医师术前就能形象直观地观察所切取皮瓣的形态和特点，并可用软件模拟手术过程，且具有可重复性。三维重建得到的图像轮廓清晰，骨骼、软组织边界清楚。穿支血管及其主要的穿支动脉显影良好，管壁边缘清楚。利用 Mimics 软件重建的穿支血管及其 3D 模型形态逼真、立体感强，各结构可分别着色、透明、独立或组合显示，及 360° 旋转，经过不同角度观察，整体显示清晰，真实反映了穿支血管的分布、走行及穿支在体表皮肤的穿出位置。通过数字化技术在个人计算机上成功地根据创面缺损的形状、大小较精确的设计了分叶穿支皮瓣。通过对皮瓣及血管进行分割、组合，形成以多彩色的形式表现的图像，其形态结构逼真，清晰显示了所重建结构的空间位置关系。可根据血管的走行及其穿支的位置设计皮瓣的切取范围。

五、相关临床意义

对于体胖患者的手（腕）、足（踝）、肘与膝关节周围、颈部等区域软组织缺损，不削薄需要切取更大面积皮瓣方能覆盖同样大小的创面，术前通过 Mimics 联合 CTA 精确计算增加的面积，实现精准覆盖。

六、研究创新性

本课题提出供区皮瓣厚度与受区创面弧度是影响皮瓣设计的两个重要因素；并成功建出穿支血管在肌肉中走行的三维模型，具有一定的创新性，可逐步在临床中推广应用。

七、实验的不足

本课题也存在一些较为局限的地方：研究样本量偏少；供区皮瓣厚度不是完全均匀的，数据的导入导出可能会出现部分信号丢失，需手动弥补，影响结果准确性；创面弧度的测量存在一定的困难；Mimics 软件三维重建效果受到 CTA 原始图像成像效果的影响，另外操作复杂费时，且 Mimics 软件对显影欠佳的穿支分辨率不高，需结合 CTA 原始图片进行半手动提取。该课题只提出了皮瓣厚度和创面弧度将影响皮瓣大小的设计，并没有得出具体的计算公式，故在未来的研究中，需进一步增加样本量，提出皮瓣设计大小的经验性公式，指导临床皮瓣大小设计。

四、结 论

1.当受区创面近似平面图形时，供区皮瓣厚度对皮瓣大小设计无明显影响。

2.当受区创面具有明显弧度时，弧度大小与皮瓣厚度均会影响皮瓣大小设计，且弧度越大，皮瓣越厚，需切取更大面积的皮瓣方能覆盖同样大小的创面。

3.三维穿支血管模型可以准确无误判断其走行，可以为临床分离血管提供依据。

4.三维重建研究技术在修复重建领域中的运用，为临床穿支皮瓣术前精准设计与个性化评估提供有效的理论依据。为术前皮瓣设计的研究提供了一种更为先进、准确且三维立体可视化的研究手段。

参考文献

[1] Hallock GG. Evidence-based medicine: lower extremity acute trauma. Plast Reconstr Surg. 2013. 132(6): 1733-41.

[2] 杨林，刘宏君，张文忠等. 改良股前外侧游离穿支皮瓣桥式交叉移植修复小腿中下段软组织缺损. 中国修复重建外科杂志. 2017. 31(10): 1240-1244.

[3] 侯春林，刘小林. 中国显微外科历史回顾. 中华显微外科杂志. 2015. 38(5): 417-419.

[4] 任义军，胡锐，严立，易新成，韩琮，王俊文. 股前外侧组织瓣修复下肢多处皮肤软组织缺损. 中华显微外科杂志. 2015. 38(5): 447-450.

[5] Zheng X, Zheng C, Wang B, et al. Reconstruction of complex soft-tissue defects in the extremities with chimeric anterolateral thigh perforator flap. Int J Surg. 2016. 26: 25-31.

[6] Maruccia M, Elia R, Caizzi G, et al. Free flap and kickstand external fixator in foot and ankle soft tissue reconstruction. The versatility of a microsurgical-friendly application of an orthopedic device. Injury. 2018. 49 Suppl 3: S105-S109.

[7] Franchi A, Häfeli M, Scaglioni MF, Elliot D, Giesen T. The use of chimeric musculocutaneous posterior interosseous artery flaps for treatment of osteomyelitis and soft tissue defect in hand. Microsurgery. 2019 .

[8] Battiston B, Antonini A, Tos P, Daghino W, Massazza G, Riccio M. Microvascular reconstructions of traumatic-combined tissue loss at foot and ankle level. Microsurgery. 2011. 31(3): 212-7.

[9] Zalavras CG. Prevention of Infection in Open Fractures. Infect Dis Clin North Am. 2017. 31(2): 339-352.

[10] 李海，邓呈亮，魏在荣等. 分叶股前外侧穿支皮瓣在血管蒂保护中的作用研究. 中国修复重建外科杂志. 2017. 31(10): 1245-1249.

[11] Wood T, Sameem M, Avram R, Bhandari M, Petrisor B. A systematic review of early versus delayed wound closure in patients with open fractures requiring flap coverage. J Trauma Acute Care Surg. 2012. 72(4): 1078-85.

[12] 符健松，高顺红，张净宇，张文龙，张云鹏，倪玉龙. 以腓肠内侧血管为受区血

管的游离股前外侧皮瓣移植修复小腿皮肤软组织缺损. 中国修复重建外科杂志. 2015. 29(07): 804-806.

[13] 李海军, 郑晓菊, 张忠, 薛学文. 股前外侧嵌合皮瓣与 Flow-through 修复四肢环形组织缺损. 中华显微外科杂志. 2017. 40(1): 97-100.

[14] 唐举玉, 汪华侨, Hallock GG 等. 关注皮瓣供区问题—减少皮瓣供区损害专家共识. 中华显微外科杂志. 2018. 41(1): 3-5.

[15] Cheng HT, Lin FY, Chang SC. Diagnostic efficacy of color Doppler ultrasonography in preoperative assessment of anterolateral thigh flap cutaneous perforators: an evidence-based review. Plast Reconstr Surg. 2013. 131(3): 471e-3e.

[16] Dorfman D, Pu LL. The value of color duplex imaging for planning and performing a free anterolateral thigh perforator flap. Ann Plast Surg. 2014. 72 Suppl 1: S6-8.

[17] Ensat F, Babl M, Conz C, Fichtl B, Herzog G, Spies M. [Doppler sonography and colour Doppler sonography in the preoperative assessment of anterolateral thigh flap perforators]. Handchir Mikrochir Plast Chir. 2011. 43(2): 71-5.

[18] Ensat F, Babl M, Conz C, et al. The efficacy of color duplex sonography in preoperative assessment of anterolateral thigh flap. Microsurgery. 2012. 32(8): 605-10.

[19] Garvey PB, Selber JC, Madewell JE, Bidaut L, Feng L, Yu P. A prospective study of preoperative computed tomographic angiography for head and neck reconstruction with anterolateral thigh flaps. Plast Reconstr Surg. 2011. 127(4): 1505-14.

[20] Pratt GF, Rozen WM, Chubb D, Ashton MW, Alonso-Burgos A, Whitaker IS. Preoperative imaging for perforator flaps in reconstructive surgery: a systematic review of the evidence for current techniques. Ann Plast Surg. 2012. 69(1): 3-9.

[21] Ribuffo D, Atzeni M, Saba L, Milia A, Guerra M, Mallarini G. Angio computed tomography preoperative evaluation for anterolateral thigh flap harvesting. Ann Plast Surg. 2009. 62(4): 368-71.

[22] Lethaus B, Loberg C, Kloss-Brandstätter A, et al. Color duplex ultrasonography versus handheld Doppler to plan anterior lateral thigh flaps. Microsurgery. 2017. 37(5): 388-393.

[23] Golusiński P, Luczewski Ł, Pazdrowski J, et al. The role of colour duplex sonography in preoperative perforator mapping of the anterolateral thigh flap. Eur

Arch Otorhinolaryngol. 2014. 271(5): 1241-7.

[24] Chung YY, Li SY, Chen MK. Three-dimensional colour Doppler imaging for evaluating perforators of the anterolateral thigh flap. Clin Otolaryngol. 2017. 42(2): 497-499.

[25] 芮永军, 张雁, 杨红等. 术前不同定位方法在股前外侧穿支皮瓣应用的对比分析. 中华显微外科杂志. 2015. 38(1): 33-37.

[26] Chen SY, Lin WC, Deng SC, et al. Assessment of the perforators of anterolateral thigh flaps using 64-section multidetector computed tomographic angiography in head and neck cancer reconstruction. Eur J Surg Oncol. 2010. 36(10): 1004-11.

[27] Zhang Y, Pan X, Yang H, Yang Y, Huang H, Rui Y. Computed Tomography Angiography for the Chimeric Anterolateral Thigh Flap in the Reconstruction of the Upper Extremity. J Reconstr Microsurg. 2017. 33(3): 211-217.

[28] Newman TM, Vasile J, Levine JL, et al. Perforator flap magnetic resonance angiography for reconstructive breast surgery: a review of 25 deep inferior epigastric and gluteal perforator artery flap patients. J Magn Reson Imaging. 2010. 31(5): 1176-84.

[29] Vasile JV, Newman T, Rusch DG, et al. Anatomic imaging of gluteal perforator flaps without ionizing radiation: seeing is believing with magnetic resonance angiography. J Reconstr Microsurg. 2010. 26(1): 45-57.

[30] Vasile JV, Levine JL. Magnetic resonance angiography in perforator flap breast reconstruction. Gland Surg. 2016. 5(2): 197-211.

[31] 唐举玉, 吴攀峰, 俞芳等. 特殊类型穿支皮瓣在创伤骨科的临床应用. 中华创伤杂志. 2014. 30(11): 1085-1088.

[32] 糜菁熠, 芮永军, 沈小芳等. 带神经的修薄股前外侧皮瓣修复手部创面. 中华显微外科杂志. 2012. 35(6): 485-487.

[33] 黎健伟, 刘勇, 任义军等. 三维重建技术在髂骨瓣临床手术中的初步应用. 中华创伤骨科杂志. 2009. 11(4): 338-341.

[34] Li YG, Chen XJ, Zhang YZ, et al. Three-dimensional digitalized virtual planning for retrograde sural neurovascular island flaps: a comparative study. Burns. 2014. 40(5): 974-80.

[35] 王娇, 刘洋, 张晓玲, 王衍, 穆建玲, 赵燕. Mimics 软件在医学图像三维重建中

的应用. 医疗卫生装备. 2015. 36(02): 115-118.

[36] 唐举玉, 卿黎明, 贺继强等. 数字化技术辅助旋股外侧动脉降支分叶穿支皮瓣设计的初步应用. 中华显微外科杂志. 2016. 39(2): 123-126.

[37] 段家章, 何晓清, 徐永清等. 数字化技术在股前外侧皮瓣修复手足创面中的应用. 中国修复重建外科杂志. 2015. 29(07): 807-811.

[38] Chae MP, Lin F, Spychal RT, Hunter-Smith DJ, Rozen WM. 3D-printed haptic "reverse" models for preoperative planning in soft tissue reconstruction: a case report. Microsurgery. 2015. 35(2): 148-53.

[39] Chae MP, Hunter-Smith DJ, Spychal RT, Rozen WM. 3D volumetric analysis for planning breast reconstructive surgery. Breast Cancer Res Treat. 2014. 146(2): 457-60.

[40] 钟世镇. 我国数字医学发展史概要. 中国数字医学. 2011. 6(12): 12-14.

[41] Zhang YZ, Li YB, Jiang YH, Tang ML, Li JW, Pei GX. Three-dimensional reconstructive methods in the visualization of anterolateral thigh flap. Surg Radiol Anat. 2008. 30(1): 77-81.

附录

典型病例一：患者周 X，男性，9 岁，车祸伤致右足背软组织缺损，2018 年 07 月 03 日我院全麻下行游离左股前外侧皮瓣移植修复右足背创面，术前采用传统布样设计，术后供区无法完全覆盖受区创面

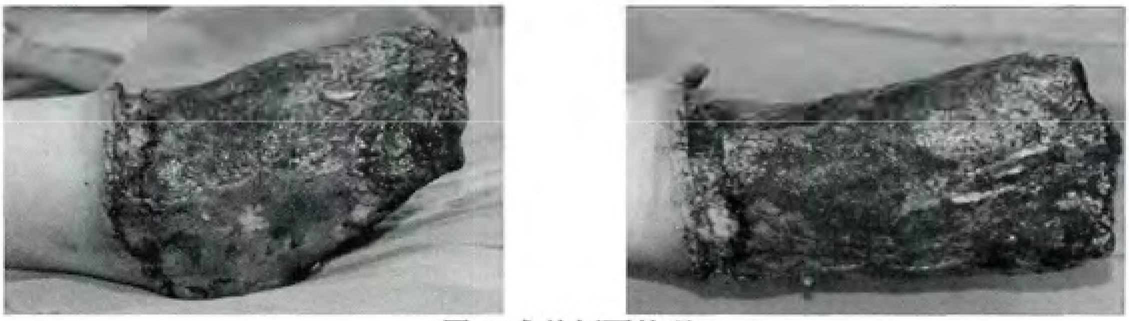

图 1 术前创面外观

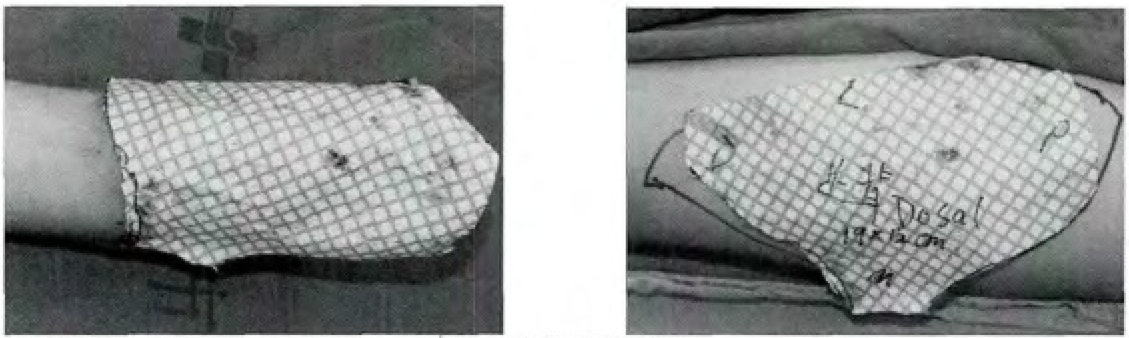

图 2 传统布样设计

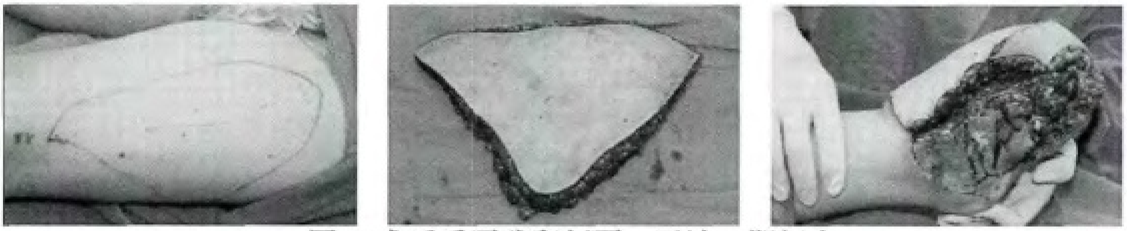

图 3 术后受区残留创面，无法一期闭合

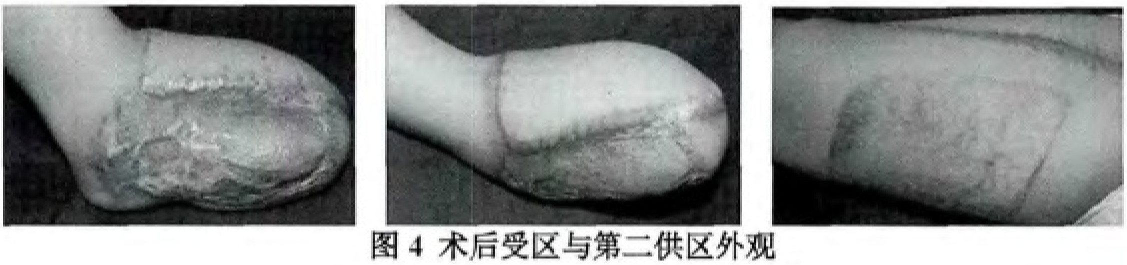

图 4 术后受区与第二供区外观

典型病例二： 患者邱 XX，女性，3 岁，车祸伤致右足跟部软组织缺损，2018 年 11 月 06 日我院全麻下行游离左股前外侧皮瓣移植修复右足跟，术前采用 Mimics 软件设计，术后供受区精准匹配

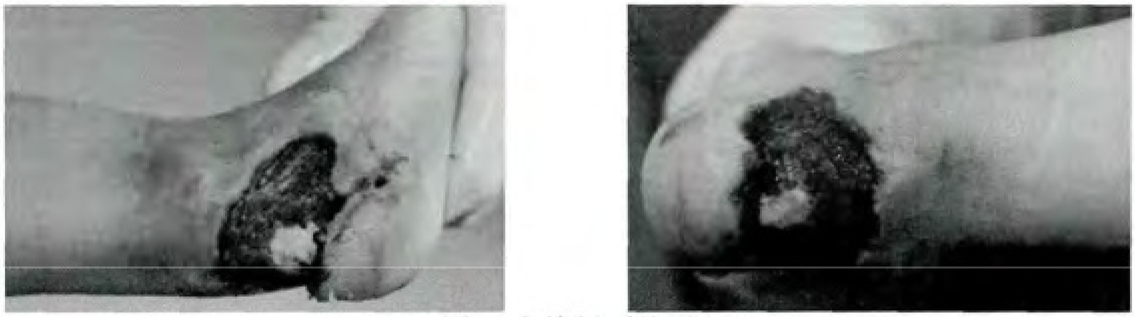

图 1 术前创面外观

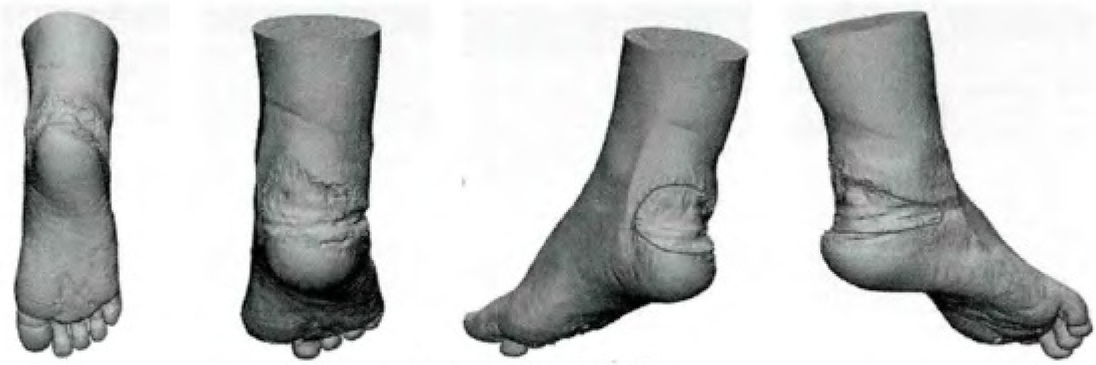

图 2 术前三维建模

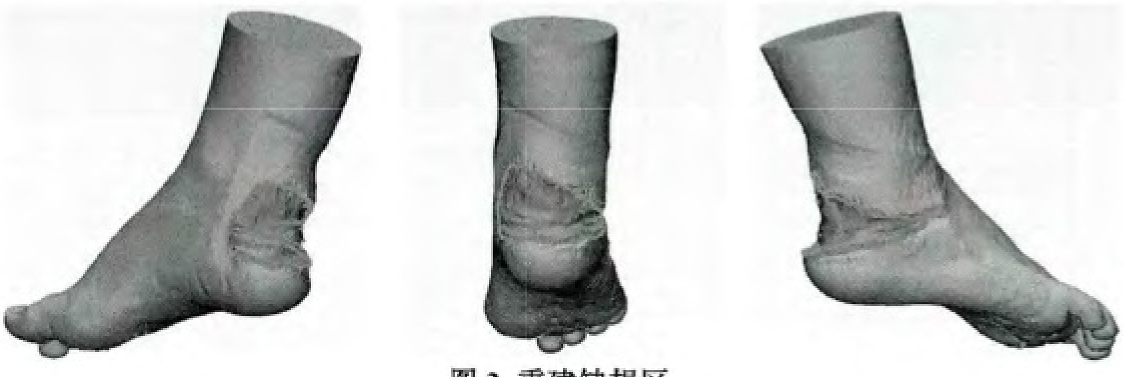

图 3 重建缺损区

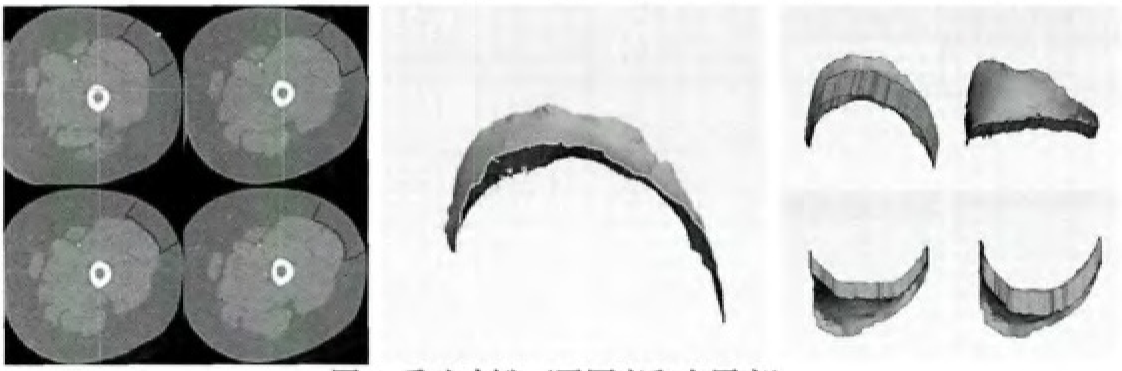

图 4 重建皮瓣（无厚度和有厚度）

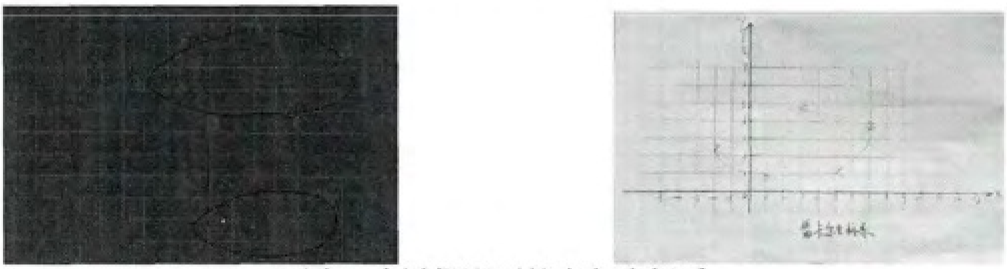

图 5 皮瓣投影至笛卡尔坐标系

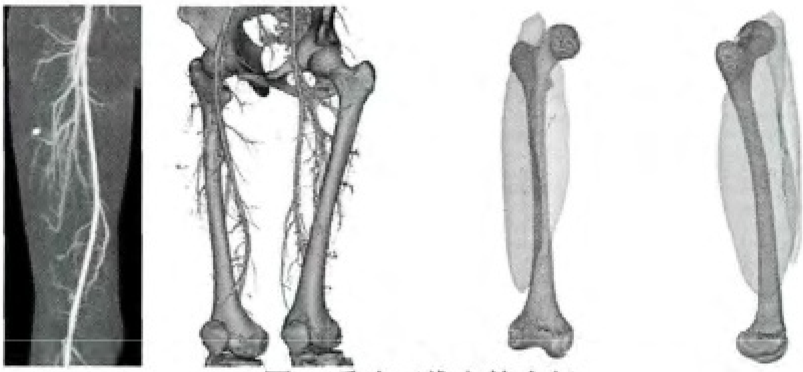

图 6 重建三维血管走行

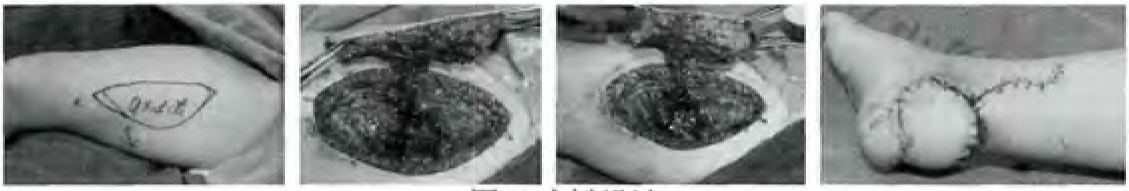

图 7 皮瓣设计

表 1 传统布样设计与 Mimics 设计对比

传统布样设计			皮瓣	Mimics 设计		
长度（cm）	宽度（cm）	表面积（cm^2）	厚度（cm）	长度（cm）	宽度（cm）	表面积（cm^2）
7.0	3.0	16.7	1.8	9.0	4.0	31.2

综　述　数字化技术在组织移植中的发展应用

惠涛涛　综述　　芮永军　审校

21世纪，数字医学逐渐成为医学发展的重要方向，它正从多个方面改变传统医学的思维模式[1-6]。近年来，随着外科手术难度的不断提高，医生不应该再单纯依靠手工测量、目测估计和空间想象力，而应该熟练地运用数字化技术软件建立三维模型，在计算机屏幕上模拟规划手术方案，实现精准医疗[7]。其中，在辅助外科手术方面表现最为突出，如复杂解剖结构的三维可视化及测量、人工假体个性化定制和虚拟仿真手术等[8]。目前，数字医学正逐渐渗透到医学的各个学科，如颌面外科、整形外科、骨科、手外科、显微外科等，从最早应用于神经外科、骨科、显微外科等，并从主要应用于骨性组织，逐渐应用于软组织[9-13]。随着高能量肢体创伤日益增多，软组织创面修复难度逐渐增加，数字医学技术与组织移植相结合将成为当今医学发展的必然要求。

一、数字化技术概念的提出

数字化技术概念的提出是建立在数字医学和中国数字人技术的基础之上的[14]。建立数字化三维解剖模型，以三维可视化进行外科手术术前设计与模拟，实现基础与临床、理论与实践的完美结合与转化[15]。术者在没有任何干扰的情况下观察、学习解剖结构，充分展示局部的解剖特征，包括臂丛、腰骶丛神经的三维结构观察以及数字化皮瓣的设计，同时以三维、全方位、可视化、动态的形式展示血管、肌肉、骨、皮肤的毗邻关系[16]。通过建立不同类型的数字化手术模型，可逼真地赋予手术模型组织一定的弹性、张力、厚度、表面积、体积等[17]，完全可模拟仿真出一种真实的手术操作过程，立体显示手术人路的毗邻解剖关系，从而大大提高手术操作的精确度，实现精准医疗。

二、数字化技术在医学中的应用现状

随着社会的进步以及生活水平的提高，患者对手术效果提出更高的要求，不仅要获得良好的感觉与功能，而且要求外形更加美观。因此，术前精准的手术设计，是我们应努力追求的目标，也是摆在我们面前的重要课题。如何寻求一种规范化、个性化、

精确化的方法是未来外科手术的发展方向。数字化医学技术通过建立个性化的三维图像，在重建软件上精确设计，使手术更加精确、安全、可靠。由于以上优点，目前数字化技术已广泛应用于人体组织器官、整形外科、颌面外科、显微外科、手外科、骨科以及临床教学等领域[18-19]。

在人体组织器官领域，数字化技术已成功被运用于人体活体组织器官模型制造。3D打印人造血管：德国研究人员利用3D打印技术，打印制作出柔韧的人造血管，并可使血管与人体融合，同时解决了血管免遭人体排斥的问题[20]。3D打印制作脸部损伤组织：与传统技术相比，该方法可以得到与患者精确匹配的相应组织，为患者重新塑造头部完整形象，外形更加美观[21]。

在整形外科领域，传统的整形外科模式是凭借医师的主观经验进行诊断分析和手术设计，缺乏客观的判断指标和有效的辅助手段，极大地影响到手术效果和手术的安全性。数字医学技术主要应用于创伤或肿瘤根治性切除所致的缺损修复，乳房整形再造，均取得了良好的效果[22]。Craig N.Creasman等[23]术前通过三维软件重建出患者双侧乳房，一方面患者可清晰地观察到手术效果，另一方面术者可进行精确的术前设计。Michael P.Chae等[22]将患者双侧乳房原始CT数据导入三维重建软件，术前评估乳房体积，并在计算机辅助下进行精准设计，制定量化、个性化的手术方案，效果显著。Edmund Fitzgerald O·Connor等[24]通过腹壁下深动脉穿支皮瓣对乳房进行再造，术前依靠CTA准确定位血管位置，管径，血管蒂长度以及血管走行，可明显减少手术时间，显著降低术后并发症。Emilio Garcia-Tutor等[25]对5名患者骶尾部缺损区进行CT扫描，通过计算机辅助测量分析缺损区形状，大小，面积，最后切取皮瓣精准修复缺损部位。

在颌面外科领域，利用数字化技术有着显著地优越性，可以显著提高精确度，制造的矫正器解决了矫形手段与辅助工具不能灵活调整的问题，能够做到矫形部位与矫形器完全匹配，提高了矫形器的拟合效果[26]。对于面部主要美学特征的重建需要精细化和个性化的计算，传统的重建手术主要依赖术者的经验、手术技巧及重建理念，也受制于供区皮瓣制备的一些局限性，可能造成重建效果难以预测或不理想。随着患者要求逐渐提高，外科医师对于重建手术的精确性、重建效果的美观和功能都有了更高的要求，数字化外科技术的辅助使重建手术更加个性化、精细化[27]。由于半侧颜面萎缩面部畸形较为复杂。完全达到解剖复位困难较大，数字化皮瓣能够比较清晰的反映组织之间的毗邻关系[28]。通过计算机辅助软件及在头颅模型上确定皮瓣填充模型的大

小，然后在手术过程中，按照模型指导手术中皮瓣大小的选择。从而达到与头颅模型模拟吻合的手术效果。

在显微外科领域，数字医学技术主要应用于术前皮瓣设计及皮瓣穿支解剖学研究等，具有良好的临床应用前景。Warren M.Rozen等[29-31]报道，术前CTA不仅可以找出最佳的穿支血管，而且可显著提高皮瓣存活率，降低供区并发症。尽管CTA等穿支血管定位技术已经成熟，但无法为术者清晰地展示血管走行的三维图像。Yuanzhi Zhang等[32]把穿支血管CTA数据导入Amira3.1软件中，可将二维穿支血管走行转化为三维图像，同时可以360°旋转观察，对切取皮瓣具有重要指导意义。齐向东等[28]术前应用数字化技术对半侧颜面萎缩患者进行CT血管成像扫描，三维重建畸形部位，设计修复所需要的个性化复合组织瓣，可前瞻性了解手术效果，能够精确切取皮瓣范围，并可以避免术中血管吻合时受区、供区不匹配现象。Michael P.Chae等[33]通过逆行功能及快速成形技术重建出缺损区，进行术前精确设计，可明显减少手术时间及术中失血量。另外，数字化医学技术在穿支血管以及简单创面设计中的应用，为复杂创面设计提供了理论基础。传统皮瓣移植的治疗目标主要是追求皮瓣成活和创面愈合，对皮瓣受区的外形与功能恢复、特别是对皮瓣供受区损害问题未引起足够重视[34]。临床随访发现部分患者皮瓣移植术后供受区均存在难看的瘢痕、甚至严重的功能障碍，对患者身心造成严重的影响。随着人们生活水平的提高，一方面对创面重建的要求越来越高，要求受区创面修复后能够获得最好的外形和功能；另一方面期望皮瓣供区的外观和功能损害尽可能最小化。唐举玉等[34]学者提出穿支皮瓣技术的精髓是“微创与美学”，其临床应用遵循“最大得失比”原则，即以最小的供区损害获得最佳的受区外形和功能，在重视皮瓣受区外形和功能重建的同时尽可能减少皮瓣供区外观和功能的损害。程国良等[35]学者在国内较早提出了修饰性再造的理念，使组织移植“功能第一、外形第二”的手术理念逐渐发展为“功能第一、外形第一”“修饰性修复与重建”。皮瓣供区选择出来遵循“功能相对次要、皮瓣血供可靠、供区尽量隐蔽”外，还应考虑皮瓣供区颜色、质地、感觉、厚度、移动度是否符合受区重建要求。随着数字化技术的不断发展以及在临床上的应用，困扰医生的上述问题将会得到较好地解决。数字化技术能提供更具体资料数据，包括皮瓣血供的血管起源、走行、类型，管径大小及血管蒂长度，以及穿支血管类型、数量、位置等详细信息；是直观的、三维的图像，对皮瓣设计及术中避免损伤都很有帮助，对临床具有指导意义。因此，术前充分了解皮瓣血供动脉

的起源、走向、分型、穿支点位置及穿支数量以及其与周围组织的毗邻关系尤为重要。术前建立三维可视化的皮瓣穿支血管解剖图像则有望解决上述的问题。

在手外科领域，数字医学技术主要应用于手部解剖研究。有学者等建立了基于解剖结构的可视化手模型并精确显示手掌部主要解剖结构[36]。黄潮桐等[37]报道的数字化虚拟手若干关键技术的研究为临床诊断、治疗方案的制定、手术相关操作等提供科学的依据。谭海涛等[38-39]通过数字化技术辅助足趾移植再造拇手指，术前在计算机模拟手术操作，可明显减少手术并发症，提高手术成活率，实现个体化治疗。

在骨科领域，数字化技术正在飞速发展，主要包括虚拟现实技术，逆向工程和快速成形技术[40]。目前，数字化技术主要应用于复杂创伤、脊柱外科、关节外科和小儿骨科等术前计划和手术模拟，在临床工作中展现出良好的应用前景。在复杂创伤方面，Long Yang等[10]通过64排双螺旋CT对肘关节周围进行扫描，将原始DICOM数据导入Mimics软件并进行三维重建，同时进行3D打印，可直观显示骨折块移位及粉碎情况，术前可进行详细地设计规划，包括如何对骨折块进行精确复位，如何维持骨折断端复位，选用何种钢板、如何对钢板安全塑形、放置钢板、置入螺钉，从而使得手术更加精准和安全，有利于提高手术效率、缩短手术时间、减少手术出血量，降低术后并发症。另外，数字化技术还运用于骨盆骨折、髋臼骨折、上下肢复杂关节内骨折等。Zhaojie Liu等[11，41]通过对患侧髋臼进行CT扫描，将获得的DICOM数据导入数字化软件中，进行三维重建，并打印髋臼骨折模型，一方面术者通过模型为患者及其家属讲解手术过程，让患者更了解自身病情和治疗过程；另一方面，可帮助术者对骨折准确分型，选择最佳的手术入路、钢板塑形以及螺钉位置等，进而缩短手术时间。在脊柱外科方面，由于脊柱与中枢神经相连，手术风险较高。因此，数字化技术主要被应用于制作个性化手术假体可提高手术精准度，就能使复杂的手术变得精准可靠[42]。Xiucan Li等[43]通过外科手术切除上颈椎甲状腺转移癌，再利用3D打印个性化内植物重建上颈椎连续性，可明显缩短手术时间，降低手术风险。Fengning Li等[42]将下颈椎CT原始扫描数据导入Mimics软件，进行三维重建，并通过3D打印制作模型，测量下颈椎螺钉相关参数以及最佳螺钉通道，有利于下颈椎螺钉准确置入，提高手术安全性。在关节手术中，根据患者自身的解剖特点，数字化技术可制作出个性化导板。Bing Qiu等[44]通过数字化技术术前个性化制作患者膝关节置换假体，可降低术中显性失血及术后隐性失血，缩短住院周期，提高手术效果。在关节置换手术中个性化导航模板可以精

确地引导髋臼假体置入的位置；在部分需要截骨矫形的手术中个性化截骨模板同样显示了其优越性。另外，通过逆向工程与快速成型技术设计的“个性化数字导航模板”已经初步应用于临床，在实施螺钉置人手术时可以预先通过逆向工程原理在三维的椎体模型上可以寻找到最佳的内固定置入方向、长度及角度，然后利用快速成型技术将计算机三维重建和逆向工程技术获得的模型及导航模板生产成实物模型，加以验证、实施手术。

在教学领域，可视化三维模型有助于更好地理解相关解剖部位，有利于指导医生个性化治疗和诊断，同时打印出来的模型能将器官和组织内部结构的细节逼真地显示出来，使医学知识变得更为直观明了，可用于临床、教学和术前模拟、优化手术方案，实现精确化、个性化手术。数字化技术主要应用于与患者术前沟通及医学教育教学等。传统的二维图像无法清晰提供立体的三维图像，完全依赖于我们主观的想象力，而三维立体图像具有很强的视觉冲击力，给人以形象、真实的感觉。一方面，当与患者进行术前沟通时，即使外科医师解剖基础扎实，手术技巧精湛，但仍无法通过二维平面图像与患者进行详细的沟通，最重要的是，在短时间内，患者及其家属也无法准确理解临床医师所告知的病情及手术方案，最终常常导致医患沟通失败，在一定程度上增加了医患纠纷和矛盾，阻碍医患关系和谐发展。而数字化技术可通过三维立体图像或制作的三维实物模型，帮助外科医生更好地进行复杂手术前研究，并与患者进行有效的沟通，使患者全面了解自身的病情。同时，数字化医学技术可允许外科医生反复多次进行术前规划，与患者及其家属共同参与手术方案设计，能够帮助患者理解“手术原因、手术方式、手术效果、手术风险、手术相关并发症”等问题。Schmauss D等[45]联合运用三维重建技术与3D打印技术制备人体心脏可视化实物模型，用于复杂手术前的研究，使术者全面掌握患者人体器官解剖结构，尤其是血管神经与周围组织空间结构毗邻关系。Laura Olivieri等[46]数字化技术重建出器质性心脏病患者心脏模型，可用于术前分析组织解剖关系及患者病情，指导介入入路，并可帮助选择合适的介入管，可显著提高手术效率，减少术者放射暴露次数。另一方面，在医学教育教学上，数字化技术能够改变以往单一死板的书本教学模式。Jean H.D. Fasel等[47]对2名尸体进行全身CT扫描，将获得的DICOM数据导入Osirix或Mimics数字化软件中，三维可视化重建出所需要的解剖结构，指导临床学生高效直观学习解剖。Justyn Pisa, AuD等[48]描述了一种新的数字化技术，可重建并打印出人体颅骨内部解剖结构，与传统尸体解剖相

比较，这种解剖模型是一种非常有价值的教学工具。Maria Teresa Ugidos Lozano等[49]认为，医学生需要掌握扎实的人体解剖学知识，传统教学模式以尸体解剖为主，然而这种教学模式缺乏灵活性，他们通过计算机辅助技术重建出颅骨三维模型，将其运用于医学本科生教学中，形象生动，可以明显提高临床教学效率。另外，通过对手术的模拟仿真训练，有利于使外科手术训练规范化、程序化、标准化，从而有助于外科医生的规范化培训，有助于提高手术的质量与治疗效果。

三、数字化技术在组织移植中应用的优势

传统的手术模式为“开放—观察—手术”，即首先开放手术部位，依靠临床医师长期积累的经验，通过对受区创面的主观观察进行手术，因而手术具有很大的盲目性，从而增加了手术风险。数字化可视技术为解决该难题提供了技术平台，术前采用CT或MRI扫描，将获得的DICOM数据导入Mimics软件，重建个性化三维图像，可将传统模式改变为“观察—开放—手术”，从而实现二维图像向立体图像、平面向立体、静态向动态的转变，实现组织移植的三维可视化。通过该方法，主刀医生可于术前对手术部位做深入的观察，术前确定手术方案，在手术规划软件上观察三维模型，对手术部位深入观察，使手术更加精确与微创，提升手术安全性。数字化技术从术前诊断设计、手术模拟、术中导航等全方位引领外科技术的深入发展，从而促使外科技术向个性化、精确化、微创化和远程化方向快速发展。其优势在于：精确定位术区解剖结构及病理组织；术前仿真模拟，制定手术方案；术中三维可视实时导航；确定切除范围和手术入路；虚拟现实、辅助教学及远程医疗。

首先，在以往的组织移植中，医生在术前制定的手术方案常缺乏对供区皮瓣厚度以及受区创面弧度定量描述，术后常常出现供受区不匹配现象。其次，主刀医生的构思和手术经验很难为手术组每一位成员所共享，手术操作不易达成默契。数字化技术不仅可以设计修复所应用的皮瓣，能够显示位置、大小、形状，而且能够计算厚度、表面积和体积，制定最佳手术方案，减少了手术中的出血、创伤，缩短了手术时间，实现精准医疗。同时改变了以前传统的通过目测和临床经验来判断手术的效果缺点。通过数字化技术设计皮瓣增加了手术的精确性，保证了手术后形态功能上的良好效果，根据模拟手术的效果，前瞻性的了解术后效果及可能存在的不足。

另外，对于复杂穿支血管的分布，术者在术前不能完全了解穿支血管的走行及其与周围结构的解剖关系，皮瓣切取时多凭借临床医生自己长期积累的经验和教训，往

往会延长手术时间，加大手术难度，并有可能增加副损伤的几率，一旦出现与术前设计不符，甚至造成手术方式终止，需改变手术方式。而数字化技术通过对皮瓣结构进行三维重建，能够清晰显示穿支血管的走行与变异情况．避免术中因血管变异导致手术失败，减少术者对经验与教训的依赖，有助于提高手术质量。

四、数字化技术在组织移植的实施与要求

1．数据的获取与手术方案的设计：术前经肘正中静脉注射碘海醇造影剂，采用64排双源螺旋CT对供受区同时进行CT扫描，以DICOM格式导人Mimics20.0重建软件，分别对骨骼、软组织Masks层进行三维构建转换。先建立一个新的蒙版（New Mask），建立成功后点击Clear Mask，在水平面图像中找到肌肉的起止点。找到肌肉起点，点击Multiple Slice Edit,选择Live Wire，可自行标记肌肉边界，一直标记到肌肉止点，所有层面均标记后点击Interpolate，再点击Apply，即可生成水平面图像上所有层面的肌肉图像。如果在标记过程中出现错误，可按Ctrl + Z进行修改。在Mask中点击Calculate Part from Mask，可在Mask下方的Parts生成三维图像。在Parts中鼠标右键点击已生成的三维图像，点击Copy。打开3-matic Medical 12.0，在Work Area中按Ctrl + V，即可显现Mimics Medical 20.0创建的肌肉三维图像。在窗口上方的功能区中，点击Fix，选择Reduce，将右下方Reduce参数中Geometrical error更改为0.4000，点击Apply；再点击Fix，选中Smooth，将右下方Smooth参数中Smooth factor更改为0.8000，点击Apply,即可生成表面比较光滑的肌肉。缺损区重建：点击开始阈值分割（start thresholding）选项，点击Custom，调整像素灰度值，将水平面图像中骨、血管、肌肉、脂肪和皮肤全部覆盖。利用区域增长（Region Growing）及裁剪蒙版（Crop Mask）将患肢从双侧肢体中单独分离出来。在Mask中点击Calculate Part from Mask，可在Mask下方的Parts生成患肢的三维图像。在Parts中鼠标右键点击已生成的三维图像，点击Copy。打开3-matic Medical 12.0，在Work Area中按Ctrl + V，即可显现Mimics Medical 20.0创建的患肢三维图像。鼠标左键点击键面上方主工具栏中Mark，再点击分组工具栏中Lasso Area Mark，填补缺损区域，在填充过程中不断点击Expand，直到完全填补缺损。注意在填补过程中不要有遗漏。填充成功后，鼠标左键点击键面主工具栏Mark中Smooth Marking Border，即可生成软组织缺损区轮廓。鼠标左键点击缺损区轮廓，并按住Shift键，可弹出Surface，点击Surface，在右上方项目管理器中出现Surface，鼠标右键点击Surface，分别选择Separate-Copy to Part-Create Part，可另外单

独生成软组织缺损区域。鼠标左键点击左上方的Surface，在右下方可分别自动显示缺损区域长、宽、表面积和体积。利用软件自带的测量工具，测量供区的厚度。点击键面左上方主工具栏中Design，再点击Local Offset，右下方弹出对话框，将Offset参数中Offset distance更改为供区的厚度，点击Apply，可生成具有厚度的缺损区域。此时鼠标左键再次点击左上方的Surface，在右下方可分别自动显示缺损区域长、宽、表面积和体积。

2. 手术方法及术后处理：供、受区血管的处理：首先准备受区，彻底清创，清除坏死的骨及软组织。根据术前Mimics20.0软件构建的个性化组织瓣，对患者供区进行点、线、面描记，并依重建图像切取组织瓣。③术后处理：常规监测生命体征，并观测皮瓣的存活情况。一般术后当天，每2h观察1次；术后l-2d，每4h观察1次；术后3～7d，每6h观察1次，重点观察皮瓣的皮温、皮色、毛细血管充盈情况及肿胀程度。常规使用抗痉挛、抗凝药物及抗生素。若发现皮瓣苍白或肿胀呈暗紫色或毛细血管充盈异常。分析导致血供障碍的原因，并相应给予保暖、解痉、止痛或拆线解除血管蒂压迫等处理，如处理后无好转，应立即手术探查，尽快恢复血供。

3. 数字医学技术在组织移植的要求：受区CT扫描参数：电压100kV、电流250mA、层厚1.0mm；供区CTA扫描参数：电压100KV、电流250mA、层厚0.7mm、层距0.625mm。②在软组织重建方面，需多多学科合作，以确保软组织重建图像的真实性。③在组织移植手术方面，要求主刀医师具有良好的显微外科、手外科操作技术水平，术前要充分利用数字医学技术对组织移植进行精心设计，根据术前设计的足趾组织瓣，术中仔细校对穿支血管走行，并依重建图像切取皮瓣，确保手术取得成功，提高手术的安全性。

五、展望

精准医疗是21世纪组织工程技术完成组织器官修复重建过程中的重要环节，数字技术将推动和完善这一环节。数字化技术已广泛应用于显微外科领域，实现了人体空间中的准确定位、三维测量数据和立体图像。这将为人体解剖学科和外科学科带来一次划时代性的革命。

为保证病人安全，提高手术安全性、精确性，提高治疗效果，提高科研及医疗的质量，应用数字医学技术辅助组织移植将是发展趋势。通过数字医学技术的研究，实现人体组织的三维可视化，仿真模拟临床上一些复杂的诊疗活动，预先制定治疗方案，

可能使临床科研工作发生根本性的变化，在医学领域中具有广泛的应用前景。

参考文献

[1] 钟世镇. 数字医学在不同学科中的探索应用[J].中华整形外科杂志,2018,34(6):前插 2.DOI:10.3760/cma.j.issn.1009-4598.2018.06.000.Chin J Plast Surg,2018,34(6):前插 2. DOI: 10.3760/cma.j.issn.1009-4598.2018.06.000.

[2] 郭传瑸. 数字医学技术是口腔颌面外科发展的一个重要方向[J].中华口腔医学杂志,2017,52(4):201-203. DOI: 10.3760/cma.j.issn.1002-0098.2017.04.001. Chuan bin G. Digital medical techniques play an important role in the development of oral and maxillofacial surgery[J]. Chin J Stomatol,2017,52(4):201-203. DOI: 10.3760/cma.j.issn.1002-0098.2017.04.001.

[3] 裴国献, 张元智. 数字骨科学:一门骨科学新分支的萌生[J].中华创伤骨科杂志,2007,9(7):601-604. DOI: 10.3760/cma.j.issn.1671-7600.2007.07.001. Guo-xian P, Yuan-zhi Z. Digital orthopaedics: a new branch of orthopaedics[J].Chin J OrthopTrauma,2007,9(7):601-604.DOI:10.3760/cma.j.issn.1671-7600.2007.07.001.

[4] 齐向东，祁佐良. 数字医学技术在整形外科中的应用[J].中华整形外科杂志,2018,34(6):407-412. The application of digital medicine in plastic and reconstructive surgery[J]. Chin J Plast Surg,2018,34(6):407-412.

[5] 张益，刘筱菁. 数字技术改变着现有的诊疗思维和模式：从外科谈起[J].中华口腔医学杂志,2016,(4):205-209. Impact of digital technology on clinical practices: perspectives from surgery[J]. Chin J Stomatol,2016,(4):205-209.

[6] 王正国，张良. 数字医学进展[J].重庆医学,2016,45(16):2161-2162. Chongqing Medical Journal,2016,45(16):2161-2162.

[7] Tan H, Yang K, Wei P, et al. A Novel Preoperative Planning Technique Using a Combination of CT Angiography and Three-Dimensional Printing for Complex Toe-to-Hand Reconstruction[J]. J Reconstr Microsurg, 2015,31(5):369-377. DOI: 10.1055/s-0035-1546419.

[8] 张绍祥. 对推动我国数字医学发展的几点认识与思考[J].中国数字医学,2009,4(01):15-18. 2009,4(01):15-18.

[9] Zeng C, Xing W, Wu Z, et al. A combination of three-dimensional printing and computer-assisted virtual surgical procedure for preoperative planning of acetabular fracture reduction[J]. Injury, 2016,47(10):2223-2227. DOI: 10.1016/j.injury.2016.03.015.

[10] Yang L, Grottkau B, He Z, et al. Three dimensional printing technology and materials for treatment of elbow fractures[J]. Int Orthop, 2017,41(11):2381-2387. DOI: 10.1007/s00264-017-3627-7.

[11] Liu ZJ, Jia J, Zhang YG, et al. Internal Fixation of Complicated Acetabular Fractures Directed by Preoperative Surgery with 3D Printing Models[J]. Orthop Surg, 2017,9(2):257-260. DOI: 10.1111/os.12324.

[12] Chan HH, Siewerdsen JH, Vescan A, et al. 3D Rapid Prototyping for Otolaryngology-Head and Neck Surgery: Applications in Image-Guidance, Surgical Simulation and Patient-Specific Modeling[J]. PLoS One, 2015,10(9):e0136370. DOI: 10.1371/journal.pone.0136370.

[13] Matarasso A, Smith DM. Combined breast surgery and abdominoplasty: strategies for success[J]. Plast Reconstr Surg, 2015,135(5):849e-860e. DOI: 10.1097/PRS.0000000000001238.

[14] 张元智, 陆声, 赵建民, 等. 数字化技术在骨科的临床应用[J].中华创伤骨科杂志,2011,13(12):1161-1165. DOI: 10.3760/cma.j.issn.1671-7600.2011.12.015.

Digital technology used in orthopedic surgery[J]. Chin J Orthop Trauma,2011,13(12):1161-1165. DOI: 10.3760/cma.j.issn.1671-7600.2011.12.015.

[15] Dérand P, Rännar LE, Hirsch JM. Imaging, virtual planning, design, and production of patient-specific implants and clinical validation in craniomaxillofacial surgery[J]. Craniomaxillofac Trauma Reconstr, 2012,5(3):137-144. DOI: 10.1055/s-0032-1313357.

[16] Rozen WM, Chubb D, Ashton MW, et al. Mapping the vascular anatomy of free transplanted soft tissue flaps with computed tomographic angiography[J]. Surg Radiol Anat, 2012,34(4):301-304. DOI: 10.1007/s00276-011-0885-8.

[17] 王凌宇, 栾杰, 李彦生, 等. CT 数据三维重建测量乳房体积在乳房整形中的应用 [J]. 组织工程与重建外科杂志 ,2012,8(3):150-153. DOI: 10.3969/j.issn.1673-0364.2012.03.007.

Lingyu W, Jie L, Yansheng LI, et al.Clinical Application of Three-Dimensional

Reconstruction Technique for Breast Volume Measurement in Mammaplasty[J].Journal of Tissue Engineering and Reconstructive Surgery,2012,8(3):150-153. DOI: 10.3969/j.issn.1673-0364.2012.03.007.

[18] 王岩. 骨科精准医疗:应用与思考[J].中华医学杂志,2015,95(31):2512-2514. DOI: 10.3760/cma.j.issn.0376-2491.2015.31.007.
@@[J].Natl Med J China,2015,95(31):2512-2514. DOI: 10.3760/cma.j.issn.0376-2491.2015.31.007.

[19] Martelli N, Serrano C, van den Brink H, et al. Advantages and disadvantages of 3-dimensional printing in surgery: A systematic review[J]. Surgery, 2016,159(6):1485-1500. DOI: 10.1016/j.surg.2015.12.017.

[20] Murphy SV, Atala A. 3D bioprinting of tissues and organs[J]. Nat Biotechnol, 2014,32(8):773-785. DOI: 10.1038/nbt.2958.

[21] Sabol JV, Grant GT, Liacouras P, et al. Digital image capture and rapid prototyping of the maxillofacial defect[J]. J Prosthodont, 2011,20(4):310-314. DOI: 10.1111/j.1532-849X.2011.00701.x.

[22] Chae MP, Rozen WM, Spychal RT, et al. Breast volumetric analysis for aesthetic planning in breast reconstruction: a literature review of techniques[J]. Gland Surg, 2016,5(2):212-226. DOI: 10.3978/j.issn.2227-684X.2015.10.03.

[23] Creasman CN, Mordaunt D, Liolios T, et al. Four-dimensional breast imaging, part II: clinical implementation and validation of a computer imaging system for breast augmentation planning[J]. Aesthet Surg J, 2011,31(8):925-938. DOI: 10.1177/1090820X11424147.

[24] Fitzgerald OE, Rozen WM, Chowdhry M, et al. Preoperative computed tomography angiography for planning DIEP flap breast reconstruction reduces operative time and overall complications[J]. Gland Surg, 2016,5(2):93-98. DOI: 10.3978/j.issn.2227-684X.2015.05.17.

[25] Garcia-Tutor E, Romeo M, Chae MP, et al. 3D Volumetric Modeling and Microvascular Reconstruction of Irradiated Lumbosacral Defects after Oncologic Resection[J]. Front Surg, 2016,3:66. DOI: 10.3389/fsurg.2016.00066.

[26] Chen S, Pan Z, Wu Y, et al. The role of three-dimensional printed models of skull in anatomy education: a randomized controlled trail[J]. Sci Rep, 2017,7(1):575. DOI: 10.1038/s41598-017-00647-1.

[27] Cohen J, Reyes SA. Creation of a 3D printed temporal bone model from clinical CT data[J]. Am J Otolaryngol, 2015,36(5):619-624. DOI: 10.1016/j.amjoto.2015.02.012.

[28] 齐向东，马立敏，张斌，等. 数字化技术对半侧颜面萎缩修复皮瓣的选择应用[J]. 中华显微外科杂志,2011,34(6):454-456. DOI: 10.3760/cma.j.issn.1001-2036.2011.06.006.
Xiang-dong QI, Li-min MA, Bin Z, et al. Selecting flap repaire hemifacial atrophy by digital technology[J]. Chin J Microsurg,2011,34(6):454-456. DOI: 10.3760/cma.j.issn.1001-2036.2011.06.006.

[29] Rozen WM, Ashton MW, Pan WR, et al. Anatomical variations in the harvest of anterolateral thigh flap perforators: a cadaveric and clinical study[J]. Microsurgery, 2009,29(1):16-23. DOI: 10.1002/micr.20550.

[30] Rozen WM, Ashton MW, Grinsell D, et al. Establishing the case for CT angiography in the preoperative imaging of abdominal wall perforators[J]. Microsurgery, 2008,28(5):306-313. DOI: 10.1002/micr.20496.

[31] Rozen WM, Anavekar NS, Ashton MW, et al. Does the preoperative imaging of perforators with CT angiography improve operative outcomes in breast reconstruction?[J]. Microsurgery, 2008,28(7):516-523. DOI: 10.1002/micr.20526.

[32] Zhang YZ, Li YB, Jiang YH, et al. Three-dimensional reconstructive methods in the visualization of anterolateral thigh flap[J]. Surg Radiol Anat, 2008,30(1):77-81. DOI: 10.1007/s00276-007-0287-0.

[33] Chae MP, Lin F, Spychal RT, et al. 3D-printed haptic "reverse" models for preoperative planning in soft tissue reconstruction: a case report[J]. Microsurgery, 2015,35(2):148-153. DOI: 10.1002/micr.22293.

[34] 唐举玉，汪华侨，Hallock GG, 等. 关注皮瓣供区问题—减少皮瓣供区损害专家共识[J]. 中华显微外科杂志,2018,41(1):3-5.DOI: 10.3760/cma.j.issn.1001-2036.2018.01.001.Chin J Microsurg,2018,41(1):3-5. DOI: 10.3760/cma.j.issn.1001-2036.2018.01.001.

[35] 程国良. 我国足趾移植拇手指再造与修复回顾与展望[J].中华手外科杂志,2007,23(2):65-68. DOI: 10.3760/cma.j.issn.1005-054X.2007.02.001.
Guo-liang C.A review and prospect of toe-to-hand transplantation in China[J].Chin J Hand Surg,2007,23(2):65-68. DOI: 10.3760/cma.j.issn.1005-054X.2007.02.001.

[36] 白桂有，张正治，熊雁，等. 手掌部分解剖结构可视化的初步研究[J].中国临床解剖学杂志,2005,23(3):227-229. DOI: 10.3969/j.issn.1001-165X.2005.03.001.

A preliminary study of visualization of anatomical structures of the hand[J].CHINESE JOURNAL OF CLINICAL ANATOMY,2005,23(3):227-229. DOI: 10.3969/j.issn.1001-165X.2005.03.001.

[37] 陈隆福，黄潮桐，李敬矿，等. 虚拟手在纵形断指临床解剖学的研究[J].中国临床解剖学杂志,2011,29(01):45-50. 2011,29(01):45-50.

[38] Wang L, Tian G, Wang M, et al. Analysis of the morphologic differences of the second toe and digits of the hand, and evaluation of potential surgical intervention to minimize the differences using computer-aided design technology[J]. Plast Reconstr Surg, 2014,134(6):902e-12e. DOI: 10.1097/PRS.0000000000000761.

[39] Zang CW, Zhang JL, Meng ZZ, et al. 3D Printing Technology in Planning Thumb Reconstructions with Second Toe Transplant[J]. Orthop Surg, 2017,9(2):215-220. DOI: 10.1111/os.12326.

[40] Chen YX, Zhang K, Hao YN, et al. Research status and application prospects of digital technology in orthopaedics[J]. Orthop Surg, 2012,4(3):131-138. DOI: 10.1111/j.1757-7861.2012.00184.x.

[41] Zeng C, Xiao J, Wu Z, et al. Evaluation of three-dimensional printing for internal fixation of unstable pelvic fracture from minimal invasive para-rectus abdominis approach: a preliminary report[J]. Int J Clin Exp Med, 2015,8(8):13039-13044.

[42] Li F, Huang X, Wang K, et al. Preparation and Assessment of an Individualized Navigation Template for Lower Cervical Anterior Transpedicular Screw Insertion Using a Three-Dimensional Printing Technique[J]. Spine (Phila Pa 1976), 2018,43(6):E348-348E356. DOI: 10.1097/BRS.0000000000002341.

[43] Li X, Wang Y, Zhao Y, et al. Multilevel 3D Printing Implant for Reconstructing Cervical Spine With Metastatic Papillary Thyroid Carcinoma[J]. Spine (Phila Pa 1976), 2017,42(22):E1326-1326E1330. DOI: 10.1097/BRS.0000000000002229.

[44] Qiu B, Liu F, Tang B, et al. Clinical Study of 3D Imaging and 3D Printing Technique for Patient-Specific Instrumentation in Total Knee Arthroplasty[J]. J Knee Surg, 2017,30(8):822-828. DOI: 10.1055/s-0036-1597980.

[45] Schmauss D, Haeberle S, Hagl C, et al. Three-dimensional printing in cardiac surgery and interventional cardiology: a single-centre experience[J]. Eur J

Cardiothorac Surg, 2015,47(6):1044-1052. DOI: 10.1093/ejcts/ezu310.

[46] Olivieri L, Krieger A, Chen MY, et al. 3D heart model guides complex stent angioplasty of pulmonary venous baffle obstruction in a Mustard repair of D-TGA[J]. Int J Cardiol, 2014,172(2):e297-298. DOI: 10.1016/j.ijcard.2013.12.192.

[47] Fasel JH, Aguiar D, Kiss-Bodolay D, et al. Adapting anatomy teaching to surgical trends: a combination of classical dissection, medical imaging, and 3D-printing technologies[J]. Surg Radiol Anat, 2016,38(3):361-367. DOI: 10.1007/s00276-015-1588-3.

[48] Hochman JB, Rhodes C, Wong D, et al. Comparison of cadaveric and isomorphic three-dimensional printed models in temporal bone education[J]. Laryngoscope, 2015,125(10):2353-2357. DOI: 10.1002/lary.24919.

[49] MTU L, Haro FB, Diaz CM, et al. 3D Digitization and Prototyping of the Skull for Practical Use in the Teaching of Human Anatomy[J]. J Med Syst, 2017,41(5):83. DOI: 10.1007/s10916-017-0728-1.

中英文缩略词表

英文缩写	英文全称	中文全称
CTA	Computed Tomography Angiography	计算机断层扫描血管造影
CT	Computed Tomography	计算机断层扫描
CDS	Color Duplex Sonography	彩色超声多普勒
MRA	Magnetic Resonance Angiography	核磁共振血管造影
CAD	computer aided design	计算机辅助设计
Mimics	Materiaise's interactive medical image control system	Materialise 公司的交互式的医学影像控制系统
DICOM	Digital Imaging and Communications in Medicine	医学数字成像和通讯

攻读学位期间公开发表的论文

Hui T, Chang ZB, Han F, Rui Y. Benign monomelic amyotrophy with lower limb involvement in an adult: A case report. Medicine (Baltimore). 2018. 97(23): e10774.

惠涛涛, 黎逢峰, 吴永伟, 刘军, 马运宏, 芮永军. 肋间动脉增压超长背阔肌皮瓣修复四肢创面. 中国修复重建外科杂志. 2018. 32(12): 1572-1575.

Hui T, Rui Y et al. Pulmonary embolism and lacunar infarction following open reduction-internal fixation of proximal humeral fractures. Injury. (Revise)

本研究得到以下基金资助

江苏省卫生计生委面上项目（H2017077）；无锡市临床医学中心建设对象（LCZXJS001）。

致 谢

不知不觉间毕业论文已经接近尾声。白驹过隙，转眼间三年硕士研究生学习生活即将结束。回首这三年在苏州大学附属无锡九院的点点滴滴，我心中思绪万千。在这三年里，既有过成功的喜悦，也有过失败的沮丧；既收获了成长，也经历了挫折。此时此刻，谨向所有关心和帮助过我的各位老师、同学、朋友和家人表示最诚挚的感谢，感谢你们出现在我的生命里，让我拥有，倍感珍惜。

首先感谢我的导师芮永军教授。能成为您的学生是我今生的荣耀，忘不了第一次师生相见，您和蔼的笑容化解了我内心的彷徨与不安。从我踏入医院的那一刻起，恩师精湛的医术，高尚的医德影响我至今。在我三年临床学习过程中，感谢恩师三年来对我的严格要求，告诫我虚心学习，指导我学习临床专业知识和操作技能，教会我作为一名临床医生，既要在临床技术上提高自己，同时更应该在医德上提升自己。在学术研究上，感谢恩师三年来对我的谆谆教导，当我对论文选题和思路感到迷茫时，是恩师给了我及时地指导和鼓励，使我打开了思路、充满了信心；当我由于懈怠、厌倦而疏于论文撰写时，是恩师给了我及时地提醒和鞭策。无论从论文选题、开题、定题、实验设计及实施，还是到论文内容的组织和篇章结构，每一个环节无不凝聚着恩师的汗水和心血，培养我严谨的科研态度和踏实的科研作风。恩师不仅授我以文，而且教我做人。恩师渊博的专业知识、严谨的治学态度，使我受益匪浅，这将是我终生学习的楷模和前进动力。路漫漫其修远兮，吾将上下而求索。在未来的学习生活和工作中，我将以恩师为榜样，除人类之病痛，助健康之完美，不忘初心，牢记使命，砥砺前行。

再次，感谢糜菁熠教授、许亚军教授、施海峰教授等专家在论文开题和答辩过程中提出的宝贵意见和给予的悉心指导。

感谢吴永伟、马运宏、刘军等老师以及创伤骨科全体医护人员在临床工作和学习生活中对我的指导和帮助。

感谢科教科吕彩霞科长、许凌云老师和王晶晶老师对我学习生活方面的帮助。

感谢手外科研究所潘筱云、毛栋老师在实验中耐心的指导和帮助，以及对我文章撰写过程中的帮助和指点。

特别感谢杨通、康永强、顾珺、徐鹏等所有师兄姐弟们的帮助。感谢你们为我课题的设计和操作提出许多建议，感谢你们在临床和生活上对我无微不至的关怀，真心的感谢你们这三年的鼓励与帮助。

衷心感谢百忙之中参加学位论文评阅和论文答辩的各位专家、教授。

感谢我的家人，感谢你们对我的理解，与我分享喜怒哀乐，陪我走过人生中重要的阶段。最后，在学位论文完成之际，再次向所有关心和帮助过我的老师、同学、朋友和家人表示最真挚的谢意！
