# Supplementary material for: Enhanced three-dimensional visualization reconstruction for perforator flaps: A case series on clinical applications and outcomes
Source: JPRAS Open. 2026 May 14;50:344–59. doi: 10.1016/j.jpra.2026.04.015 (PMC13240778; doi:10.1016/j.jpra.2026.04.015)
Supplement: Supplementary file 4 [file mmc4.pdf]

述　评

影响皮瓣成活的血管因素分析

魏在荣

作者单位:563003　贵州　遵义,遵义医学院附属医院 整形外科

作者简介:魏在荣(1974-),男,四川泸州人,主任医师,硕士研究生导师.

【关键词】 皮瓣;　血管解剖;　组织瓣;　蒂部

doi:10.3969/j.issn.1673-7040.2014.03.001

皮瓣是具有血液供应的包含皮肤的组织瓣,由蒂部和瓣部构成。蒂部是皮瓣转移或移植后的血供来源,可包含血管、皮肤、筋膜、肌肉、神经等多种组织,但必须包含供血血管;瓣部是皮瓣的主体,同样可包含皮肤、筋膜、神经、肌肉、肌腱、骨等,也必须包含供血血管。笔者仅探讨简单的皮瓣,即包含皮肤、筋膜、血管,部分包含神经。临床切取的皮瓣要完全成活,蒂部供给的血液必须能顺利地到达瓣部近端、中部、远端。若皮瓣的瓣部不能获得有效的血供,瓣部将出现部分甚至全部坏死。导致皮瓣坏死的原因较多,但主要还是皮瓣内血管的血供问题。深部的动脉血经过皮瓣蒂部血管、瓣部近端血管、瓣部中部血管,最后到达瓣部远端血管,营养整个皮瓣。如果深部动脉压恒定,皮瓣自身血管结构则是影响皮瓣成活的唯一因素。笔者仅对皮瓣获得良好血供的自身血管解剖因素作如下阐述。

1　皮瓣的发展历史

皮瓣外科的临床应用已有很长的历史,但发展缓慢,直至 20 世纪 70 年代,随着人们对皮瓣血供认识的逐步深入,其才得以迅速发展。1973 年,根据直接皮肤血管和肌皮血管的皮肤穿支血管在皮肤内的口径大小、走行方向和供血范围的不同,将皮瓣分为轴型皮瓣和随意型皮瓣,并在临床上广泛应用。1981 年,B Pontén报道并命名了筋膜皮瓣,此皮瓣包含深筋膜结构,且深筋膜血管网是对皮瓣成活有重要作用的一类皮瓣。该命名方式仅描述了皮瓣的解剖层次,没有描述皮瓣的血管解剖,因此,该命名方式在临床上的应用逐渐减少。1993 年,Bertelli 等首先提出了以肢体皮神经营养血管为供血基础的神经皮瓣,该皮瓣成活的主要血管是皮瓣内存在一条链状血管[1]。由于对该类皮瓣血供模式充分的认识,故在临床上广泛应用。

皮瓣被分为随意型皮瓣、轴型皮瓣和链状供血皮瓣,人们对皮瓣血供模式的认识逐渐清晰。随着人们对皮瓣生理解剖的进一步认识,笔者[2-3]将皮瓣内供血血管分为轴心血管、链状血管、网状血管;据此将皮瓣分为轴心血管供血皮瓣、网状血管供血皮瓣、链状血管供血皮瓣、混合血管供血皮瓣;该分类方式几乎囊括了目前所有切取的皮瓣,对皮瓣血供认识进一步清晰,临床应用逐渐增多,具有重要的指导意义[4-6]。

2　皮瓣的血管解剖结构分类

深部知名动脉在向远心端走行的同时,浅出分支营养周围组织,这些分支分为Ⅰ~Ⅳ级。Ⅰ级穿支[7]:骨穿支、肌腱穿支、肌肉穿支、神经穿支、皮肤穿支。Ⅰ级血管之间较少有直接联系。皮肤穿支进入深筋膜后分为Ⅱ级穿支[7]:上行支和下行支,两者再分出Ⅲ级分支进入浅筋膜,Ⅲ级穿支再分出Ⅳ级穿支进入真皮层,形成真皮下血管网。Ⅰ~Ⅳ级血管形成了穿支血管树,其供血范围称为解剖学供区[6]。据此皮瓣供血血管分类如下。

2.1　轴心血管　指人体深部知名血管系统,如桡动脉及伴行静脉、尺动脉及伴行静脉、腓动脉及伴行静脉、胫后动脉及伴行静脉、指固有动脉及伴行静脉等。

2.2　穿支血管　指由深部知名血管发出的无名、Ⅰ级分支血管系统,包括肌皮穿支、肌间隙穿支、肌间隔穿支、筋膜穿支等,属于特殊类型的轴心血管。

2.3　链状血管　Ⅱ级穿支在深筋膜层通过连续 2 个以上的真性吻合形成链状血管。链状血管常与皮神经

万方数据

伴行[1],但是也可在其他组织中被发现,特别是在肌肉和神经干内,也可在延迟皮瓣内发现[8-10]。上述链状血管供血的范围可以命名为链状血管体区,在此区,皮瓣切取后无血供障碍,不受长宽比例的限制。上述血管链常位于四肢、腹部,一般与深部轴型动脉走行一致,最典型的血管链位于小腿、前臂、手指,血管链常与皮神经伴行。小腿链状血管区有腓动脉走行区(腓肠外侧皮神经走行区)、胫后动脉走行区(隐神经走行区)、胫前动脉走行区(腓浅神经走行区)、腓肠神经走行区;前臂链状血管区有桡动脉走行区(前臂外侧皮神经走行区)、尺动脉走行区(前臂内侧皮神经走行区);手指链状血管区有指固有神经背侧支走行区等。根据皮神经走行切取的皮瓣即为链状血管供血皮瓣。有报道,在动物实验和临床上,通过延迟皮瓣的方式应用了标准的链状血管皮瓣-神经岛状皮瓣[11]。

2.4　网状血管　Ⅲ、Ⅳ级穿支血管共同构成了网状血管。Ⅲ级穿支在浅筋膜形成浅筋膜血管网,相邻Ⅲ级穿支间有真性吻合、阻塞性吻合及潜在吻合[12]。Ⅳ级穿支进入浅筋膜上、真皮下、真皮层,含有真皮层血管网、真皮下层血管网和/或皮下层血管网,这是穿支血管树的树冠。相邻Ⅳ级穿支血管间的真性吻合、阻塞性吻合及潜在吻合形成血管网。上述网状血管供血的皮瓣成为网状血管供血皮瓣[2],即指皮瓣蒂部、瓣部内无Ⅰ级穿支轴心血管,Ⅱ级穿支血管也较少(即使有真性吻合也仅有较短的链状血管)。胸骨前、胫骨前的皮肤缺乏皮下组织,皮下血管网没有直接吻合[13-14],网状血管对于切取的皮瓣变得尤为重要,这是最典型的网状血管供血皮瓣,皮瓣的长宽比例最好为 1.0∶1.0,否则皮瓣远端不能有效地灌注摄取足够营养,必然出现坏死。在其他非乏血管区,皮下组织发达,Ⅱ、Ⅲ级穿支血管较发达,有部分较短的链状血管,如背部[15],腹部[16],小腿内侧、后侧、外侧区[14],手部,前臂[14]等,这些部位切取的皮瓣长宽比例可达 1.5∶1.0,甚至更大。在上述区域切取网状供血皮瓣时,不能把长的血管链包含其中,否则皮瓣血供模式为链状血供,不再受长宽比例的限制。头面部皮下组织不是非常发达,但皮肤及皮下Ⅱ级穿支血管、Ⅲ级穿支血管、Ⅳ级穿支血管真性吻合或直接吻合大量存在,虽然没有形成较长的链状血管,但形成了连续的血管网络[1],即丰富的网状血管。切取皮瓣时其长宽比例可达 5.0∶1.0,皮瓣远端均无血供障碍。

3　皮瓣良好血供的条件

皮瓣切取完毕,无血管痉挛即可观察皮瓣血运。此时皮瓣血供决定因素为蒂部血管、瓣部血管是否通畅。皮瓣静脉回流与深部知名血管、Ⅰ级分支、Ⅱ级分支、Ⅲ级分支、Ⅳ级分支,或与蒂部血管、瓣部近端血管、瓣部中部血管、瓣部远端血管的方向相反,形成一血管环。而血管环的血流通畅,是皮瓣获得良好血供的必要条件。

轴心血管供血皮瓣的切取范围在轴心血管的解剖学供区,蒂部血供能通过瓣部轴心血管有效供应(即可形成通畅的血管环),皮瓣血运好。穿支血管供血皮瓣的切取范围在穿支血管的解剖学供区,蒂部血供能通过瓣部分支血管有效供应,皮瓣血运好。超出解剖血供区范围的皮瓣由潜在吻合血管提供血运[17],若继续扩大皮瓣范围将出现血供障碍。

链状血管供血皮瓣的瓣部由链状血管系统供血,纵贯瓣部主体;其供血范围相当于一个血管体区,同样可形成通畅的血管环,皮瓣切取后无血供障碍问题。这在皮瓣的三、四维解剖中已得到证实[18]。

网状血管供血皮瓣的瓣部不含轴心血管和链状血管,仅含有真皮层血管网、真皮下层血管网和/或皮下层血管网等。瓣部成活的范围与瓣部血管网血管之间吻合情况有关或与瓣部血管密度有关,吻合越多(血管密度越高),特别是真性吻合越多,皮瓣成活相对越长。通过大量血管吻合,同样可形成通畅的血管环。

混合血管供血皮瓣的瓣部血管组合方式有轴心血管-网状血管、轴心血管-链状血管-网状血管、链状血管-网状血管等。皮瓣的近端为轴心血管和链状血管(能形成通畅血管环),无供血障碍,只有到了网状供血部分才会出现血供障碍(血管环有可能中断)[19]。其切取要受血管吻合情况的影响,长宽比将受到限制。

综上所述,轴心血管供血皮瓣、链状血管供血皮瓣具有良好的供血血管,无血供障碍。网状血管供血皮瓣在超出大量网状吻合血管范围后,将由大量的潜在吻合血管替代,不能很好地形成血管环,皮瓣远端部分坏死是必然的。因此,深部血管通畅、蒂部充足的血供、瓣部良好的吻合血管,才能对皮瓣的瓣部近端、中部、远端提供有效供血,切取的皮瓣才能完全成活。

知晓影响皮瓣成活的血管因素,其目的是帮助临床医师更好地在临床中应用皮瓣。而皮瓣的设计是皮瓣应用的首要步骤。皮瓣的设计应遵循:①首先判断皮瓣供区深部血管是否通畅、其Ⅰ~Ⅳ级穿支的情况[20];②根

万方数据

据供瓣区解剖学特征设计皮瓣蒂部、瓣部的外形和范围,解剖学特征包括是否有链状血管、血管密度高低、血管吻合情况等;③根据受区范围调整皮瓣瓣部范围,进一步判断该供瓣区是否适合修复受区缺损。

参考文献:

[1] Taylor GI, Corlett RJ, Dhar SC, et al. The anatomical (angiosome) and clinical territories of cutaneous perforating arteries: development of the concept and designing safe flaps[J]. Plast Reconstr Surg, 2011,127(4):1447-1459.

[2] 魏在荣. 皮瓣命名方式探讨[J]. 中华损伤与修复杂志(电子版), 2011,6(3):337-341.

[3] 魏在荣. 皮瓣血供模式分类方式初步报告[J]. 遵义医学院学报, 2012,35(4):344-346.

[4] 魏在荣, 孙广峰, 唐修俊, 等. 腘窝中间动脉蒂混合供血皮瓣修复儿童腘窝烧伤瘢痕挛缩[J]. 中国修复重建外科杂志, 2012,26(8): 946-949.

[5] 吴必华, 魏在荣, 唐修俊, 等. 胫后动脉穿支蒂混合供血皮瓣修复儿童小腿创面[J]. 中华小儿外科杂志, 2013,34(5):349-352.

[6] Taylor GI, Ives A, Dhar S. Vascular territories. Plastic surgery[M]. Philadelphia: Saunders, 2006:317-364.

[7] Wei ZR, Sun GF, Wang DL, et al. Reconstruction of the achilles tendon and overlying skin defect: 3 case reports[J]. Ann Plast Surg, 2013 Jul 11.

[8] Taylor GI, Corlett RJ, Caddy CM, et al. An anatomic review of the delay phenomenon: Ⅱ Clinical applications[J]. Plast Reconstr Surg, 1992, 89(3): 408-416; discussion 417-408.

[9] Morris SF, Taylor GI. Predicting the survival of experimental skin flaps with a knowledge of the vascular architecture[J]. Plast Reconstr Surg, 1993,92(7):1352-1361.

[10] Dhar SC, Taylor GI. The delay phenomenon: the story unfolds[J]. Plast Reconstr Surg, 1999,104(7):2079-2091.

[11] Sönmez E, Ozdemir H, Safak T, et al. A modification of the neural-island flap 'split neural-island flap'[J]. J Plast Reconstr Aesthet Surg, 2009,62(1):85-92.

[12] Zhuang Y, Hu S, Wu D, et al. A novel in vivo technique for observations of choke vessels in a rat skin flap model[J]. Plast Reconstr Surg, 2012,130(2):308-317.

[13] Saint-Cyr M, Wong C, Schaverien M, et al. The perforasome theory: vascular anatomy and clinical implications[J]. Plast Reconstr Surg, 2009, 124(5):1529-1544.

[14] Lecours C, Saint-Cyr M, Wong C, et al. Freestyle pedicle perforator flaps: clinical results and vascular anatomy[J]. Plast Reconstr Surg, 2010, 126(5):1589-1603.

[15] Minabe T, Harii K. Dorsal intercostal artery perforator flap: anatomical study and clinical applications[J]. Plast Reconstr Surg, 2007,120(3): 681-689.

[16] Schaverien M, Saint-Cyr M, Arbique G, et al. Arterial and venous anatomies of the deep inferior epigastric perforator and superficial inferior epigastric artery flaps[J]. Plast Reconstr Surg, 2008,121(6):1909-1919.

[17] 陈世新, 吴东方, 丁茂超, 等. 穿支体区血管及其相互间吻合的3D可视化研究[J]. 中国临床解剖学杂志, 2011,29(3):237-242.

[18] Wong C, Saint-Cyr M, Mojallal A, et al. Perforasomes of the DIEP flap: vascular anatomy of the lateral versus medial row perforators and clinical implications[J]. Plast Reconstr Surg, 2010,125(3):772-782.

[19] Villa M, Saint-Cyr M, Wong C, et al. Extended vertical rectus abdominis myocutaneous flap for pelvic reconstruction: three-dimensional and four-dimensional computed tomography angiographic perfusion study and clinical outcome analysis[J]. Plast Reconstr Surg, 2011,127(1):200-209.

[20] Qassemyar Q, Havet E, Sinna R. Vascular basis of the facial artery perforator flap: analysis of 101 perforator territories[J]. Plast Reconstr Surg, 2012,129(2):421-429.

(收稿日期:2013-11-15)

读者·作者·编者

参考文献中英文作者名的著录方法

在医学期刊的论文中,引用英文文献的比例很高,但有不少作者将英、美人的姓名搞错,以至检索核对时出现错姓、错名或姓名全错。英、美人姓名的习惯写法是:名-名-姓,“名”可以有 1 个、2 个或 3 个,但“姓”只有 1 个。因此,从书籍或期刊中的姓名转录到文献时,要将次序调整为姓-名-名。“姓”是不可以简写的;“名”可以缩写,用第一个字母大写,不用缩写点。数据库在著录作者姓名时,已经调整为“姓-名-名”,可照录。

例如:John Quincy Public 写为 Public JQ。

万方数据
