# Supplementary material for: Enhanced three-dimensional visualization reconstruction for perforator flaps: A case series on clinical applications and outcomes
Source: JPRAS Open. 2026 May 14;50:344–59. doi: 10.1016/j.jpra.2026.04.015 (PMC13240778; doi:10.1016/j.jpra.2026.04.015)
Supplement: Supplementary file 5 [file mmc5.pdf]

解放军医学院
解放军总医院

硕士学位论文

(C)1994-2022 China Academic Journal Electronic Publishing House. All rights reserved. http://www.cnki.net

分类号：R622+.9 密　级：公开

基于核磁共振成像的数字可视化技术在整形外科常见体表肿瘤、穿支皮瓣手术中的应用

The application of digital visualization technology based on MRI in superficial tumor excision and perforator flap operations in plastic surgery

作者姓名：栗　利

学科专业：整形外科

导　　师：韩　岩　教授

答辩委员会主席：祁佐良

论文答辩日期：二O一九年五月二十三日

院校地址：北京市复兴路28号

邮政编码：100853

(C)1994-2022 China Academic Journal Electronic Publishing House. All rights reserved. http://www.cnki.net

解放军医学院
研究生学位论文原创性声明

秉承我院“忠诚、敬业、和谐、创新”的学风，本人声明：所呈交的论文是我本人在导师指导下进行的研究工作及取得的研究成果。据我所知，除了文中特别加以标注和致谢的地方外，论文中不包含任何其他个人或集体已经发表或撰写过的研究成果，也不含为获得或其他教育机构的学位及证书而使用过的材料，对本文的研究作出贡献的个人或集体，均已在文中做了明确的说明并表示谢意。

申请学位论文与资料若有不实之处，本人承担一切相关责任。

论文作者签名：栗利　　日期：2019.5.23

指导教师签名：郭亮　　日期：2019.5.23

解放军医学院
研究生学位论文版权使用授权书

本人保证毕业离院后，发表论文或使用论文工作成果时署名单位为，有权保留并向国家有关部门或机构送交论文原件、复印件和电子版本，可以采用影印、缩印、扫描或其它手段保存论文以供被查阅和借阅。可以公布学位论文的全部或部分内容（保密内容除外）。

论文作者签名：栗利　　日期：2019.5.23

指导教师签名：郭亮　　日期：2019.5.23

(C)1994-2022 China Academic Journal Electronic Publishing House. All rights reserved.　http://www.cnki.net

目　　录

英文缩略词表......1
中文摘要......3
英文摘要......5
前　言......7
第一部分 3D 打印肿瘤与肿瘤切除导板在常见体表肿瘤手术中的应用......11
1 引言......11
2 材料与方法......11
3 结果......13
4 典型病例......14
5 讨论......20
6 结论......23
第二部分 肿瘤三维投影与“肿瘤地图”投影在常见体表肿瘤手术中的应用......25
1 引言......25
2 材料与方法......25
3 结果......26
4 典型病例......27
5 讨论......29
6 结论......31
第三部分 基于 MRA 成像的“穿支血管地图”在整形外科皮瓣手术中的应用......32
1 引言......32
2 材料与方法......32
3 结果......33
4 典型病例......34

(C)1994-2022 China Academic Journal Electronic Publishing House. All rights reserved. http://www.cnki.net

5 讨论......36
6 结论......39
总 结......40
参考文献......42
文献综述......44
攻读学位期间发表文章情况......49
致 谢......50

(C)1994-2022 China Academic Journal Electronic Publishing House. All rights reserved. http://www.cnki.net

英文缩略词表

英文缩写	英文全称	中文全称
MRI	Magnetic Resonance Imaging	核磁共振
MRA	Magnetic Resonance Angiography	核磁共振血管成像
CTA	Computed Tomography Angiography	计算机断层扫描血管造影
MM	Malignant Melanoma	恶性黑色素瘤
SCC	Squamous Cell Carcinoma	鳞癌
BCC	Basal Cell Carcinoma	基底细胞癌
DFSP	Dermatofibrosarcoma Protuberans	皮肤隆突性纤维肉瘤
WLE	Wider Local Excision	扩大切除法
MMS	Mohs Micrographic Sugery	莫氏手术法
AR	Augmented Reality	增强现实
VR	Virtual Reality	混合现实
DICOM	Digital Imaging and Communications in Medicine	医学数字影像和通讯
LAVA	Liver Accelerated Volume Acquisition	肝脏容积加速采集
LDM	Localization with Distance Measurement	测量法
LIP	Localization with Image Projection	投影法
LPPF	Localization with Printed Plastic Film	打印法
DIEP	Deep Inferior Epigastric Perforator	腹壁下动脉穿支
DIEA	Deep Inferior Epigastric Artery	腹壁下动脉

(C)1994-2022 China Academic Journal Electronic Publishing House. All rights reserved. http://www.cnki.net

作　　者：栗利
学科专业：外科学（整形外科学）
导　　师：韩岩

基于核磁共振成像的数字可视化技术在整形外科常见体表肿瘤、穿支皮瓣手术中的应用

中文摘要

研究目的

探索数字可视化技术在整形外科领域的应用，具体指通过多种数字化手段将核磁共振成像数据进行转化和可视化应用，指导整形外科常见体表肿瘤的精确化切除和穿支皮瓣手术的精确化设计。

研究方法

研究对象来自于整形外科 2017 年 1 月-2018 年 12 月间收治的患者，患者因体表肿瘤需经手术切除，或因创面需行穿支皮瓣手术修复，按照相应要求选择入组。对患者行高分辨率核磁共振扫描，数据导入后处理软件进行三维重建和三维设计。而后：

1、对体表肿瘤患者，构建体表肿瘤数字模型，设计个性化肿瘤切除导板，以 3D 打印方式成模，指导手术。

2、对体表肿瘤患者，设计个性化“肿瘤地图”，以便携式投影仪将肿瘤三维模型和“肿瘤地图”投影与患者体表，指导手术。

3、对需行穿支皮瓣手术患者，分析穿支血管，设计个性化“穿支血管地图”，以测量法、投影法、打印法分别定位于患者体表，指导手术。

研究结果

1、各病例均可通过三维重建获得肿瘤数字模型，设计出个性化肿瘤切除导板，并通过 3D 打印成模，对手术有指导价值；病理结果提示基于核磁共振成像的 3D 打印肿瘤切除导板指导体表肿瘤切除手术的精确度为 100%。

2、各病例均可设计出个性化“肿瘤地图”，将肿瘤模型和“肿瘤地图”投影于患者体表，对手术有指导价值。

3、MRA 可显示部分穿支血管，平均每例 4.5±2.1 支；各病例均可设计出个性化“穿

(C)1994-2022 China Academic Journal Electronic Publishing House. All rights reserved.　http://www.cnki.net

支血管地图”，并通过三种方法定位于体表，辅助术者选择最佳穿支设计手术。

研究结论

1、3D 薄层 MRI 序列可对体表肿瘤清晰成像，满足三维重建需求。

2、对体表肿瘤患者，3D 打印肿瘤模型有助于术者准确了解肿瘤形态。肿瘤三维模型投影有助于术者准确了解肿瘤范围。

3、对病情较复杂的体表肿瘤患者，我们推荐使用“肿瘤切除导板法”，临床思路为：“MRI 扫描→设计个性化肿瘤切除导板→3D 打印→手术应用”；或推荐使用“肿瘤地图投影法”，临床思路为：“MRI 扫描→设计个性化’肿瘤地图’→体表投影→手术应用“。以此提高体表肿瘤切除的精确度。

4、高分辨率 MRA 可显示部分穿支血管，在穿支皮瓣手术中有较强的应用价值。

5、对病情较复杂的拟行穿支皮瓣手术患者，我们推荐使用“穿支血管地图法”，临床思路为:“MRI 扫描→设计个性化’穿支血管地图’→体表定位（测量法、打印法、投影法）→手术应用”，可有助于术者设计最佳穿支皮瓣。

关键词 体表肿瘤、穿支皮瓣、MRI/MRA、3D 打印、投影、数字化技术、可视化、肿瘤切除导板、肿瘤地图、穿支血管地图

(C)1994-2022 China Academic Journal Electronic Publishing House. All rights reserved. http://www.cnki.net

The application of digital visualization technology based on MRI in superficial tumor excision and perforator flap operations in plastic surgery

Abstract

Objective

To explore the application of digital visualization technology in the field of plastic surgery. Specifically, to study a variety of digital visualization means to apply the MRI data to guide the precise resection of common superficial tumors and the precise design of perforator flap operations in plastic surgery.

Methods

The subjects of this study were from the patients admitted to the plastic surgery department of the PLA general hospital from January 2017 to December 2018. The patients were in need of surgical resection for superficial tumors or surgical repair with perforator flaps for large wounds. They were enrolled according to the corresponding requirements. All the patients underwent high-resolution MRI scan, and the data were imported into the post-processing software for 3D reconstruction and model designing. Afterwards:

1. For patients with superficial tumors, a digital tumor model was constructed and a personalized guide plate for tumor resection was designed. They were molded by 3D printing and used to guide the surgery.

2. For patients with superficial tumors, a personalized "tumor map" was designed and a portable projector was used to project the three-dimensional tumor model and "tumor map" onto the patient's body surface to guide the surgery.

3. For patients in need of perforator flap surgery, the perforator arteries were analyzed, and a personalized "perforator map" was designed. Three different methods were used to locate the "perforator map" onto the patient's body surface to guide surgery including: LDM （Localization with Distance Measurement）、LIP（Localization with Image Projection）and LPPF（Localization with Printed Plastic Film）.

Results

1. For every case, a digital tumor model can be obtained via 3D reconstruction with MRI data and a personalized guide plate for tumor resection can be designed accordingly. The models can be molded by 3D printing and showed significant values in guiding surgery. The pathological results suggested that the accuracy of 3D-printed guide plate for

(C)1994-2022 China Academic Journal Electronic Publishing House. All rights reserved. http://www.cnki.net

tumor resection based on MRI in guiding superficial tumor resection was 100%.

2. For every case, a personalized "tumor map" can be designed. The digital tumor model and "tumor map" can be projected onto the patient's body surface, which is of guiding value for surgery.

3. MRA can display partial perforator arteries, with an average number of 4.5±2.1 per case. For every case, a personalized "perforator map" can be designed. By means of three methods (LDM、LIP and LPPF) it can be located onto the patients' body surface accurately to assist the operator to select the best perforator for surgery.

Conclusions

1. 3D thin layer MRI sequence can clearly image superficial tumors and meet the requirements of 3D reconstruction.

2. For patients with superficial tumors, a 3D-printed tumor model is helpful for surgeons to accurately understand the tumor morphology and the projection of 3D tumor model onto the body surface is helpful for surgeons to accurately determine the tumor range.

3. For patients with complex superficial tumors, we recommend the use of " guide plate for tumor resection method". The clinical process is: "MRI scan → Design personalized guide plate for tumor resection → 3D printing → Surgical application ". Besides, we recommend the use of "tumor map projection method". The clinical process is: "MRI scan → Design personalized" tumor map "→ Body surface projection → Surgical application". In this way, the accuracy of tumor resection can be improved.

4. High resolution MRA can display part of the perforators, which is of unique value in perforator flap surgery.

5. For patients with complicated conditions that are requiring perforator flap surgery, we recommend the use of "perforator map method". The clinical process is: "MRI scan→ Design personalized 'perforator map' → Body surface localization (LDM、LIP and LPPF) → Surgical application", which is helpful for surgeons to make the best perforator flap designing.

Keywords superficial tumor, perforator flap, MRI/MRA, 3D printing, projection, digital technology, visualization technology, guide plate for tumor resection, tumor map, perforator map

(C)1994-2022 China Academic Journal Electronic Publishing House. All rights reserved. http://www.cnki.net

前 言

一、研究概述

在整形外科领域，体表肿瘤切除手术和穿支皮瓣修复手术是两种传统而常见的手术，是临床工作的重要内容。对整形外科医师而言，一方面要尽量切净肿瘤，避免瘤体残留，一方面又要尽量保护正常组织，减小创面面积，尤其当肿瘤位于面部等重要区域时，组织可谓“寸土寸金”，较大的组织缺损修复后对美观影响较大。因此如何在尽量保存正常组织的同时切净肿瘤是整形外科医生面临的重要问题，具有较大的临床意义[1]。部分体表肿瘤手术后易反复复发，或因就医不及时致肿瘤巨大，如何处理好这种棘手的复杂病例更是对外科医生的一种考验。

当今科技迅猛发展，技术革新和融合创新已成为推动学科进步的重要力量。我们课题组结合影像、病理等学科，尝试将数字化技术应用于整形外科的体表肿瘤切除手术和穿支皮瓣修复手术，以解决工作中遇到的难题，提高手术的精确程度和可视程度。

本课题研究分为两个方向共三个部分，其中第一、二部分为“基于 MRI 的数字化技术指导体表肿瘤切除”方向，第三部分为“基于 MRI 的数字化技术指导穿支皮瓣修复”方向。二者均以 MRI 数据为基础，通过后处理软件进行三维重建和相应设计，以 3D/平面打印、三维/平面投影方式应用于手术。研究初步展现了较强的临床应用价值，并为下一步深入研究奠定了基础。

二、体表肿瘤切除相关研究

（一）研究背景

体表肿瘤包括皮肤肿瘤和软组织肿瘤。皮肤肿瘤是指原发于皮肤或其附属器或转移至皮肤的肿瘤，有良性、恶性之分，常见良性皮肤肿瘤包括色素痣、血管瘤等，恶性皮肤肿瘤包括恶性黑色素瘤（MM）、鳞癌（SCC）、基底细胞癌（BCC）、皮肤隆突性纤维肉瘤（DFSP）等[2]。软组织肿瘤指起源于间叶组织位于软组织内的肿瘤，如起源于脂肪、肌肉、血管、纤维等，亦有良性、恶性之分，良性的称为瘤，如脂肪瘤、神经纤维瘤，恶性的称为肉瘤，如横纹肌肉瘤、恶性纤维组织细胞瘤等。体表肿瘤是整形外科的常见病种，通常需要以手术治疗为主，切除肿物，创面行拉拢缝合、局部/游离皮瓣修复、植皮等手段修复。

（二）部分体表恶性肿瘤难以切净与其形态特性、生长特点密切相关

部分体表恶性肿瘤难以切净，手术后容易复发，这与多种因素有关，如肿瘤病理

(C)1994-2022 China Academic Journal Electronic Publishing House. All rights reserved. http://www.cnki.net

类型、形态特性、生长特点等。例如，皮肤隆突性纤维肉瘤[3,4]，瘤体可发出触手样突起，瘤体在皮下延伸至肿瘤中心较远的地方，肿瘤呈现“外小内大”的形态；另外，恶性黑色素细胞瘤呈现浸润性生长，其多发于表皮、真皮交界处，随病情发展肿瘤向深层浸润，呈现“冰山样生长”，浸润深度是判断分期和愈后的重要指标，也是临床手术需重点关注的因素；另外，头皮鳞癌、头皮转移癌等体表肿瘤容易沿着帽状腱膜下、筋膜下的间隙扩散生长，手术时应充分考虑是否有局部扩散。

（三）体表肿瘤常见手术方法

目前临床常用的手术方法有两种：扩大切除法和莫氏手术法[2]。

1、扩大切除法（WLE, Wide Local Excision）

在肿瘤肉眼边界之上扩大一定范围进行切除，术中取边界组织行冰冻切片确定边缘是否切净。其优点是简单易行，临床应用广泛。其缺点包括：①扩大范围仅为经验性指导。临床医师通常根据肿瘤病理和分级确定扩大范围，最新版的 NCCN Guideline（Version2019.2）建议扩大切除范围为：BCC 4mm，SCC 4-6mm，DFSP 20mm。临床遇到的体表肿瘤即使为同一病理型，其大小、位置、浸润情况等各不相同，其相应的扩大范围应当也不尽相同，因此仅根据仅病理型确定扩大切除范围有失准确。②肉眼边界不可靠。部分体表肿瘤呈现“冰山式”生长，瘤体的一部分外露于体表，其余部分则位于皮下，如 DFSP 呈“触手样”生长，触手可在皮下延伸到距离瘤体中央很远的地方，因此仅凭肉眼判断边界并不准确。另外瘤体向各方向的生长可能受局部组织疏松程度影响，并非呈现绝对的球形，若始终以默认的扩大范围做圆形切口设计，必然造成误差，理想状态应以肿瘤实际形态做个性化的扩大范围设计。③术中冰冻为概率性检测。术中冰冻作为一项快捷有效的检查方式，对肿瘤切净具有重要意义，已广泛应用于外科各专业的肿瘤手术中，但切取时取点有限，通常取上、下、左、右、基底五处，其他方向的切缘是否干净无从保证，尤其对较大的肿瘤，单纯的五点取材漏诊率显然更高，可见术中取点冰冻是一种概率性检测。此外病理医师在制冰冻片时要对标本进一步再切取，更增加了假阴性率。

2、莫氏手术法（MMS，Mohs Micrographic Surgery）

该技术源自二十世纪三十年代，由法国外科医生 Frederic Mohs 最早提出，也因此而得名。通过“切取切缘-送检冰冻-阳性者再次切取”的反复步骤，对全部切缘行冰冻病理检测，从而保证切缘的绝对阴性，同时最大限度的减少损伤正常组织[5]。外科

(C)1994-2022 China Academic Journal Electronic Publishing House. All rights reserved. http://www.cnki.net

医生在操作时通常将切缘分为不同区域进行标记，同时在纸上画出相应图示，一一对应。通常以 45 度角度斜行取材，将其压平后制取冰冻切片。莫氏手术的优点很明显——可在完整切除肿瘤的同时最大限度地保护正常组织，因此是一种十分理想的手术方式[6]。但其缺点也不容忽视：①手术对病理医师的依赖程度很高，术中需要进行大量的冰冻切片取材、制片、读片，需要病理医师的紧密配合，因此国外开展莫氏手术较多的医院通常有专门的病理医师配合术者，病理室甚至直接设于手术室旁，而在国内三甲综合医院，病理科的日常工作量已经很大，很难满足莫氏手术的需求，限制了其开展。②手术时间较长，增加了手术成本，限制了术者进行手术的数量，同时较长时间的等待也增加了老年患者的手术风险。③仅适用于体积较小的肿瘤，对较大的肿瘤，尤其是复杂体表肿瘤或复发性体表肿瘤，行莫氏手术极其费时费力，临床应用的可行性极低。

（四）课题研究思路

上述两种方法各有利弊，临床以“扩大切除法”使用较多。临床医生手术多以体表肿瘤的肉眼手感边界为参考设计扩大的肿瘤切除范围，误差较大，最理想的方法是以肿瘤病理边界为参考设计肿瘤切除范围，但以目前技术在术前获得其病理学边界是不可能的。影像学边界较之于肉眼手感边界无疑更接近真实的病理学边界，我课题组提出“以肿瘤的影像学边界为参考设计手术切除范围”的理念，提出肿瘤切除的新思路——“数字化技术指导的下体表肿瘤精确切除法”，具体包括“肿瘤模型和肿瘤切除导板法“、“肿瘤投影和‘肿瘤地图’投影法”。

具体思路为：使用数字化技术，将肿瘤的影像学数据进行三维重建，通过肿瘤切除导板和“肿瘤地图”投影，于患者体表精确定位出肿瘤的影像学边界，在此基础上行扩大切除，提高了肿瘤切除的精确程度。该方法尤其适用于手术难度较大的复发型和复杂型体表肿瘤，能为术者提供丰富的信息支持，大大提高手术的可行性，提高手术精确度，具有很强的临床应用价值。

三、穿支皮瓣修复相关研究

（一）研究背景

1、穿支皮瓣的概念及其探查方法

穿支皮瓣是当前整形外科的研究热点。穿支血管是指直径大于 0.5mm 的小血管分支。对整形外科医生而言，穿支血管的出现使皮瓣手术更加灵活和个性化，使皮瓣

(C)1994-2022 China Academic Journal Electronic Publishing House. All rights reserved. http://www.cnki.net

设计进入"自由时代 "。对穿支皮瓣手术而言，确定优质的穿支血管是手术成功的关键。

当前寻找合适穿支的方法包括超声（便携式多普勒、彩色多普勒）、CTA、MRA、DSA、吲哚氰绿造影等。其中 CTA 普遍被认为是当前穿支血管成像的首选，临床已经得到广泛应用。其优点是清晰度高，扫描速度快，数据可以进行后处理重建形成三维图像。其缺点是病人要承受辐射，需要注射造影剂，存在过敏可能，不适合肾功能较差者，且对操作者有一定技术要求，需要设定特定的扫描时间方能清晰显示小血管。

2、穿支血管的分布特点

人体的主干血管通常恒定，而穿支血管变异较大。此外，在某一区域可能有多支穿支血管可供使用。术者最终选择何种方案往往依据所掌握的解剖知识和个人手术经验，初步判断创面附近某处可能存在穿支血管，借助超声、CT、MRI 等辅助手段对该兴趣区进行检查，如为阳性即可考虑选择该穿支设计皮瓣。其信息来源相对局限，无法保证所选穿支就是最佳方案。因此全面地、个性化地了解患者穿支血分布管情况十分必要。

（二）研究思路

近年来国外学者对 MRA 显示穿支血管的研究逐渐增多，国内尚未见相关研究报道。我课题组使用 MRA 对穿支血管进行研究，初步取得了一定研究成果。我们提出"穿支血管地图"的概念，建议对较复杂的拟行穿支皮瓣手术的患者使用"基于 MRI 的穿支血管地图定位法"。术前对患者术区附近进行全面扫描，将各穿支血管三维重建，穿出点于体表标注，并测量其直径、穿出肌肉点到体表距离等数值，从垂直于冠状位前方截图即可得到一个个性化的、全面的、直观的"穿支血管地图"，为术者提供决策依据。

(C)1994-2022 China Academic Journal Electronic Publishing House. All rights reserved. http://www.cnki.net

第一部分 3D 打印肿瘤与肿瘤切除导板在常见体表肿瘤手术中的应用

1 引言

本研究对适合体表肿瘤成像及三维重建的 MRI 序列进行了初步探索。通过 3D 打印肿瘤模型帮助术者准确了解体表肿瘤的肿瘤形态。在传统扩大切除法的基础上提出了“基于 MRI 的肿瘤切除导板指导体表肿瘤切除”的新方法。对病情较复杂的体表肿瘤患者，我们推荐的临床思路为:“MRI 扫描→设计个性化肿瘤切除导板→3D 打印→手术应用”。

2 材料与方法

2.1 研究对象

研究范围为解放军总医院整形外科 2017 年 1 月-2018 年 12 月收治的体表肿瘤病人，从中选择合适的对象进行研究。入组标准包括：①瘤体较大（直径>3cm）需行外科手术切除；②肿瘤可为初发或复发；③未见远处转移；④患者可配合进行体表肿瘤高分辨率 MRI 扫描，平均时间约半小时。其中进行 MRI 扫描的具体要求包括：①无增强造影剂如钆剂过敏；②肾功能正常（血肌酐：男 54-106umol/L，女 44-97umol/L）；③无明显幽闭恐惧症。排除标准为：①恶性黑色素细胞瘤。因其生长特性独特，早期易转移，单纯手术往往效果不佳，本研究暂不予纳入。②因各种原因无法行增强 MRI 扫描者。所有患者在进行研究前均充分告知，征得同意，研究课题立项时已通过医院伦理委员会审查备案。课题先后纳入病例 17 例，其中设计较好、数据完备的病例 5 例。

2.2 进行体表肿瘤 MRI 扫描

2.2.1 扫描体位

体位因瘤体位置而异，通常取与术中相同体位，保持瘤体面向上。如肿瘤位于头部、胸部、腹部取仰卧位，如位于背部、臀部取俯卧位。

2.2.2 扫描序列

T1、T2 加权序列平扫，增强后 T1 加权序列；根据部位选择薄层 3D 序列：3D-SPACE、3D-Cube、3D-LAVA；

2.2.3 扫描参数

采用 GE 3.0T DISCOVERY MR750 核磁共振机，配有 8 通道相控阵线圈，使用阵

(C)1994-2022 China Academic Journal Electronic Publishing House. All rights reserved. http://www.cnki.net

列空间敏感编码技术（Array spatial sensitivity encoding technique, ASSET），设定层厚2mm，层间距-1mm，相位加速因子 2Ph，TR 3ms，TE 1.3ms，FOV 40x32，矩阵 270x160，空间分辨率为 1.5x2x1mm，反转角 12°，接收带宽 125kHz。

2.3 三维重建瘤体，设计肿瘤导板

将所得 Dicom 数据导入影像重建软件，常用 Mimics、3Dslicer、AW4.6。选择瘤体清晰显示的薄层 3D 序列，设定阈值对肿瘤区域进行自动提取，或通过手动描记添加提取，获得肿瘤三维数据。可根据需要向瘤体周围扩大一定距离设计肿瘤扩大切除模型，如皮肤隆突性纤维肉瘤可扩大 2cm。于肿瘤边缘向体表做垂直投影，得到肿瘤的最大体表投影，即为肿瘤切除导板模型，可认为影像所示瘤体均位于该投影范围内。根据部位不同可在导板上添加设计相应卡槽，辅助导板准确固定于体表，如头部肿瘤导板设计经双测耳上卡槽，腹部肿瘤导板设计经腹壁外侧缘的卡槽。在同一坐标系内同时提取正常组织、骨骼、血管等数据。设定坐标系方便后续测量及定位。将各数据均以 STL 格式导出。

2.4 3D 打印模型

将 STL 数据导入打印机，具体扫描参数见下，通常选择聚光敏树脂材料。瘤体切除模型可选择透明材料，更清晰显示瘤体部分。根据需要对模型各部分上色。通常将瘤体涂为黄色，标记物涂为绿色，动脉涂为红色。

表 1 光固化激光 3D 机扫描参数（RS6000，上海联泰）

脉冲频率	30-100KHz
激光功率	1000mW
激光波长	355nm
光斑直径	0.12-0.20mm
典型扫描速度	6-10m/s
成型范围	600×600×400mm
分层厚度	0.05-0.25mm
成型材料	光敏树脂
文件格式	STL
外形尺寸	1460×1250×1900（mm）
重量	1400KG

(C)1994-2022 China Academic Journal Electronic Publishing House. All rights reserved.   http://www.cnki.net

2.5 手术应用

2.5.1 应用肿瘤模型

术者通过分析肿瘤模型可准确了解瘤体形态特点，包括肿瘤大小、在体位置、层次深度、各切面形态等，优化手术设计，避免单纯依据肿瘤的肉眼手感边界通过视诊、触诊行经验性扩大切除带来的肿瘤漏切。

2.5.2 应用肿瘤切除导板

沿瘤体边界做垂直于皮肤的垂线，与皮肤相交点为肿瘤影像学边界的体表投影，提取其内部部分即形成肿瘤导板模型。将肿瘤导板通过设计的卡槽固定于体表，导板边缘即为瘤体边缘，以记号笔标记于体表。以此瘤体边缘线为参考，设计肿瘤扩大切除范围。通常基底细胞癌取 4mm，鳞癌 4-6mm，皮肤隆突性纤维肉瘤取 20mm。

2.6 病理-大体-影像边界比对

将完整切除的标本进行边缘大病理检测。取材点分布在瘤体周缘各个方位，具体数目依据肿瘤大小而定。纵向取材，取材时应当横跨肿瘤肉眼切缘，包含过渡地带。以石蜡包埋，HE 染色。经数字病理切片扫描仪扫描，导入阅片软件分析。比较肿瘤的实际病理边缘、肉眼边界、肿瘤导板切缘（即影像边界）之间的差距。各工具设备参数见下表。课题目前共对 17 处切缘进行了病理检测，纳入统计范围。

表 2 大病理切片研究所需主要工具耗材

工具设备	规格型号	厂家
全自动脱水机	Leica	Leica Biosystems，德国
病理组织包埋机	Leica	Leica Biosystems，德国
大病理切片机	Leica SM2000R	Leica Biosystems，德国
特制大载玻片	8×13cm	飞舟玻料有限公司，江苏
特制大盖玻片	7×12cm	飞舟玻料有限公司，江苏
全自动数字病理切片扫描仪	KF-PRO-120	江丰生物信息技术有限公司，浙江
数字切片阅片软件	K-ViEWER	江丰生物信息技术有限公司，浙江

3 结果

（1）各病例体表肿瘤均可在高分辨率 MRI 下清晰显像，图像质量满足三维重建需求。

（2）各病例均可通过三维重建提取肿瘤数字模型，并设计出个性化肿瘤切除导

(C)1994-2022 China Academic Journal Electronic Publishing House. All rights reserved. http://www.cnki.net

板。

（3）各病例的肿瘤数字模型和肿瘤切除导板均经可 3D 打印成模，帮助术者充分了解肿瘤形态，辅助精确设计肿瘤扩大切除切口。

（4）术后病理检测显示：肿瘤实际病理边界位于肿瘤导板切缘内侧的比例为 100%。提示基于 MRI 的 3D 打印肿瘤切除导板指导体表肿瘤切除手术的精确度为 100%。

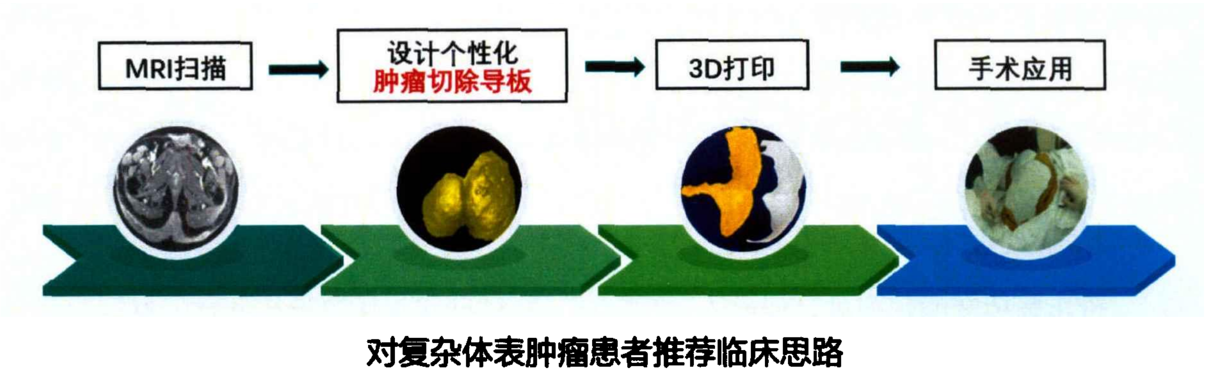

图 1 对复杂体表肿瘤患者推荐临床思路

4 典型病例

4.1 头顶部复发性鳞癌。

患者女性，38 岁，因头顶部肿物 3 月余入院，3 年前因头部鳞癌手术，本次入院考虑为“头顶部鳞癌复发”。入院后行头部核磁扫描，提取肿瘤三维模型，同时设计扩大 2cm 切口。将模型进行 3D 打印。应用模型指导手术范围设计。手术切除肿瘤后创面行游离背阔肌皮瓣修复。

意义：通过对肿瘤的核磁数据进行三维重建得到瘤体的三维模型，能够准确了解其形态特征。该肿瘤瘤体体表外部分虽呈现球形，但其皮下部分呈现不对称性，瘤体向左侧颞部有较多延伸。如行常规扩大 2cm 切除，容易漏切部分瘤体，盲目性较大。可见通过 3D 打印肿瘤模型的方法可以精确了解肿瘤形态，在此基础上行扩大切除设计，可以提高肿瘤切除的精确性。

(C)1994-2022 China Academic Journal Electronic Publishing House. All rights reserved. http://www.cnki.net

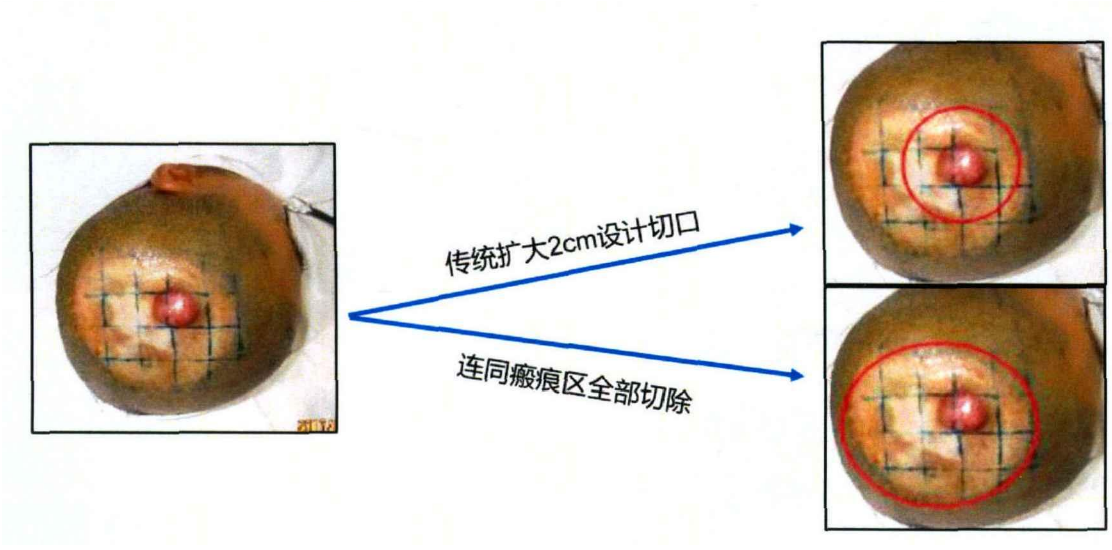

图 2 传统扩大切除法，仅以体表所见瘤体形态扩大切除，盲目性较大

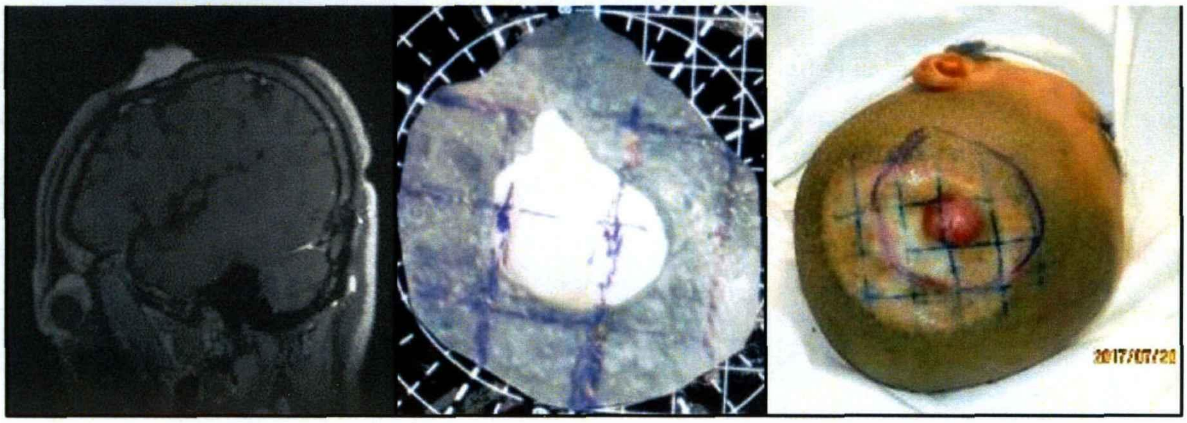

图 3 MRI 三维重建瘤体，提示瘤体深部呈不规则形状，向左下方延伸，提示应以此扩大设计切口。

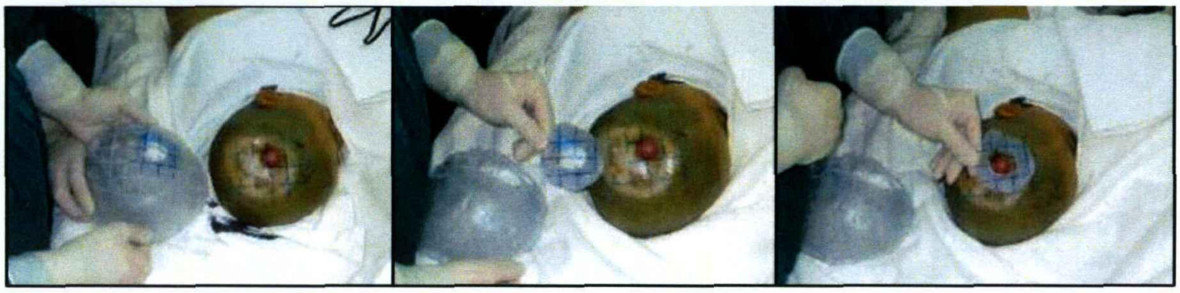

图 4 3D 打印肿瘤模型术中应用

(C)1994-2022 China Academic Journal Electronic Publishing House. All rights reserved. http://www.cnki.net

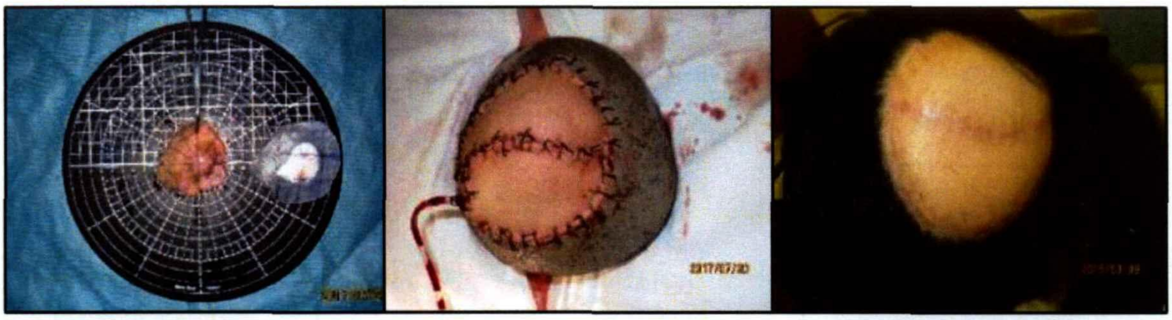

图 5 瘤体-模型比对，创面修复及愈后情况

4.2 背部巨大皮肤隆突性纤维肉瘤

患者男性，59 岁，因背部巨大肿物入院，7 年间因背部皮肤隆乳纤维肉瘤行手术 3 次，此次入院考虑肿瘤复发。瘤体巨大，其范围难以评估。术前对其行 MRI、CT 扫描。提取肿瘤模型，设计个性化肿瘤切除导板。通过 3D 打印成模，指导肿瘤扩大切除范围设计。肿瘤切除后创面以局部背部皮瓣+植皮术修复。

意义：1、患者瘤体范围巨大，单纯凭肉眼、手感难以准确判定手术范围，通过基于影像数据的 3D 打印模型、肿瘤切除导板可为判定肿瘤范围提供参考。2、模型可为术者提供更多信息，例如通过模型可见瘤体与骨骼关系，瘤体包绕肩胛骨下部，前次手术时去除了部分肋骨，复发的瘤体已侵入肋间缺损处，紧贴胸膜。手术时术者对肩胛骨、肋间隙处的瘤体进行了着重处理。

(C)1994-2022 China Academic Journal Electronic Publishing House. All rights reserved. http://www.cnki.net

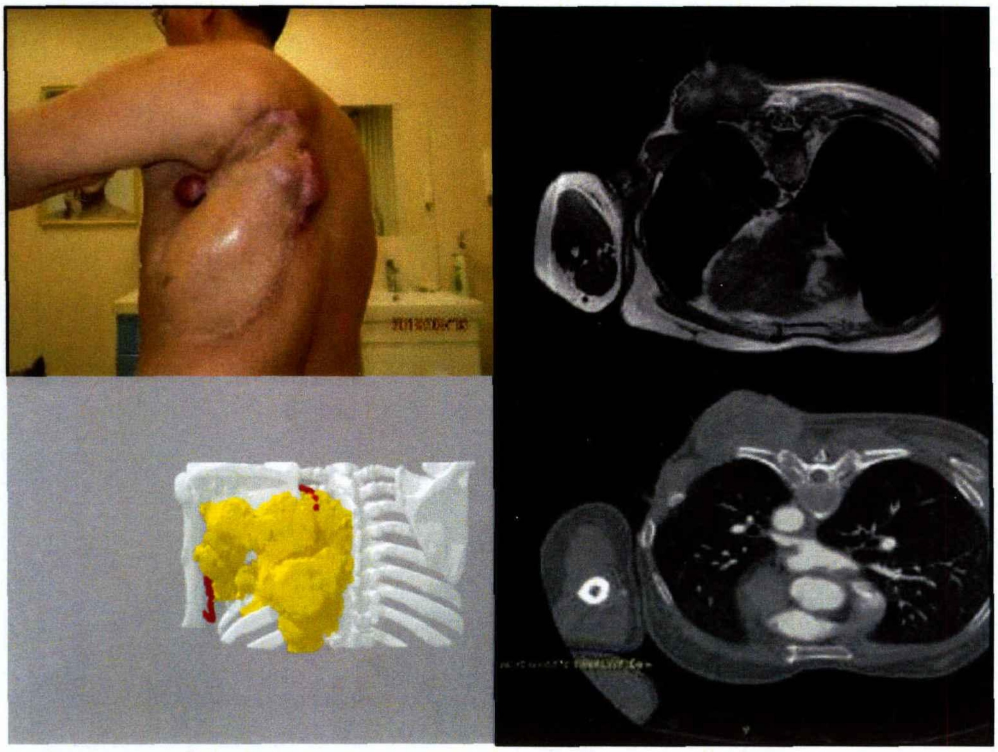

图 6 基于 MRI 数据重建肿瘤，并提取 CT 的骨骼、血管等数据，合成数字化模型

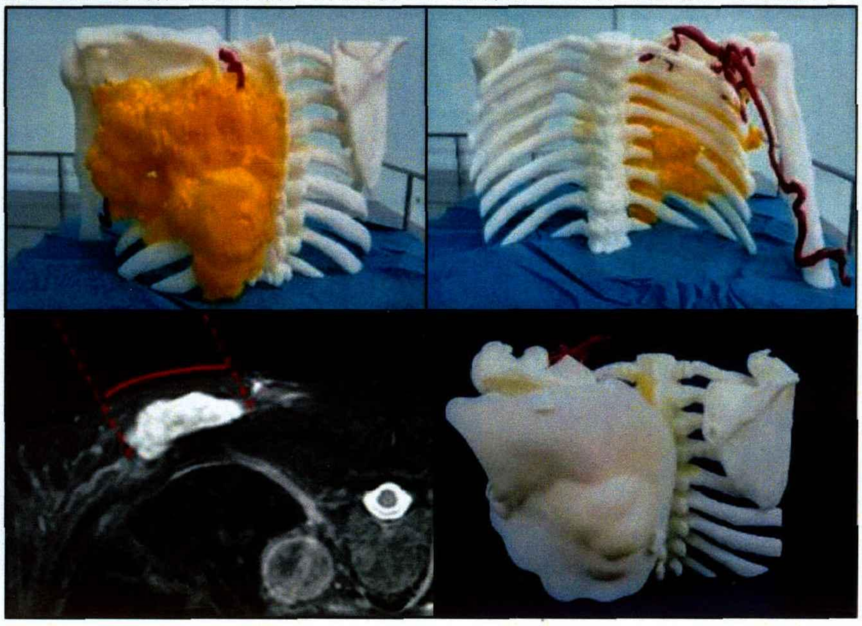

图 7 设计肿瘤切除导板模型，以 3D 打印将肿瘤模型、肿瘤切除导板模型成模

(C)1994-2022 China Academic Journal Electronic Publishing House. All rights reserved. http://www.cnki.net

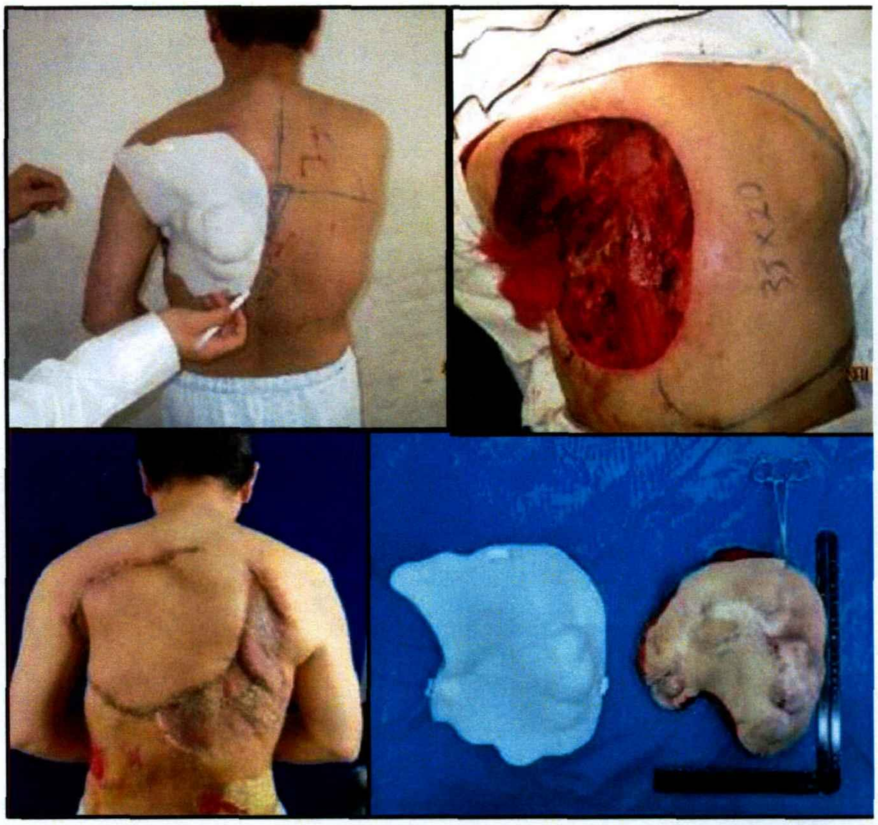

图 8 肿瘤切除导板模型指导手术

4.3 臀部巨大鳞癌

患者男性，44 岁，因臀部巨大鳞癌入院。患者臀部部患有大汗腺炎 20 余年，臀部病变范围逐渐扩大，病理证实为鳞癌。病变边界有色素沉着印迹，但瘤体范围不清，深部侵及范围不清。通过行 MRI 扫描，行瘤体三维重建，设计肿瘤切除导板。以模型指导手术范围设计，肿瘤切除后创面予植皮修复。

意义：1、通过参考肿瘤切除导板，可帮助术者进一步精确判定瘤体范围，通过参考肿瘤模型，可帮助术者进一步精确了解瘤体深度。2、术后进行病理-大体-影像比对研究，结果显示肿瘤边界全部位于影像/导板边界之内。

(C)1994-2022 China Academic Journal Electronic Publishing House. All rights reserved. http://www.cnki.net

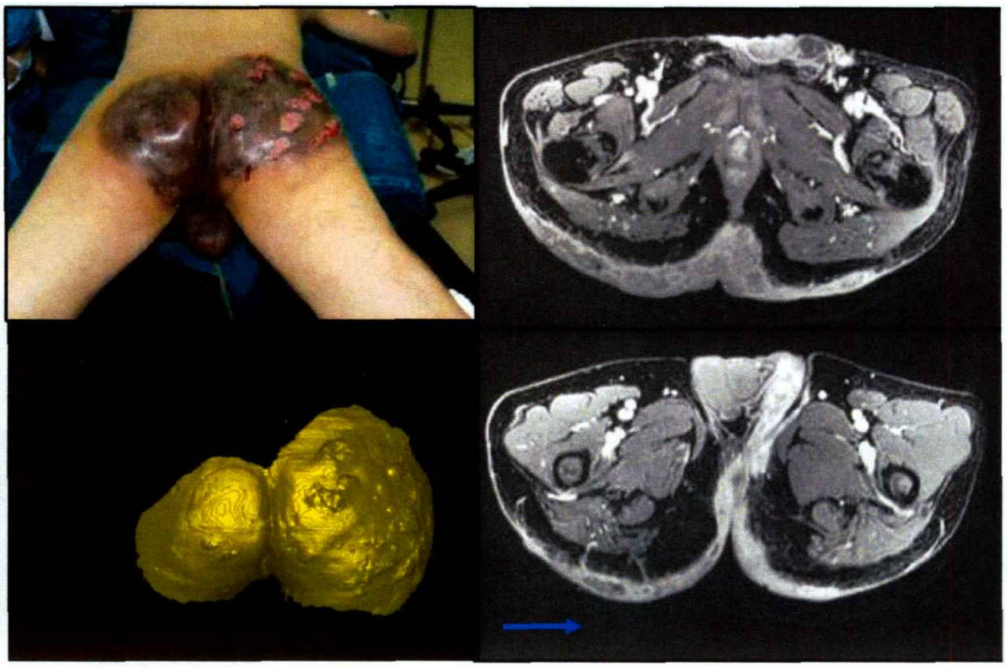

图 9 以臀部 MRI 数据三维重建获取肿瘤三维模型

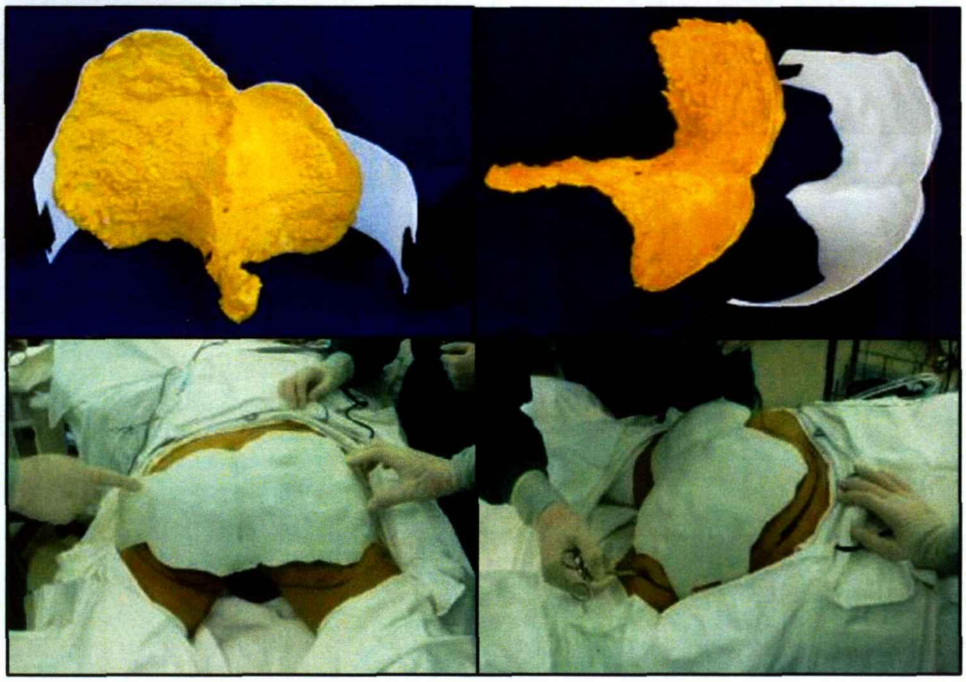

图 10 设计肿瘤切除导板，3D 打印成模，指导手术

(C)1994-2022 China Academic Journal Electronic Publishing House. All rights reserved.   http://www.cnki.net

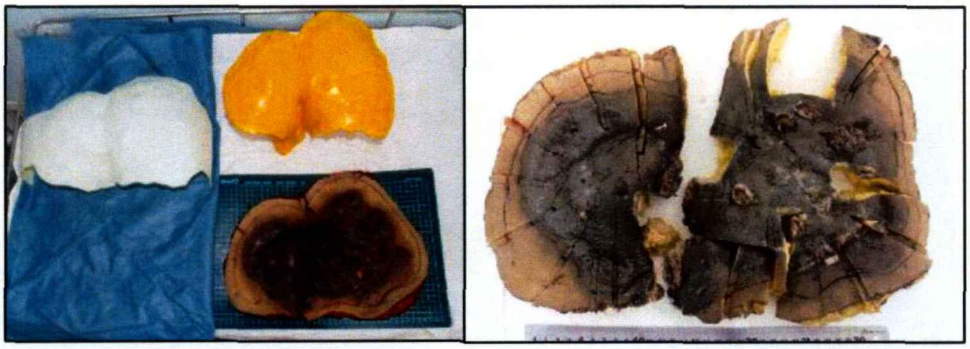

图 11 模型和肿瘤比对，对肿瘤边缘取材病理检测

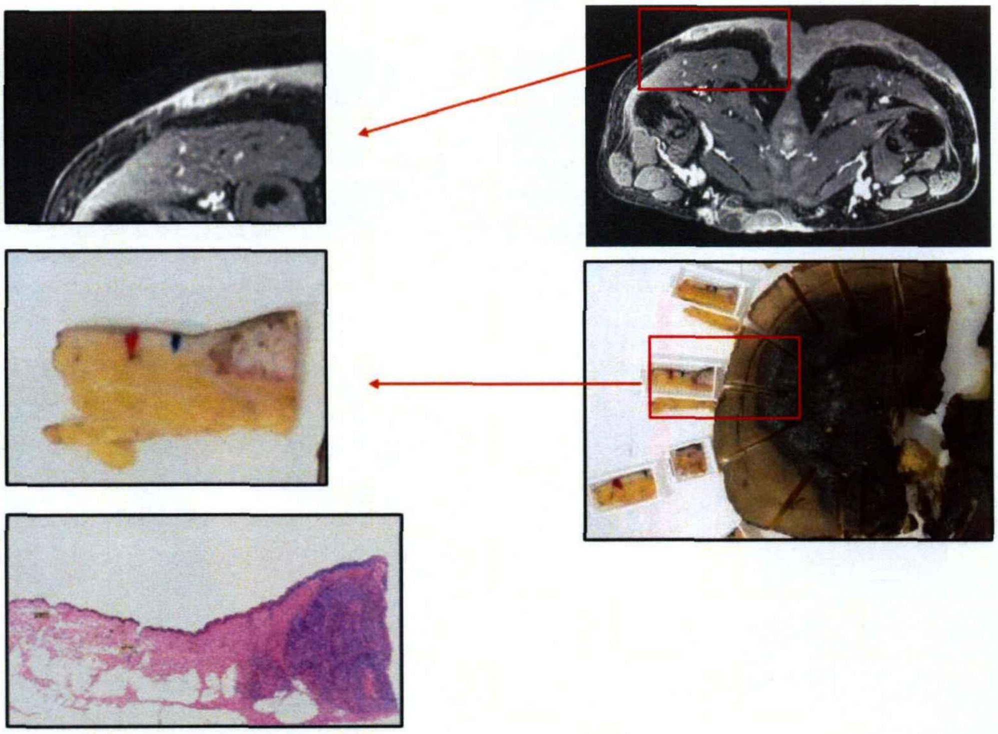

图 12 对肿瘤边界进行病理-大体-影像（导板）比对，可见肿瘤边缘位于影像(导板)线以内

5 讨论

5.1 体表肿瘤 MRI 成像及三维重建具有较强的临床应用价值

肿瘤 MRI 成像已经广泛应用于外科学的各个领域，针对不同肿瘤发展出相应扫描序列，对疾病定性诊断有着重要意义。例如神经外科通常加扫 DWI 弥散加权序列[7]，乳腺外科通常加扫 DCE 动态增强序列或 3D-VIBRANT 序列[8]，而整形外科缺乏体表肿瘤特有的扫描序列，相关研究不多。这是由于：1、体表肿瘤位置较表浅，触诊视诊简单易行，切取瘤体组织行病理学检查即可确诊定性，MRI 未被医生重视。2、体表肿瘤可发于身体任何浅表部位，整形外科医生跟据其生长部位开检查单，影像科医生依据部位选择扫描序列，并没有研发出针对体表肿瘤的特定扫描序列。

MRI 扫描的目的通常是定性诊断，其层厚、层间距通常较大，并没能涵盖全部

(C)1994-2022 China Academic Journal Electronic Publishing House. All rights reserved. http://www.cnki.net

层信息，存在信息遗漏。只有高分辨率 3D 核磁扫描序列才能完整涵盖全部层信息，满足三维重建需求。临床上使用的高分辨率 3D 核磁扫描序列多样，参数各异，适用于不同性质的肿瘤，但在体表肿瘤方面尚无相应研究[9]。我们课题组通过摸索发现 3D-SPACE、3D-Cube、3D-LAVA 序列[10, 11]比较适合体表肿瘤成像及三维重建，并摸索出最佳成像参数，填补了相关研究领域的空白。

核磁共振的图像通常为横截面图像，即 2D 信息，包括轴位、矢状位、冠状位、MIP 任意截面重建等。外科医师通常通过读片获取所需信息，如肿瘤大小、毗邻关系等，有经验的医师可在脑海中将 2D 信息转化为 3D 信息，指导临床应用，这对医师的解剖知识和临床经验具有较高要求。随着数字化技术的发展，通过图像后处理软件三维重建特定组织的技术日益成熟，可直接呈现 3D 信息，提高了手术可视化和精确化程度，为外科医师手术决策提供了有力支持，同时也缩短了年轻医师学习曲线[12, 13]。例如当前临床已经广泛应用的冠状动脉三维重建、脑血管三维重建等技术，其价值已得到广泛认可。而在整形外科领域，对体表肿瘤三维重建方面的研究还很少。本课题组对体表肿瘤的三维重建和可视化进行了探索研究，初步取得一定成果，展示出较强的临床应用价值。

5.2 准确了解“肿瘤形态”对术者成功实施手术具有重要意义

“肿瘤形态”包含了诸多要素，包括瘤体的形状、大小、在体位置、浸润层次等。通常情况下，整形外科医师依靠视诊和触诊判断体表肿瘤的“肿瘤形态”，这种粗糙的判断方法对于良性体表肿瘤尚可适用，而对于恶性体表肿瘤或较复杂的体表肿瘤不免存在误差。实践中我们发现遇到以下情况时有必要对肿瘤形态进行充分细致评估：

5.2.1 瘤体可能呈不对称生长。

在传统观念认为肿瘤呈现扩张性生长，其形态应为标准的球形或对称性形状，但实践中我们发现，肿瘤形态受其生长位置和局部组织疏松程度的影响。例如，头皮鳞癌和头皮转移癌浸润到帽状筋膜层后，容易沿其下的潜在间隙局部扩散，在重力作用下瘤体容易向在体位置较低的方向延伸生长，使瘤体外形呈现不对称性[14]。

5.2.2 瘤体可能呈现“触手样”、“外小内大”的形态特点

某些体表肿瘤切除后容易反复发作，我们认为其特殊的肿瘤形态是其中一个重要原因。例如皮肤隆突纤维肉瘤（DFSP）。DFSP 起源于皮肤组织，但在皮下呈“触手样”浸润生长，可延伸至距离瘤体中央很远的地方[15]。瘤体露出体表的部分通常较小，其

(C)1994-2022 China Academic Journal Electronic Publishing House. All rights reserved. http://www.cnki.net

深部的实际体积远大于此，肿瘤形态呈现“外小内大”的特点。术者如果不能对其肿瘤形态进行充分评估，极易造成露切，导致肿瘤复发。

5.2.3 巨大、复发体表肿瘤情况复杂

反复发作、多次手术后的体表肿瘤患者常令整形外科医师感到棘手，一方面肿瘤形态难以评估，其形态、大小、深度等仅凭肉眼难以准确判断，另一方面反复的手术操作已经破坏了局部正常组织结构，增加了评估难度。例如在一例背部复发性 DFSP 患者中我们发现，由于前次手术中去掉了部分肋骨，复发的肿瘤已经沿胸廓缺损处侵入胸廓，累及胸膜，肿瘤形态呈现术后特有的表现。例如在头皮肿瘤中，瘤体可沿帽状筋膜下局部扩散生长，提高了肿瘤完整切除的难度。

对于以上这些情况，尽管大多数整形外科医生均能认识到：对体表肿瘤行 CT、MRI 等影像学检查是一个很好的选择，但仅仅停留在这一步是不够的。我们建议：1、对体表肿瘤应进行特定的高分辨率 3D 核磁扫描；2、对瘤体进行三维重建，准确了解其“肿瘤形态”；3、应用多种手段将三维重建数据用于指导临床手术[16, 17]。

5.3 3D 打印肿瘤模型可帮助医生准确了解肿瘤形态

三维重建数据有多种应用方式，我们课题组选择通过 3D 打印将肿瘤模型实物化。3D 打印技术是快速成型技术的一种，其原理是在计算机控制下依照模型数据将粉末状的原材料喷出，逐层打印黏合，最后叠加成形。3D 打印技术的出现革新了生产方式，被誉为“第三次工业革命”，近年来相关技术研究得到迅猛发展，在医疗领域的应用也逐年增多[18]。我们课题组将肿瘤模型以 3D 打印的方式呈现出来，将虚拟的数字模型转化为实物模型，一方面帮助医生准确了解肿瘤形态，优化制定手术方案，一方面也为医生向患者及家属交待病情提供了形象工具[19]。

5.4 3D 打印肿瘤切除导板进一步将“可视”转化为“可用”

3D 打印肿瘤模型准确呈现了肿瘤形态，解决了“可视”问题。但肿瘤模型本身存在一定厚度，无法直接贴合于体表指导瘤体定位，如何在患者体表定位肿瘤边界仍是一个待解决的问题。我们课题组提出了设计“肿瘤切除导板”的思路，将“可视”转化为“可用”，直接指导临床手术。

肿瘤切除导板是指基于肿瘤数字模型设计的、固定于体表后能全部涵盖肿瘤范围的导板结构。设计时通常于肿瘤模型边缘各点依次向体表做垂直投影，各投影点所包绕形成的范围即为肿瘤切除导板的主体。为将导板更准确固定于体表，在其边缘可增

(C)1994-2022 China Academic Journal Electronic Publishing House. All rights reserved. http://www.cnki.net

添设计个性化卡槽，例如头部肿瘤导板设计经双测耳上卡槽，腹部肿瘤导板设计经腹壁外侧缘的卡槽。使用时通过固定卡槽即可固定导板。

应用肿瘤切除导板的意义在于：1、理论上可认为，导板的边缘线即为肿瘤影像学边缘在体表的投影线，全部瘤体均位于导板覆盖范围内。在此导板边缘线上设计扩大切除范围，较之于直接在肿瘤的肉眼边缘上设计扩大切除范围无疑更加准确。2、导板呈扁平形，其底面可与体表紧密贴合，在使用时直接扣放于体表固定，解决了肿瘤模型因有厚度而无法直接使用的问题。3、导板的范围涵盖了全部瘤体，包括瘤体沿皮下延伸的部分，因此将导板模型直接扣放于瘤体模型上时可能并不能完全紧密贴合，二者之间的间隙即为瘤体皮下延伸部分之上的组织，间隙厚度反映了该处瘤体的深度。

5.5 对肿瘤边界进行病理-大体-影像（导板）边界比对

病理检测是评价手术方法准确性的金标准，对于本课题提出的“基于 MRI 的肿瘤切除导板指导体表肿瘤切除”“这一方法我们进行了病理学研究。

本课题组在前期研究中发现：1、病理大切片在体表肿瘤的边界研究中具有不可替代的重要价值。2、对瘤体进行“连续病理大切片→数字切片扫描→病理软件分析→病理三维重建”，可以更精确掌握体表肿瘤的形态特征。3、体表肿瘤存在突破瘤体主体的散在肿瘤灶，这些可能是导致肿瘤切不净易复发的重要原因。4、形态上，体表肿瘤的病理-大体观-影像之间存在着较强的一致性[2]。

在本研究中我们继续使用病理大切片，对肿瘤边界进行了进一步的影像和病理比对。众所周知，MRI 的原始图像为 2D 信息，要进行比对必须先将影像信息转化为病理切片上能识别的信息，这是困扰研究者的一大难题。我们将肿瘤切除导板的边界视为 MRI 影像学边界，肿瘤切除后以刀进行划痕标记，并涂为红色，刀痕和染色在病理切片上均可显示，这样就巧妙地实现了影像-病理的可比性。

尽管目前进行的病理检测全部显示肿瘤实际病理边界位于肿瘤导板切缘内侧，但数目较少，统计学意义尚不足，有待于在今后的研究中进一步扩大样本量。

此外，肿瘤患者的愈后情况和复发情况是评价肿瘤切除方法的重要指标，由于随访时间不足，本研究尚未进行相关统计分析，这也是今后的改进方向。

6 结论

（1）通过选择合适的序列和设定相应参数，高分辨率 MRI 可将体表肿瘤充分显

(C)1994-2022 China Academic Journal Electronic Publishing House. All rights reserved. http://www.cnki.net

影，满足三维重建需求。

（2）基于 MRI 数据可获得体表肿瘤数字模型，可通过 3D 打印成模，有助于术者更好地了解肿瘤形态。

（3）基于 MRI 数据可设计得到个性化肿瘤切除导板，可通过 3D 打印成模，将肿瘤影像学边界精确定位于体表，指导术者行扩大切除。

（5）对于较复杂的体表肿瘤患者，使用“基于 MRI 的 3D 打印肿瘤切除导板法”是一个很好的选择。目前数据显示，该方法指导体表肿瘤切除手术的精确度为 100%，具有较强的临床应用价值。

(C)1994-2022 China Academic Journal Electronic Publishing House. All rights reserved. http://www.cnki.net

第二部分 肿瘤三维投影与“肿瘤地图”投影在常见体表肿瘤手术中的应用

1 引言

本研究使用平板电脑和便携式投影仪构建了简单的增强现实（AR，Augmented Reality）设备。提出“肿瘤地图”的概念，通过投影肿瘤三维模型和个性化的“肿瘤地图”，帮助医生确定肿瘤影像学边界。本研究设计了一种定位三角，可辅助 MRI 数据在体表的投影定位。对病情较复杂的体表肿瘤患者，我们推荐的临床思路为:“MRI 扫描→设计个性化肿瘤地图→投影定位→手术应用”。

2 材料与方法

2.1 研究对象

研究范围为解放军总医院整形外科 2017 年 1 月-2018 年 12 月收治的体表肿瘤病人，从中选择合适的对象进行研究。入组标准包括：（1）肿瘤位于躯干胸腹部体表，该部位表面曲率较小适合投影操作；（2）肿瘤较大（直径>3cm）需外科手术切除；（3）肿瘤可为初发或复发；（4）未见远处转移；（5）患者能配合进行 MRI 扫描，平均时间约半小时，具体要求包括①无增强造影剂如钆剂过敏；②肾功能正常（血肌酐：男 54-106umol/L，女 44-97umol/L）；③无明显幽闭恐惧症。排除标准为：因各种原因无法行增强 MRI 扫描者。课题先后纳入病例 9 例，其中数据较完备的病例 4 例。

2.2 对体表肿瘤行 MRI 检查

2.2.1 扫描序列

T1、T2 平扫，增强后 T1 平扫；薄层 3D 序列：3D-SPACE、3D-Cube、3D-LAVA；

2.2.2 扫描参数

GE 3.0T MR750 核磁共振机，层厚 2mm，层间距-1mm，相位加速因子 2Ph，TR 3ms，TE 1.3ms，FOV 40x32，矩阵 270x160，空间分辨率为 1.5x2x1mm，反转角 12°，接收带宽 125kHz。

2.2.3 其他设定

病人检查时保持体位与术中相同，通常取仰卧位，瘤体朝上。于其肿瘤附近体表固定两个定位三角，并在皮肤上做好标记。进行核磁共振扫描时胸腹部加装呼吸门控，嘱患者平静呼吸，必要时吸气后屏气。

2.3 设计体表肿瘤三维模型和“肿瘤地图”

(C)1994-2022 China Academic Journal Electronic Publishing House. All rights reserved. http://www.cnki.net

2.3.1 设计体表肿瘤三维模型

将 Dicom 数据导入重建软件如 Mimics、3Dslicer、AW4.6。依次进行：（1）提取肿瘤组织。选择瘤体清晰显示的薄层 3D 序列，设定阈值对肿瘤区域进行自动提取，或手动描记添加提取，获得肿瘤完整的三维数据。（2）提取定位三角。以同样方法单独提取定位三角，将二者在同一坐标体系内进行融合。必要时可继续提取正常组织、血管、骨骼等数据。（3）对各组织模型上色，通常肿瘤为黄色，定位三角为绿色，血管为红色，骨骼为白色。将各数据融合即可得到最终版体表肿瘤三维模型，导出为 STL/PLY 格式。

2.3.2 设计“肿瘤地图”

在冠状位下取模型的体表垂直投影图，即可得到“肿瘤地图”。添加坐标轴。通常以典型的体表解剖标志设计横纵坐标轴，如胸部区域可设计 X 轴为水平线，Y 轴为胸骨中线；或者参考定位三角的底边设计横坐标，其垂线为纵坐标。导出为 JPG/PNG 格式。

2.4 应用肿瘤三维模型投影和“肿瘤地图”投影指导手术

2.4.1 应用肿瘤三维模型投影指导手术

将体表肿瘤三维模型导入 ipad、智能手机、PC 等终端，使用三维软件打开，通常通过简单操作即可实现模型的旋转、缩放、移动。连接便携式无线投影仪，可将三维数据实时投射于病人体表。投影仪应保持垂直于体表，通过调整远近距离改变投影面积。将模型的定位三角与皮肤标记处匹配，即可实现模型投影在体表的准确定位。

2.4.2 应用“肿瘤地图”投影指导手术

将“肿瘤地图”以读图软件打开，投影后通过将定位三角与皮肤标记处匹配、坐标轴与体表坐标轴匹配实现“肿瘤地图”的精确定位。

术前利用投影显示完整的瘤体范围，以记号笔于体表描记边界，即为肿瘤的影像学边界，在此基础上设计扩大切除切口。术中继续投影，为术者实时提供肿瘤范围、周边血管等信息。

3 结果

（1）全部病例均可通过分析高分辨 MRI 数据获取肿瘤三维模型；

（2）通过使用移动终端和便携式投影仪，肿瘤三维模型可帮助术者于患者体表描记肿瘤影像学边界；

(C)1994-2022 China Academic Journal Electronic Publishing House. All rights reserved. http://www.cnki.net

（3）全部病例均可设计得到“肿瘤地图”，通过投影法帮助术者于患者体表描记肿瘤影像学边界，并提供血管、骨骼等毗邻结构信息。

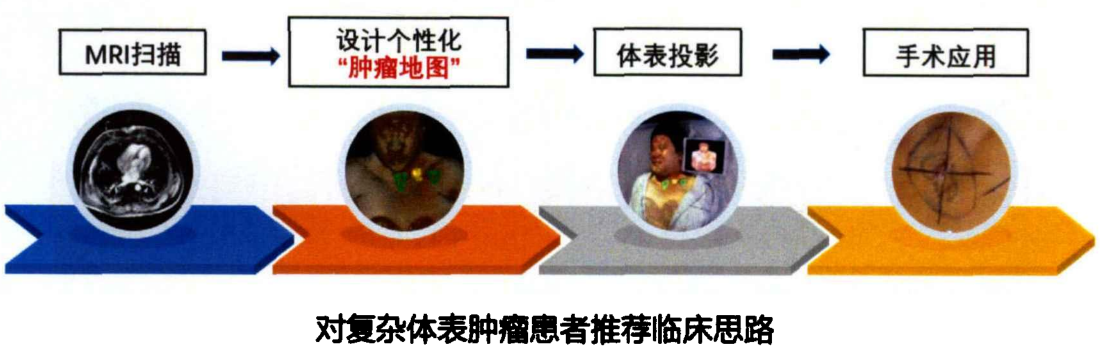

图 13 对复杂体表肿瘤患者推荐临床思路

4 典型病例

左侧锁骨下复发性皮肤隆突性纤维肉瘤。

患者女，36 岁，因左侧锁骨下皮肤隆突性纤维肉瘤复发 1 月入院。半年前层行手术切除，遗留瘢痕，此次复发拟行手术治疗。予行 MRI，对瘤体行三维重建，设计“肿瘤地图”，将其投影于体表，判定肿瘤范围。予行扩大切除术，局部皮瓣转移修复。

意义：患者肿瘤体表部分较小，且与瘢痕混杂，难以判断瘤体边界，盲目扩切容易露切。通过“肿瘤地图”法，可以充分定位其影像学边界，在此基础上进行扩切，提高了手术准确度。

(C)1994-2022 China Academic Journal Electronic Publishing House. All rights reserved.   http://www.cnki.net

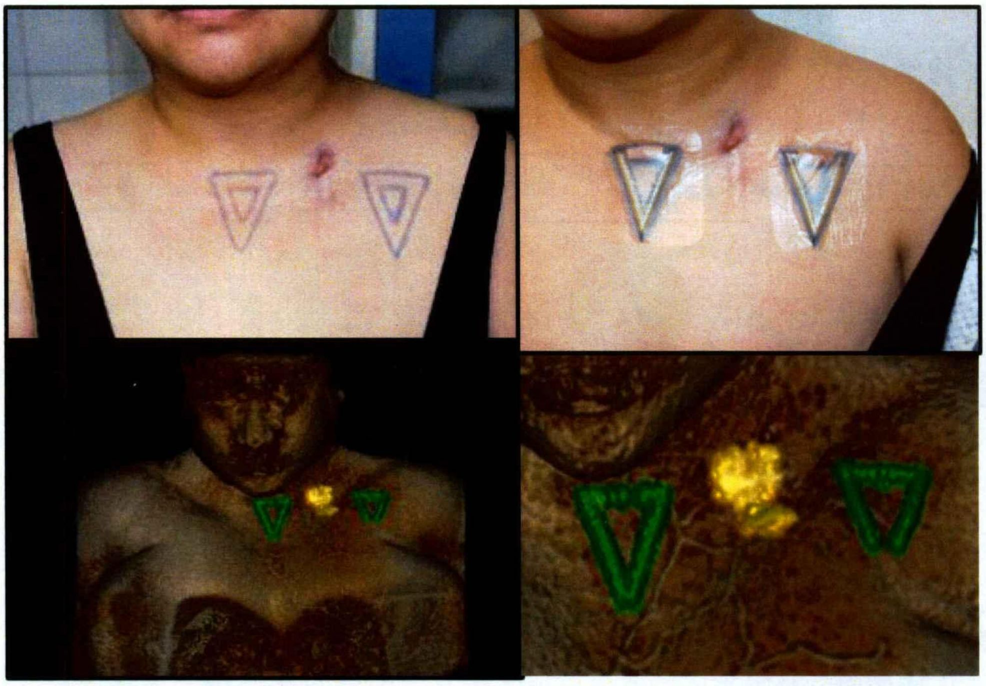

图 14 体表固定定位三角后行 MRI，三维重建，设计得到“肿瘤地图”（左下）

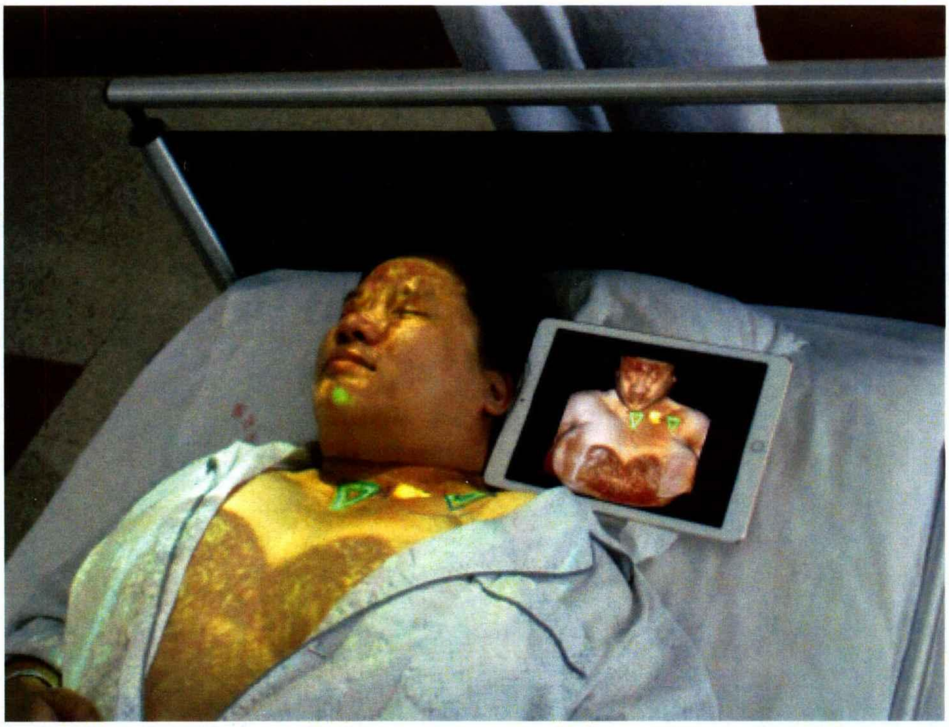

图 15 以投影法将“肿瘤地图”定位于体表

(C)1994-2022 China Academic Journal Electronic Publishing House. All rights reserved. http://www.cnki.net

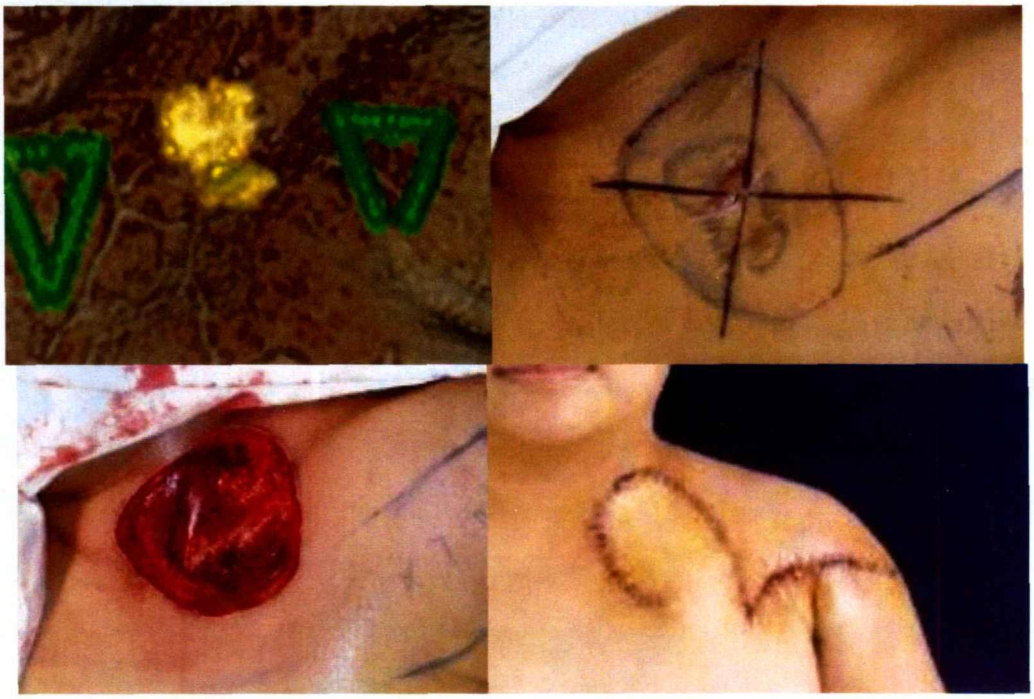

图 16 体表描记肿瘤影像学边界，设计扩大切除范围，局部皮瓣转移修复

5 讨论

5.1 肿瘤三维投影与““肿瘤地图”投影具有较强的临床应用价值

体表肿瘤虽然位置浅表，但其肉眼所见的部分并不是瘤体的全部，很多肿瘤呈现“外小内大“、”冰山样“的形态特点，瘤体可沿皮下向周边延伸生长，此外复杂性、复发性体表肿瘤的形态更是复杂多样。准确了解“肿瘤形态”对术者成功实施手术具有重要意义。我们通过对瘤体进行核磁扫描和三维重建，获取得到肿瘤模型数据，能够对肿瘤形态有一个准确全面的认识。

将肿瘤模型数据进一步应用于临床有很多方法，我们课题组先后尝试了 3D 打印法、虚拟现实眼镜法、体表投影法等，各有利弊。其中体表投影法简单易行，值得推广。将肿瘤模型数据以 STL 格式导入移动终端，如平板电脑、智能手机、笔记本电脑等，连接便携式投影仪，于瘤体正上方垂直投影，通过垂直方向移动投影仪调整投影大小，使定位三角、坐标系于体表标记完全重合，此时即认为肿瘤三维模型准确定位于体表，可行肿瘤边缘描记。

实践中我们发现，三维格式的肿瘤数据可以随意缩放、移动、旋转，强大的可调节能力虽然便于各角度观察模型，但也同时增加了将其准确投影定位的难度。通常通过调节 2 组 4 个变量实现匹配：模型角度、模型体积、投影仪角度、投影仪高度。为

(C)1994-2022 China Academic Journal Electronic Publishing House. All rights reserved. http://www.cnki.net

了进一步提高操作精确度，我们课题组首次提出了“肿瘤地图“的概念，即在冠状位下取肿瘤模型的体表垂直投影图，同时包含肿瘤、定位三角、坐标系以及其他必要信息，导出为 JPG/PNG 格式。“肿瘤地图“是垂直位视图，只要保证投影仪位于垂直位置，无需调节模型角度、投影仪角度，仅需调节剩余 2 个变量：模型体积、投影仪高度。简化了调整流程，提高了投影精度。

“肿瘤形态“是一个三维空间概念，它涵盖的信息不仅包括肿瘤直径、在体位置，还包括肿瘤厚度、在体深度等。投影法的实质是舍弃了肿瘤厚度、在体深度等 z 轴信息，而仅应用 x、y 轴的二维信息指导手术，帮助术者了解肿瘤的位置、大小、浸润范围等。二维数据方便临床直接应用。但应当认识到，肿瘤的完整切除是一个三维概念，仅有二维的切净是不够的，在手术时还应同时关注肿瘤深部的切净。

5.2 使用定位三角辅助投影于体表准确定位

如何实现投影与体表的精确匹配定位是一个困扰临床医生的问题。脑外科医生进行颅内肿瘤定位投影时使用维生素 E 胶囊定位，可将投影与实物实现注册匹配，但是实践中我们发现胶囊呈现椭球形，其投影距离、角度难以精确控制。S. Hummelink 等在研究中利用投影仪将腹壁下动脉穿支重建图像投影于体表，巧妙地选择放置尺子作为参考，通过使投影达到指定刻度实现放大倍数的确定[20]。该方法有一定临床价值，但放置尺子的方法可重复性差，误差较大。

我们课题组设计了一种三角定位装置，可用于将核磁数据于体表精确匹配。该定位装置为一密闭中空管道，通常将外观设计为三角形，亦可设计成圆形、矩形、多边形等各种形状。内部注入维生素 E 乳剂，可于 T1、T2 扫描下均显影。以硅胶为原料进行 3D 打印制作成模。使用时将定位三角固定于患者体表，硅胶柔软可与体表良好贴合。患者携带定位三角进行核磁检查，在后续三维重建瘤体时同时重建定位三角，二者处于同一坐标系内，可保持空间相对位置的稳定。在投影定位时将定位三角与体表描记痕迹精确匹配，即可做到核磁数据于体表精确定位。选择三角形设计是因为其形状较之于圆形、椭圆形更适合定位使用，在调整距离、角度时匹配更精确。

5.3 投影法的优点、缺点探讨

投影法将电脑中的三维/平面视图投射到现实的患者身体表面，是一种简单的“增强现实”（AR，Augmented reality ）技术。可以提高整形外科手术的可视化程度，同时有利于肿瘤的精确切除。AR 技术是指通过数字化技术将虚拟的信息叠加到现实场

(C)1994-2022 China Academic Journal Electronic Publishing House. All rights reserved. http://www.cnki.net

景，是当前数字化领域的研究热点，在医学领域具有广阔的应用前景[2]。

投影法最大的优点是直观、可视化能力强，它将断面的肿瘤影像视图转化为可视的投影视图，直接呈现肿瘤的影像学边界，为术者提供帮助。其次，还能根据需要投影肿瘤毗邻的重要结构，如穿支血管、骨骼、正常组织等，帮助整形科医生整体把握术区的解剖关系。此外，投影可以持续应用到术中。我们将投影仪固定于手术灯或其它固定架上，术前进行良好定位后保持病人、支架位置不动，投影仪可于术中持续进行投影，不受手术进程影响。即使术中皮肤切开掀起，体表标记模糊或擦除的情况下，仍能继续起到定位指示的作用。需要注意的是，神经外科定位中存在“影像漂移”，开颅后组织形变引起定位偏移，我们发现整形科亦存在类似问题，如切掉体表肿瘤、切开皮肤后，软组织会因为张力而向创面垂直方向扩张，我们称之为浅表“软组织漂移”，同样会导致定位不准。其对浅表组织影响大，越深层组织影响越小，我们在应用中应考虑到这种误差。

投影法的缺点为定位不够稳定，误差较大。其误差主要来源于：（1）投影与实物匹配时产生的误差；（2）平面图投射于体表后产生的曲面偏差。实践中我们发现，实现投影与实体的坐标系、定位三角标志完全重合并非易事，需要长时间的调节修正，且可重复性差。投影仪本身的晃动亦可对匹配稳定性造成影响，病房内我们最初以双手手持投影仪晃动较大，后改为自拍杆固定及可伸缩支架固定，手术间内我们将投影仪固定于手术灯，已能充分减少投影仪晃动所致定位误差。

6 结论

（1）使用肿瘤三维模型投影可帮助医生于患者体表准确描记肿瘤影像学边界。

（2）“肿瘤地图”包含了肿瘤范围、肿瘤位置、毗邻组织（如血管、骨骼）等信息，可以通过投影法应用于临床，对临床医师具有指导意义。

（3）使用移动终端和便携式投影仪可以构成简单的增强现实设备，是数字化技术应用于体表肿瘤治疗的初步探索。

（4）对于较复杂的体表肿瘤患者，使用“基于 MRI 的肿瘤地图投影法“是一个很好的选择，可有助于术者参照肿瘤的影像学边界设计手术。

(C)1994-2022 China Academic Journal Electronic Publishing House. All rights reserved. http://www.cnki.net

第三部分 基于MRA成像的“穿支血管地图”在整形外科皮瓣手术中的应用

1 引言

本研究探索了MRA对穿支血管的成像能力。提出设计个性化的“穿支血管地图”。通过三种定位方法（测量法、打印法、投影法）将其定位于体表，指导术者选择最佳穿支。对较复杂的拟行穿支皮瓣手术的患者，我们推荐的临床思路为:“MRA/CTA扫描→设计个性化“穿支血管地图”→体表定位（测量法、打印法、投影法）→手术应用”

2 材料与方法

2.1 研究对象

研究范围为解放军总医院整形外科2017年1月-2018年12月收治的患者，从中选择合适的对象进行研究。入组标准包括:（1）患者因各种原因形成较大创面，需接受穿支皮瓣转移修复或游离皮瓣修复创面;（2）患者可配合进行MRA扫描，平均时间约半小时，具体要求包括①无增强造影剂如钆剂过敏；②肾功能正常（血肌酐：男54-106umol/L，女44-97umol/L)；③无明显幽闭恐惧症。排除标准为:（1）创面修复未使用穿支血管，包括局部任意皮瓣转移修复、植皮修复、直接拉拢缝合;（2）因各种原因无法行MRA扫描者。课题先后纳入病例17例，其中设计较好、数据完备的病例6例。

2.2 行MRA检查

根据患者病情选定扫描区域,对拟探查穿支血管区域进行MRA扫描,获得Dicom数据。采用3.0T MRI磁共振设备(GE Healthcare),八通道相控阵线圈,采用3D-LAVA（three-dimensional liver accelerated volume acquisition, GE Healthcare)，技术参数：TR＜30ms，TE＜5ms，FA15°，层厚1.8mm，间隔0，带宽125KHz，FOV40cm，矩阵大小256×244。造影剂以0.1mmol/kg剂量和2ml/s流速静脉给药，盐水冲洗20ml。

2.3 三维重建影像数据，设计“穿支血管地图”

将Dicom数据导入Mimics/AW4.6进行处理。（1）进行VR（Volume Rendering）容积再现三维重建。重建皮瓣供区的软组织、主干血管、体表标记物，得到一个模型主体。（2）确定穿支血管位置。逐层读片，将全部穿支血管进行标记，对疑似穿支穿出点采用不同颜色标记。（3）于模型表面建立同体表相同的横纵坐标系，通常参考体

(C)1994-2022 China Academic Journal Electronic Publishing House. All rights reserved. http://www.cnki.net

表解剖结构，如腹部以脐为中点、胸部以经两乳头连线为横轴、以胸骨中线为纵轴等，或以定位三角的顶点和底边为参考。(4) 取模型冠状位的垂直投影截图，即得到个性化的“穿支血管地图”，导出为 jpg/png 格式。

2.4 使用“穿支血管地图”指导手术

使用三种方法将“穿支血管地图“准确定位于体表：

2.4.1 测量法（Localization with Distance Measurement , LDM）

参考所建坐标系在“穿支血管地图”上测得各穿支血管点的横纵坐标数值，在体表上借助软尺将各点逐一描记。其他感兴趣点亦可通过测量法描记。

2.4.2 投影法（Localization with Image Projection , LIP）

将“胸部穿支血管地图”导入平板电脑，连接便携式投影仪，调整投影仪角度、距离，可通过匹配影像和体表的定位三角、坐标系，使地图准确定位于患者体表，清晰显示穿支血管穿出点位置、毗邻关系等，如对穿支血管重建亦可清晰显示穿支血管走形。

2.4.3 打印法（Localization with Printed Plastic Film, LPPF）

将“胸部穿支血管地图”以 1:1 的比例打印于透明塑料薄膜上，通过与体表定位三角匹配，将塑料膜与体表完全贴合，在穿支血管等感兴趣处戳孔，即可将其标注于皮肤。

比较各穿支血管情况，制定手术方案，选取最佳穿支设计皮瓣手术。

3 结果

（1）各病例均能通过 MRA 检查显示穿支血管，平均每例 4.5±2.1 支；

（2）各病例均能设计得到“穿支血管地图”，术者依此选择出最佳穿支进行手术；其中 1 例患者术中最佳穿支被破坏，参照“穿支血管地图”选择了备选穿支，成功实施手术。

（3）三种定位方法（测量法、打印法、投影法）均可将“穿支血管地图”准确定位于体表，指导术者实施手术。

(C)1994-2022 China Academic Journal Electronic Publishing House. All rights reserved.   http://www.cnki.net

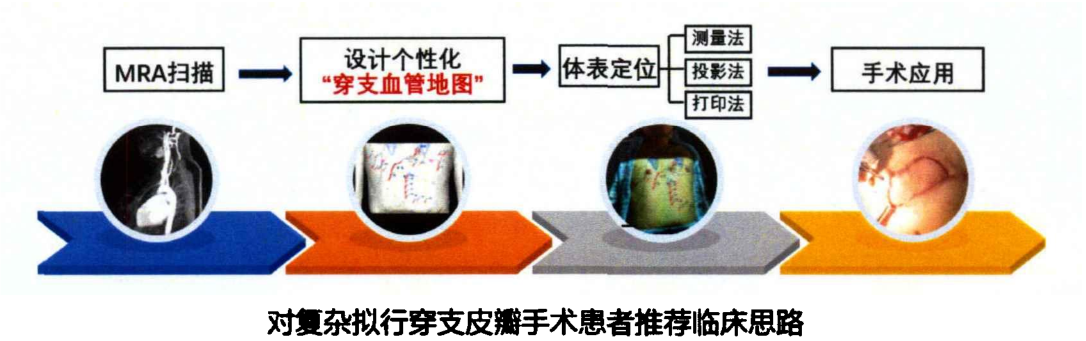

图 17 对复杂拟行穿支皮瓣手术患者推荐临床思路

4 典型病例

患者女性，48 岁，因乳腺癌伴胸壁转移 3 月入院。胸壁可见一肿物，拟与乳腺外科协同手术，行乳腺癌根治术+胸壁肿物切除+皮瓣修复术。对其行胸壁 MRA，分析穿支血管，设计“穿支血管地图”。将其以三种方法定位于体表，可见其胸部有多条穿支可供使用，分别位于肿物的上方、下方、右下方，将穿支进行编号备用。术中切除肿瘤后破坏其中多条血管，最终选择右下方一较大肋间动脉穿支行穿支皮瓣设计。

意义：患者胸部有多条穿支可供使用，切除肿物后破坏了部分穿支，通过分析“穿支血管地图”，发现位于肿物右下方较大的肋间穿支 18 尚可以使用，可见“穿支血管地图”为手术提供了最佳选择。

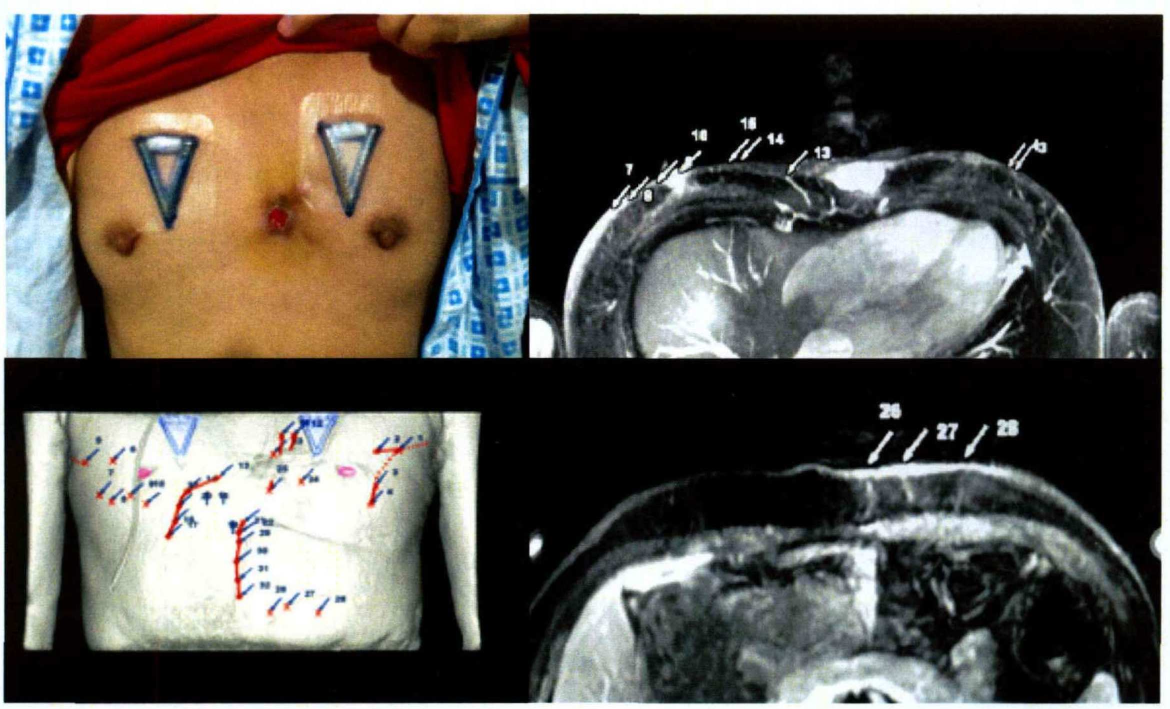

图 18 对胸部行 MRA 扫描，分析穿支，设计“穿支血管地图”（左下）

(C)1994-2022 China Academic Journal Electronic Publishing House. All rights reserved. http://www.cnki.net

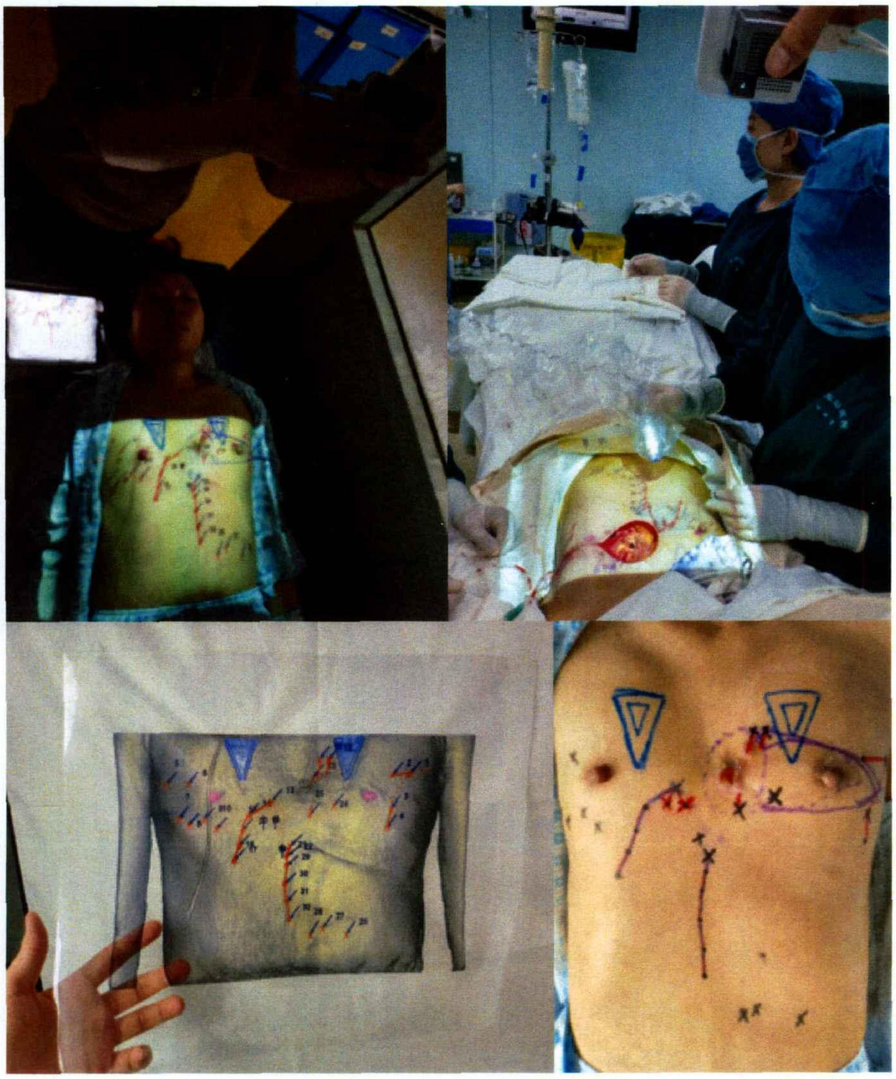

图 19 使用投影法、打印法、测量法，分别将“穿支血管地图”定位于体表，选择最佳穿支 18

(C)1994-2022 China Academic Journal Electronic Publishing House. All rights reserved. http://www.cnki.net

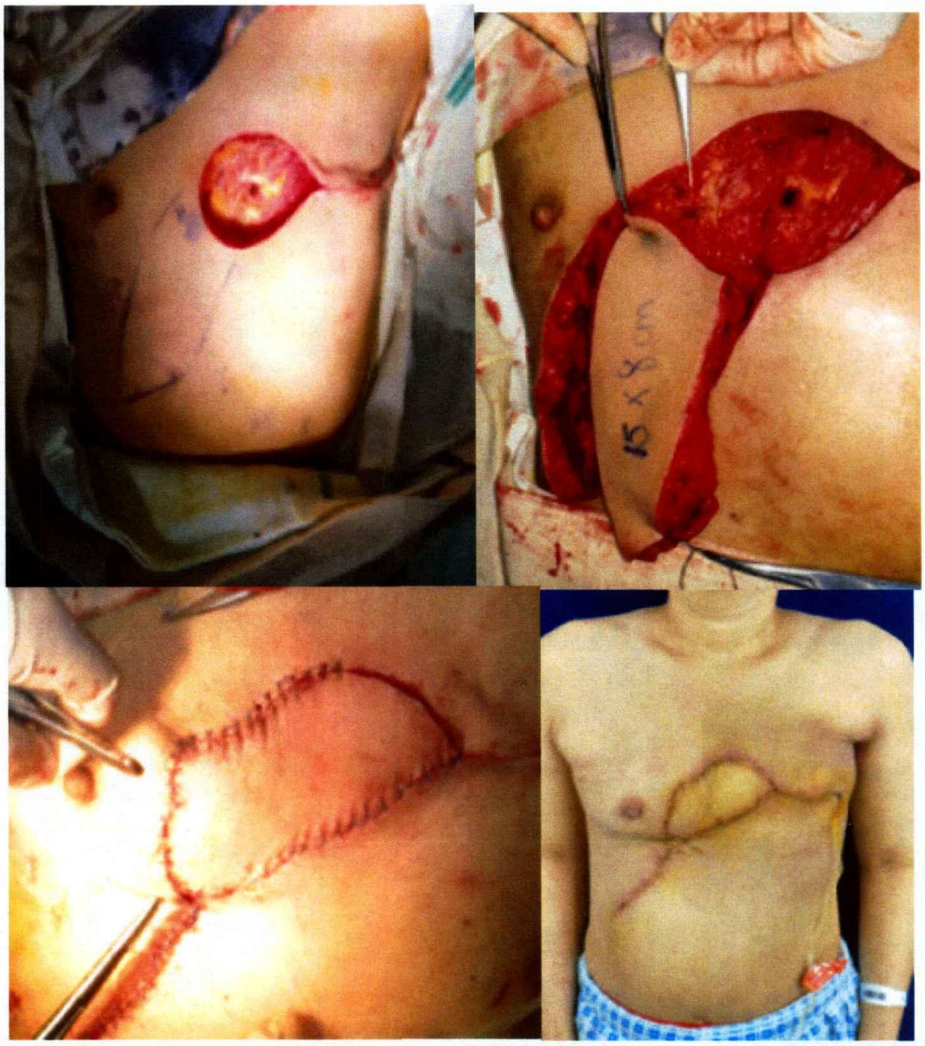

图 20 术中验证穿支 18 存在，为一少见的粗大肋间动脉穿支，成功实施穿支皮瓣手术。

5 讨论

5.1 MRA 显示穿支血管具有独特价值。

穿支血管的成像研究中，CTA 和超声的研究较多，MRA 相对较少[21]。近年来国外学者对 MRA 显示穿支血管的研究逐渐增多，国内尚未见相关研究报道。核磁共振扫描序列繁多，研究潜力巨大。核磁血管成像有多种序列可供选择。其中 TOF 序列于脑血管领域应用较多，但难以显示穿支小血管。目前研究表明增强 LAVA，TRICKS 序列可以显示较小的穿支血管。有研究表明，高分辨率核磁可以使用对比造影剂，在血管停留更久，达到 CT 相似的空间分辨率，可以充分显示小血管[22, 23]。我们在研究中也发现，通过设定合适的扫描参数，MRA 可显示部分穿支血管，具有独特的临床应用价值。

MRA 显示穿支血管的优势主要体现在：（1）MRA 能避免辐射。接受皮瓣手术的

(C)1994-2022 China Academic Journal Electronic Publishing House. All rights reserved.   http://www.cnki.net

病人很多是因罹患肿瘤而手术，有研究表明肿瘤病人更容易因受到辐射而罹患新的肿瘤，因此应当尽量避免 CT 等医源性辐射。MRA 如可达到 CTA 的血管成像能力不失为一个很好的替代选择。（2）体表肿瘤患者通常要进行增强 MRI 检查，我们建议对体表肿瘤病人进行增强 MRI 扫描时加入 LAVA 或 TRICKS 等血管成像序列，在获得肿瘤清晰影像的同时也可显示穿支血管，一次扫描获得多种信息，较小了病人负担。（3）MRA 数据也可以进行三维重建，可以进一步设计个性化的“穿支血管地图”，指导术者选择最佳手术方案。

MRA 也有缺点，其价格相对 CTA 较贵；也需要注射造影剂，不适合肾功能较差者；MRA 扫描时间较长，扫描胸腹部时需要病人憋气配合，限制了扫描范围。因此目前 MRA 穿支血管成像并不能完全取代 CTA，有待进一步探索研究[24]。

5.2 “穿支血管地图”有助于整形外科医生全面、个性化地了解各穿支情况，做出最佳手术决策。

人体的穿支血管变异较大，管径、位置、数目都存在较大的特异性。此外，在某一区域可能有多支穿支血管适合设计皮瓣进行手术。术者选择何种方案通常依据所掌握的解剖知识和个人手术经验。首先初步判断创面附近某处可能存在穿支血管，而后借助超声、CT、MRI 等辅助手段对该兴趣区进行检查，如为阳性即可考虑选择该穿支设计皮瓣。其信息来源相对局限，无法保证所选是最佳方案。因此全面地、个性化地了解患者胸部穿支血分布管情况十分必要。

我们课题组提出基于 MRA 的“胸部穿支血管地图”的概念。术前对患者胸部进行全面扫描，将各穿支血管三维重建，穿出点于体表标注，并测量其直径、穿出肌肉点到胸壁距离等数值，从垂直于冠状位前方截图即可得到一个个性化的、全面的、直观的“胸部穿支血管地图”，为术者提供决策依据。此方法同样使用于 CTA 数据。

应用“胸部穿支血管地图”的优势体现在：（1）将 2D 影像信息转化为 3D 模式显示，再转化为 2D 影像指导手术，提高了手术可视化水平；（2）全面呈现创面周边各穿支的位置、走形、直径等重要信息，方便术者进行比较从而选择最佳方案；（3）个性化显示穿支血管情况，避免了因血管缺失变异所致手术失败，同样也可以发现某些少见的优质穿支，为手术成功提供更多思路。（4）有助于术者制定多套手术方案，一旦首选穿支被破坏或不可用，可选择备选穿支实施备选方案。

5.3 为将“穿支血管地图”精确定位于体表，可使用以下三种方法：测量法、投影

(C)1994-2022 China Academic Journal Electronic Publishing House. All rights reserved. http://www.cnki.net

法、打印法。

5.3.1 测量法

通常采用“坐标法”，即在体表建立横纵坐标轴，将各穿支穿出点按照坐标值于体表进行描记。测量法已应用较多，最稳定，最易实施，也容易推广至头部、四肢等非躯体部位。其主要误差来源于：（1）坐标系建立误差；（2）测量误差。测量法需于VR模型表面和体表相同位置建立坐标系，在案例中我们选择胸部以平乳头连线为横轴、腹部以脐为坐标系原点建立坐标系，而实践中发现坐标系未能完全对应，原因可能在于：（1）患者双侧乳头并不严格处于同一水平面上；（2）影像检查时体位未能严格正躺，经肚脐垂线未能恰好通过耻骨前联合。在后续病例中我们考虑让病人严格躺正，以过胸骨中线、脐为纵轴建立坐标系，应该能减小误差。于VR模型上测得各点坐标值存在测量误差，将其于体表逐一描记也产生误差，原因在于：（1）个体操作因素；（2）体表为一曲面，而测量、描记的数值均为将其视为近似平面后的直线距离。因此我们采用三人分别独立进行测量描记，最终取平均值以减少误差。后续我们考虑于VR模型表面测量曲面距离，于体表描记时使用软尺，以减小误差，基于这一技术改进，可能能更好的适用于头部、四肢等曲率更大的部位。

5.3.2 投影法

通过便携式投影仪，将“穿支血管地图”垂直投射于体表。通过匹配定位装置和坐标系，可实现精确定位。该方法有良好的可视化效果，能于体表清晰显示血管走形、穿出点位置、与术区毗邻关系等信息。

投影法最大优点是直观、可视化强，不仅能描记穿支血管，还能显示主干血管、毗邻结构（如肿瘤、骨骼等），能帮助整形科医生整体把握术区的解剖关系，而且术中可继续投影，只要投影仪及病人的位置保持相对稳定，即使术中皮肤切开掀起，体表标记错位或擦除的情况下，仍能指导其深处的血管定位。其缺点为匹配性较差，定位够不稳定。

“穿支血管地图”为3D立体模型的正面投影截图，将其投影于体表相当于再次应用于3D物体表面，在转化过程中不可避免会产生误差。这提示我们可以尝试直接将3D穿支血管模型应用于术中，比如使用虚拟现实（VR，Virtual Reality）眼镜，尽管其定位匹配尚不够精确，但具有较强的研究潜力。

5.3.3 打印法

(C)1994-2022 China Academic Journal Electronic Publishing House. All rights reserved. http://www.cnki.net

我们将穿支血管地图以1:1的比例打印于透明塑料薄膜上，通过与定位三角匹配，将其与体表完全贴合，在穿支血管等感兴趣处戳点，即可用记号笔将其标注于皮肤。我们使用透明塑料膜为载体，通过戳孔，可将相应信息描记于体表，实践中我们发现打印法更适用于胸部等曲度较小的部位，且当区域内有较大的突起如体表巨大肿瘤、巨大乳腺等时，薄膜不易贴覆，该方法适用度不高。

打印法可视为投影法的进一步改进，一方面保留了其直观、可视化强、可显示主干血管及骨等结构的优点，另一方面避免了投射法的匹配不准确问题，定位好后可粘贴固定于体表，可重复性强。我们尝试使用了两种质地的塑料膜，厚者显示清晰，但质地硬，与皮肤贴合较差，薄者与皮肤贴合紧密，但可视化显示略差。与投影法相比，打印法的缺点之一是无法于术中实时定位，下一步我们考虑选择超薄的粘性贴膜，消毒后紧贴于皮肤表面，可直接于其上做手术切口，术中需要时只需对合皮肤，即可获取解剖定位信息。对于这一设想，选择于人体无毒的打印染料是一个重要考虑点。同投影法一样，打印法的一个主要误差来源是2D-3D转化后的信息偏倚。

三种定位方法各有优缺点，适用于不同情况。在今后的临床工作中值得进一步探索和改进。

6 结论

（1）通过选择合适的扫描序列和扫描参数，高分辨率MRA可使部分穿支血管显影，清晰度接近CTA，提示MRA在显示穿支血管中的价值可部分取代CTA，成为精确化皮瓣手术的又一重要工具。

（2）“穿支血管地图”可直观形象地显示术区各穿支血管位置、走形、毗邻关系等信息，为术者选择最佳穿支血管设计皮瓣提供重要依据，具有较强的临床应用价值。其数据来源不仅限于MRA，亦可来自CTA。

（3）三种定位方法（测量法、打印法、投影法）均可将“穿支血管地图”定位于体表，对术者制定最佳手术方案有提供指导。各方法均有不同的优缺点和适应范围，有待于临床的进一步探索改进。

（4）“穿支血管地图+体表精确定位”的方法，可以成为促进整形外科皮瓣手术进一步可视化、精确化的临床新思路。

(C)1994-2022 China Academic Journal Electronic Publishing House. All rights reserved. http://www.cnki.net

总　结

一、本研究创新之处

1、课题组提出利用数字化技术对体表肿瘤的影像学边界进行可视化显示，在此基础上进行扩大切除设计，避免了在肉眼手感边界上进行扩大切除设计的盲目性。

2、课题组提出利用数字化技术对穿支血管的位置进行可视化显示，为选择最佳穿支提供依据，避免了仅仅根据术者经验或单纯的血管探查检查进行穿支血管选择的盲目性。

3、首次提出“肿瘤切除导板”、“肿瘤地图”、“穿支血管地图”等概念，并进行了初步临床应用；

4、研发使用了基于 MRI 的体表“定位三角”；

5、对体表肿瘤标本进行了病理-大体-影像边界比对研究；

6、针对病情较复杂的体表肿瘤患者，我们推荐使用“肿瘤切除导板法”，临床思路为：“MRI 扫描→设计个性化肿瘤切除导板→3D 打印→手术应用”；或推荐使用“肿瘤地图投影法”，临床思路为：“MRI 扫描→设计个性化’肿瘤地图’→体表投影→手术应用“。以此提高体表肿瘤切除的精确度。

7 、对病情较复杂的拟行穿支皮瓣手术患者，我们推荐使用“穿支血管地图法”，临床思路为:“MRA 扫描→设计个性化’穿支血管地图’→体表定位（测量法、打印法、投影法）→手术应用”，可有助于术者设计最佳穿支皮瓣。

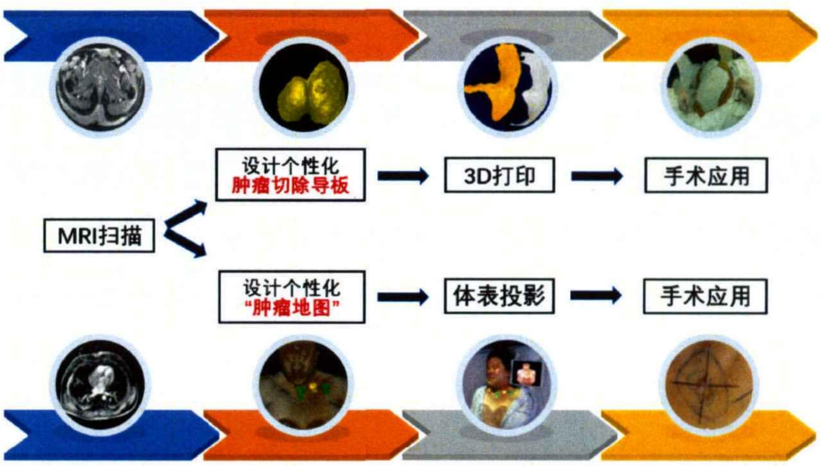

对复杂体表肿瘤患者推荐临床思路

(C)1994-2022 China Academic Journal Electronic Publishing House. All rights reserved.　http://www.cnki.net

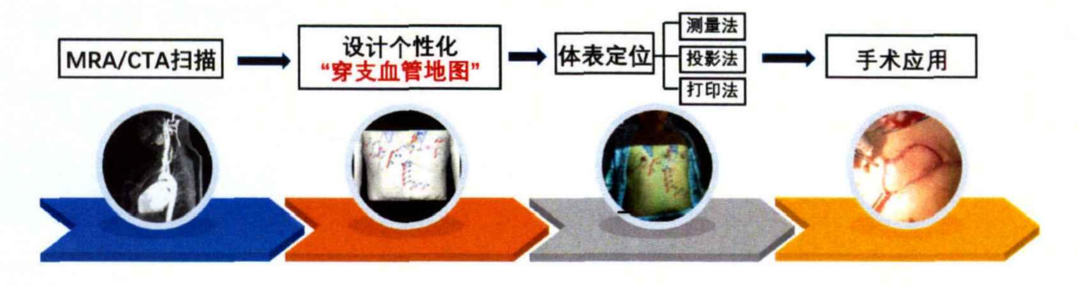

对复杂拟行穿支皮瓣手术患者推荐临床思路

二、本研究不足之处

1、本研究为临床方法学研究，目前初步建立了方法体系，纳入的病例数尚不足；

2、对切除标本进行病理检查的数目不足，统计学意义尚不足；

3、对体表肿瘤的病人没能详细统计愈后和复发情况，随访时间不足；

4、对穿支血管探查的评价不够细致，仅统计了数目，未统计全直径、距离等信息；

(C)1994-2022 China Academic Journal Electronic Publishing House. All rights reserved. http://www.cnki.net

参考文献

[1] 郝永红, 宋慧锋, 许明火. 隆突性皮肤纤维肉瘤诊断与治疗 [J]. 中国美容医学, 2013, 22(20): 2012-5.

[2] 韩新鸣. 常见体表非黑色素恶性肿瘤的形态学研究 [D]; 中国人民解放军医学院, 2017.

[3] 张良, 陈娜, 郑亮. 隆突性皮肤纤维肉瘤 21 例临床分析 [J]. 中国皮肤性病学杂志, 2018, 32(01): 38-41.

[4] 舒敬德, 杨君, 朱巧俐. 隆突性皮肤纤维肉瘤外科治疗和复发因素 [J]. 中国中西医结合外科杂志, 2011, 17(03): 308-10.

[5] Chaput B, Filleron T, Guellec S L. Dermatofibrosarcoma protuberans: Margins reduction using slow-Mohs micrographic surgery. Experience with 35 patients [J]. Annales de chirurgie plastique esthétique, 2014, 59(4):

[6] Mosterd K, Krekels G A, Nieman F H. Surgical excision versus Mohs' micrographic surgery for primary and recurrent basal-cell carcinoma of the face: a prospective randomised controlled trial with 5-years' follow-up [J]. Lancet Oncology, 2008, 9(12):

[7] 郭彩凤, 张涛, 宋田君. 3.0T 磁敏感加权成像(SWI)对脑部疾病的诊断价值分析 [J]. 影像研究与医学应用, 2018, 2(23): 88-90.

[8] 张丽, 韩立新, 曹惠霞. 3.0 T 磁共振扩散加权成像和 VIBRANT 动态增强在鉴别乳腺腺病与乳腺癌中的价值 [J]. 临床放射学杂志, 2017, 36(03): 342-6.

[9] Barral J K, Bangerter N K, Hu B S. In vivo high - resolution magnetic resonance skin imaging at 1.5 T and 3 T [J]. Magnetic Resonance in Medicine, 2010, 63(3):

[10] 刘华, 郝凯, 翟冬枝. 3.0T 磁共振多期增强扫描对胰腺癌术前诊断及手术可切除性评估中的价值 [J]. 中国 CT 和 MRI 杂志, 2019, 17(03): 90-3.

[11] 陆龙, 郑后珍. MRILAVA 动态增强序列联合 DWI 在乳腺良恶性肿瘤诊断中应用 [J]. 中外女性健康研究, 2018, 15): 24-5.

[12] 张彧, 吴安华, 韩帅. MRI 影像三维重建在垂体瘤质地的术前评估中的应用 [J]. 中国医科大学学报, 2019, 48(03): 220-4+9.

[13] 罗艳, 张吉琛. 基于 MRI 三维重建的正常眼部与眼眶肿瘤的软组织测量分析 [J]. 临床眼科杂志, 2018, 26(06): 510-3.

(C)1994-2022 China Academic Journal Electronic Publishing House. All rights reserved. http://www.cnki.net

[14] 肖海涛, 王怀胜, 刘晓雪. 应用游离皮瓣修复头皮恶性肿瘤术后缺损 18 例 [J]. 中国修复重建外科杂志, 2016, 30(01): 87-90.

[15] Farma J M, Ammori J B, Zager J S. Dermatofibrosarcoma protuberans: how wide should we resect? [J]. Annals of surgical oncology, 2010, 17(8): 2112-8.

[16] 丁煜昊. 三维可视化技术在神经外科的应用研究 [D]; 昆明医科大学, 2017.

[17] 范应方, 项楠, 蔡伟. 三维可视化技术在精准肝切除术前规划中的应用 [J]. 中华肝脏外科手术学电子杂志, 2014, 3(05): 8-11.

[18] 付军, 郭征, 王臻. 多种 3-D 打印手术导板在骨肿瘤切除重建手术中的应用 [J]. 中国修复重建外科杂志, 2014, 28(03): 304-8.

[19] 吴昆旻. 3D 打印技术在鼻鼻窦恶性肿瘤外科治疗中的应用 [D]; 第二军医大学, 2017.

[20] Hummelink S, Hameeteman M, Hoogeveen Y. Preliminary results using a newly developed projection method to visualize vascular anatomy prior to DIEP flap breast reconstruction [J]. Journal of plastic, reconstructive & aesthetic surgery : JPRAS, 2015, 68(3): 390-4.

[21] Nahabedian M Y. Overview of perforator imaging and flap perfusion technologies [J]. Clinics in plastic surgery, 2011, 38(2): 165-74.

[22] Kagen A C, Hossain R, Dayan E. Modern Perforator Flap Imaging with High-Resolution Blood Pool MR Angiography [J]. Radiographics : a review publication of the Radiological Society of North America, Inc, 2015, 35(3): 901-15.

[23] Cina A, Barone-Adesi L, Rinaldi P. Planning deep inferior epigastric perforator flaps for breast reconstruction: a comparison between multidetector computed tomography and magnetic resonance angiography [J]. Eur Radiol, 2013, 23(8): 2333-43.

(C)1994-2022 China Academic Journal Electronic Publishing House. All rights reserved. http://www.cnki.net

文献综述

核磁共振血管成像在腹壁下动脉穿支皮瓣手术中的应用进展

核磁共振血管成像（MRA）是临床探查血管的重要手段之一，研究表明 MRA 在腹壁下动脉穿支皮瓣（DIEP）手术中具有重要的应用价值。本文拟对 MRA 在腹壁下动脉穿支皮瓣手术中的应用进展做一综述。

一、腹壁下动脉穿支的影像学检查方法。

腹壁下动脉穿支（deep inferior epigastric perforator flap，DIEP）发自于腹壁下动脉（deep inferior epigastric artery，DIEA），穿过腹直肌支配腹壁组织。DIEP 皮瓣被认为是当前乳房再造的最新技术和最佳选择[1-3]，对穿支血管进行充分的影像学评估对于提高手术效率和改善愈后意义很大[4, 5]。

DIEP 穿支血管变异较大，手术的难点在于如何准确、快速、安全地解剖出最适合乳房再造的穿支血管。传统方式下医生通过术中解剖探查各穿支情况，综合评估后选择保留最佳穿支，耗时较长，对个人技术经验要求很高。合适的术前影像学检查可提高手术效率。常用穿支血管检查方法包括手持多普勒超声（US）、彩色多普勒超声（CDS）、CTA、MRA。其中 CTA 和 MRA 可提供穿支血管的详细信息，包括位置、直径、数量、走形等，能帮助外科医生进行良好的手术设计，减少不必要的解剖，缩短麻醉时间，减少并发症[5-8]。

选择穿支的首要因素是血管直径，管径越大可供养皮瓣面积越大，术后并发症越少。其次的选择因素是血管走形，在腹直肌内走形越短、越垂直者，分离越容易，对肌肉损伤越小。另外还应考虑腹壁下深动脉(DIEA)分支类型、与主要浅静脉的直接静脉连接、左右半腹间的浅静脉交通支等因素[1]。外科医师通常参考术前影像设计手术方案，但术中所见与术前规划并非完全一致，这是由于影像信息存在假阴性率，某些更粗大或走形更垂直的穿支可能未充分显影，术中术者根据具体情况可能改变既定方案，选择其他更合适的穿支。

二、MRA 对腹壁下动脉穿支皮瓣手术有指导价值

传统认为 MRA 分辨率低，较难显示小血管，但随着高分辨率核磁及相应序列的出现，MRA 显示穿支成为可能，部分国外学者对核磁指导 DIEP 皮瓣手术进行了研

(C)1994-2022 China Academic Journal Electronic Publishing House. All rights reserved. http://www.cnki.net

究[1,9]，认为MRA显示穿支血管具有独特的重要价值。当前CTA通常被认为是穿支血管探查的最佳选择，可以清晰显示直径超过1mm的穿支血管，且扫描时间很快，扫描范围可较大，病人不需要长时间憋气即可配合，临床应用较多，但其缺点也很明显，病人要承受电离辐射，并有碘过敏风险[4,5]。有研究表明，MRA和CTA两种血管造影技术对手术最佳穿支选择的指导价值相近。

术前MRA对血管成像的好处是提高手术效率。既往研究已经证实，DIEP术前行MRA影像评估均可缩短手术时间。Greenspun D等研究发现术前行MRA评估可缩短手术时间约66分钟[8]。国内的部分研究中选择以手术时间评价手术效率，以皮瓣存活率评价病人愈后，MRA组和CTA组结果无统计学差异，提示两种血管造影在提高手术效率、改善愈后方面价值相近。

术前MRA分析对选择手术方案和预估愈后具有一定参考价值。有学者认为通过充分的术前影像评估（如MRA/CTA），可选出不适合行DIEP手术者，因此腹部抽脂术史并不是DIEP再造乳房的绝对禁忌[10]。由此可见，对于腹壁情况较复杂的病人术前影像学检查（MRA）具有更重要的意义。

三、MRA **探查腹壁下动脉穿支血管的优势**

MRA主要优点体现在两方面：避免辐射和造影剂安全。

1、避免辐射。电离辐射会破坏细胞DNA，引起点突变和易位，这些突变均和癌症有关。一次腹部CT扫描通常要受到6-10 mSv的辐射量，相当于普通环境下三年的辐射总量[11]。另外有研究表明，乳腺癌患者罹患第二种癌症的风险可能更大，例如伴有癌基因（BRCA）突变的病人容易同时罹患乳腺癌和卵巢癌[12]，因此乳腺癌后寻求乳房再造者应尽可能避免腹部暴露于辐射，医生予CTA检查时应慎重，这也促使医生不断探索MRA等可替代CTA的穿支血管成像检查手段。

2、造影剂安全。CTA使用碘化造影剂，MRA使用含钆造影剂。统计表明，碘造影剂急性过敏反应的发生率为3%，钆造影剂的过敏反应发生率0.07%，相差几个数量级[13]。碘化造影剂即使在肾功能正常的患者中也可能引起肾功能不全，而钆造影剂仅对肾功能不全者诱发肾源性系统性纤维化，且比较罕见[14]。相较之下，MRA血管造影剂更安全。当然不论是行何种血管造影，提前进行充分的肾功能评估都必不可少。

(C)1994-2022 China Academic Journal Electronic Publishing House. All rights reserved. http://www.cnki.net

四、MRA 探查腹壁下动脉穿支血管的缺点

MRA 的主要缺点是分辨率较低，同 CTA 相比对穿支小血管成像能力较差。不使用对比剂的 MRA，即时间飞越法(time of flight，TOF)MRA 和相位对比(phase contrast，PC)MRA 对穿支血管无法显影。早期的三维动态增强磁共振血管成像（3DDCE-MRA）也效果不佳，Rozen WM 等使用 TIM（total image matrix）序列对 DIEP 患者进行了术前评估，发现 MRA 空间分辨率相对较低，对穿支血管成像能力不佳，对手术指导意义不及 CTA[15]。近年来随着核磁技术不断发展，对小血管的成像清晰度逐渐提高，可以显示直径 1mm 的穿支血管。如 Greenspun D 等使用 THRIVE (T1 High Resolution Isotropic Volume Examination)序列，对 DIEP 行术前评估，其显示穿支血管的假阳性率为 0%，即所有 MRA 显示的穿支血管均可于术中证实，假阴性率为 4%，50 个皮瓣中仅有 2 个血管未在 MRA 上显示[8]。Cina A 等通过前瞻性对比研究比较了 3D-LAVA 序列的 MRA 和 CTA 对 DIEP 血管成像的效果，结果二者对优势穿支血管的探查准确率均为 91.3%，提示在术前评价 DIEP 皮瓣方面 MRA 可部分替代 CTA[1]。Kagen AC 等使用 TRICKS (time resolved imaging of contrast kinetics)和 LAVA 序列对包括 DIEP 在内的全身多种皮瓣进行了血管造影研究，穿支血管均可显像清楚[9]。

MRA 的另一个主要问题是产生伪影。使用高分辨率三维梯度回波(LAVA-XV)不可避免会产生伪影，多分布于身体周围[9]。

五、MRA 探查腹壁下动脉穿支的改进

通过选择合适的扫描序列和优化扫描参数，当前高分辨率 MRA 对穿支血管的成像可基本满足临床需求。临床通常使用 3.0T 核磁的 3D-LAVA 序列进行腹部、臀部、胸部的穿支血管扫描，使用 TRICKS 序列对头部、四肢的穿支血管进行扫描，对数据进行 MIP、VR 重建分析，可清晰显示穿支位置及走形。LAVA 序列已被广泛应用于腹部脏器的血管成像研究，其扫描速度快，扫描范围广，具有良好的脂肪抑制效能，且显示血管的清晰度较高，可达到与 CT 相似的空间分辨率。因其在行 3D 容积扫描采集时，分别对 x、y、z 面采取相应的处理办法，包括减少频率方向值，采用加速因子 ASSET 技术和部分 K 空间采集技术，其使用的钆磷维塞三钠造影剂可以在血管内停留更长时间，提高了血管成像能力。

Kagen AC 等提倡对腹壁下动脉穿支使用俯卧位扫描，认为可以减少腹壁运动，稳定腹壁组织，从而减少磁敏感伪影。如扫描时间较长（大于 2 分钟），俯卧位不需

(C)1994-2022 China Academic Journal Electronic Publishing House. All rights reserved. http://www.cnki.net

要使用抗蠕动药物（如胰高血糖素）即可减少肠运动伪影[9]。国内部分研究则认为，俯卧位常导致腹部软组织变形严重，仍推荐采用仰卧位扫描，因与术中体位相同，更有利于穿支血管的体表定位。

总之，当前高分辨率 MRA 对腹壁下动脉穿支皮瓣手术有重要应用价值，其成像能力可与 CTA 接近，可为外科医生开展皮瓣手术提供帮助，同时避免了 CTA 承受电离辐射、使用碘造影剂的缺点，有着很强的临床应用价值。可以预见，随着核磁成像技术的不断发展和更多核磁序列的出现，MRA 对穿支血管的成像能力会越来越强，为临床医生提供更多有益帮助。

参考文献

[1] Cina A, Barone-Adesi L, Rinaldi P. Planning deep inferior epigastric perforator flaps for breast reconstruction: a comparison between multidetector computed tomography and magnetic resonance angiography [J]. Eur Radiol, 2013, 23(8): 2333-43.

[2] Tonseth K A, Hokland B M, Tindholdt T T. Quality of life, patient satisfaction and cosmetic outcome after breast reconstruction using DIEP flap or expandable breast implant [J]. Journal of plastic, reconstructive & aesthetic surgery : JPRAS, 2008, 61(10): 1188-94.

[3] De Greef C. [Breast reconstruction by DIEP free flap: about 100 cases] [J]. Annales de chirurgie plastique et esthetique, 2005, 50(1): 56-61.

[4] Rozen W M, Anavekar N S, Ashton M W. Does the preoperative imaging of perforators with CT angiography improve operative outcomes in breast reconstruction? [J]. Microsurgery, 2008, 28(7): 516-23.

[5] Rozen W M, Garcia-Tutor E, Alonso-Burgos A. Planning and optimising DIEP flaps with virtual surgery: the Navarra experience [J]. Journal of plastic, reconstructive & aesthetic surgery : JPRAS, 2010, 63(2): 289-97.

[6] Pinel-Giroux F M, El Khoury M M, Trop I. Breast reconstruction: review of surgical methods and spectrum of imaging findings [J]. Radiographics : a review publication of the Radiological Society of North America, Inc, 2013, 33(2): 435-53.

[7] Nahabedian M Y. Overview of perforator imaging and flap perfusion technologies [J]. Clinics in plastic surgery, 2011, 38(2): 165-74.

[8] Greenspun D, Vasile J, Levine J L. Anatomic imaging of abdominal perforator flaps without ionizing radiation: seeing is believing with magnetic resonance imaging

(C)1994-2022 China Academic Journal Electronic Publishing House. All rights reserved. http://www.cnki.net

angiography [J]. Journal of reconstructive microsurgery, 2010, 26(1): 37-44.

[9] Kagen A C, Hossain R, Dayan E. Modern Perforator Flap Imaging with High-Resolution Blood Pool MR Angiography [J]. Radiographics : a review publication of the Radiological Society of North America, Inc, 2015, 35(3): 901-15.

[10] De Frene B, Van Landuyt K, Hamdi M. Free DIEAP and SGAP flap breast reconstruction after abdominal/gluteal liposuction [J]. Journal of plastic, reconstructive & aesthetic surgery : JPRAS, 2006, 59(10): 1031-6.

[11] Brenner D J, Hall E J. Computed tomography--an increasing source of radiation exposure [J]. The New England journal of medicine, 2007, 357(22): 2277-84.

[12] Lemon J A, Phan N, Boreham D R. Multiple CT Scans Extend Lifespan by Delaying Cancer Progression in Cancer-Prone Mice [J]. Radiation research, 2017, 188(4.2): 495-504.

[13] Parfrey P. The clinical epidemiology of contrast-induced nephropathy [J]. Cardiovascular and interventional radiology, 2005, 28 Suppl 2(S3-11.

[14] Ragunatha S, Palit A, Inamadar A C. Nephrogenic fibrosing dermopathy [J]. Indian journal of dermatology, venereology and leprology, 2009, 75(1): 63-7.

[15] Rozen W M, Stella D L, Bowden J. Advances in the pre-operative planning of deep inferior epigastric artery perforator flaps: magnetic resonance angiography [J]. Microsurgery, 2009, 29(2): 119-23.

(C)1994-2022 China Academic Journal Electronic Publishing House. All rights reserved. http://www.cnki.net

攻读学位期间发表文章情况

[1] 栗利，张德康，韩岩，李梦露，张志辉，王惠英. 高分辨率 MRA 指导腹壁下动脉穿支皮瓣重建乳房的应用探讨 [J]. 中国医学影像学杂志, 2019, 27（6）：

[2] 陈犹白，张巍，栗利. 脂肪干细胞成肌分化及修复骨骼肌损伤：应用现状及未来研究方向［J］. 中国组织工程研究，2018，22(01)：126-32.

[3] 王一名，郭伶俐，栗利. 遗传性外毛根鞘囊肿的诊断及治疗一例 [J]. 中国美容整形外科杂志, 2019, 30(01): 63-4.

(C)1994-2022 China Academic Journal Electronic Publishing House. All rights reserved. http://www.cnki.net

致 谢

完成毕业论文意味着研究生生涯接近尾声，回顾三年时光，对恩师的感谢涌上心头。能入师门是我此生的荣幸。导师德高望重，在行业内口碑极佳；医技精湛，广受患者爱戴好评；治学严谨，繁重工作之余仍坚持躬耕科研；诲人不倦，对学生言传身教悉心指导。

导师经常鼓励我们要有创新意识。他常说，创新的最高层次是“无中生有”，从零开始创造一个新事物，需要长期的积累和灵感的火花，难度较大；而创新的第二个层次是“移花接木”，善于学习其他学科先进的技术应用到自己的领域，产生新的 idea，这也是创新。他鼓励我们多走出去，多与其他科室、辅诊科室，甚至是理工科交流，于融合中求创新。

导师鼓励我们在困难面前要努力坚持。他多年来一直有个心愿：临床上疑难复杂的体表肿瘤病人反复发作反复手术，怎样才能把肿瘤更精确地切除掉呢？这个问题临床意义很大，但要解决必须同病理科、影像科紧密协作，难度也很大，国内外相关的研究并不多。尽管如此他从未轻言放弃，多年来指导多位师兄师姐在病理和影像方向上进行探索研究，获得了一些突破性的研究成果。我曾经一度因为觉得课题太难而想放弃，但在他的鼓励之下坚持下来。正所谓念念不忘，必有回响，数字化技术的迅猛发展给我们以启迪，我们将其引入课题研究，不断摸索改进，终于对体表肿瘤切除提出了新的手术方法，为医生提供了新的临床思路。同时也在穿支皮瓣手术方面获得灵感，进行了相应研究。目前课题组初步建立了方法学体系，有待进一步的临床应用和改良。相信导师的心愿终有一天可以实现，让更多患者因此受益。

导师的言传身教让我不断成长，获益匪浅，期待在这样一位领路人的指引下，我终能成长为一名优秀合格的整形外科医生。

在此我还想真诚地感谢各位老师好友们，我的每一分进步都离不开你们的帮助和鼓励。

我想感谢各位领导：宋杰政委、张志辉主任、王惠英医生。感谢你们对我的支持鼓励。

我想感谢整形外科各位老师以及我的师兄弟姐妹：郭伶俐主任、陶然主任、雷永红主任、陈淼护士长、杨丽丽护士长、柴密医生、周志强医生、舒军医生、韩新鸣医

(C)1994-2022 China Academic Journal Electronic Publishing House. All rights reserved. http://www.cnki.net

生、马洪伟医生、陈召阳医生、姜伟乾医生、马超医生、李彦医生等。感谢你们对我学术和生活方面无私细致的指导帮助。

我想感谢影像科各位老师和朋友：程流泉主任、张爱莲主任、张德康医生、罗春才医生、闫飞医生等。感谢你们对我影像学方面无私细致的指导帮助。

我想感谢病理科各位老师和朋友：陈光勇主任、李金龙医生等。感谢你们对我病理学方面无私细致的指导帮助。

我想感谢 3D 打印公司各位老师和朋友：宿红刚经理、张国伟经理等。感谢你们对我数字化技术方无私细致的指导帮助。

我想感谢我的家人对我的支持、关怀和付出。

应感谢之人实多，请恕无法逐一列举。

硕士毕业为过往之结束，亦是未来之序曲。期待未来永葆初心，砥砺前行。

(C)1994-2022 China Academic Journal Electronic Publishing House. All rights reserved. http://www.cnki.net
