# Supplementary material for: Enhanced three-dimensional visualization reconstruction for perforator flaps: A case series on clinical applications and outcomes
Source: JPRAS Open. 2026 May 14;50:344–59. doi: 10.1016/j.jpra.2026.04.015 (PMC13240778; doi:10.1016/j.jpra.2026.04.015)
Supplement: Supplementary file 6 [file mmc6.docx]

STROBE Statement—checklist of items that should be included in reports of observational studies

|  | Item No. | Recommendation | Page  No. | Relevant text from manuscript |
| --- | --- | --- | --- | --- |
| **Title and abstract** | 1 | (*a*) Indicate the study’s design with a commonly used term in the title or the abstract | 1 | Enhanced Three-Dimensional Visualization Reconstruction for Perforator Flaps: A Case Series on Clinical Applications and Outcomes |
|  |  | (*b*) Provide in the abstract an informative and balanced summary of what was done and what was found | 2 | Methods: This case series evaluates the clinical applications and outcomes of enhanced three-dimensional visualization reconstruction for perforator flaps in 15 patients (16 flaps in total) treated between January 2021 and December 2024. |
| Introduction | | | |  |
| Background/rationale | 2 | Explain the scientific background and rationale for the investigation being reported | 3 | Perforator flaps, pioneered by Koshima et al. from 1989, are essential for complex soft tissue reconstruction… |
|  | 3 | State specific objectives, including any prespecified hypotheses | 3 | To address these limitations, this study introduces an enhanced 3D visualization protocol enabling accurate perforator localization and precise 3D vascular modeling for intelligent flap design |
| Methods | | | |  |
| Study design | 4 | Present key elements of study design early in the paper | 3 | To address these limitations, This retrospective case series introduces an enhanced 3D visualization protocol… |
| Setting | 5 | Describe the setting, locations, and relevant dates, including periods of recruitment, exposure, follow-up, and data collection | 3-4 | This study was conducted at two hospitals: Wuxi Ninth People’s Hospital Affiliated to Soochow University and Jiangsu University’s Affiliated Hospital. Data were collected from the medical records of 15 patients (16 flaps) with traumatic soft tissue defects who underwent perforator flap surgery using enhanced 3D visualization reconstruction between January 2021 and December 2024. Follow-up evaluations were conducted monthly for the first three months post-surgery, followed by periodic checks via clinic visits or WeChat. |
| Participants | 6 | (*a*) *Cohort study*—Give the eligibility criteria, and the sources and methods of selection of participants. Describe methods of follow-up  *Case-control study*—Give the eligibility criteria, and the sources and methods of case ascertainment and control selection. Give the rationale for the choice of cases and controls  *Cross-sectional study*—Give the eligibility criteria, and the sources and methods of selection of participants | 3-4 | This is a case series study.  Eligibility criteria: Data were collected from the medical records of 15 patients (16 flaps) with traumatic soft tissue defects who underwent perforator flap surgery using enhanced 3D visualization reconstruction.  Follow-up: Follow-up evaluations were conducted monthly for the first three months post-surgery, followed by periodic checks via clinic visits or WeChat. |
|  |  | (*b*) *Cohort study*—For matched studies, give matching criteria and number of exposed and unexposed  *Case-control study*—For matched studies, give matching criteria and the number of controls per case |  | Not applicable. |
| Variables | 7 | Clearly define all outcomes, exposures, predictors, potential confounders, and effect modifiers. Give diagnostic criteria, if applicable | 4-8 | Outcomes: Presented in part Ⅵ. Effectiveness Assessment. (page 7-8)  Exposures: Including optimized CTA scanning protocols, indirect extraction method for small vessel reconstruction, manual tracing of perforators, digital reverse flap design, and use of 3D-printed surgical guides. (page 4-6)  Diagnostic criteria:  ①Flap Survival: Using the "Blood Circulation Evaluation Index for Finger Replantation" from the Chinese Medical Association's Hand Surgery Society's functional assessment criteria.(page 7-8)  ②Hand and Foot Function: Using the Michigan Hand Outcomes Questionnaire (MHQ) for hand function and the AOFAS Ankle Hindfoot Scale for foot function.(page 8) |
| Data sources/ measurement | 8* | For each variable of interest, give sources of data and details of methods of assessment (measurement). Describe comparability of assessment methods if there is more than one group | 7-8 | Presented in part Ⅵ. Effectiveness Assessment. |
| Bias | 9 | Describe any efforts to address potential sources of bias | 3-4 | Data was collected from two hospitals to reduce selection bias that might arise from a single-center study. Data were independently collected and verified by experienced researchers to minimize information bias. In postoperative assessments, follow-up and outcome evaluations were conducted by researchers who were blinded to the study whenever possible. |
| Study size | 10 | Explain how the study size was arrived at | 3-4 | The study size was determined based on the availability of eligible patients who underwent perforator flap surgery using enhanced 3D visualization reconstruction between January 2021 and December 2024 at the two participating hospitals. A total of 15 patients (16 flaps) were included in the study. This retrospective case series included all eligible patients within the specified time frame to ensure a comprehensive evaluation of the clinical application and outcomes of the enhanced 3D visualization technique. |

Continued on next page

| Quantitative variables | 11 | Explain how quantitative variables were handled in the analyses. If applicable, describe which groupings were chosen and why | 8 | For normally distributed data, mean and standard deviation (mean ± SD) were used for description; for skewed data, median and interquartile range (median [Q1, Q3]) were used. For example, patient age (51 years, range 21-68 years) and wound area (94.3 cm², range 55.3-147.3 cm²) were presented in this manner. |
| --- | --- | --- | --- | --- |
| Statistical methods | 12 | (*a*) Describe all statistical methods, including those used to control for confounding | 8 | SAS 9.4 was used for all statistical computations. Present categorical data as counts (%). Assess normality of continuous data via Shapiro-Wilk test. For normally distributed data, use mean ± standard deviation [x ̅±s]. For skewed data, present as median (Q1, Q3). |
|  |  | (*b*) Describe any methods used to examine subgroups and interactions |  | None. |
|  |  | (*c*) Explain how missing data were addressed |  | Missing data were minimal in this study. |
|  |  | (*d*) *Cohort study*—If applicable, explain how loss to follow-up was addressed  *Case-control study*—If applicable, explain how matching of cases and controls was addressed  *Cross-sectional study*—If applicable, describe analytical methods taking account of sampling strategy |  | In this retrospective case series, loss to follow-up was minimal. |
|  |  | (*e*) Describe any sensitivity analyses |  | Not applicable. |
| Results | | | | |
| Participants | 13* | (a) Report numbers of individuals at each stage of study—eg numbers potentially eligible, examined for eligibility, confirmed eligible, included in the study, completing follow-up, and analysed | 4 | 15 patients (16 flaps) are included in the study. |
|  |  | (b) Give reasons for non-participation at each stage |  | Not applicable. |
|  |  | (c) Consider use of a flow diagram |  | Not applicable. |
| Descriptive data | 14* | (a) Give characteristics of study participants (eg demographic, clinical, social) and information on exposures and potential confounders | 15 | Presented in Table 1. Patient demographic and characteristics of Wound |
|  |  | (b) Indicate number of participants with missing data for each variable of interest |  | Missing data were minimal in this study. |
|  |  | (c) *Cohort study*—Summarise follow-up time (eg, average and total amount) | 19 | Presented in Table 3  Mean follow-up duration: 8 months (range 6-11 months).  Total follow-up duration: 120 months (15 patients, with an average follow-up of 8 months per patient). |
| Outcome data | 15* | *Cohort study*—Report numbers of outcome events or summary measures over time | 8-9 | Outcome event numbers: Among the 15 patients (16 flaps), all flaps were successfully transplanted. Of these, 15 flaps achieved complete survival, while 1 flap experienced partial necrosis.  Summary measures: The mean follow-up duration was 8 months (range 6-11 months). During the follow-up period, hand function assessment using the Michigan Hand Outcomes Questionnaire (MHQ) indicated that 6 out of 10 patients (60%) had good or excellent function; foot function assessment using the AOFAS Ankle Hindfoot Scale showed that all 5 patients had good or excellent function.  Other detailed descriptions of outcome data are presented in the Results section. |
|  |  | *Case-control study—*Report numbers in each exposure category, or summary measures of exposure |  | Not applicable. |
|  |  | *Cross-sectional study—*Report numbers of outcome events or summary measures |  | Not applicable. |
| Main results | 16 | (*a*) Give unadjusted estimates and, if applicable, confounder-adjusted estimates and their precision (eg, 95% confidence interval). Make clear which confounders were adjusted for and why they were included |  | Not applicable. |
|  |  | (*b*) Report category boundaries when continuous variables were categorized |  | Not applicable. |
|  |  | (*c*) If relevant, consider translating estimates of relative risk into absolute risk for a meaningful time period |  | Not applicable. |

Continued on next page

| Other analyses | 17 | Report other analyses done—eg analyses of subgroups and interactions, and sensitivity analyses |  | Not applicable. | 17 | Report other analyses done—eg analyses of subgroups and interactions, and sensitivity analyses |
| --- | --- | --- | --- | --- | --- | --- |
| Discussion | | | | |  |  |
| Key results | 18 | Summarise key results with reference to study objectives | 13 | Presented in the Conclusion section. |  |  |
| Limitations | 19 | Discuss limitations of the study, taking into account sources of potential bias or imprecision. Discuss both direction and magnitude of any potential bias | 13 | The limitations of this study include a small sample size, retrospective design, technical learning curve, subjective errors in manual operation steps, and relatively short follow-up duration. |  |  |
| Interpretation | 20 | Give a cautious overall interpretation of results considering objectives, limitations, multiplicity of analyses, results from similar studies, and other relevant evidence | 13 | Presented in the Conclusion section. |  |  |
| Generalisability | 21 | Discuss the generalisability (external validity) of the study results | 13 | Presented in the Conclusion section. |  |  |
| Other information | |  | | |  |  |
| Funding | 22 | Give the source of funding and the role of the funders for the present study and, if applicable, for the original study on which the present article is based | 14 | Presented in the Acknowledgement section. |  |  |

*Give information separately for cases and controls in case-control studies and, if applicable, for exposed and unexposed groups in cohort and cross-sectional studies.

**Note:** An Explanation and Elaboration article discusses each checklist item and gives methodological background and published examples of transparent reporting. The STROBE checklist is best used in conjunction with this article (freely available on the Web sites of PLoS Medicine at http://www.plosmedicine.org/, Annals of Internal Medicine at http://www.annals.org/, and Epidemiology at http://www.epidem.com/). Information on the STROBE Initiative is available at www.strobe-statement.org.
